# Pt^+^(C_2_H_2_)_*n*_ Complexes Studied with Selected-Ion Infrared Spectroscopy

**DOI:** 10.1021/acs.jpca.3c02734

**Published:** 2023-06-27

**Authors:** Anna G. Batchelor, Joshua H. Marks, Timothy B. Ward, Michael A. Duncan

**Affiliations:** Department of Chemistry, University of Georgia, Athens, Georgia 30602, United States

## Abstract

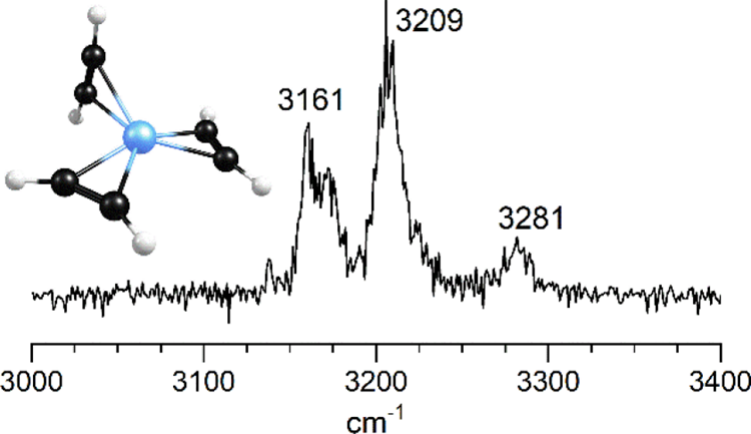

Platinum cation complexes with multiple acetylene molecules are studied with mass spectrometry and infrared laser spectroscopy. Complexes of the form Pt^+^(C_2_H_2_)_*n*_ are produced in a molecular beam by laser vaporization, analyzed with a time-of-flight mass spectrometer, and selected by mass for studies of their vibrational spectroscopy. Photodissociation action spectra in the C–H stretching region are compared to the spectra predicted for different structural isomers using density functional theory. The comparison between experiment and theory demonstrates that platinum forms cation−π complexes with up to three acetylene molecules, producing an unanticipated asymmetric structure for the three-ligand complex. Additional acetylenes form solvation structures around this three-ligand core. Reacted structures that couple acetylene molecules (e.g., to form benzene) are found by theory to be energetically favorable, but their formation is inhibited under the conditions of these experiments by large activation barriers.

## Introduction

The catalytic activity of platinum for hydrocarbon conversion reactions is well documented.^1−4^ Platinum catalysts, especially those with the metal incorporated into zeolites, are used widely in industry.^1−9^ Because of this relevance, the molecular details of the coordination and reactivity of platinum with a variety of small hydrocarbons, including olefins and acetylenes, have been investigated.^9−16^ In a series of studies, we have examined metal ion–acetylene complexes to explore cation−π bonding and possible metal-mediated catalytic processes.^17−31^ Platinum provides a potentially reactive system with an electronic structure contrasting to that of the metals studied previously (Cu, Ag, Au, V, Zn, Fe, and Ni). The present work reports the infrared (IR) photodissociation spectroscopy and computational chemistry for platinum cation–acetylene complexes of the form Pt^+^(C_2_H_2_)_*n*_ (*n* = 1–6). Vibrational spectroscopy reveals the coordination structures and allows evaluation of possible acetylene coupling reactions.

Metal ion–acetylene complexes have been investigated with mass spectrometry, computational chemistry, electronic spectroscopy, and IR spectroscopy.^17−55^ IR photodissociation spectroscopy measurements compared to the predictions of theory have provided the most detailed information about structures and bonding.^20−30^ Mono-acetylene complexes with a variety of metals form cation−π complexes, with metal ion binding in a symmetric position to the triple bond of acetylene. In these structures, the H–CC bond angle is changed, moving the hydrogen atoms away from the linear axis and away from the metal. Charge transfer from the acetylene to the metal shifts electron density, weakening the bonding in acetylene and lowering the C–H vibrational frequencies. Similar binding configurations and charge transfer occur for multi-acetylene complexes, with smaller vibrational frequency shifts as the charge transfer is distributed more widely. Multiple ligands also provide the possibility of isomeric structures, with reaction products coupling acetylene molecules lying at energies comparable to or even lower than unreacted structures. In the case of vanadium cation complexes, reactions form five-membered ring metallacycle complexes and eventually benzene.^26^ Similar structures are predicted for other metals but are not generally observed because of significant activation barriers to reactions. In zinc complexes, acetylene coupling reactions join molecules end-to-end, producing polyacetylene structures.^27^ Coordination spheres are generally filled with two (gold),^23^ three (copper),^24^ or four (nickel, silver, iron)^21,28,30^ acetylene ligands. Unreactive metals such as copper, silver, and gold form interesting solvation structures for complexes beyond these coordination spheres, with CH−π bonding between the inner and outer acetylene species.

In the present report, we use IR photodissociation spectroscopy of mass-selected ions to investigate the Pt^+^(C_2_H_2_)_*n*_ complexes up to and beyond the coordination sphere. Density functional theory (DFT) computations investigate structures and predict vibrational patterns for comparison to the experiments. We find that reactions coupling acetylene molecules are energetically favorable, but we do not detect the resulting products. Reactions are apparently inhibited by significant activation barriers under the conditions of our experiment.

## Methods

Ion–molecule complexes of the forms Pt^+^(C_2_H_2_)_*n*_ and Pt^+^(C_2_H_2_)_*n*_Ar_*m*_ were produced by laser vaporization in a pulsed-nozzle supersonic expansion of argon containing about 1% acetylene.^56^ The ions were analyzed and specific ions mass selected for study with a reflectron time-of-flight spectrometer designed for photodissociation experiments.^57,58^ Mass selection was accomplished with pulsed deflection plates using the flight time through the first flight tube of the instrument. Photodissociation takes place at the turning point in the reflectron field, and fragment mass analysis is accomplished using the flight time through the second flight tube section. Tunable IR radiation for these experiments was provided by a Nd:YAG-pumped optical parametric oscillator (OPO) laser system (LaserVision). Because the binding energies of acetylene ligands to platinum cations are generally greater than the IR photon energy, ion–molecule complexes were ″tagged″ with argon atoms to enhance photodissociation yields. IR excitation of acetylene vibrations leads to the loss of argon from these complexes. Larger complexes with multiple ligands fragment by losing acetylene. In each case, the yield of the fragment ion mass was recorded versus the IR photon energy to obtain the IR spectrum. Computational studies of complexes with or without argon were used to investigate the effects of argon attachment on the spectra.

Computational studies on the platinum cation–acetylene complexes were carried out with the Gaussian16 program package^59^ using DFT and the B3LYP functional with the ECP60MDF Stuttgart/Cologne pseudopotential,^60^ combined with the corresponding correlation consistent triple zeta basis set (cc-pVTZ-pp) for platinum. The cc-pVTZ basis set was used for C and H. IR frequencies from theory were scaled by a factor of 0.96 for comparison to experimentally measured spectra. Energetics were corrected for the non-scaled zero point energies.

## Results and Discussion

**Figure 1:**
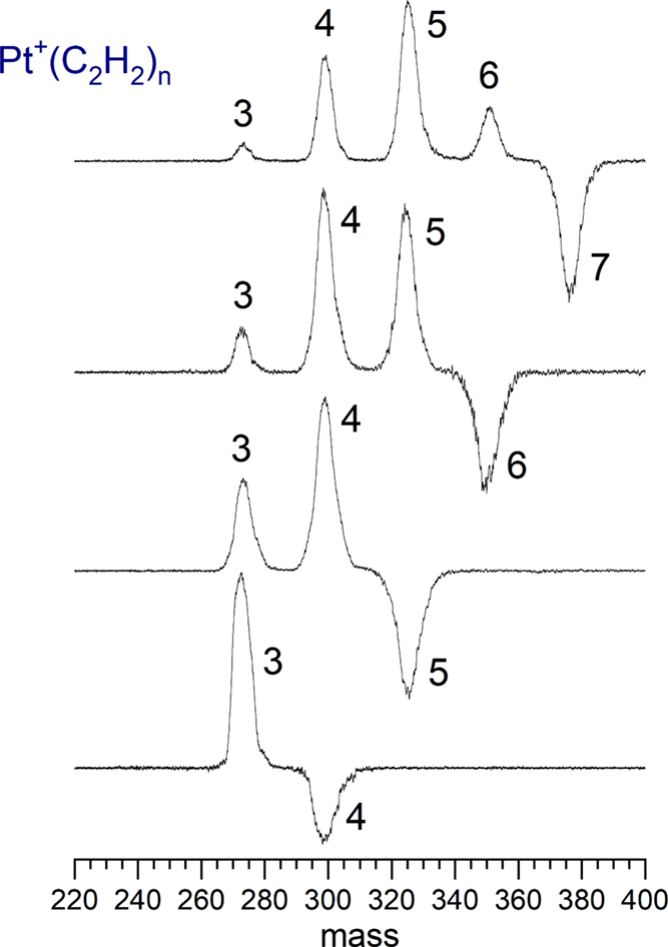
Fragmentation mass spectra of Pt^+^(acetylene)_*n*_ complexes, *n* = 7 (top) to *n* = 4 (bottom), with IR excitation. Weakly bonded external ligands are eliminated, but the *n* = 3 ion with the strongly bonded coordination sphere survives. In each case, the IR laser is tuned to the strongest resonance for that cluster in the 3100–3200 cm^–1^ range.

Laser vaporization of a platinum rod with an expansion gas containing about 1% acetylene in helium produces a distribution of cation-molecular complexes of the form Pt^+^(C_2_H_2_)_*n*_. A representative mass spectrum is presented as Figure S1 in the Supporting Information. When argon is added as the expansion gas, Pt^+^(C_2_H_2_)_*n*_Ar_*m*_ complexes are also formed. By selecting different Pt^+^(C_2_H_2_)_*n*_ complexes and tuning the IR laser through the C–H stretching region, we find no photodissociation of the smaller complexes (*n* ≤ 3), but photodissociation is detected for the larger complexes (*n* ≥ 4). Dissociation does not occur for the small complexes because the bond energies are greater than the photon energy in the C–H stretching region. The larger clusters photodissociate by losing one or more intact acetylene molecules because these complexes have additional acetylene molecules in the second coordination sphere that are more weakly bound. The fragmentation mass spectra for these larger complexes are shown in Figure 1. This data was collected in a difference mode of operation in which the mass spectrum of a selected ion without the dissociation laser is subtracted from that with it on. The negative peak indicates the depletion of the selected parent ion and the positive peaks indicate the photofragments produced. The mass peaks are broad because of the 194, 195, and 196 isotopes of platinum (abundances: 32.9, 33.8, 25.3%), which are of comparable abundance and not resolved. Each complex loses one or more acetylene molecules until it reaches the *n* = 3 complex, and then no further fragmentation occurs. The survival of the *n* = 3 complex indicates that three acetylene molecules form the strongly bonded coordination sphere around the Pt^+^ ion.

**Figure 2:**
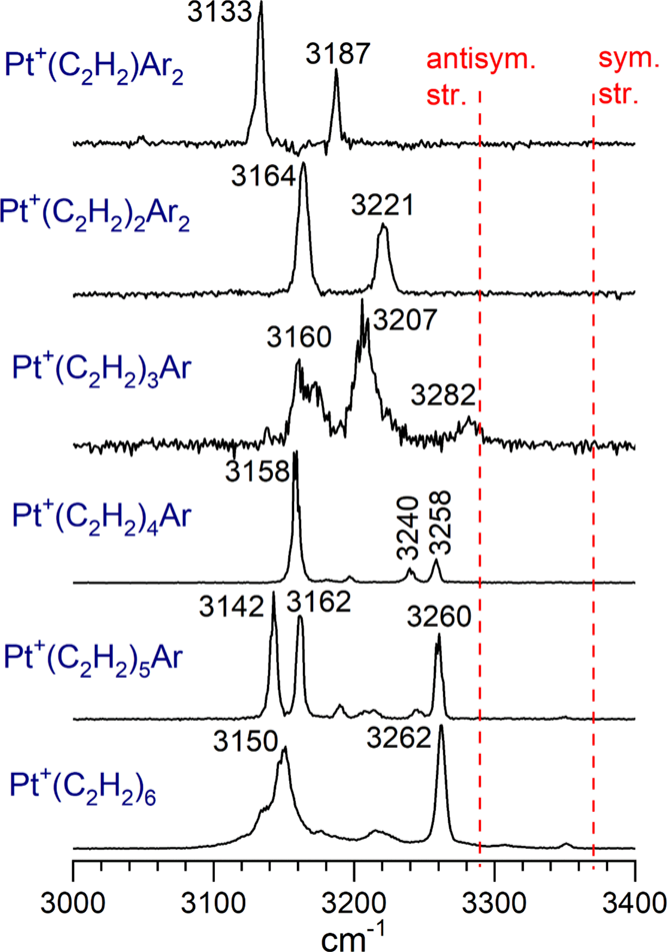
IR photodissociation spectra of Pt^+^(C_2_H_2_)_*n*_Ar_*m*_ and Pt^+^(C_2_H_2_)_*n*_ for *n* = 1–6. The *n* = 1–5 species are measured in the fragment ion channel corresponding to the loss of argon, whereas the *n* = 6 cluster was measured in the fragment ion mass channel corresponding to the loss of acetylene.

The IR photodissociation spectra obtained for the Pt^+^(C_2_H_2_)_*n*_ species (*n* = 1–6) are presented in Figure 2. The *n* = 1–5 complexes are measured via tagging with argon, whereas the *n* = 6 complex is recorded via the elimination of acetylene. The dashed red vertical lines indicate the positions of the symmetric and antisymmetric stretches of the isolated acetylene molecule at 3289 and 3374 cm^–1^, respectively.^61^ As shown, all the vibrational features for these platinum–acetylene complexes occur at frequencies lower than those for isolated acetylene. Such red-shifted vibrations for acetylene induced by metal binding have been seen for all the metal ion–acetylene complexes studied previously.^20−30^ Consistent with the Dewar-Chatt-Duncanson model of cation-molecular bonding,^62−65^ charge transfer shifts electron density from the acetylene to the metal, weakening the bonding in the acetylene and lowering its vibrational frequencies. The specific patterns in these C–H stretch vibrations are investigated by comparison to the predictions of DFT computations.

**Figure 3:**
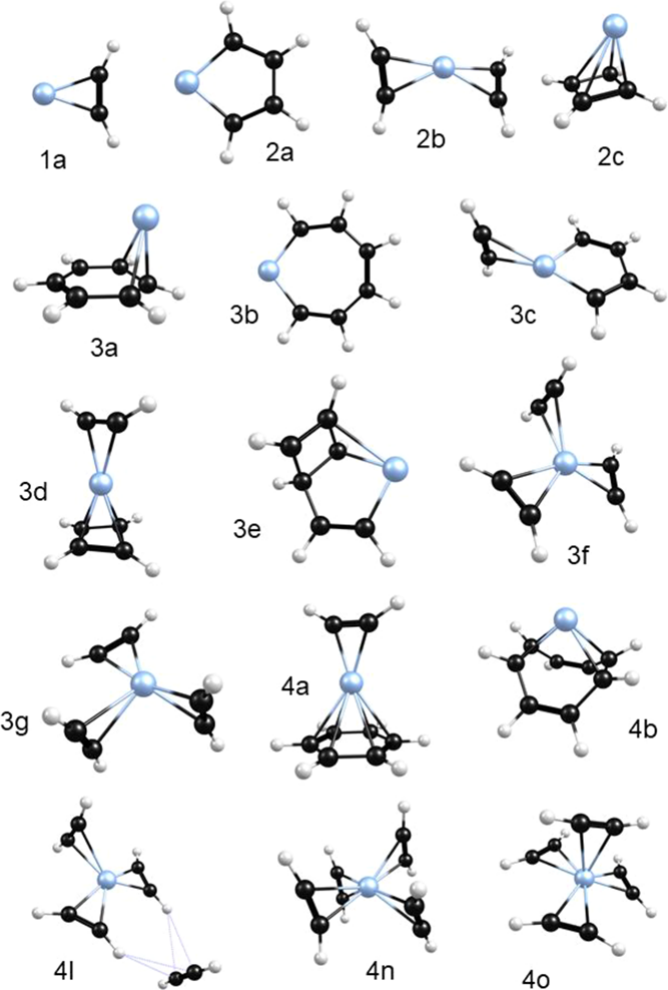
Lowest energy structures identified for Pt^+^(C_2_H_2_)_1–4_ resulting from DFT computations. A more complete collection of structures is presented in the Supporting Information.

Computational studies using DFT and an effective core potential appropriate for platinum were conducted on these Pt^+^(acetylene)_*n*_ complexes to determine their structures and to predict IR spectra corresponding to each structural isomer identified. These results are summarized in Table 1, and the full details are presented in the Supporting Information file. Figure 3 shows the lowest energy structures identified for the *n* = 1–3 cluster sizes, with selected structures for the *n* = 4 clusters. Each cluster size with multiple acetylene ligands has isomeric structures corresponding to both unreacted and reacted acetylenes. We investigated the effect of argon tagging on the IR spectra by computing structures and predicting IR spectra for tagged versus corresponding tag-free clusters for the *n* = 1 and 2 complexes (see multiple figures in the Supporting Information). We found that argon does not affect the structures in any significant way and induces only small frequency shifts (<10 to 20 cm^–1^) on the vibrations. Therefore, the spectra from theory presented below for each cluster size are those for the tag-free species.

**Table 1:** Energies (hartree), Relative Energies (kcal/mol), and Bond Dissociation Energies (BDE; kcal/mol) Computed for the Different Isomers of Pt^+^(C_2_H_2_)_*n*_ Complexes^a^

*n*	2S + 1	isomer	*E* (hartree)	Δ*E* (kcal/mol)	BDE (kcal/mol)
0	2		–119.082866	+0.0	
0	4		–119.055477	+17.2	
1	2	1a	–196.540703	+0.0	76.1
1	4	1b	–196.450071	+56.9	36.4
2	2	2a	–273.983334	+0.0	66.6
2	2	2b	–273.949982	+20.9	45.6
2	2	2c	–273.935422	+30.1	36.5
3	2	3a	–351.433277	+0.0	92.1^b^
					73.7^c^
3	2	3b	–351.406341	+16.9	75.2
3	2	3c	–351.36955	+40.0	52.1
3	2	3d	–351.360337	+45.8	46.3
3	2	3e	–351.350917	+51.7	40.4
3	2	3f	–351.314674	+74.4	17.7
3	2	3g	–351.312461	+75.8	16.3
4	2	4a	–428.859319	+0.0	130.6^b^
					53.7^c^
4	2	4b	–428.824796	+21.7	108.9
4	2	4l	–428.656995	+127.0	3.6
4	2	4n	–428.647886	+132.7	–2.1
4	2	4o	–428.643971	+135.1	–4.6

a BDE values are for the elimination of acetylene, except as noted.

b Elimination of acetylene.

c Elimination of benzene.

**Figure 4:**
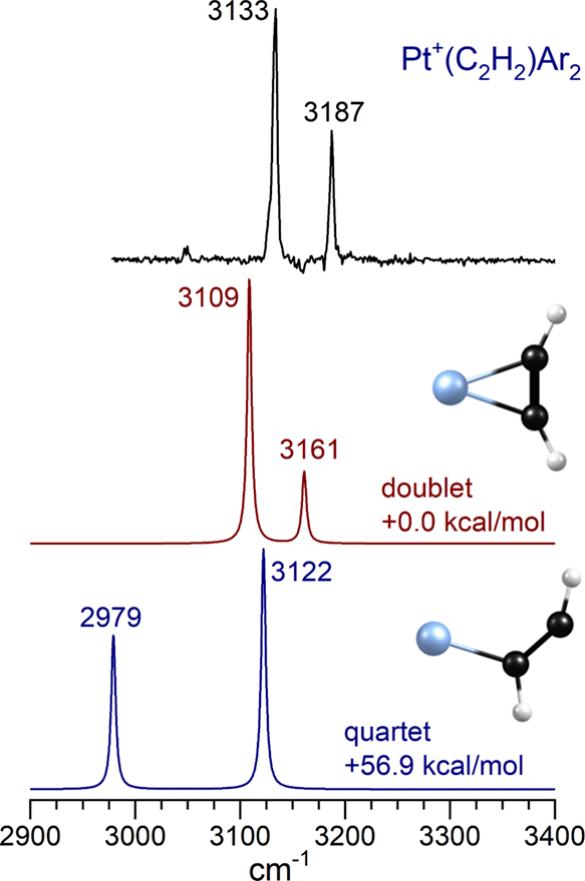
IR spectrum of Pt^+^(C_2_H_2_)Ar compared to the predictions of theory for the lowest lying doublet and quartet species of Pt^+^(C_2_H_2_).

Figure 4 shows the IR spectrum of the Pt^+^(C_2_H_2_)Ar_2_ complex compared to the spectra predicted by DFT for the most stable isomer of the doublet spin state and that of the quartet spin state. The experimental spectrum has two bands at 3133 and 3187 cm^–1^, shifted, respectively, by 156 and 187 cm^–1^ to lower frequencies from the positions of the free-acetylene vibrations at 3289 and 3374 cm^–1^. As indicated, the doublet state lies at lower energy and the quartet spin state lies much higher in energy. The doublet-quartet energy difference from theory in this complex is much greater than the known experimental spacing for the isolated platinum atomic cation (^2^D–^4^F spacing of 13.7 kcal/mol),^166^ and the same is found for other larger complexes. We therefore eliminate the quartet states from further discussion. As shown, the doublet has a cation−π structure with two IR bands matching the experiment reasonably well, although both of the predicted bands are slightly (∼20 cm^–1^) lower in frequency than those in the experiment. We therefore conclude that the structure predicted by theory is qualitatively correct and that the two bands observed are the antisymmetric and symmetric stretches of the acetylene ligand bound to Pt^+^.

**Figure 5:**
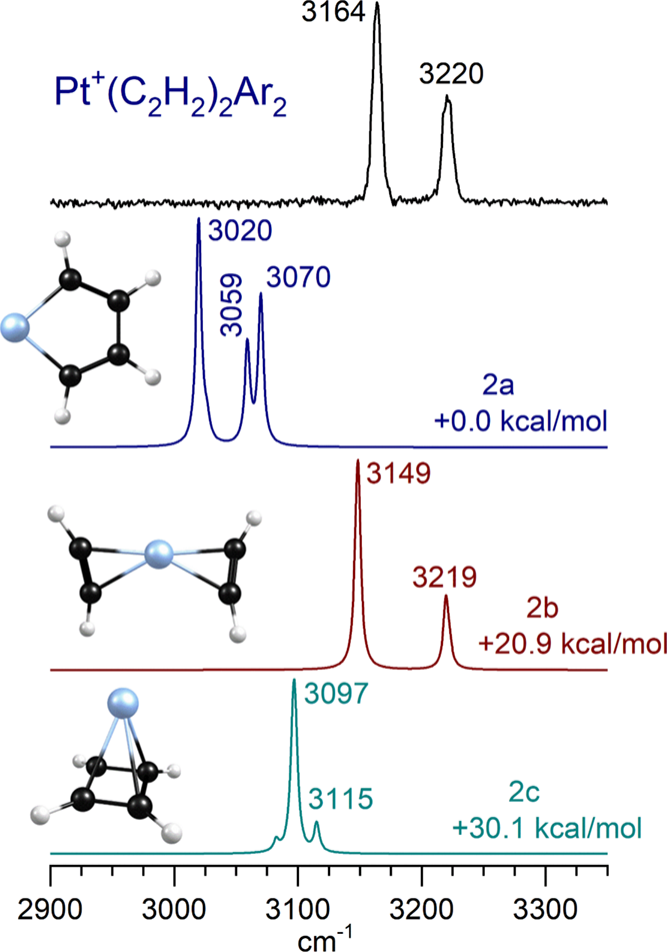
Infrared spectrum of Pt^+^(C_2_H_2_)_2_Ar compared to the predictions of theory for the three lowest lying doublet isomers of Pt^+^(C_2_H_2_)_2_.

Figure 5 shows the IR spectrum measured for Pt^+^(C_2_H_2_)_2_Ar_2_ compared to the predictions of theory for different isomeric structures possible for this ion composition. Again, the experiment has two bands at 3164 and 3220 cm^–1^, which are 125 and 154 cm^–1^ to the red from the acetylene vibrations. These red shifts are smaller than those seen for the *n* = 1 complex, consistent with the charge-transfer interaction being reduced because of delocalization over multiple ligands. Theory now identifies three main isomers, an unreacted species (2b) with two separate acetylene molecules, and two reacted isomers with metallacycle (2a) and cyclobutadiene (2c) structures, respectively. The spectrum for the unreacted isomer 2b matches the experiment, whereas those for the reacted isomers do not, even though reacted isomer 2a is predicted by theory to be the most stable structure. Isomer 2b has an asymmetric bow-tie structure with unequal Pt–C distances for the two carbons on each ligand.

**Figure 6:**
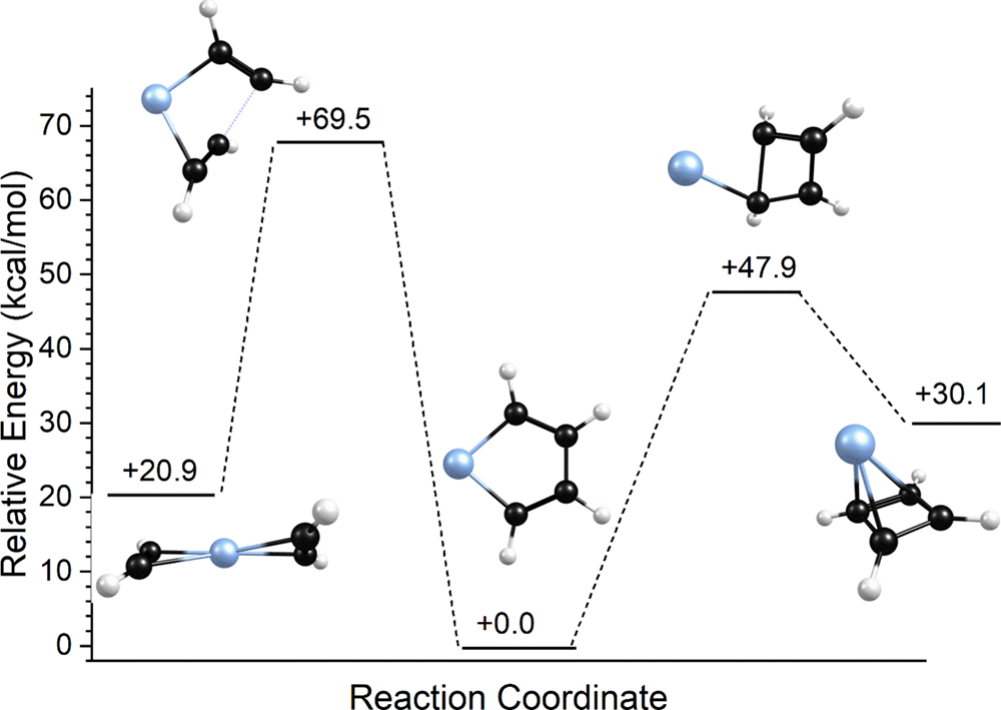
Reaction coordinate for the Pt^+^(C_2_H_2_)_2_ system.

The apparent lack of reactivity in this system can be understood by referring to the reaction coordinate shown in Figure 6. As indicated, computations make it possible to identify not only the relative energies of structural isomers but also the reaction barriers between isomers on the potential energy surface. As shown, isomer 2b likely represents the structure of the initial encounter complex formed by adding a second acetylene to the Pt^+^(C_2_H_2_) complex. There is a significant activation energy of about 49 kcal/mol separating this structure from the more stable reacted structure 2a. It is easy to see then that isomer 2b would not rearrange to isomer 2a except at very high temperature. Isomer 2c is less stable, but as shown it is also separated from 2a by a significant barrier. It therefore makes sense that our experiment produces primarily the unreacted isomer 2b.

**Figure 7:**
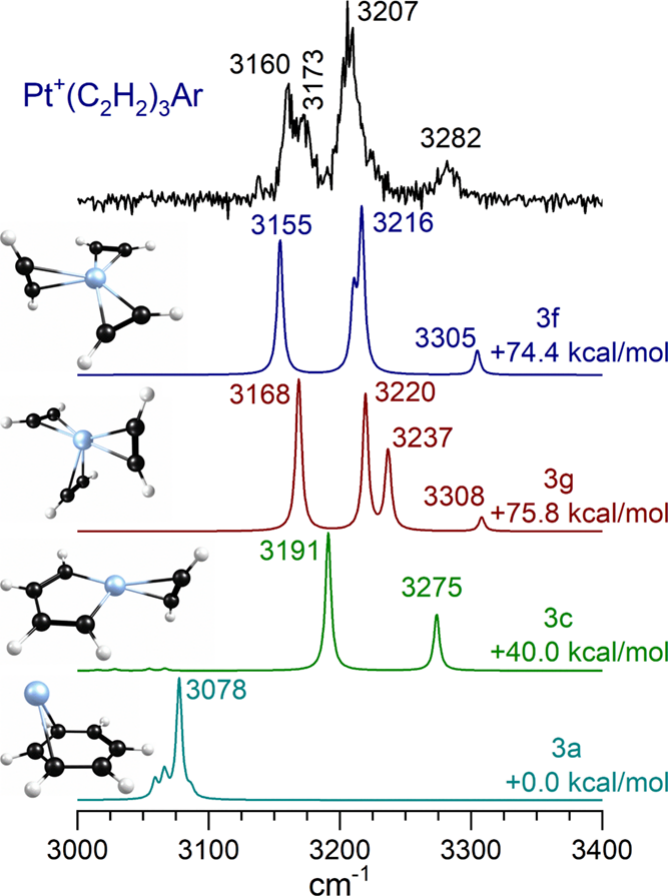
IR spectrum of Pt^+^(C_2_H_2_)_3_Ar compared to the predictions of theory for four selected doublet isomers of Pt^+^(C_2_H_2_)_3_.

Figure 7 shows the IR spectrum measured for Pt^+^(C_2_H_2_)_3_Ar compared to the predictions of theory for low-lying isomers 3a, 3c, 3g, and 3f. The spectra of other isomers are presented in the Supporting Information. As shown in Figure 2, the two main bands in this spectrum fall at almost the same frequencies as those for the *n* = 2 complex, but with the addition of a much weaker band at 3282 cm^–1^. The most stable structure is the platinum–benzene complex, isomer 3a, which could be formed by a cyclotrimerization reaction. As shown in Figure 3, the structure of Pt^+^(benzene) has the metal ion over the edge of the ring and not on the sixfold axis. Previous work at lower levels of theory found the C_6v_ structure,^66,67^ whereas the present structure is more like that found previously for Ag^+^(benzene).^68^ Isomer 3c has a metallacycle structure like that of isomer 2a but with an extra acetylene binding opposite the ring. Isomers 3f and 3g are both unreacted species with three separate acetylene molecules coordinated individually to the platinum ion. They differ in the angular orientation of the acetylenes. 3f has two acetylenes in a near-planar configuration with one perpendicular to the plane, whereas 3g has one acetylene defining a plane containing the metal and two acetylenes perpendicular to this. Again, the unreacted isomers lie at much higher energies than reacted ones, but isomer 3f has a spectrum best matching the experiment. The two more intense bands seen here for the *n* = 3 complex fall at almost the same positions as the two bands seen for the *n* = 2 complex. According to theory, these bands for the *n* = 3 complex arise from the same kinds of symmetric and antisymmetric C–H stretches of the near-planar bonded acetylenes seen for the *n* = 2 complex, with the higher frequency of these at 3207 cm^–1^ overlapped by the antisymmetric stretch of the single out-of-plane molecule. The higher frequency band at 3282 cm^–1^ is from the symmetric stretch of the single out-of-plane molecule. These higher frequencies (i.e., less red-shifted) for the out-of-plane acetylene show that it more weakly interacts with the platinum ion than the other two ligands. Weaker peaks in this spectrum suggest that there could be a minor contribution from co-existing isomer 3g, consistent with its energetic proximity to 3f.

The 3f isomer suggested by the IR patterns has two distinct kinds of acetylene ligands. Those in the near-planar configuration have similar structures and bonding, each with Pt–C bond distances of 2.079 and 2.086 Å. The out-of-plane acetylene has much longer Pt–C bond distances of 2.387 and 2.405 Å. Likewise, the bond energies are quite different, with a binding energy for the third (out-of-plane) ligand in the *n* = 3 complex of 17.7 kcal/mol compared to the value of 45.6 kcal/mol for the second acetylene in the *n* = 2 complex. It thus appears that the first two acetylenes are much more strongly bonded than the third and that the fourth (see below) is much more weakly bonded than the third. The third ligand is apparently bound strongly enough to avoid fragmentation with the IR laser tuned to the C–H stretches, as shown in Figure 1. The C–H stretch frequency corresponds to an energy of about 9 kcal/mol, and the computed binding energy of the third acetylene is 17.7 kcal/mol, consistent with this. This coordination behavior is somewhat similar to the situation we documented previously for the Au^+^(C_2_H_2_)_*n*_ complexes,^24^ which had a strongly bonded coordination of two and a secondary coordination of three ligands. Counting acetylene as a four-electron donor, which is often the case in organometallic chemistry,^2−4^ Au^+^(C_2_H_2_)_2_ is an 18-electron complex, explaining its stability. Pt^+^(C_2_H_2_)_2_ is a 17-electron species, almost reaching the 18-electron configuration, perhaps explaining its relative stability. Pt^+^(C_2_H_2_)_3_ has an additional ligand beyond this, whose full electron density is not needed to achieve the 18-electron configuration, and therefore, this acetylene is more weakly bonded. However, it is not obvious why it occupies such an unusual binding configuration.

**Figure 8:**
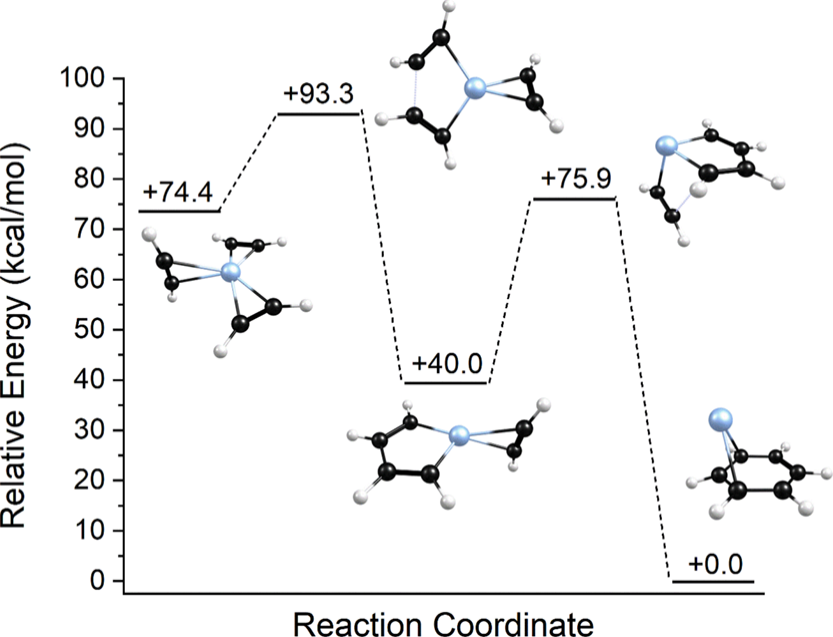
Reaction coordinate for the Pt^+^(C_2_H_2_)_3_ system.

A consideration of the reaction coordinate for the *n* = 3 complex, as shown in Figure 8, explains why the unreactive isomers are detected. The unreacted isomer 3f is a logical starting point for consideration of reactions since this structure is likely to form when a third acetylene encounters the unreacted Pt^+^(C_2_H_2_)_2_ complex (isomer 2b, which is indicated by spectroscopy to be the structure present for the *n* = 2 species). As indicated in the reaction coordinate figure, structure 3c is connected to 3f via a transition state bringing two acetylenes together, but this transition state lies almost 19 kcal/mol higher in energy than isomer 3f. This activation barrier is apparently too high to be accessed under the conditions of our experiment. If isomer 3c were formed, the barrier connecting this to the most stable isomer 3a would lie at about the same energy as the entrance channel, but this reaction does not proceed because isomer 3c does not form. Therefore, the formation of Pt^+^(benzene) is highly exothermic from Pt^+^(C_2_H_2_)_3_, but it is kinetically inhibited. This same kind of behavior was recently documented for Fe^+^(C_2_H_2_)_*n*_ complexes.^30^

**Figure 9:**
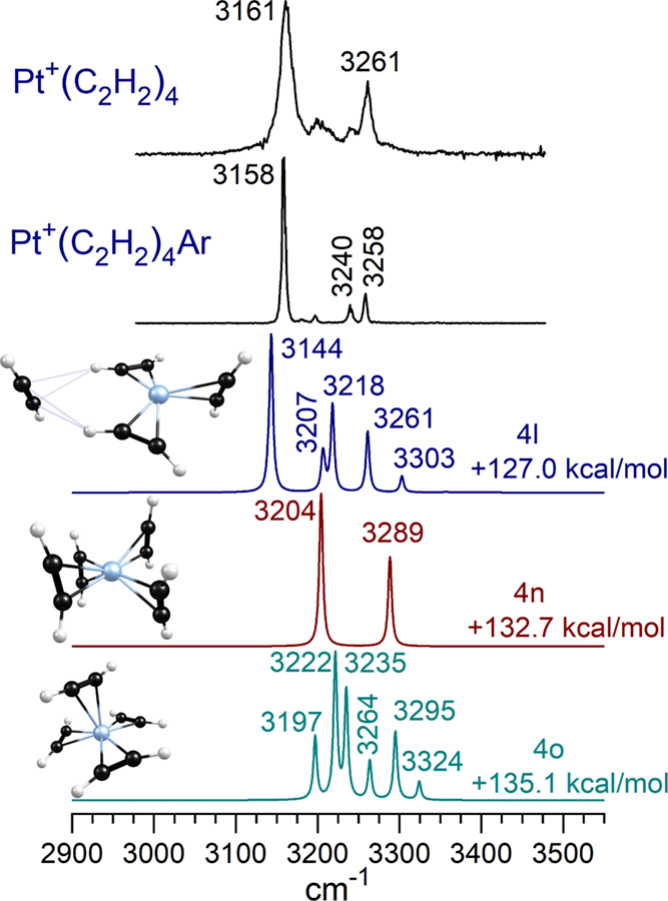
Infrared spectrum of Pt^+^(C_2_H_2_)_4_ and Pt^+^(C_2_H_2_)_4_Ar compared to the predictions of theory for three selected doublet isomers of Pt^+^(C_2_H_2_)_4_.

Figure 9 shows the IR spectra obtained for Pt^+^(C_2_H_2_)_4_ with and without argon tagging. Except for differences in linewidths, the spectra are almost identical, indicating that the argon binding has an insignificant effect on the structure of this ion and its spectrum. The many different isomers identified for this cluster size are described in the Supporting Information; Figure S55 shows their structures and Table S24 presents their relative energies. The spectra for three selected isomers are shown in Figure 9 for comparison to the experiment. Although none agree perfectly, isomer 4l has a spectrum most similar to the patterns in the experiment. It has an inner coordination of three unreacted acetylenes, in a configuration much like that of isomer 3f, which is the species detected in the experiment for the *n* = 3 cluster. Addition of a single additional second-sphere acetylene produces a 3 + 1 structure. The second-sphere molecule is bound to two inner-sphere ligands via CH−π hydrogen bonds. This kind of “solvation” interaction was found previously for the second-sphere ligands of other metal ion–acetylene complexes.^23,24,28^ According to theory, the most intense IR bands are those from the strongly coordinated inner-sphere ligands. The external acetylene has bands that are less shifted from those of acetylene and also less intense. Isomer 4n has a structure close to square-planar, which is of course the familiar configuration for many neutral d^8^ platinum complexes.^1−4,9,10^ Its two-band spectrum matches the qualitative pattern in the experiment, but the band shifts from isolated acetylene are much less than those measured. Isomer 4o has a near-tetrahedral structure, with a spectrum much more complex than that in the experiment. Because of the poor agreement with theory and the weaker bands in the experiment, minor concentrations of these isomers other than 4l cannot be ruled out.

**Figure 10:**
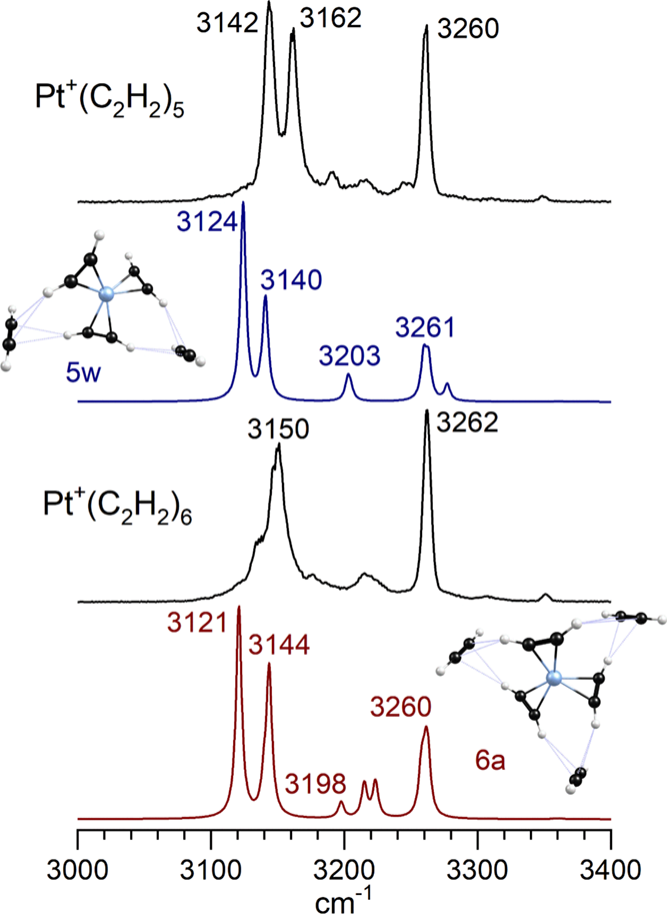
Spectra for Pt^+^(C_2_H_2_)_5_Ar and Pt^+^(C_2_H_2_)_6_ compared to the spectra predicted by theory for two selected isomers.

The number of isomeric structures possible for these systems increases dramatically with cluster size. Therefore, we have identified many more isomers for the *n* = 5 complexes than for the *n* = 4 species. These include many reacted and unreacted structures, as detailed in the Supporting Information. For the *n* = 6 complexes, we have focused only on selected unreacted structures since the data for smaller clusters seems to favor such species. Therefore, our comparison of experiment to theory for the *n* = 5 and 6 cluster sizes is not complete, but as shown in Figure 10, we have found structures whose spectra agree reasonably well with the patterns in the experiment. In these cases, unreacted structures with a three-ligand core ion and either two or three solvating external acetylene molecules seem to be consistent with the experimental spectra. The Pt^+^(C_2_H_2_)_5_ complex was measured both with and without argon tagging. Its tagged spectrum (Figure 2) has sharp bands at 3142, 3162, and 3260 cm^–1^, which become slightly broader in the tag-free spectrum shown here in Figure 10. The Pt^+^(C_2_H_2_)_6_ complex was only measured without tagging. Its spectrum has a broader band near 3150 cm^–1^, with a weak shoulder on the high energy side that roughly matches the positions of the 3142 and 3162 cm^–1^ bands seen for Pt^+^(C_2_H_2_)_5_. A higher energy band at 3262 cm^–1^ matches the 3260 cm^–1^ band seen for Pt^+^(C_2_H_2_)_5_. The similarity of these two spectra is clear. As shown in the figure, the predicted spectra for the Pt^+^(C_2_H_2_)_5_ complex with two external acetylene molecules and that for the Pt^+^(C_2_H_2_)_6_ complex with three external acetylenes match the experimental spectra. The intense bands near 3140–3160 cm^–1^ come from the core acetylene molecules involved in CH−π hydrogen bonds with the external molecules, whereas the 3260 cm^–1^ band is from the antisymmetric stretches of the external molecules. This latter feature is only slightly red-shifted from the antisymmetric stretch in acetylene itself. A very weak band for both complexes occurs near 3350 cm^–1^, which is the symmetric stretch of the external molecules. It is also close to the symmetric stretch of isolated acetylene. The bands near 3150 cm^–1^ are therefore the signature for the hydrogen bonding of confined acetylene molecules and the band near 3260 cm^–1^ is the signature for the external, solvating acetylene molecules. Although the experiment does not have enough detail to confirm this, theory indicates that the three-ligand core ion in these solvated structures has gradually evolved to have equivalent ligands. Apparently, the CH−π bonding configurations are more favorable in this symmetric structure. A similar effect was noted previously for the Cu^+^(C_2_H_2_)_*n*_ and Au^+^(C_2_H_2_)_*n*_ complexes.^23,24^

The experimental spectra and the comparison to the predictions of theory make it possible to determine the overall picture of platinum ion interactions with acetylene molecules in these small clusters. Although cyclization reactions are found by theory to be energetically favorable, producing structures such as metallacycles, metal-cyclobutadienes, or metal-benzenes, there is no compelling evidence in IR spectroscopy for any such reacted structures. Instead, all cluster sizes exhibit coordination to and solvation of platinum ions by intact acetylene molecules. The coordination is completed with three acetylenes in an unanticipated structure having two equivalent and one distinct binding configuration. This core ion forms the basis for the structure of larger clusters, which add external acetylenes attached via CH−π hydrogen bonding. Three of these solvating acetylene molecules complete a secondary solvation shell. The unreacted structures that we detect all lie higher in energy than many possible reacted structures involving ligands linked in metallacycle, cyclobutadiene, or benzene structures. We show in the smaller clusters that such reactions are kinetically inhibited by significant activation barriers, and it is logical to assume that similar barriers inhibit reactions in the larger clusters. Under the conditions of our experiment, coordination and solvation of platinum cations with acetylene take place instead of ligand-coupling reactions.

## Conclusions

Platinum–acetylene ion–molecule complexes containing up to six acetylene ligands have been produced in a supersonic molecular beam, identified and selected by mass, and studied with IR laser photodissociation spectroscopy. The IR experiments are complemented by computational quantum chemistry at the DFT level. Platinum ions in these Pt^+^(C_2_H_2_)_*n*_ complexes bind to acetylene molecules via cation−π bonding. A symmetric structure is identified for the mono-acetylene complex, but the *n* = 2 and *n* = 3 complexes have asymmetric structures. The *n* = 3 complex has two equivalent and one distinct ligand binding configuration, completing the coordination sphere. This core ion forms the basis for larger clusters which grow by adding external acetylene molecules bound via CH−π bonding. Although cyclization reactions are found by theory to be energetically favorable, producing a variety of structures such as metallacycles, metal-cyclobutadienes, or metal-benzenes and their combinations, there is no compelling evidence in IR spectroscopy for such structures. Computational studies of reaction potential energy paths find significant activation barriers, explaining the lack of reactivity for these platinum systems. In previous work, certain metal ion systems (V^+^ and Zn^+^) were able to initiate acetylene coupling reactions,^26,27^ but similar unreactive behavior was documented for other transition metal ions (Cu^+^, Ag^+^, Au^+^, and Fe^+^).^23,24,28,30^ It is known from industrial chemistry that acetylene cyclization to form benzene involves significant reaction barriers, and apparently, these same issues affect the chemistry in small gas-phase clusters.

## Supporting Information Available

Full citation for ref (59), sample mass spectrum of the clusters produced, and details of the DFT computations done, including the structures and energetics for each of the cluster ions considered (PDF)
