# Supplementary material for: Pt+(C2H2)n Complexes Studied with Selected-Ion Infrared Spectroscopy
Source: J Phys Chem A. 2023 Jun 27;127(27):5704–12. doi: 10.1021/acs.jpca.3c02734 (PMC10350954; doi:10.1021/acs.jpca.3c02734)
Supplement: Supplementary file 1 — jp3c02734_si_001.pdf [file jp3c02734_si_001.pdf]

Supporting Information

$Pt^+(C_2H_2)_n$ Complexes Studied with Selected-Ion Infrared Spectroscopy

Anna G. Batchelor,[1] Joshua H. Marks,[1] Timothy B. Ward,[1] and Michael A. Duncan[1]*

[1]Department of Chemistry, University of Georgia, Athens, GA 30602

*Email: maduncan@uga.edu

Table of Contents

Gaussian16 full reference	S3
Figure S1: Mass spectrum of $Pt^+(C_2H_2)_n$	S4
**(C_2H_2)**	
Table S1: energy and Figure S2: structure	S5 – S6
**Pt^+**	
Table S2: relative energies	S7
**$Pt^+(C_2H_2)$ & $Pt^+(C_2H_2)Ar_2$**	
Tables S3 – S4: relative energies	S8 – S9
Figures S3 – S14: cartesian coordinates & unscaled vibrational frequencies	S10 – S21
Figures S15 – S18: simulated spectra	S22 – S25
**$Pt^+(C_2H_2)_2$ & $Pt^+(C_2H_2)_2Ar_2$**	
Tables S5 – S6: relative energies	S26 – S27
Figures S19 – S39: cartesian coordinates & unscaled frequencies	S28 – S48
Figures S40 – S46: simulated spectra	S49 – S55
**$Pt^+(C_2H_2)_3$**	
Tables S7 – S8: relative energies	S – S
Tables S41 – S78: cartesian coordinates & unscaled vibrational frequencies	S – S
Figures S79 – S86: simulated spectra	S – S
**$Pt^+(C_2H_2)_4$**	
Table S9: relative energies	S
Figure S87: diagram of isomers	S
Tables S10 – S80: cartesian coordinates & unscaled vibrational frequencies	S – S
Figures S88 – S90: simulated spectra	S – S
**$Pt^+(C_2H_2)_5$**	
Table S81: relative energies	S
Figure S91: diagram of isomers	S
Tables S82 – S98: cartesian coordinates & unscaled vibrational frequencies	S – S
Figures S92-93: simulated spectra	S – S
**$Pt^+(C_2H_2)_6$**	
Table S99: relative energies	S
Tables S100 – S109: cartesian coordinates & unscaled vibrational frequencies	S –
Figures S94 – S95: simulated spectra	S –

Full citation for reference 59:

Frisch, M. J.; Trucks, G. W.; Schlegel, H. B.; Scuseria, G. E.; Robb, M. A.; Cheeseman, J. R.; Scalmani, G.; Barone, V.; Petersson, G. A.; Nakatsuji, H.; Li, X.; Caricato, M.; Marenich, A. V.; Bloino, J.; Janesko, B. G.; Gomperts, R.; Mennucci, B.; Hratchian, H. P.; Ortiz, J. V.; Izmaylov, A. F.; Sonnenberg, J. L.; Williams-Young, D.; Ding, F.; Lipparini, F.; Egidi, F.; Goings, J.; Peng, B.; Petrone, A.; Henderson, T.; Ranasinghe, D.; Zakrzewski, V. G.; Gao, J.; Rega, N.; Zheng, G.; Liang, W.; Hada, M.; Ehara, M.; Toyota, K.; Fukuda, R.; Hasegawa, J.; Ishida, M.; Nakajima, T.; Honda, Y.; Kitao, O.; Nakai, H.; Vreven, T.; Throssell, K.; Montgomery, J. A., Jr.; Peralta, J. E.; Ogliaro, F.; Bearpark, M. J.; Heyd, J. J.; Brothers, E. N.; Kudin, K. N.; Staroverov, V. N.; Keith, T. A.; Kobayashi, R.; Normand, J.; Raghavachari, K.; Rendell, A. P.; Burant, J. C.; Iyengar, S. S.; Tomasi, J.; Cossi, M.; Millam, J. M.; Klene, M.; Adamo, C.; Cammi, R.; Ochterski, J. W.; Martin, R. L.; Morokuma, K.; Farkas, O.; Foresman, J. B.; Fox, D. J. Gaussian 16 (Revision C.01), Gaussian, Inc., Wallingford CT, 2016.

All calculations were performed using density functional theory (DFT) with the B3LYP functional. The fully relativistic ECP60MDF Stuttgart/Cologne pseudopotential and corresponding correlation consistent triple-ζ basis set (cc-pVTZ-pp) were used for the platinum atoms. The all electron cc-pvtz basis was used for carbon and hydrogen. The thresholds for energy and structure optimizations were set to “tight,” and all calculations used an “ultrafine” integration grid. The structures presented were checked for electronic wavefunction stability with the “stable=opt” keyword. All structures except for the calculated transition states were free of imaginary vibrational frequencies. All electronic energies are zero-point vibrational energy (ZPVE) corrected.

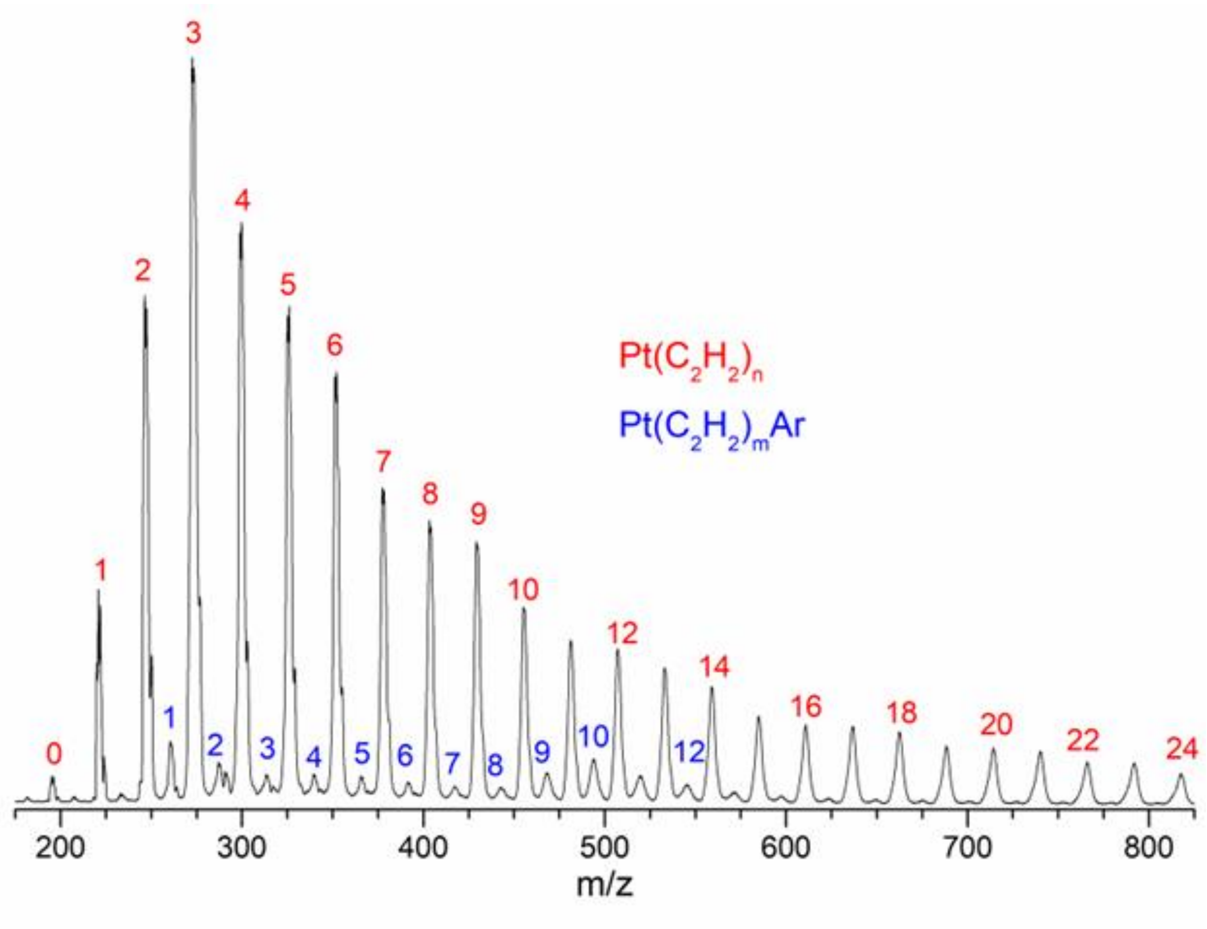

Figure S1. Mass spectrum of $Pt^+(C_2H_2)_n$ ions produced via laser vaporization of a platinum rod in a supersonic expansion of argon with acetylene.

Table S1. C_2H_2 calculated at the B3LYP/Def2TZVP level of theory using Gaussian16.

2s + 1	E (hartree)	Relative E (kcal/mol)
1	-77.336561	+0.0

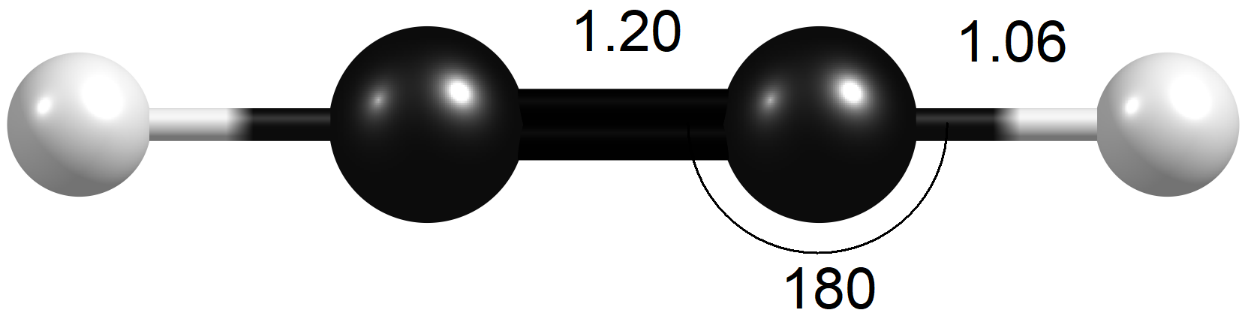

Figure S2. The optimized geometry of C_2H_2 followed by its predicted frequencies (cm^{-1}) and IR intensities (km/mol).

Frequency (cm^{-1})	Intensity (km/mol)
653.2805	0
653.2805	0
767.9801	96.2065
767.9801	96.2065
2071.728	0
3415.0376	89.1282
3518.5136	0

Table S2. Pt^+ electronic energy calculated at the B3LYP/cc-pVTZ(-pp) level with fully relativistic ECP on the platinum atom.

2s + 1	E (hartree)	Relative E (kcal/mol)
2	-119.082866	+0.0
4	-119.055477	+17.2

Table S3. $Pt^+(C_2H_2)$ electronic energy calculated at the B3LYP/cc-pVTZ(-pp) level with Stuttgardt/Koeln pseudopotential.

Isomer	2s + 1	E (hartree)	Relative E (kcal/mol)	C_2H_2 BE (kcal/mol)
1a	2	-196.540703	+0.0	76.1
1b	4	-196.450071	+56.8	36.4
1c	4	-196.447341	+58.6	34.7
1d	4	-196.444980	+60.1	33.2

Table S4. $Pt^+(C_2H_2)Ar_2$ electronic energy calculated at the B3LYP/cc-pVTZ(-pp) level with Stuttgardt/Koeln pseudopotential.

Isomer	2s + 1	E (hartree)	Relative E (kcal/mol)	Ar BE (kcal/mol)
1aI	2	-1251.68009	+0.0	12.9
1aII	2	-1251.677564	+1.6	11.4
1aIII	2	-1251.661677	+11.6	1.4
1bI	2	-1251.581145	+62.1	7.7
1bII	2	-1251.580573	+62.4	7.3
1cI	2	-1251.580286	+62.6	8.9
1cII	2	-1251.578287	+63.9	7.7
1cIII	2	-1251.57027	+68.9	2.6

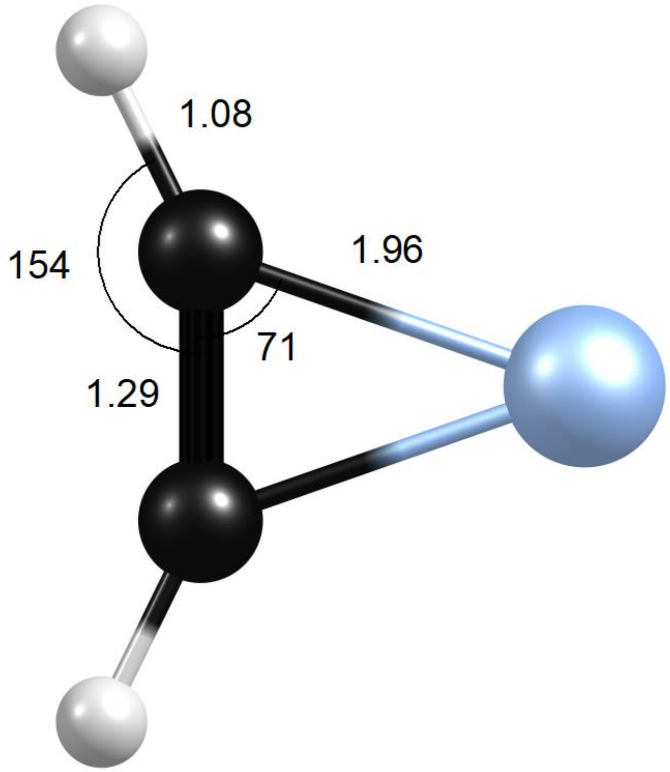

Figure S3. The optimized geometry of 1a-doublet $Pt^+(C_2H_2)$ followed by its predicted frequencies (cm^{-1}) and IR intensities (km/mol).

Frequency (cm^{-1})	Intensity (km/mol)
533.72	7.3317
563.2752	0.4771
733.8709	82.1506
752.1907	0
861.2148	10.3934
905.5309	84.0209
1628.7822	17.9081
3238.2267	162.0831
3292.7126	43.7313

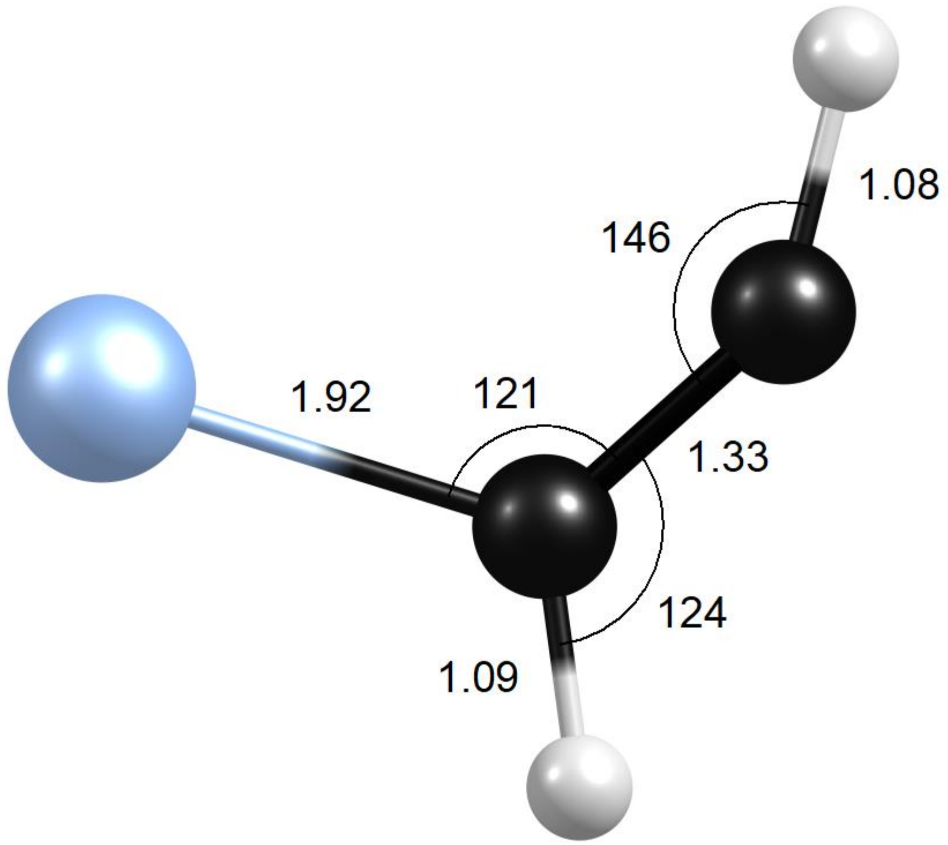

Figure S4. The optimized geometry of isomer 1b-quartet $Pt^{+}(C_2H_2)$ followed by its predicted frequencies (cm^{-1}) and IR intensities (km/mol).

Frequency (cm^{-1})	Intensity (km/mol)
242.1507	7.2285
574.9551	16.6206
639.168	131.3208
677.3277	3.5848
785.686	39.5536
1088.3522	14.8023
1373.0161	11.1744
3103.2381	43.4248
3252.2514	68.0296

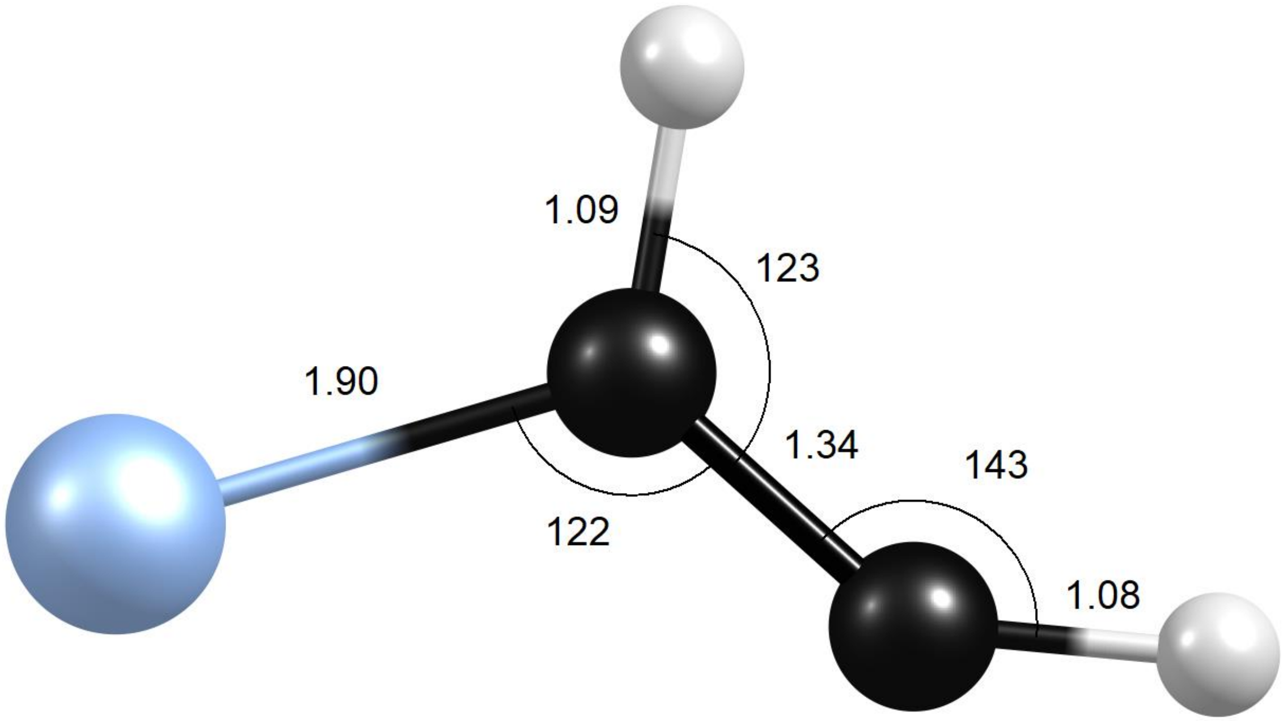

Figure S5. The optimized geometry of isomer 1c-quartet $Pt^{+}(C_2H_2)$ followed by its predicted frequencies (cm^{-1}) and IR intensities (km/mol).

Frequency (cm^{-1})	Intensity (km/mol)
252.9525	7.2486
503.8322	42.2483
537.4228	62.2465
720.6953	19.3892
847.6311	2.2854
1138.3023	15.5152
1322.6356	9.5757
3039.77	24.1562
3220.906	81.3903

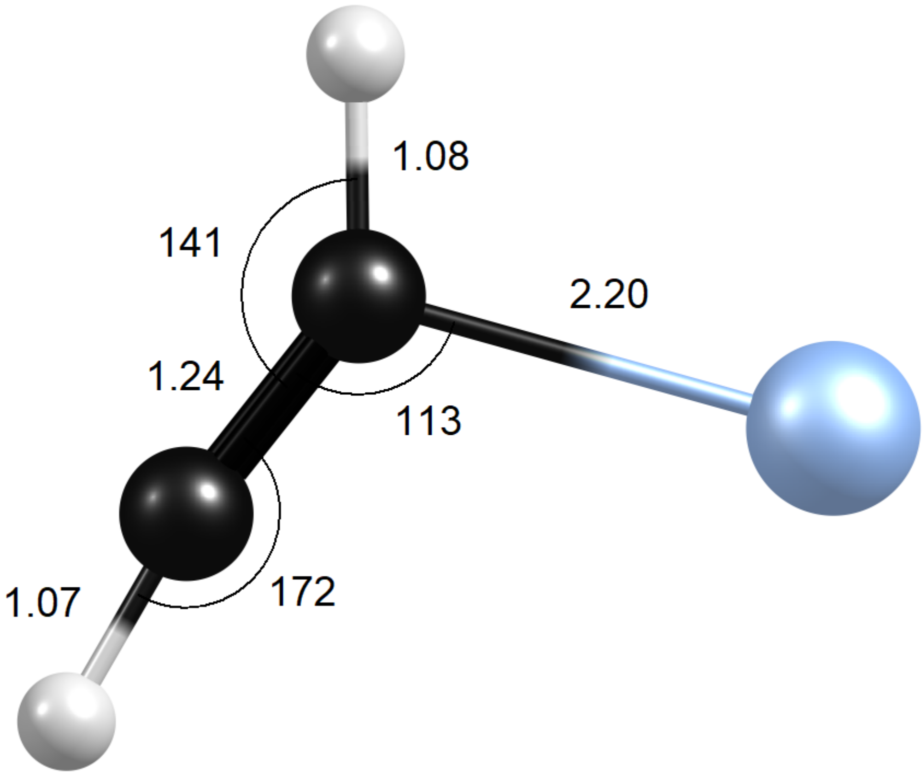

Figure S6. The optimized geometry of isomer 1d-quartet $Pt^+(C_2H_2)$ followed by its predicted frequencies (cm^{-1}) and IR intensities (km/mol).

Frequency (cm^{-1})	Intensity (km/mol)
167.9568	0.3369
217.0895	0.2248
641.0403	4.3844
684.2162	69.4841
750.0448	76.0854
895.0925	17.514
1769.1425	11.4039
3236.0208	138.3188
3374.3849	127.0839

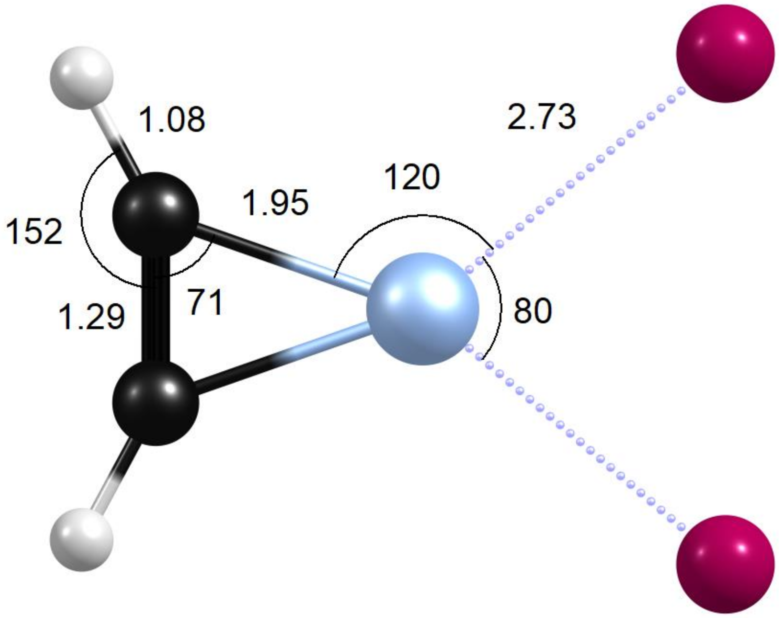

Figure S7. The optimized geometry of isomer 1aI-doublet $Pt^{+}(C_2H_2)Ar_2$ followed by its predicted frequencies (cm^{-1}) and IR intensities (km/mol).

Frequency (cm^{-1})	Intensity (km/mol)
63.5653	0
65.9392	0.0898
66.0107	0.0672
74.1142	4.2699
109.1256	7.9242
128.8092	6.9982
556.6254	6.338
593.5711	0.018
736.3167	63.9266
773.0115	0
868.0641	2.8518
927.9489	81.6367
1616.4926	7.7856
3247.4345	112.802
3297.963	44.0182

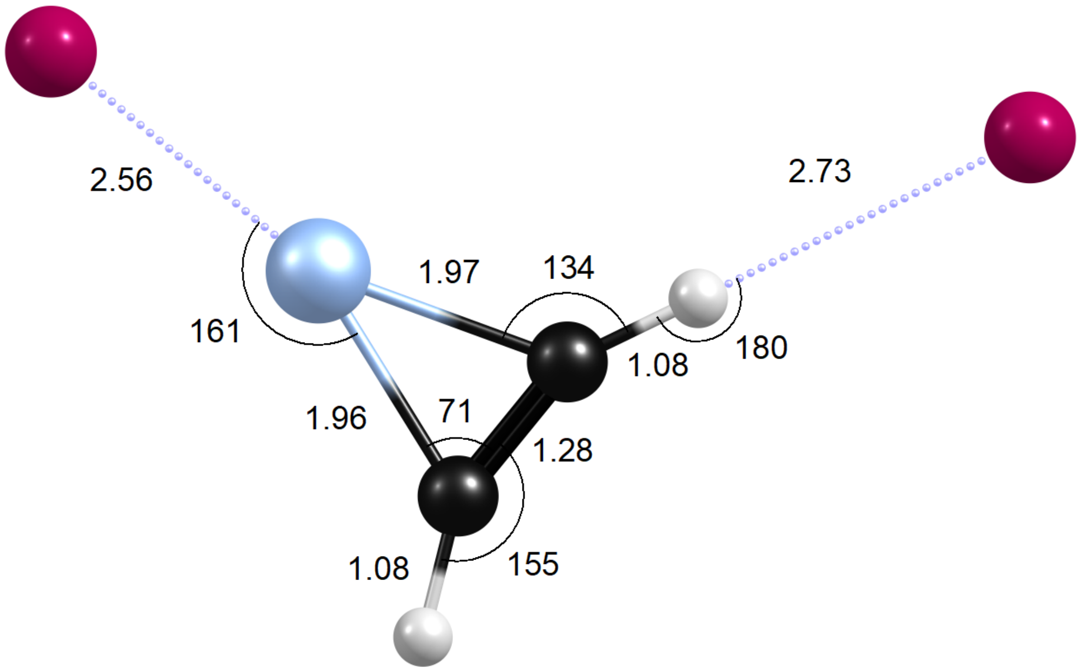

Figure S8. The optimized geometry of isomer 1aII-doublet $Pt^{+}(C_2H_2)Ar_2$ followed by its predicted frequencies (cm^{-1}) and IR intensities (km/mol).

Frequency (cm^{-1})	Intensity (km/mol)
9.6811	0.1942
31.1091	0.1393
34.8631	0.2639
50.519	2.4612
50.8425	5.7539
161.273	6.2411
531.5027	6.9607
566.0395	1.5706
735.9697	66.0968
760.3913	3.6342
863.1154	4.9313
903.3345	64.5638
1662.0227	17.8425
3236.4413	244.6236
3298.8423	61.8452

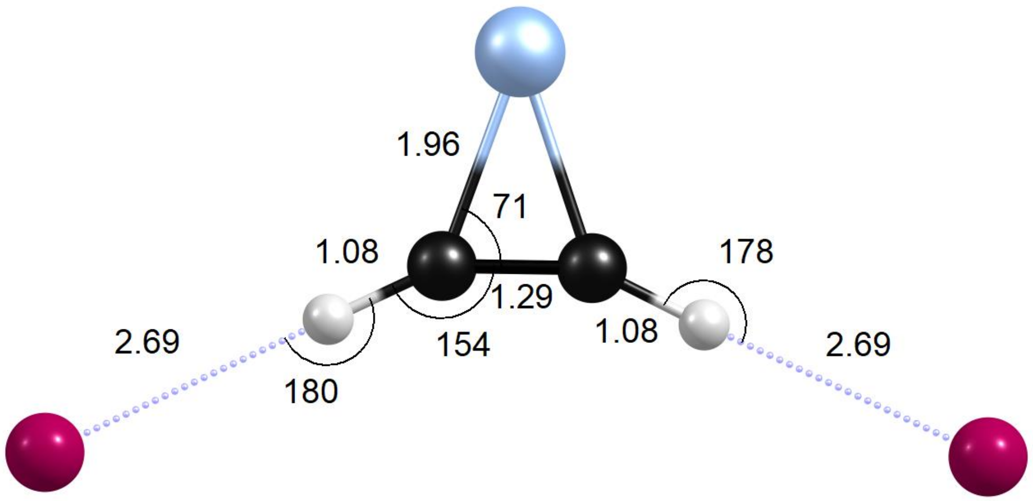

Figure S9. The optimized geometry of isomer 1aIII-doublet $Pt^+(C_2H_2)Ar_2$ followed by its predicted frequencies (cm^{-1}) and IR intensities (km/mol).

Frequency (cm^{-1})	Intensity (km/mol)
6.9155	0.5039
18.8605	0.0226
19.8994	2.52
35.7521	0.7629
54.0843	0
57.1731	7.8454
532.8036	7.5177
564.9094	3.933
748.2992	60.413
762.5085	0
874.1966	3.7686
912.5715	85.1146
1627.071	28.7739
3209.8008	372.6325
3267.4569	97.9415

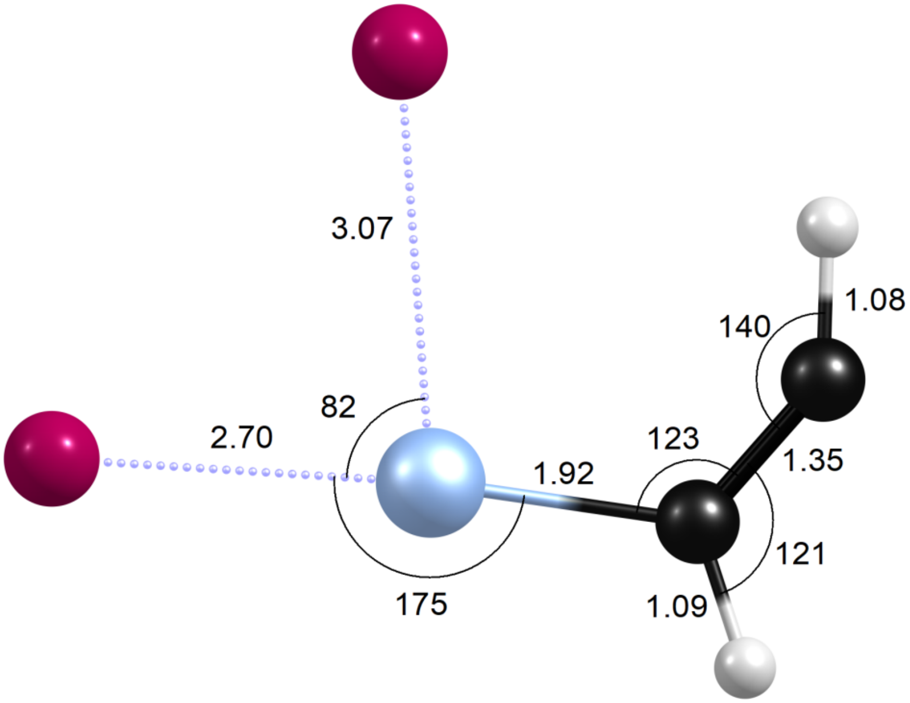

Figure S10. The optimized geometry of isomer 1bI-quartet $Pt^{+}(C_2H_2)Ar_2$ followed by its predicted frequencies (cm^{-1}) and IR intensities (km/mol).

Frequency (cm^{-1})	Intensity (km/mol)
27.4325	2.9003
44.1006	0.2238
55.9564	3.2789
75.6543	3.2951
79.583	0.0078
122.3495	10.7222
265.3002	7.8543
615.4929	6.6498
674.8922	1.0984
791.0695	59.3423
822.4981	33.6144
1165.8127	4.5282
1360.8723	47.2128
3087.3617	17.8323
3231.6125	25.176

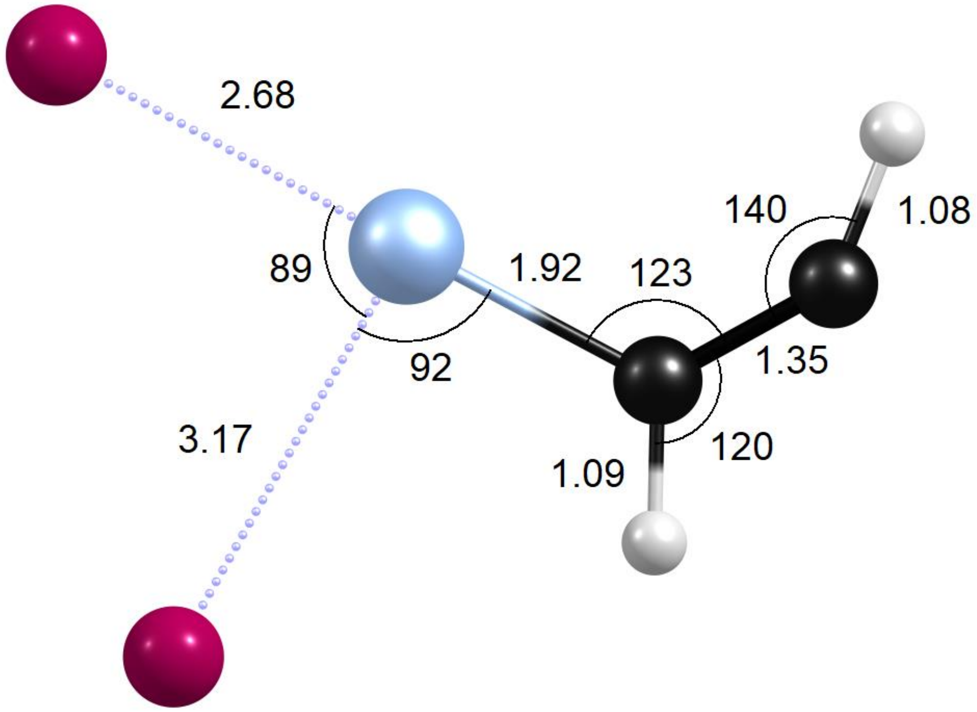

Figure S11. The optimized geometry of isomer 1bII-quartet $Pt^{+}(C_2H_2)Ar_2$ followed by its predicted frequencies (cm^{-1}) and IR intensities (km/mol).

Frequency (cm^{-1})	Intensity (km/mol)
31.2634	0.0278
33.7731	3.2783
48.3133	5.0085
63.7674	0.0058
71.7776	1.6656
125.8809	11.3665
265.8523	6.2576
620.2875	5.9475
678.288	0.4443
801.4275	54.3003
820.8248	29.4064
1168.9638	3.7272
1355.4086	54.4014
3092.8374	11.4657
3227.1757	31.5795

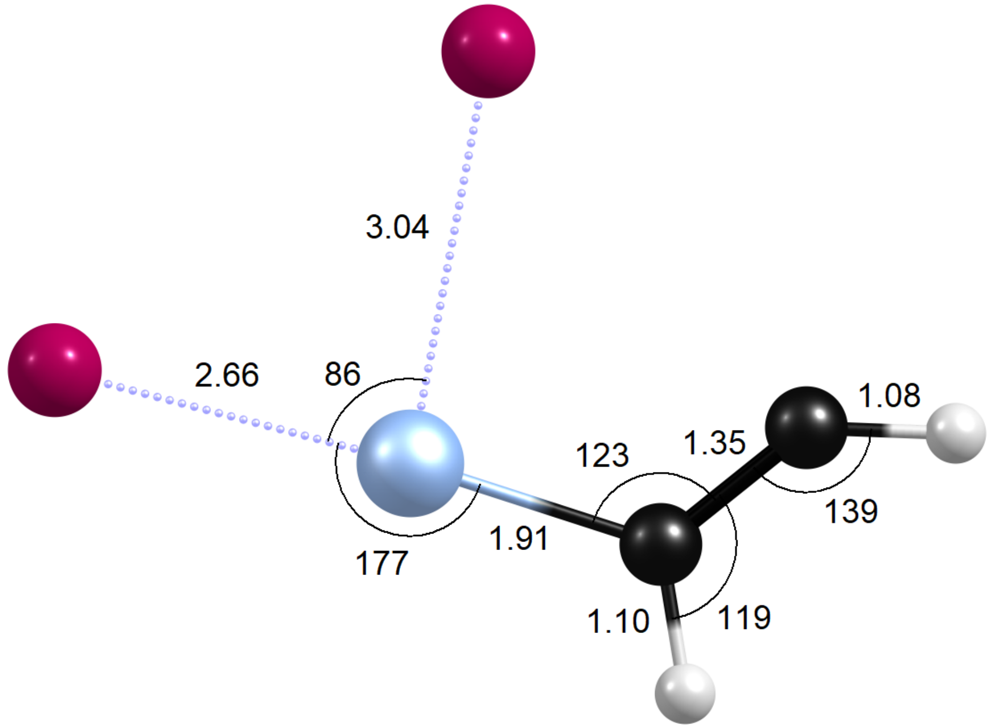

Figure S12. The optimized geometry of isomer 1cI-quartet $Pt^{+}(C_2H_2)Ar_2$ followed by its predicted frequencies (cm^{-1}) and IR intensities (km/mol).

Frequency (cm^{-1})	Intensity (km/mol)
34.2524	1.9205
42.0651	0.1035
61.1075	4.1299
77.7243	4.249
92.7739	3.1854
133.9382	8.9253
279.9157	4.3476
531.6307	52.62
584.6086	9.3279
806.932	22.1437
892.4083	2.1806
1177.9259	12.5948
1348.5021	62.6256
3021.5472	8.8481
3201.8436	31.1816

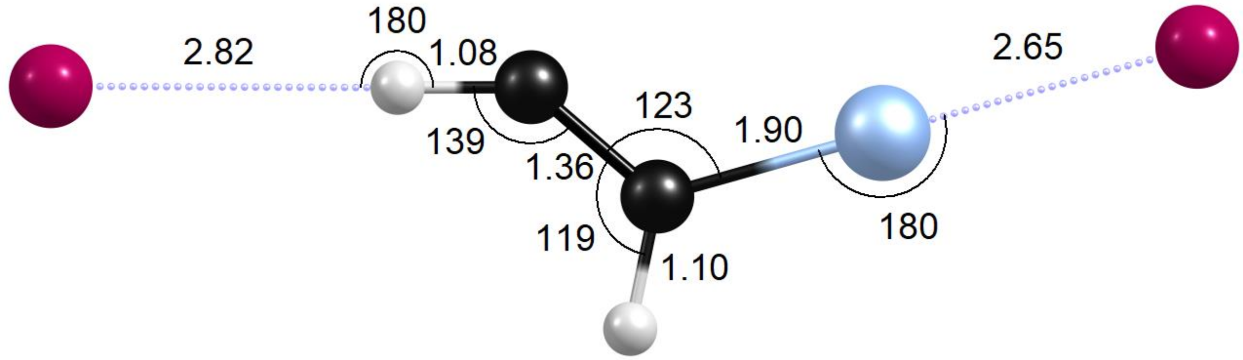

Figure S13. The optimized geometry of isomer 1cII-quartet $Pt^+(C_2H_2)Ar_2$ followed by its predicted frequencies (cm^{-1}) and IR intensities (km/mol).

Frequency (cm^{-1})	Intensity (km/mol)
10.1165	1.1745
14.2634	0.7766
29.6081	1.3498
64.7201	5.2872
71.1741	3.8307
139.2163	10.4024
283.0815	2.8046
533.9075	44.1742
593.6369	11.2846
807.9334	20.1538
896.385	2.7219
1177.329	11.0724
1340.459	68.0238
3018.5984	9.4302
3186.6548	99.4017

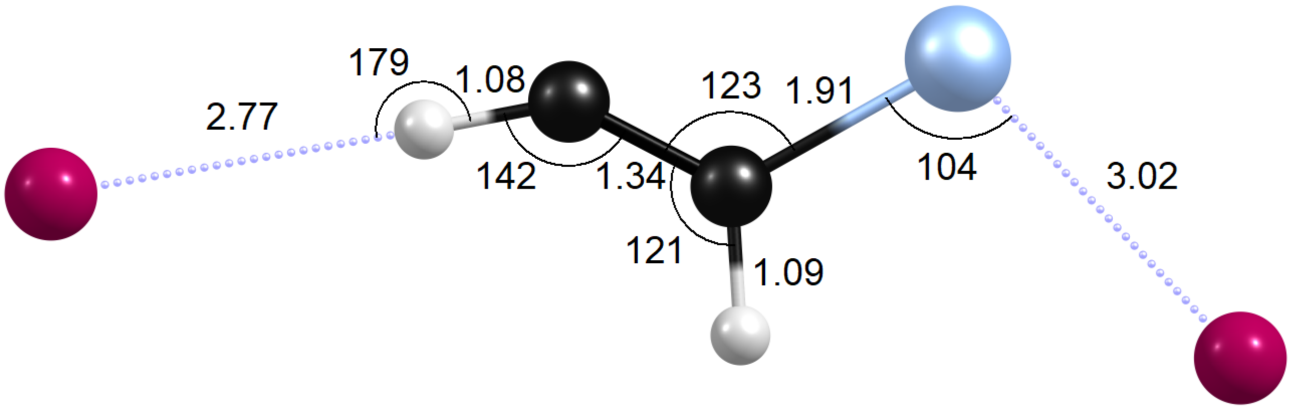

Figure S14. The optimized geometry of isomer 1cIII-quartet $Pt^{+}(C_2H_2)Ar_2$ followed by its predicted frequencies (cm^{-1}) and IR intensities (km/mol).

Frequency (cm^{-1})	Intensity (km/mol)
9.117	0.6945
15.444	4.4325
24.9308	2.7479
32.1039	1.6815
37.2086	0.0406
64.2168	8.0172
273.5396	5.688
531.5012	31.9821
552.4229	50.9168
744.1446	23.2211
859.7553	3.2142
1151.0608	12.6978
1322.3913	28.0339
3039.2777	16.2621
3197.0343	162.5426

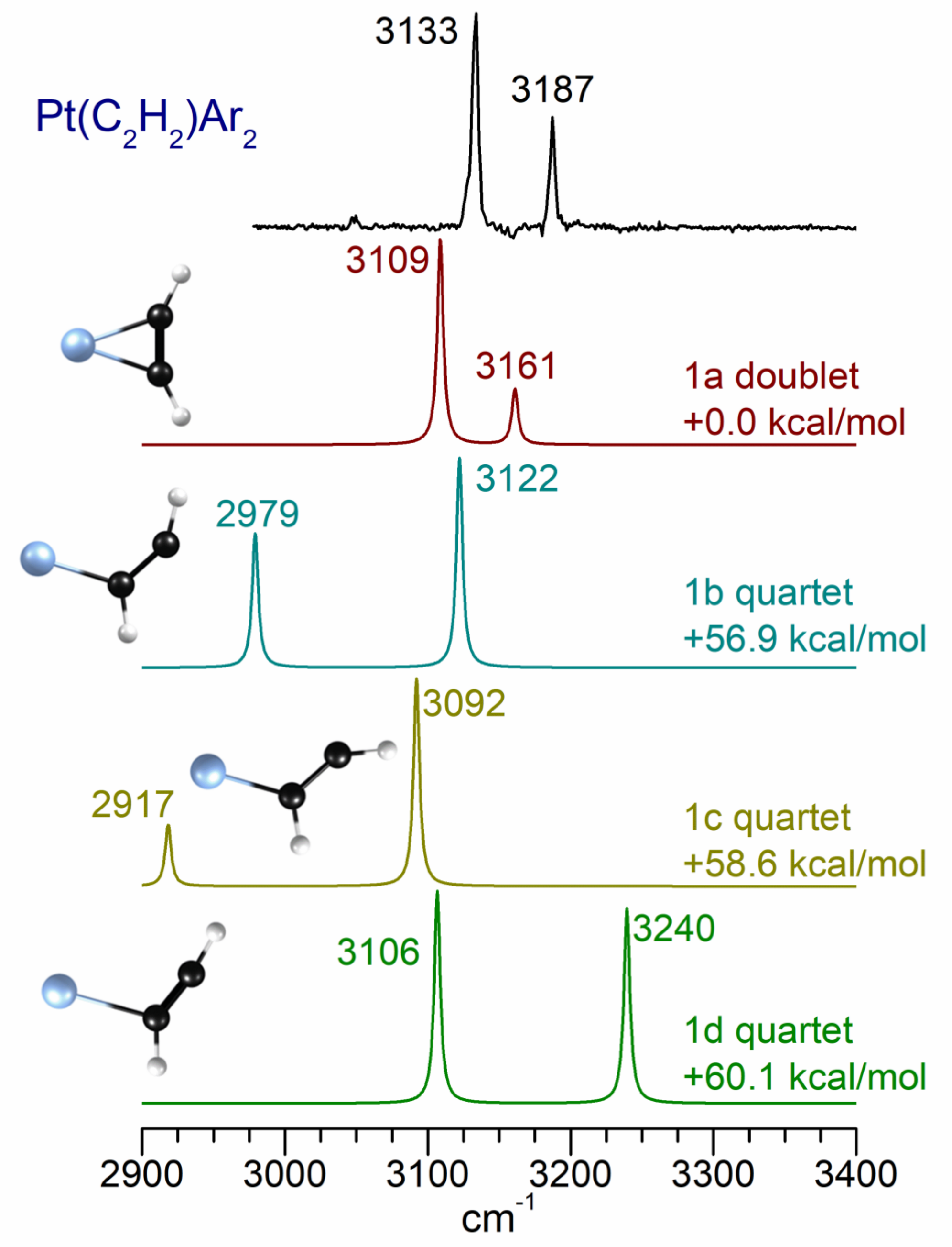
3133
3187
Pt(C2H2)Ar2
3109
3161
1a doublet
+0.0 kcal/mol
2979
3122
1b quartet
+56.9 kcal/mol
3092
2917
1c quartet
+58.6 kcal/mol
3106
3240
1d quartet
+60.1 kcal/mol
2900
3000
3100
3200
3300
3400
cm-1

Figure S15.

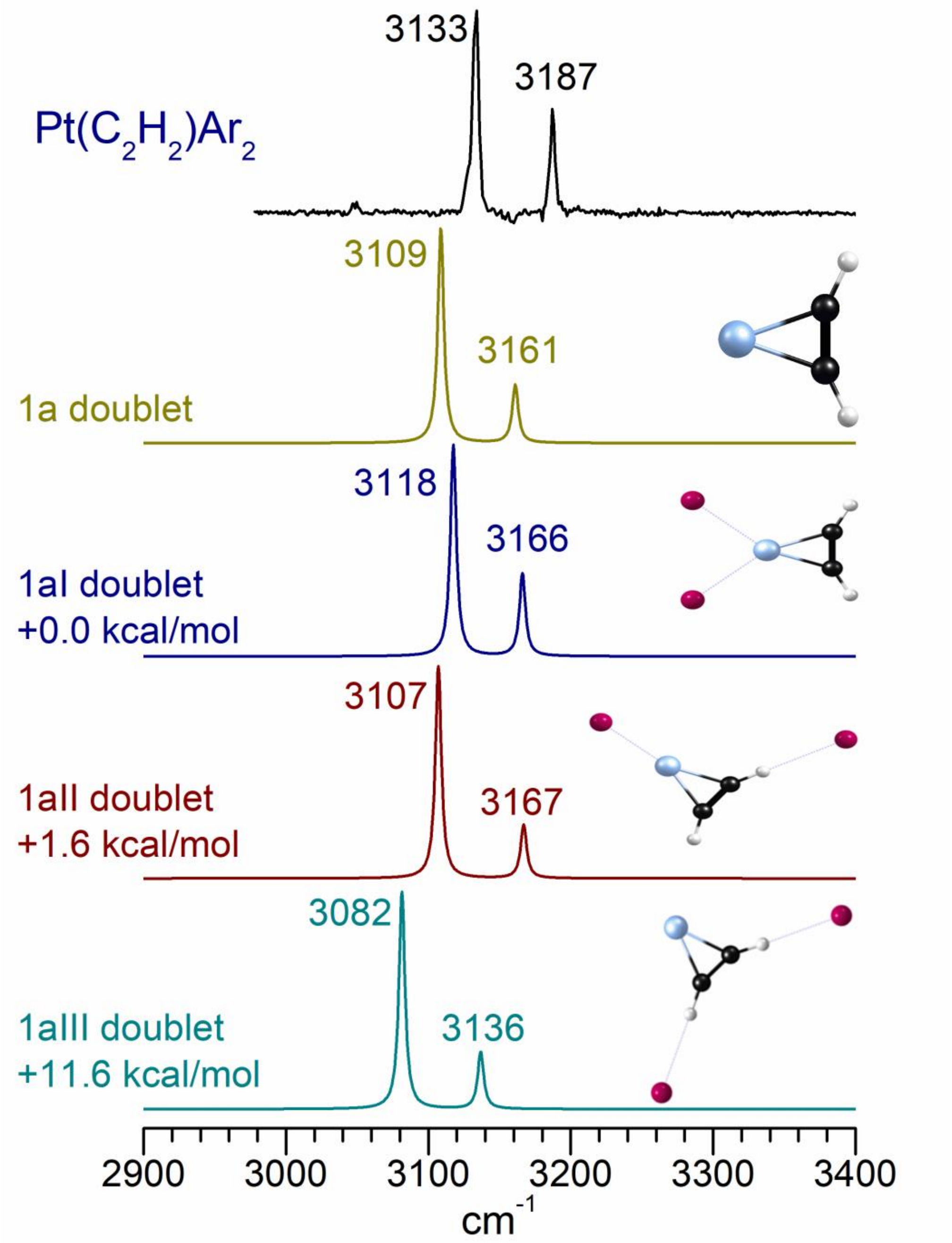
3133
3187
$Pt(C_2H_2)Ar_2$
3109
3161
1a doublet
3118
3166
1aI doublet
+0.0 kcal/mol
3107
3167
1aII doublet
+1.6 kcal/mol
3082
3136
1aIII doublet
+11.6 kcal/mol
2900
3000
3100
3200
3300
3400
cm-1
Figure S16.

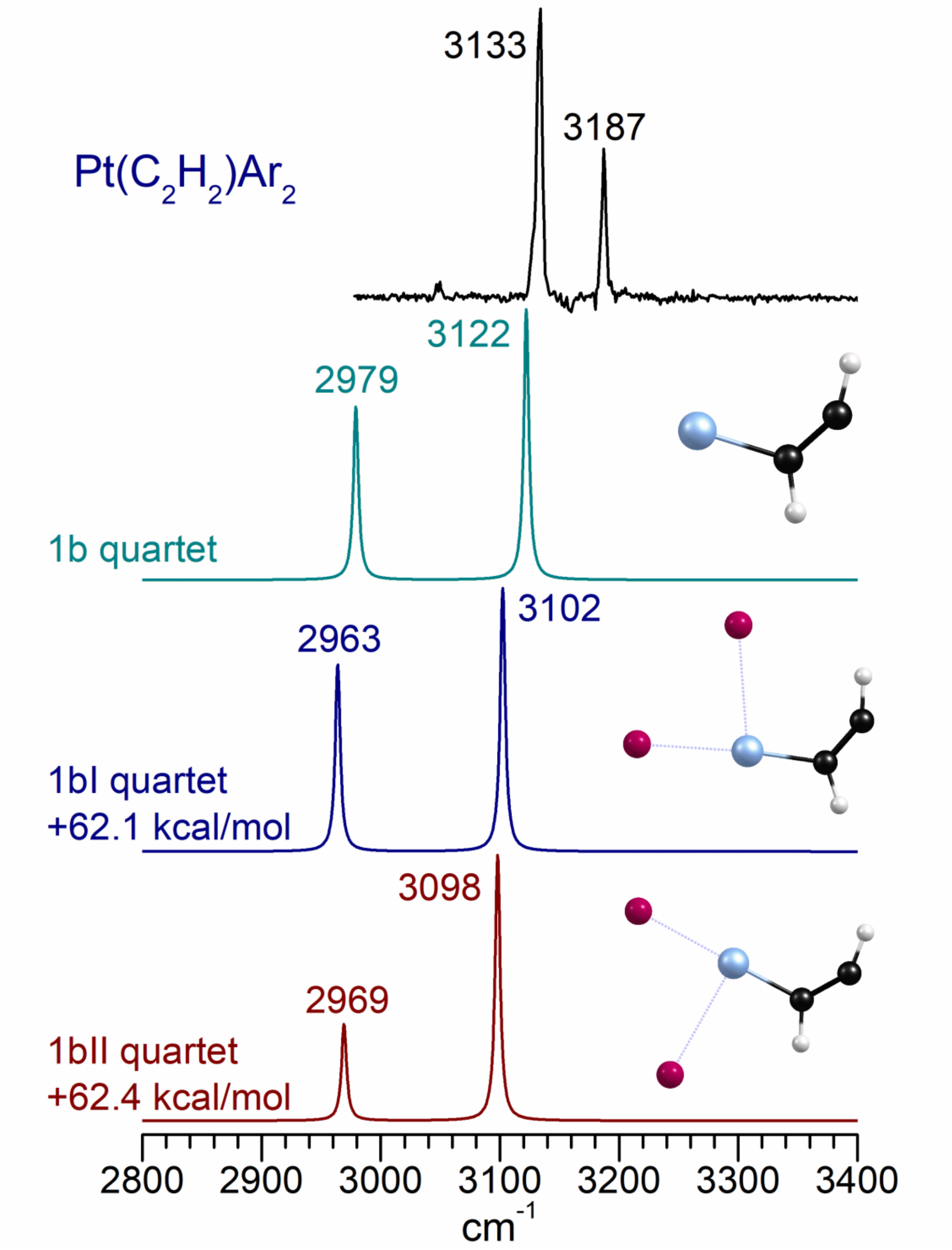
3133
3187
$Pt(C_2H_2)Ar_2$
3122
2979
1b quartet
3102
2963
1bI quartet
+62.1 kcal/mol
3098
2969
1bII quartet
+62.4 kcal/mol
2800
2900
3000
3100
3200
3300
3400
cm^{-1}

Figure S17.

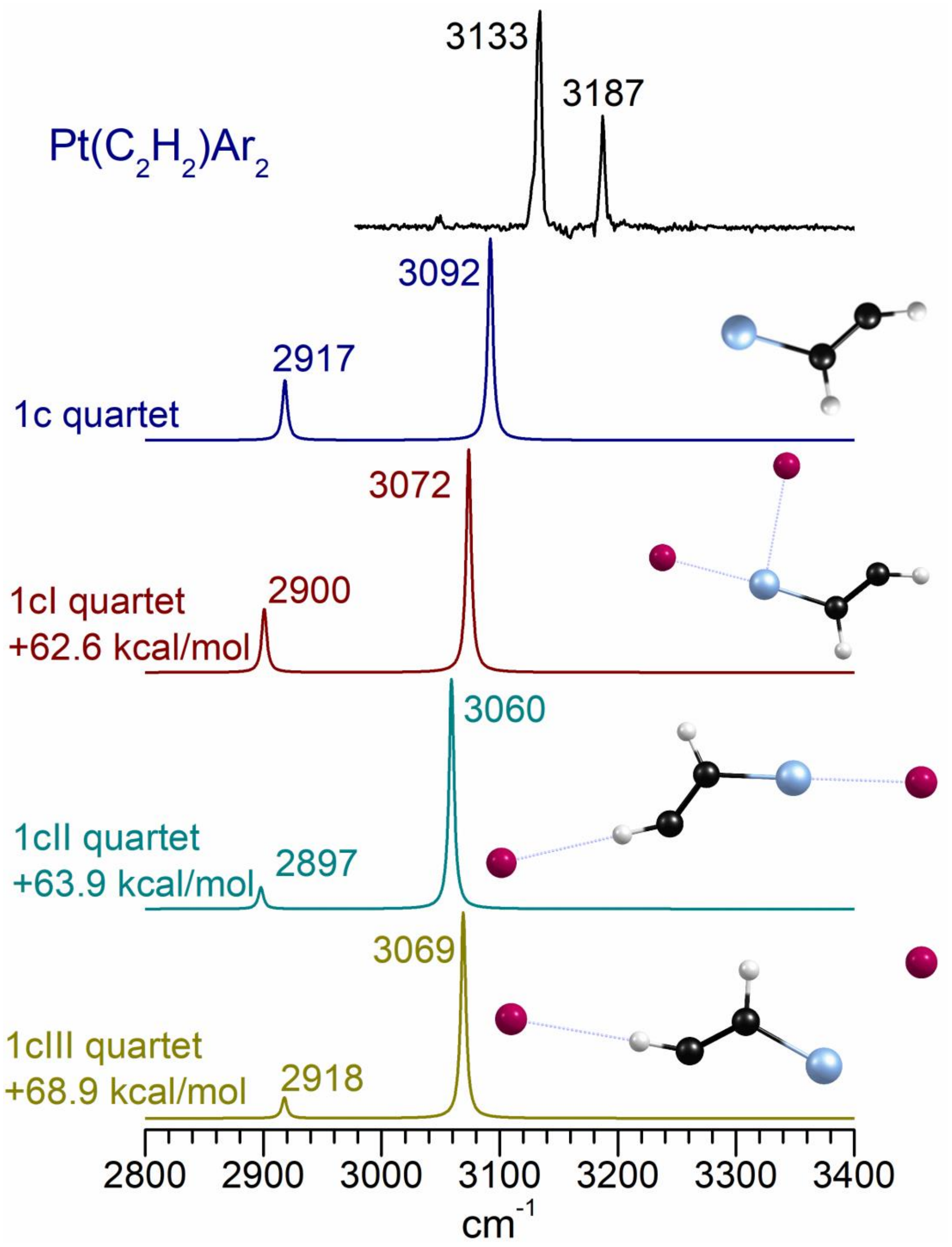
3133
3187
Pt(C2H2)Ar2
3092
2917
1c quartet
3072
2900
1cI quartet
+62.6 kcal/mol
3060
1cII quartet
+63.9 kcal/mol
2897
3069
1cIII quartet
+68.9 kcal/mol
2918
2800 2900 3000 3100 3200 3300 3400
cm-1

Figure S18.

Table S5. $Pt^+(C_2H_2)_2$ electronic energy calculated at the B3LYP/cc-pVTZ(-pp) level with Stuttgardt/Koeln pseudopotential.

Isomer	2S+1	Energy (hartree)	Relative E (kcal/mol)
2a	2	-273.983334	+0.0
2a	4	-273.890069	+58.52468359
2b	2	-273.949982	+20.92870045
2b	4	-273.852844	+81.88372875
2c	2	-273.935422	+30.06524034
2c	4	-273.869157	+71.64716452
TS1	2	-273.872591	+69.49229652
TS2	2	-273.90706	+47.86266784

Table S6. $Pt^+(C_2H_2)Ar_2$ electronic energy calculated at the B3LYP/cc-pVTZ(-pp) level with Stuttgardt/Koeln pseudopotential.

Isomer	2S+1	Energy (hartree)	Relative E (kcal/mol)
2aI	2	-1329.111814	0
2aI	4	-1329.018818	58.35594626
2aII	2	-1329.111667	0.092306663
2aII	4	-1329.014836	60.85468952
2aIII	2	-1329.111575	0.150037547
2bI	2	-1329.071014	25.60245476
2bI	4	-1328.975738	85.38906017
2bII	2	-1329.070945	25.64575292
2bIII	2	-1329.070593	25.8666363
2cI	2	-1329.068509	27.17436633
2cI	4	-1328.996393	72.42784922
2cII	2	-1329.065896	28.81404893
2cII	4	-1328.996159	72.57468647

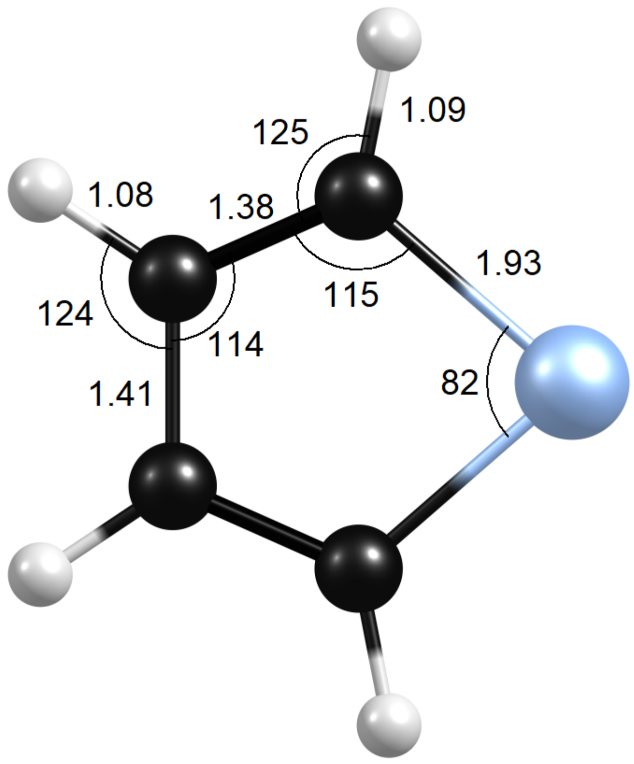

Figure S19. The optimized geometry of isomer 2a-doublet $Pt^+(C_2H_2)_2$followed by its predicted frequencies (cm^{-1}) and IR intensities (km/mol).

Frequency (cm^{-1})	Intensity (km/mol)	Frequency (cm^{-1})	Intensity (km/mol)
224.7258	0.0178	1101.9555	10.3621
387.8154	2.387	1126.2887	0.2987
400.0389	0	1254.5269	35.2448
535.2535	0.2837	1370.4655	1.8358
673.1773	110.8358	1467.2551	45.3909
717.2516	26.4547	1500.3811	11.3829
801.0845	2.8032	3145.5998	21.4869
802.3176	0	3152.57	1.8892
931.399	0.7431	3186.5871	9.4732
987.0843	0	3197.8477	14.043
1058.8453	0.0393		

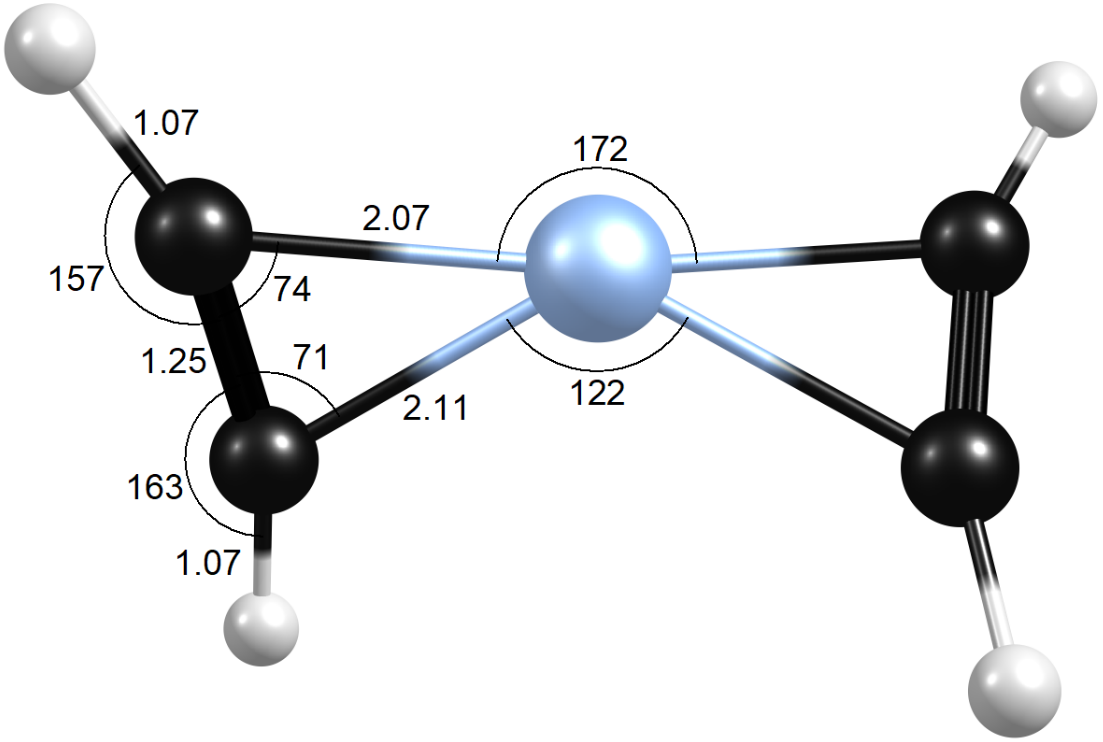

Figure S20. The optimized geometry of isomer 2b-doublet $Pt^{+}(C_2H_2)_2$ followed by its predicted frequencies (cm^{-1}) and IR intensities (km/mol).

Frequency (cm^{-1})	Intensity (km/mol)	Frequency (cm^{-1})	Intensity (km/mol)
49.019	1.8205	765.3282	80.4175
71.1631	8.6817	809.0386	29.4037
125.5204	5.1345	825.8351	17.6024
228.3191	6.4914	834.0834	44.8737
417.6063	2.6988	1777.3587	13.242
473.5887	5.5707	1796.5269	0.0115
488.8768	13.9987	3278.1771	79.5845
699.9532	9.8366	3279.835	262.125
711.1581	1.4691	3353.6607	103.5515
743.6462	32.3208	3356.6017	23.0295
758.1885	5.1143		

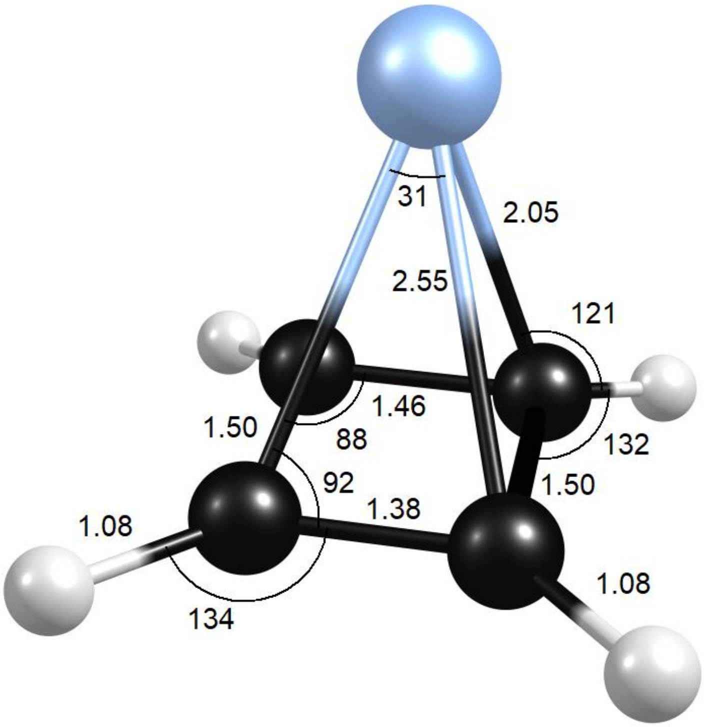

Figure S21. The optimized geometry of isomer 2c-doublet $Pt^{+}(C_2H_2)_2$ followed by its predicted frequencies (cm^{-1}) and IR intensities (km/mol).

Frequency (cm^{-1})	Intensity (km/mol)	Frequency (cm^{-1})	Intensity (km/mol)
160.6175	1.8293	1012.4796	5.6548
247.8755	1.6365	1161.0348	11.1379
496.7608	10.1913	1203.8132	1.5305
550.8528	3.5525	1277.5934	14.2439
784.2084	64.8094	1312.9931	36.3716
859.3665	20.6092	1459.5902	51.0978
883.5265	5.0482	3210.8525	2.6467
914.7048	11.7276	3225.0249	26.1323
943.0081	12.4489	3227.0182	21.2786
956.29	0.1973	3244.9216	6.8435
975.979	6.7325		

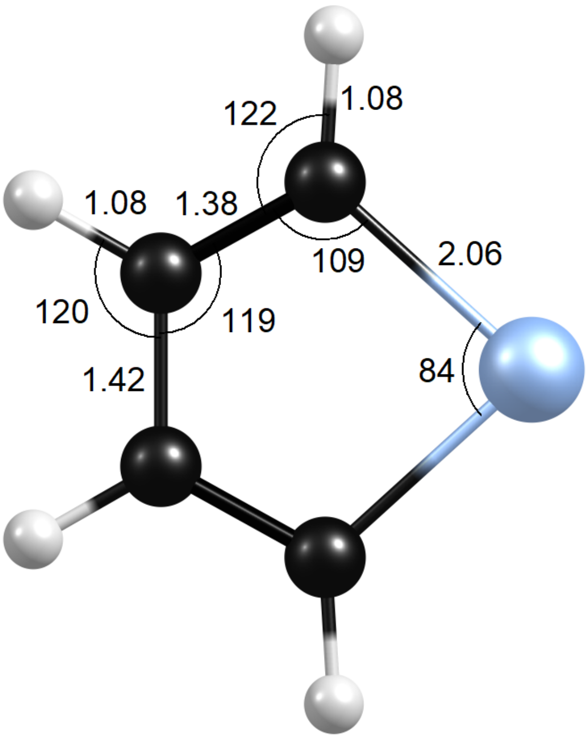

Figure S22. The optimized geometry of isomer 2a-quartet $Pt^+(C_2H_2)_2$ followed by its predicted frequencies (cm^{-1}) and IR intensities (km/mol).

Frequency (cm^{-1})	Intensity (km/mol)	Frequency (cm^{-1})	Intensity (km/mol)
175.6219	0.028	1089.7644	19.3213
253.009	0	1124.6012	1.0557
289.5359	0.3806	1226.768	3.3372
337.4184	9.6422	1322.0128	1.0669
621.8154	9.1424	1473.1853	72.3685
632.3651	0	1480.3279	3.1719
672.2528	71.7511	3152.2906	0.3682
749.9319	0.2551	3162.2293	8.7092
982.4063	0.0339	3172.7367	14.8708
996.8306	0.0793	3176.4052	1.1667
1011.5454	0		

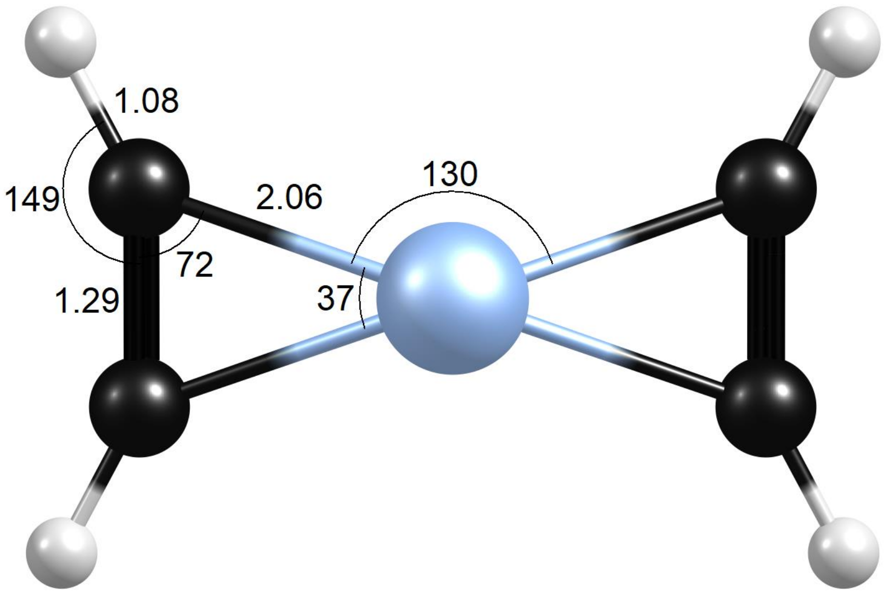

Figure S23. The optimized geometry of isomer 2b-quartet $Pt^{+}(C_2H_2)_2$ followed by its predicted frequencies (cm^{-1}) and IR intensities (km/mol).

Frequency (cm^{-1})	Intensity (km/mol)	Frequency (cm^{-1})	Intensity (km/mol)
108.2086	11.899	825.3673	17.5622
133.4134	17.8918	862.953	0
233.6808	0	870.4273	7.0684
377.9103	4.348	878.1998	112.1814
445.1941	0.0453	1532.6587	10.4236
511.1221	2.0982	1626.012	1.0466
526.5589	0	3198.7611	128.8635
636.6097	7.0902	3199.8788	0.0008
666.6812	78.3	3245.5842	89.1246
751.0824	17.5978	3246.7546	3.1368
791.4644	0		

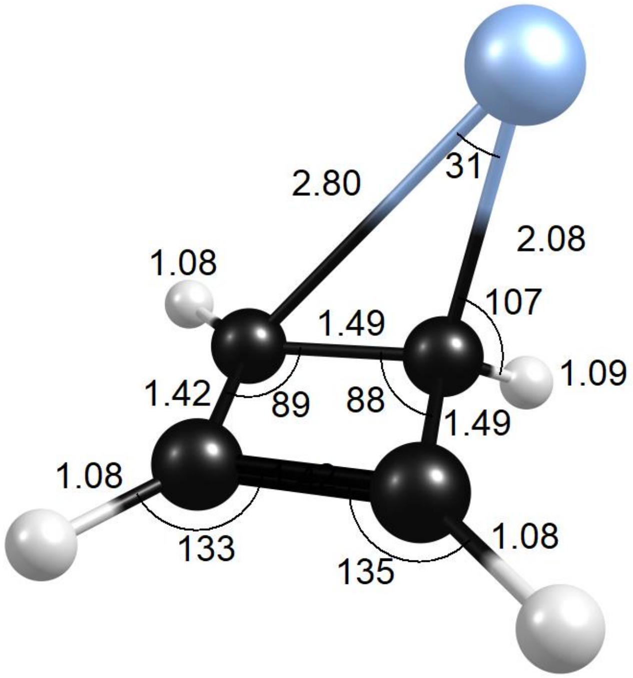

Figure S24. The optimized geometry of isomer 2c-quartet $Pt^+(C_2H_2)_2$ followed by its predicted frequencies (cm^{-1}) and IR intensities (km/mol).

Frequency (cm^{-1})	Intensity (km/mol)	Frequency (cm^{-1})	Intensity (km/mol)
85.2285	0.2211	1025.3569	59.9029
164.1517	0.0004	1158.4329	2.5733
328.2093	1.0818	1208.4128	1.7053
548.6946	25.5731	1208.7954	0.142
632.8425	0.526	1338.0877	13.8774
635.1799	113.5125	1361.6276	104.6095
868.7366	8.4126	3122.5074	6.4755
904.2356	0.8328	3209.9814	6.221
915.1092	25.3685	3251.8927	28.5041
949.8619	2.9247	3258.9858	0.0058
956.5249	8.78		

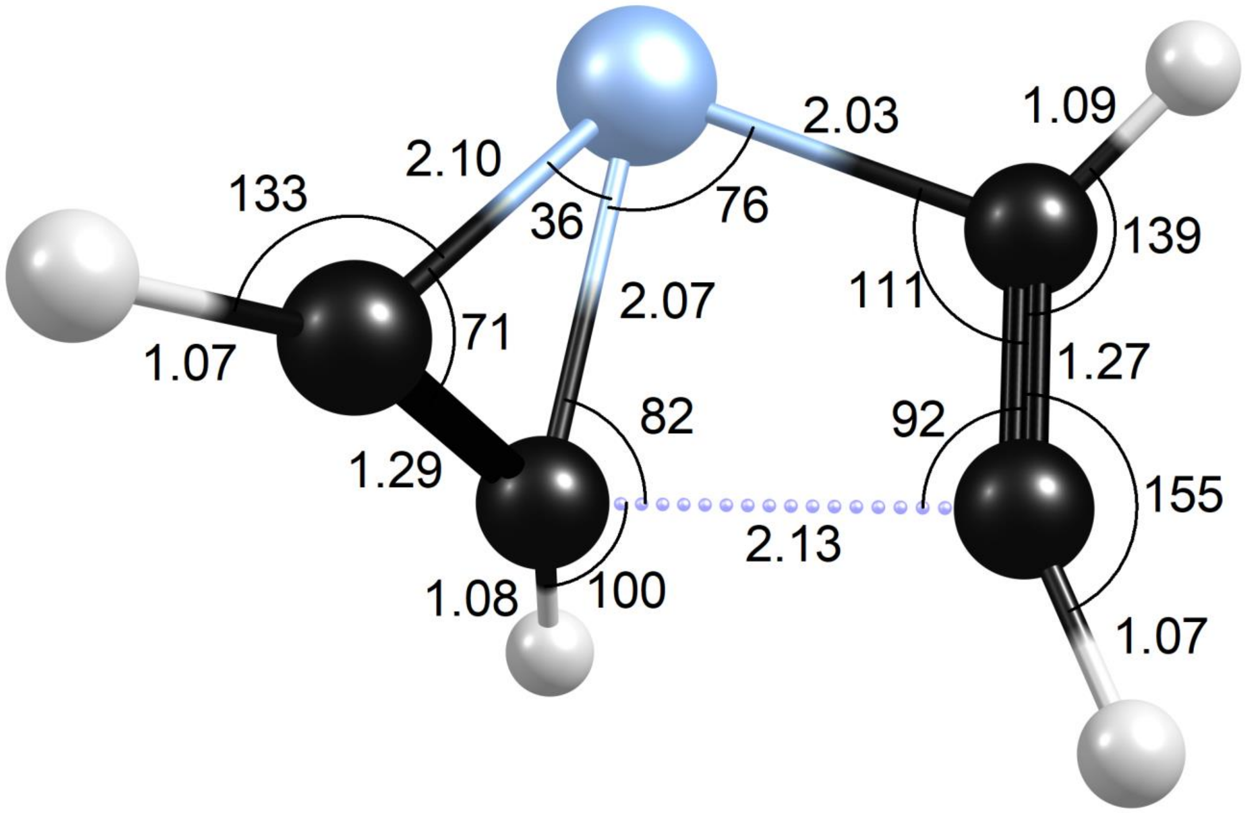

Figure S25. The optimized geometry of TS1-doublet $Pt^{+}(C_2H_2)_2$ followed by its predicted frequencies (cm^{-1}) and IR intensities (km/mol).

Frequency (cm^{-1})	Intensity (km/mol)	Frequency (cm^{-1})	Intensity (km/mol)
-678.1475	66.273	784.9897	21.4285
133.9597	3.2024	847.2185	28.7265
218.0881	3.6871	899.0335	15.9065
316.4545	3.6774	933.3641	12.3987
401.3348	34.0736	1534.3634	0.2772
408.1158	13.9055	1689.0717	26.2603
484.3808	17.1365	3147.099	51.8525
513.1504	22.9209	3236.8094	82.862
659.5346	12.3679	3304.9951	83.467
676.3901	43.5707	3323.3339	133.011
682.5123	56.2882		

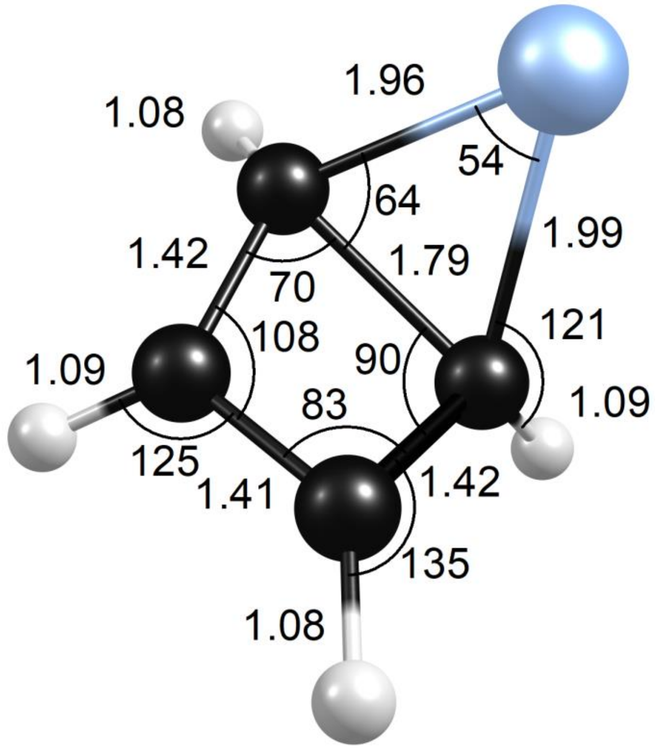

Figure S26. The optimized geometry of TS2-doublet $Pt^+(C_2H_2)_2$ followed by its predicted frequencies (cm^{-1}) and IR intensities (km/mol).

Frequency (cm^{-1})	Intensity (km/mol)	Frequency (cm^{-1})	Intensity (km/mol)
-297.4272	11.2595	1063.4342	9.5052
195.2198	2.1982	1066.9291	3.6619
257.9048	16.411	1181.3431	22.1383
406.4144	11.4515	1194.4279	13.0596
572.6238	19.6298	1324.6921	7.2487
644.4477	23.345	1370.5361	10.0492
750.0487	15.4749	3140.7215	16.3549
865.6925	45.537	3147.248	19.284
908.439	18.9597	3167.1241	21.7017
944.8489	11.5404	3276.8855	61.2535
1025.3584	7.6406		

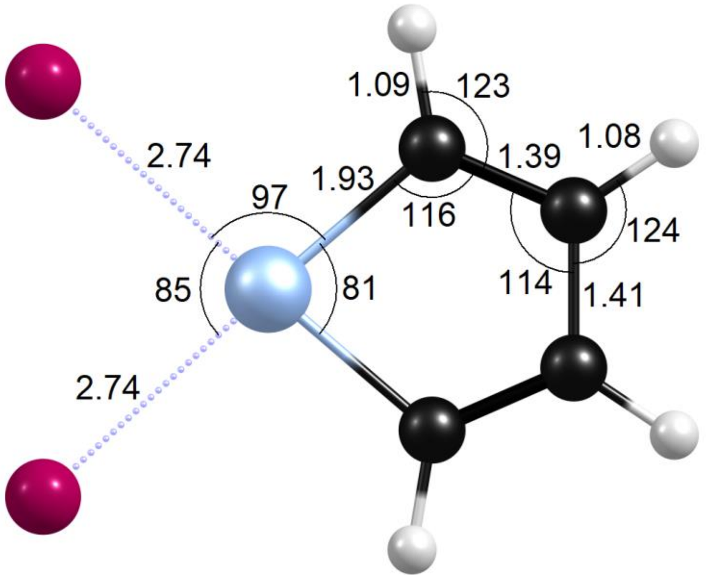

Figure S27. The optimized geometry of isomer 2aI-doublet $Pt^{+}(C_2H_2)_2Ar_2$ followed by its predicted frequencies (cm^{-1}) and IR intensities (km/mol).

Frequency (cm^{-1})	Intensity (km/mol)	Frequency (cm^{-1})	Intensity (km/mol)
57.8452	0.0083	947.5504	0.8955
59.5174	2.3684	992.0141	0
69.0336	0	1064.8531	0.0655
78.8887	1.3169	1108.9707	6.524
107.1652	3.7428	1130.8201	0.0586
116.0269	7.3319	1273.854	28.6493
249.7658	0.5683	1383.1487	5.0418
397.7096	3.5071	1469.5045	42.9328
402.004	0	1504.0048	11.1018
540.6772	0.0043	3147.9096	2.7655
682.9146	87.1292	3153.3571	0.1191
715.1922	17.1003	3187.6249	2.9111
812.4792	0	3199.3569	6.1784
818.3319	1.8279		

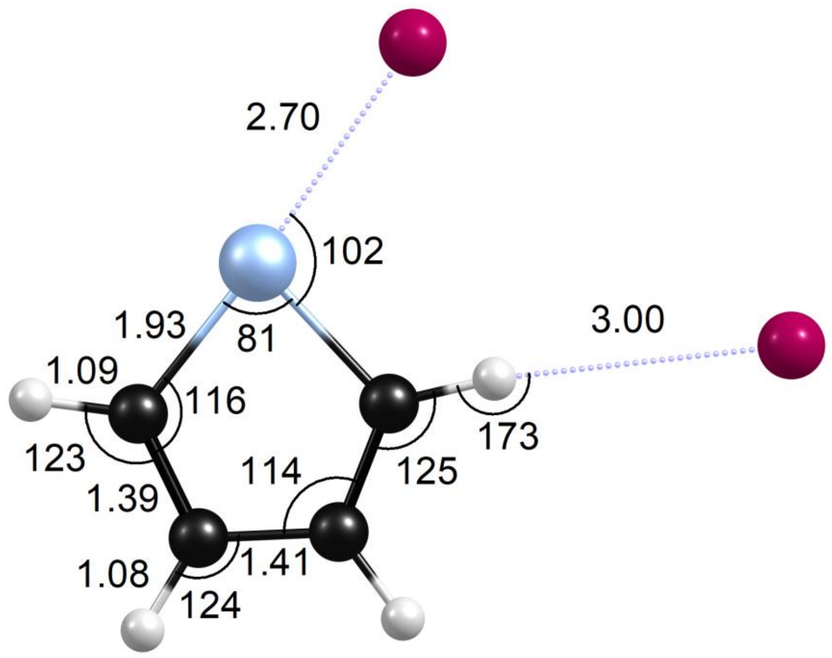

Figure S28. The optimized geometry of isomer 2aII-doublet $Pt^+(C_2H_2)_2Ar_2$ followed by its predicted frequencies (cm^{-1}) and IR intensities (km/mol).

Frequency (cm^{-1})	Intensity (km/mol)	Frequency (cm^{-1})	Intensity (km/mol)
5.1452	0.3058	940.6027	1.0661
10.2538	0.6214	989.781	0.0005
25.1481	1.2537	1063.6807	0.2493
58.0365	1.2642	1106.1598	8.8708
65.0157	1.4276	1128.3739	0.101
123.1546	6.7937	1264.5514	31.9284
238.6466	0.177	1378.2343	4.2367
392.5495	2.9578	1468.2242	46.3184
399.9962	0.1617	1502.5066	11.2677
538.033	0.4585	3142.8213	11.9418
679.5745	92.4342	3153.4494	12.1847
715.3068	20.634	3186.9038	7.686
808.9938	0.0809	3199.5474	8.3904
809.829	1.8419		

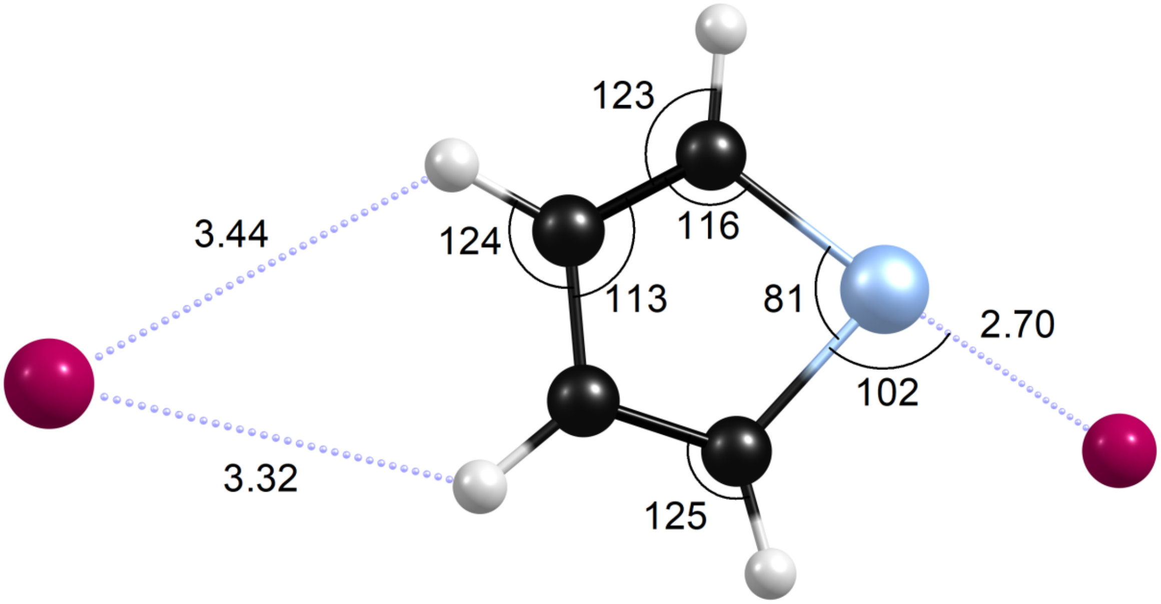

Figure S29. The optimized geometry of isomer 2aIII-doublet $Pt^+(C_2H_2)_2Ar_2$ followed by its predicted frequencies (cm^{-1}) and IR intensities (km/mol).

Frequency (cm^{-1})	Intensity (km/mol)	Frequency (cm^{-1})	Intensity (km/mol)
4.8087	1.119	941.0904	0.5568
7.8463	1.5021	991.2379	0
20.2029	0.7456	1062.944	0.1573
58.0379	1.0595	1104.4261	7.0925
64.4108	1.2071	1127.0113	0.3186
122.874	7.0048	1263.0084	33.5397
238.2372	0.3876	1377.5949	2.4824
392.7737	3.7872	1467.9486	44.0273
399.6905	0.0786	1502.2323	15.6279
537.7055	0.4301	3142.9582	7.5018
678.3128	93.1346	3154.9121	3.1957
715.0555	23.3304	3188.5743	5.6371
806.3235	0.0363	3200.9196	10.9771
809.4088	2.2216		

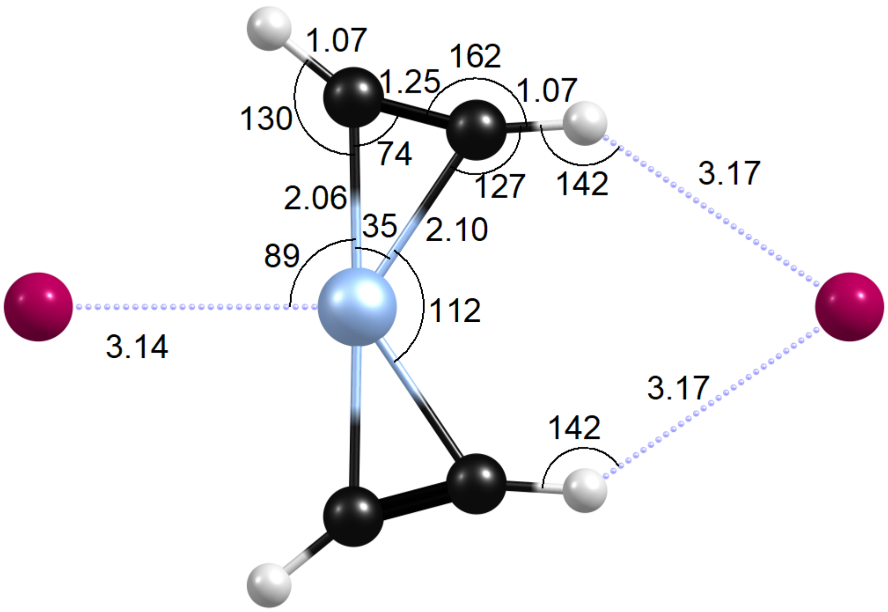

Figure S30. The optimized geometry of isomer 2bI-doublet $Pt^{+}(C_2H_2)_2Ar_2$ followed by its predicted frequencies (cm^{-1}) and IR intensities (km/mol).

Frequency (cm^{-1})	Intensity (km/mol)	Frequency (cm^{-1})	Intensity (km/mol)
7.2875	1.0141	717.3552	0
8.4464	0.774	755.3194	0.0148
23.845	0.6878	755.3708	3.9967
46.3987	0.1659	765.3586	102.589
48.4007	2.6787	820.2677	17.8822
53.9038	0	828.1963	5.4438
57.5613	0.6038	851.3676	85.8469
78.9618	9.2572	1762.862	8.6218
142.2279	6.8555	1791.4657	0.2218
261.281	3.4037	3283.2307	69.8649
437.9891	4.1709	3285.0446	245.2144
475.0705	5.2288	3353.4821	78.8956
523.1467	17.3307	3356.5262	43.3563
709.7308	17.9423		

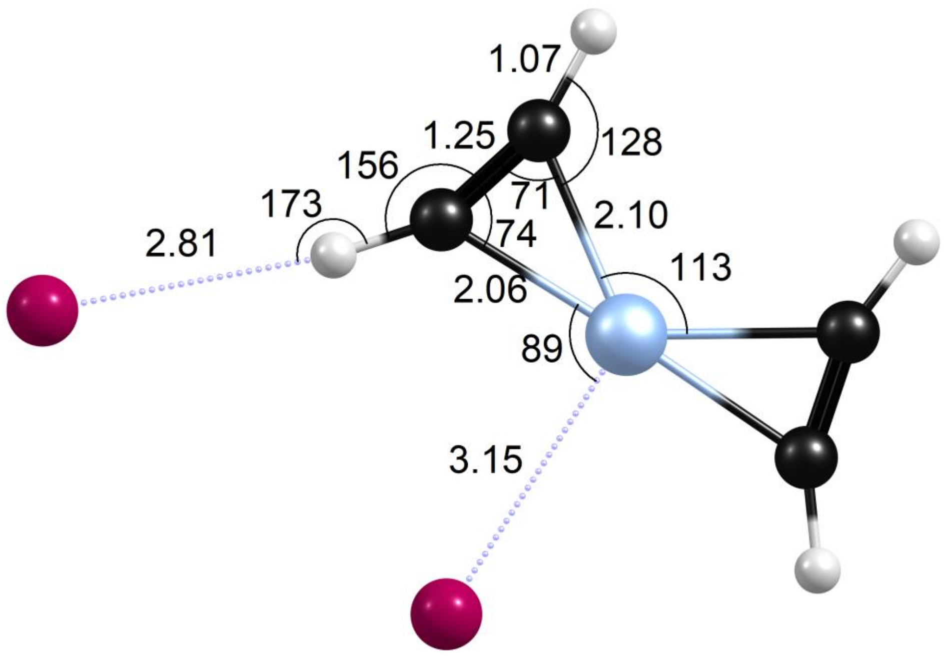

Figure S31. The optimized geometry of isomer 2bII-doublet $Pt^{+}(C_2H_2)_2Ar_2$ followed by its predicted frequencies (cm^{-1}) and IR intensities (km/mol).

Frequency (cm^{-1})	Intensity (km/mol)	Frequency (cm^{-1})	Intensity (km/mol)
9.4763	0.014	715.0082	0.3287
9.9028	0.0222	757.6534	22.2674
30.3125	1.1592	759.5456	3.2129
46.8942	0.0055	764.6717	89.5264
47.4182	2.5116	825.0883	15.8154
49.1829	0.757	829.6082	3.7532
57.2076	1.9418	852.833	63.4323
81.3929	7.5653	1763.9986	14.2282
142.0895	5.1038	1792.1148	1.8631
260.5042	4.402	3270.9451	224.3352
437.5013	3.7671	3285.1959	152.1873
474.4563	4.8208	3352.0676	90.3492
520.9428	20.5332	3357.0067	48.2511
711.4491	7.7936		

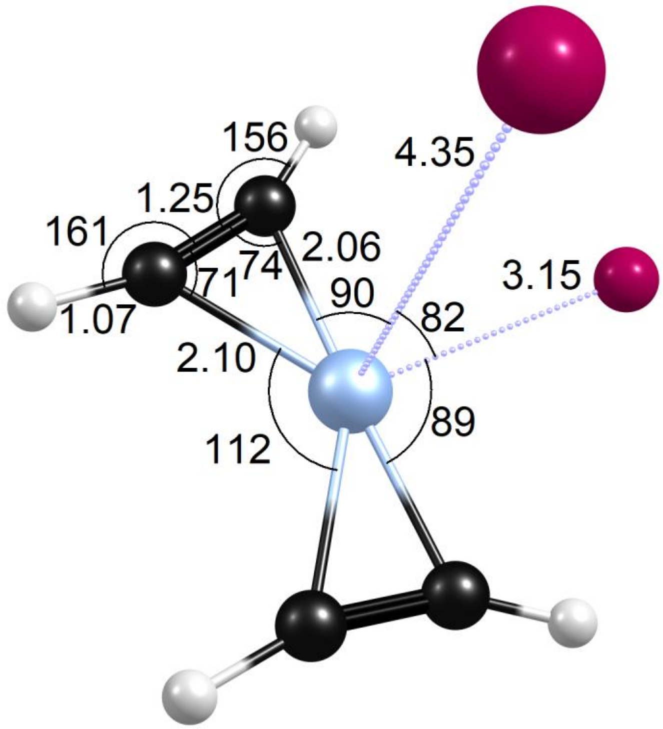

Figure S32. The optimized geometry of isomer 2bIII-doublet $Pt^{+}(C_2H_2)_2Ar_2$ followed by its predicted frequencies (cm^{-1}) and IR intensities (km/mol).

Frequency (cm^{-1})	Intensity (km/mol)	Frequency (cm^{-1})	Intensity (km/mol)
4.9626	0.3195	713.7509	0.0004
6.5114	0.0808	753.8195	0.1859
11.7562	0.8275	754.9629	3.9012
43.546	0.0002	761.8729	116.1084
45.9315	0.0638	820.312	21.7699
47.4477	2.5708	829.7411	3.571
52.7323	0.7894	852.8995	62.027
79.1086	9.4195	1764.2802	10.319
140.9374	4.8682	1792.5497	0.5973
260.0213	3.8482	3284.3469	63.1066
437.446	4.389	3286.0253	218.175
474.0176	5.3805	3355.651	89.6359
521.5356	18.054	3358.1127	36.6081
708.4707	14.2735		

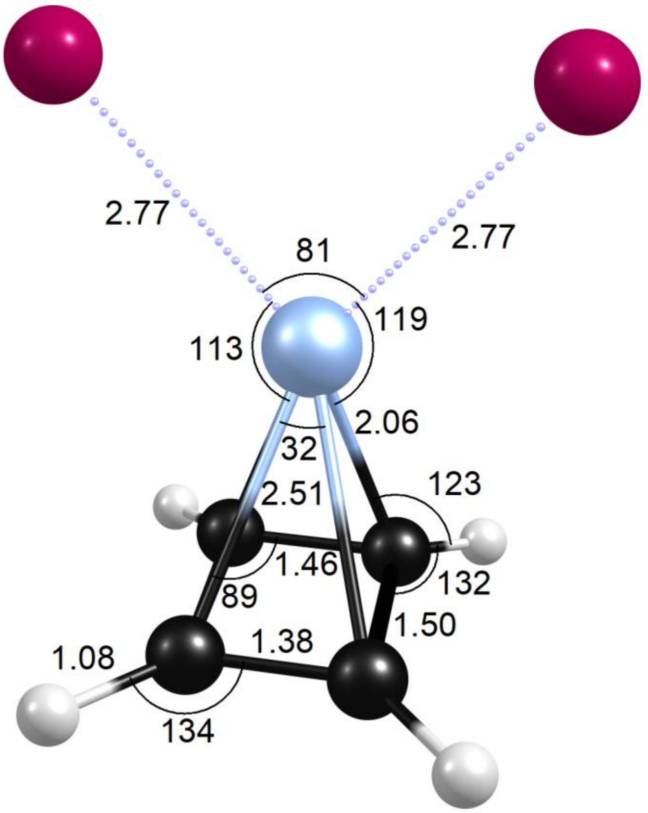

Figure S33. The optimized geometry of isomer 2cI-doublet $Pt^{+}(C_2H_2)_2Ar_2$ followed by its predicted frequencies (cm^{-1}) and IR intensities (km/mol).

Frequency (cm^{-1})	Intensity (km/mol)	Frequency (cm^{-1})	Intensity (km/mol)
49.1172	0.7833	942.8461	6.2431
56.2031	0.1341	943.3958	0.1874
58.1806	0.3186	966.2599	3.3564
59.1878	2.0247	1010.7022	3.891
90.826	5.2873	1164.4257	1.8777
108.7452	9.6995	1201.9286	1.1868
161.6191	2.748	1278.7427	4.9501
261.0899	0.48	1311.8824	32.4883
487.5021	8.3245	1455.6133	31.6579
571.5111	1.7144	3214.322	0.0571
779.2429	69.2191	3227.5217	18.3785
848.6715	15.0779	3233.144	14.5147
878.3037	12.4358	3246.0514	2.8624
897.6434	2.754		

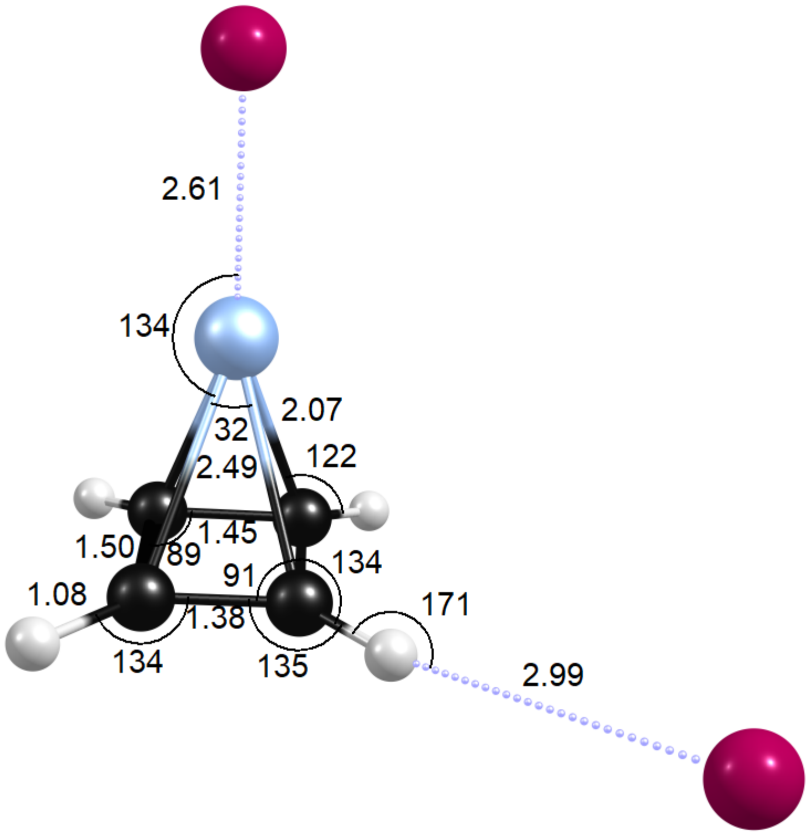

Figure S34. The optimized geometry of isomer 2cII-doublet $Pt^+(C_2H_2)_2Ar_2$ followed by its predicted frequencies (cm^{-1}) and IR intensities (km/mol).

Frequency (cm^{-1})	Intensity (km/mol)	Frequency (cm^{-1})	Intensity (km/mol)
4.4687	0.4057	941.039	0.3256
9.3268	1.2691	947.9673	7.6609
18.8373	0.2062	966.4224	3.6444
29.6273	2.8955	1015.6255	4.1698
60.7567	2.7067	1160.4766	2.7159
129.9723	8.4535	1203.794	1.1395
177.197	1.3954	1303.4982	7.1896
242.8228	1.2753	1308.6811	32.6317
479.5423	10.8097	1457.5207	32.0178
555.4295	2.3054	3215.8157	1.332
779.4524	61.0792	3229.3224	34.0881
848.1783	13.0999	3236.0277	22.274
874.5272	7.6878	3247.9529	6.3764
894.054	2.061		

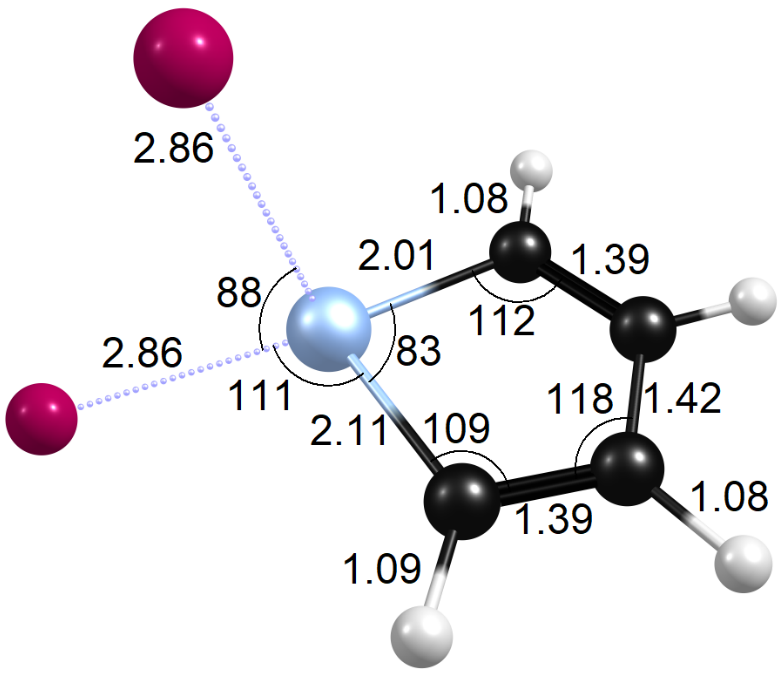

Figure S35. The optimized geometry of isomer 2aI-quartet $Pt^+(C_2H_2)_2Ar_2$ followed by its predicted frequencies (cm^{-1}) and IR intensities (km/mol).

Frequency (cm^{-1})	Intensity (km/mol)	Frequency (cm^{-1})	Intensity (km/mol)
30.3286	2.4338	979.4541	0.3824
34.4388	0.0439	1002.013	0.4985
38.9001	1.4578	1015.0851	0.2611
69.3974	0.1998	1095.4437	18.0204
79.9464	5.06	1123.2407	0.982
91.805	6.812	1234.2662	8.5037
183.4627	0.2724	1325.1674	4.0875
280.4904	0.8931	1467.8254	56.8591
312.7936	5.1843	1475.2066	15.9086
390.5156	1.4159	3147.7789	0.6098
580.7866	16.8255	3158.9601	3.1099
647.8642	26.7497	3166.5797	4.5324
690.3966	43.2106	3176.3662	2.3494
761.8046	3.2337		

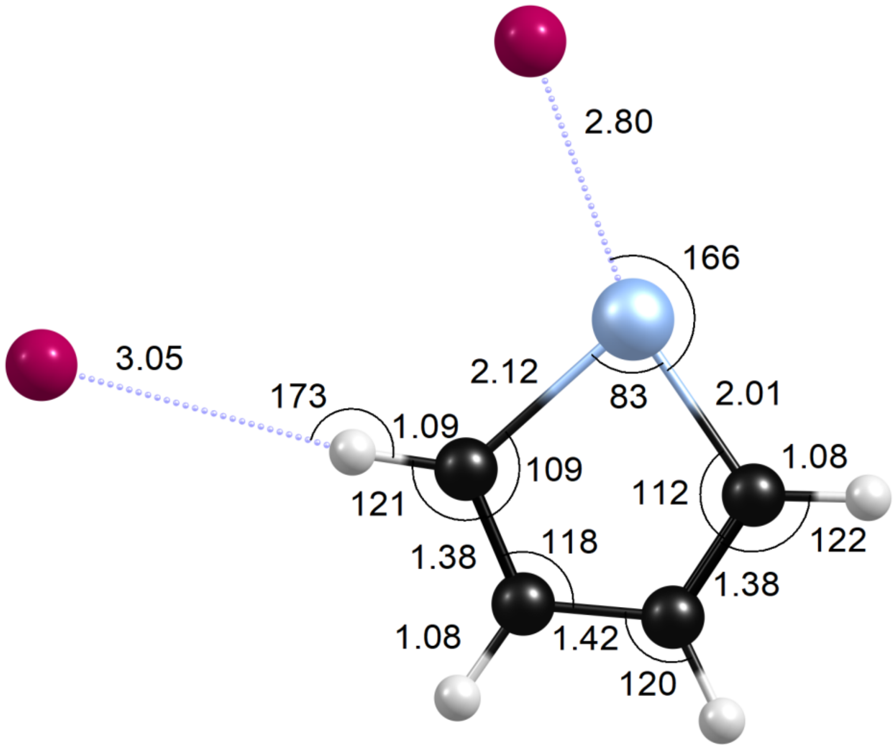

Figure S36. The optimized geometry of isomer 2aII-quartet $Pt^+(C_2H_2)_2Ar_2$ followed by its predicted frequencies (cm^{-1}) and IR intensities (km/mol).

Frequency (cm^{-1})	Intensity (km/mol)	Frequency (cm^{-1})	Intensity (km/mol)
5.0009	0.4004	987.3755	0.3426
8.0348	0.7859	1006.2164	1.3898
22.6136	0.8589	1013.5931	0.0037
31.6625	0.9916	1095.8406	23.9621
37.4233	3.2343	1126.2651	0.1346
95.962	6.3537	1237.6594	9.8351
119.2902	0.237	1329.4679	3.8478
246.6138	0.1034	1474.2597	72.7215
271.2219	1.6197	1487.7336	2.1205
384.6768	1.9293	3148.8182	3.8376
607.3887	5.8685	3159.6146	4.6662
622.4244	3.4622	3167.3174	11.1796
681.3527	61.2295	3176.4748	3.1446
755.7414	0.4592		

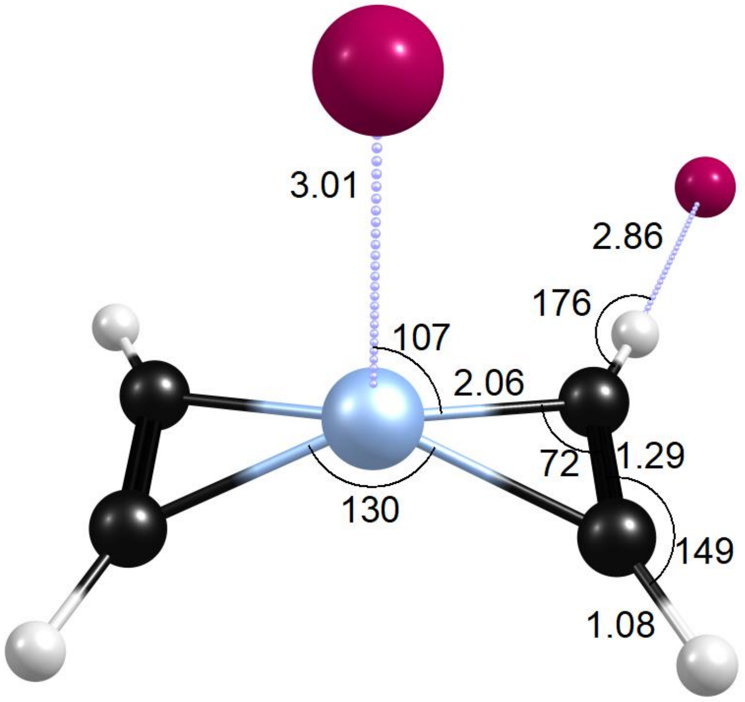

Figure S37. The optimized geometry of isomer 2bI-quartet $Pt^+(C_2H_2)_2Ar_2$ followed by its predicted frequencies (cm^{-1}) and IR intensities (km/mol).

Frequency (cm^{-1})	Intensity (km/mol)	Frequency (cm^{-1})	Intensity (km/mol)
7.6061	0.222	669.8132	65.7057
9.5246	0.2104	746.3166	21.5422
31.3375	0.6397	793.7008	0.1029
37.8347	0.1774	824.7607	11.2359
53.4589	5.2655	864.0516	0.177
69.9658	7.2397	874.6425	15.6811
109.6446	6.4162	878.767	103.7826
133.3573	13.4173	1539.7146	6.3216
237.3699	0.0193	1638.6646	4.1627
375.6271	3.5656	3197.7824	125.2876
442.8242	0.0138	3202.5074	37.8478
508.8042	1.1698	3246.4663	78.1605
521.5806	0.1328	3249.9075	22.4569
645.4098	8.5636		

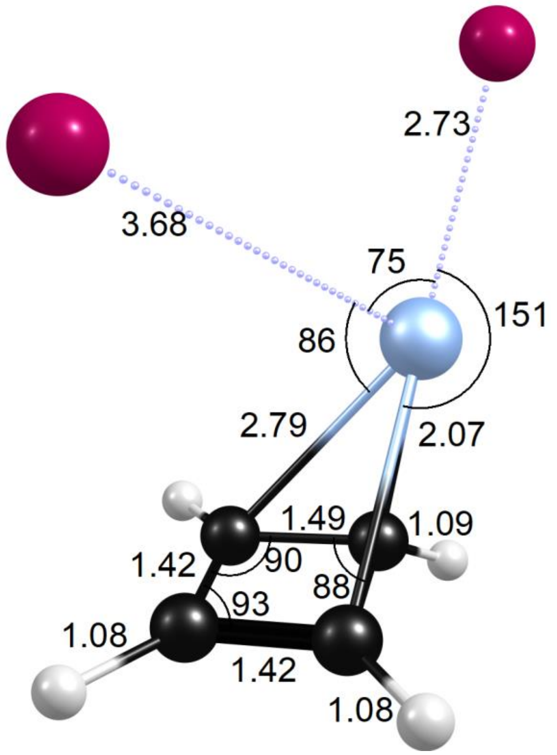

Figure S38. The optimized geometry of isomer 2cI-quartet $Pt^{+}(C_2H_2)_2Ar_2$ followed by its predicted frequencies (cm^{-1}) and IR intensities (km/mol).

Frequency (cm^{-1})	Intensity (km/mol)	Frequency (cm^{-1})	Intensity (km/mol)
14.8301	0.6393	911.5488	29.0105
17.9746	0.6533	940.2383	4.3599
22.6425	0.6696	981.2392	6.4658
43.199	1.729	1007.0751	41.7686
53.2197	0.6614	1178.5679	4.9773
88.8356	2.2311	1209.9052	2.5667
114.6204	4.9954	1213.0266	0.6479
158.3112	0.0671	1340.9092	8.4438
336.5497	2.1911	1354.8254	90.8852
555.5913	12.3773	3118.9746	4.2346
628.0593	0.3842	3210.9769	4.2763
635.3739	107.1828	3251.2559	22.9616
880.8077	11.7252	3258.4745	0.1208
888.4649	1.564		

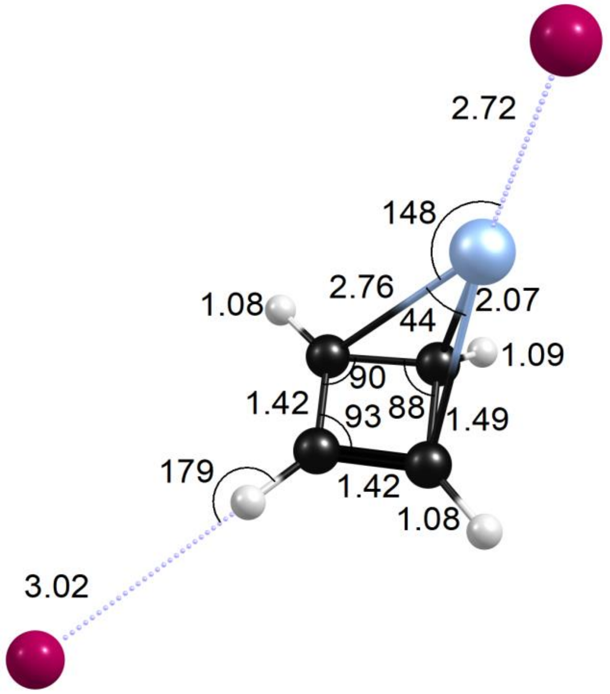

Figure S39. The optimized geometry of isomer 2cII-quartet $Pt^{+}(C_2H_2)_2Ar_2$ followed by its predicted frequencies (cm^{-1}) and IR intensities (km/mol).

Frequency (cm^{-1})	Intensity (km/mol)	Frequency (cm^{-1})	Intensity (km/mol)
5.0661	0.6759	913.2307	21.8469
9.969	1.053	941.1094	3.5194
24.3104	1.2201	982.5266	4.9941
42.5913	1.5846	1004.1348	34.4109
54.3743	0.7512	1183.3253	5.2119
92.5367	3.2132	1209.0799	2.4906
120.5039	4.938	1213.2404	0.4512
155.1315	0.0852	1343.3498	7.0248
340.078	1.4371	1353.3399	93.4955
559.5408	10.8581	3130.9554	5.0491
631.1067	0.4859	3209.8438	16.8932
640.6512	97.8611	3250.263	24.0642
880.593	11.3526	3257.4498	0.1139
887.1031	2.6111		

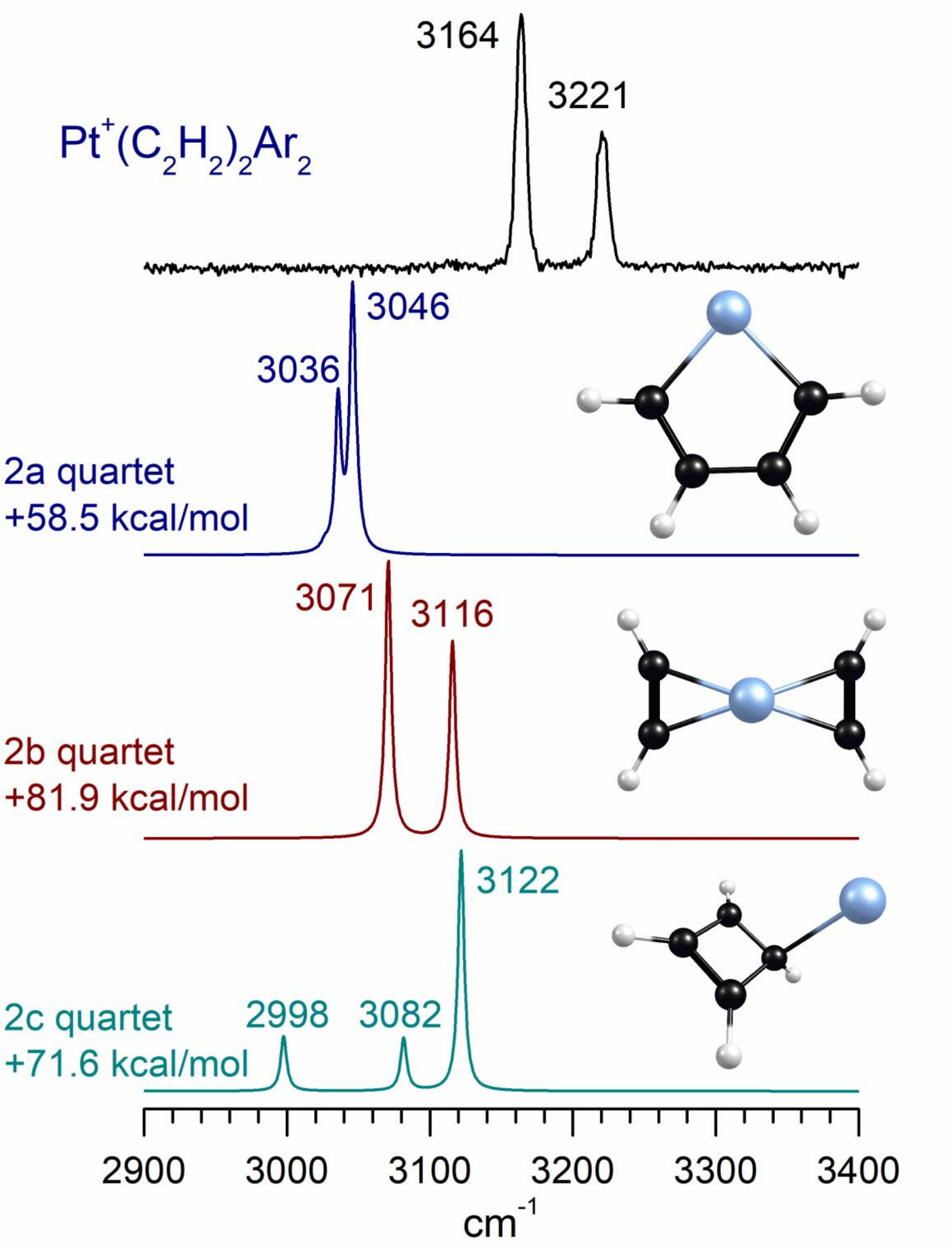
3164
3221
Pt$^+$(C$_2$H$_2$)$_2$Ar$_2$
3046
3036
2a quartet
+58.5 kcal/mol
3071
3116
2b quartet
+81.9 kcal/mol
3122
2998
3082
2c quartet
+71.6 kcal/mol
2900
3000
3100
3200
3300
3400
cm^{-1}

S40.

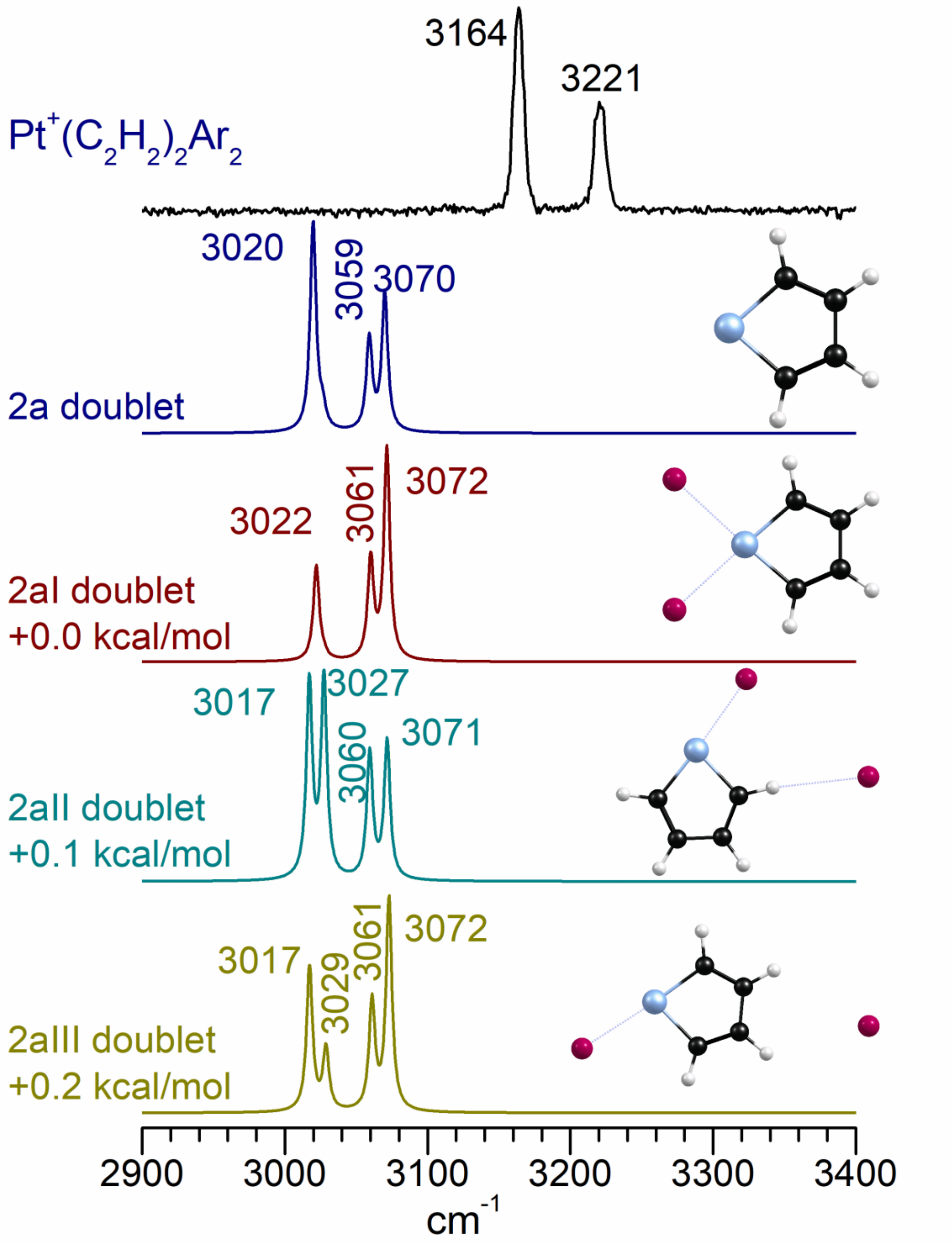
3164
3221
Pt$^+$(C$_2$H$_2$)$_2$Ar$_2$
3020
3059
3070
2a doublet
3022
3061
3072
2aI doublet
+0.0 kcal/mol
3017
3027
3060
3071
2aII doublet
+0.1 kcal/mol
3017
3029
3061
3072
2aIII doublet
+0.2 kcal/mol
2900
3000
3100
3200
3300
3400
cm^{-1}

S41.

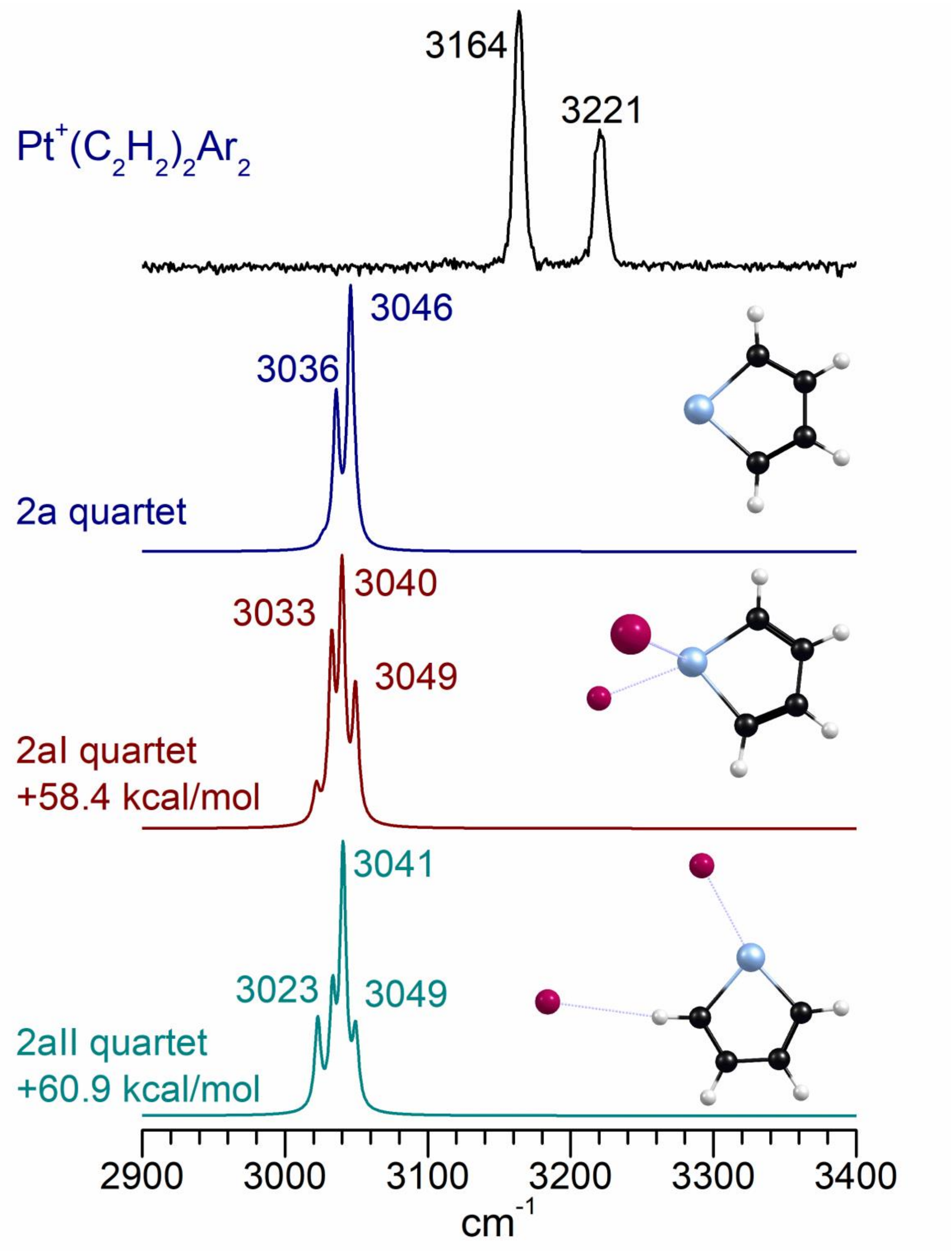

3164
3221
Pt$^+$(C$_2$H$_2$)$_2$Ar$_2$
3046
3036
2a quartet
3040
3033
3049
2aI quartet
+58.4 kcal/mol
3041
3023
3049
2aII quartet
+60.9 kcal/mol
2900
3000
3100
3200
3300
3400
cm^{-1}

S42.

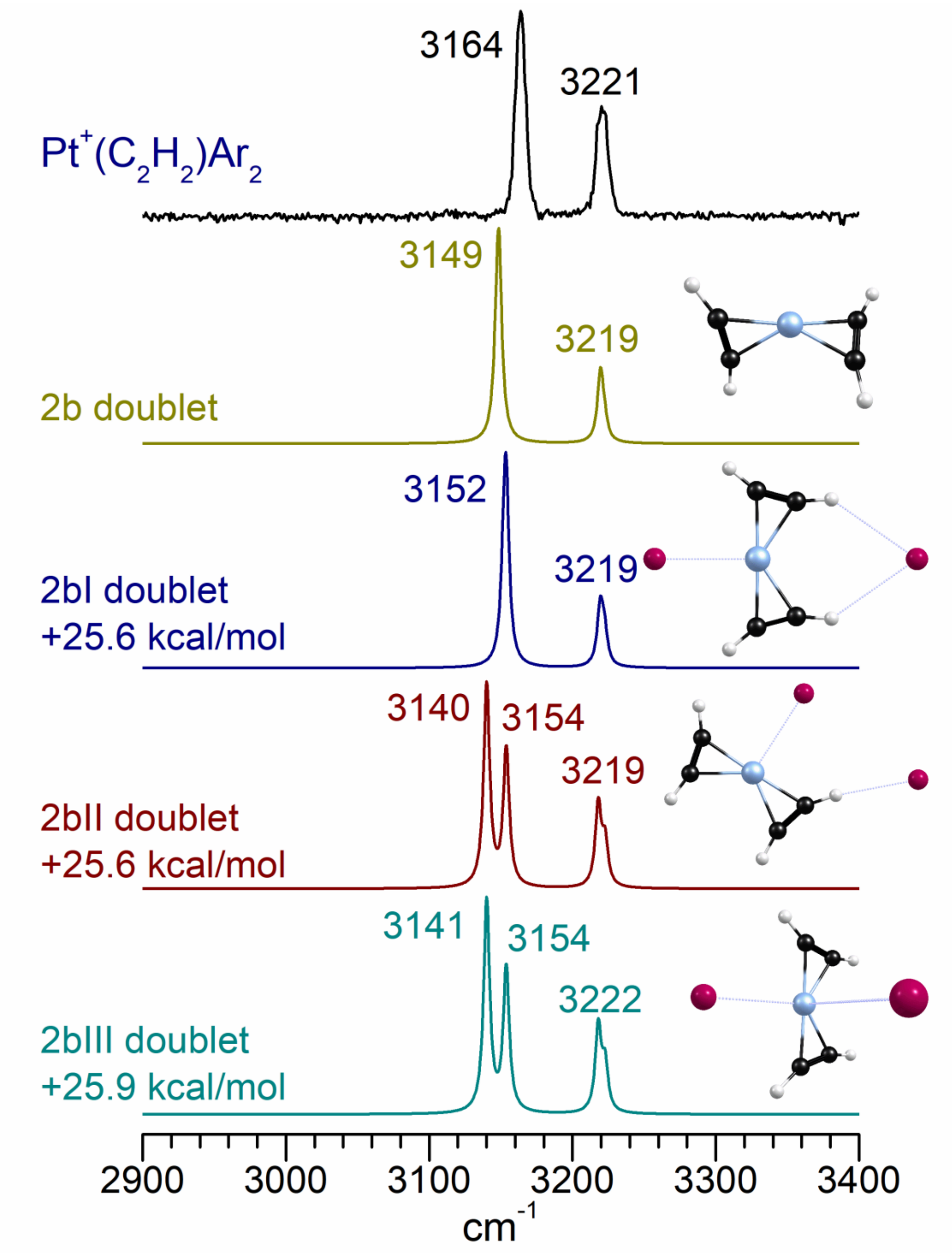
3164
3221
$Pt^+(C_2H_2)Ar_2$
3149
3219
2b doublet
3152
3219
2bI doublet
+25.6 kcal/mol
3140
3154
3219
2bII doublet
+25.6 kcal/mol
3141
3154
3222
2bIII doublet
+25.9 kcal/mol
2900
3000
3100
3200
3300
3400
cm^{-1}

S43.

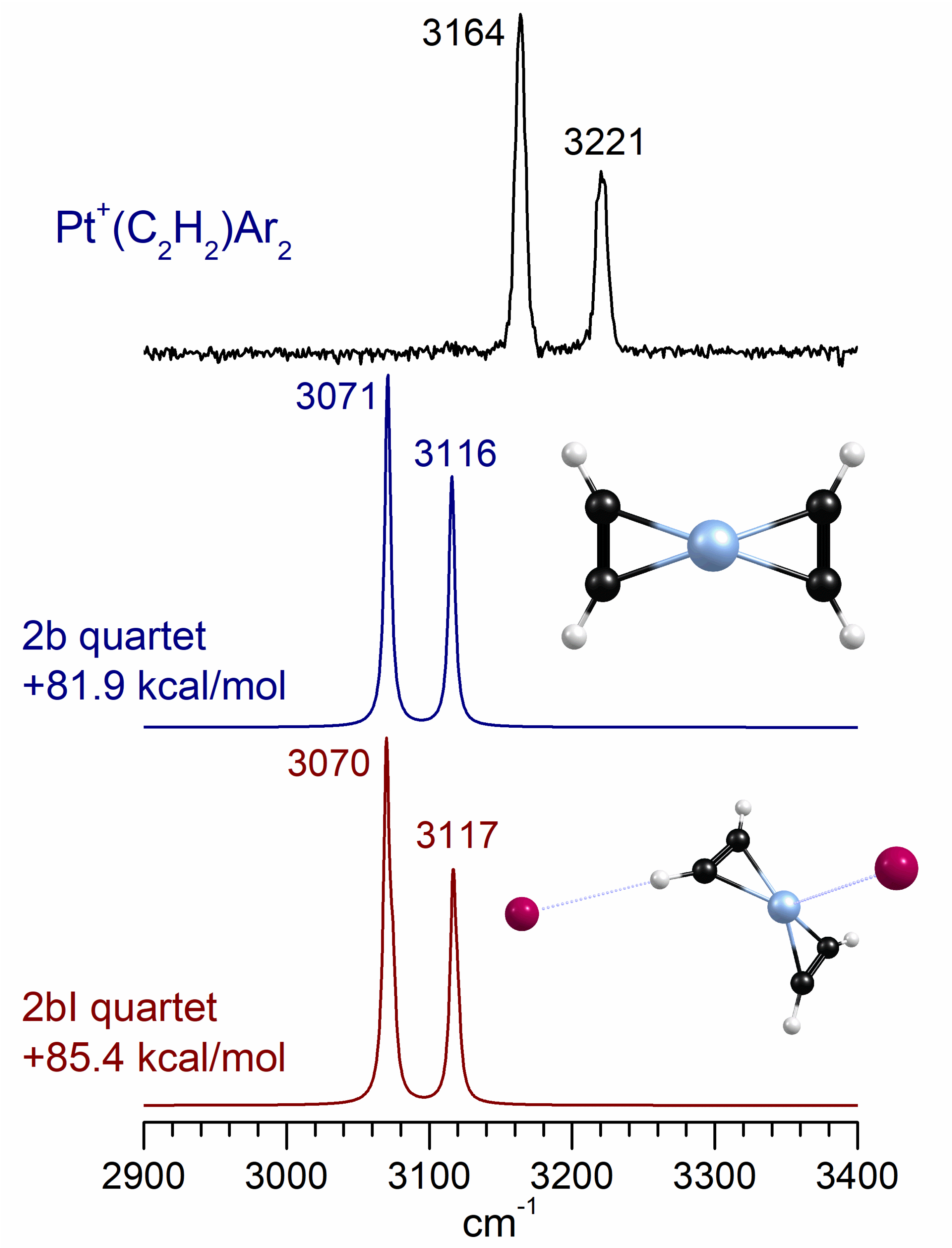
3164
3221
Pt+(C2H2)Ar2
3071
3116
2b quartet
+81.9 kcal/mol
3070
3117
2bI quartet
+85.4 kcal/mol
2900
3000
3100
3200
3300
3400
cm-1

S44.

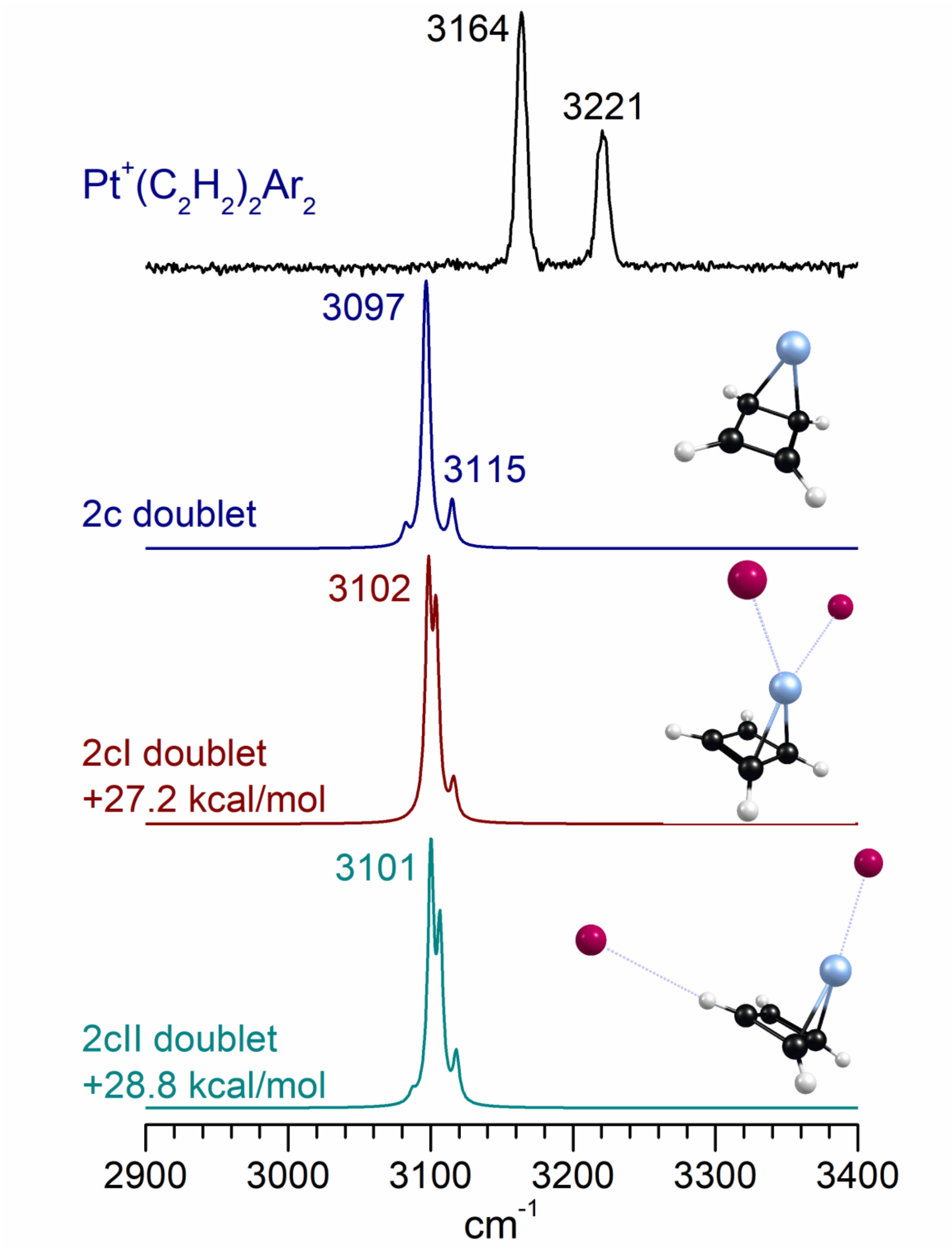

3164
3221
Pt+(C2H2)2Ar2
3097
3115
2c doublet
3102
2cI doublet
+27.2 kcal/mol
3101
2cII doublet
+28.8 kcal/mol
2900
3000
3100
3200
3300
3400
cm-1

S45.

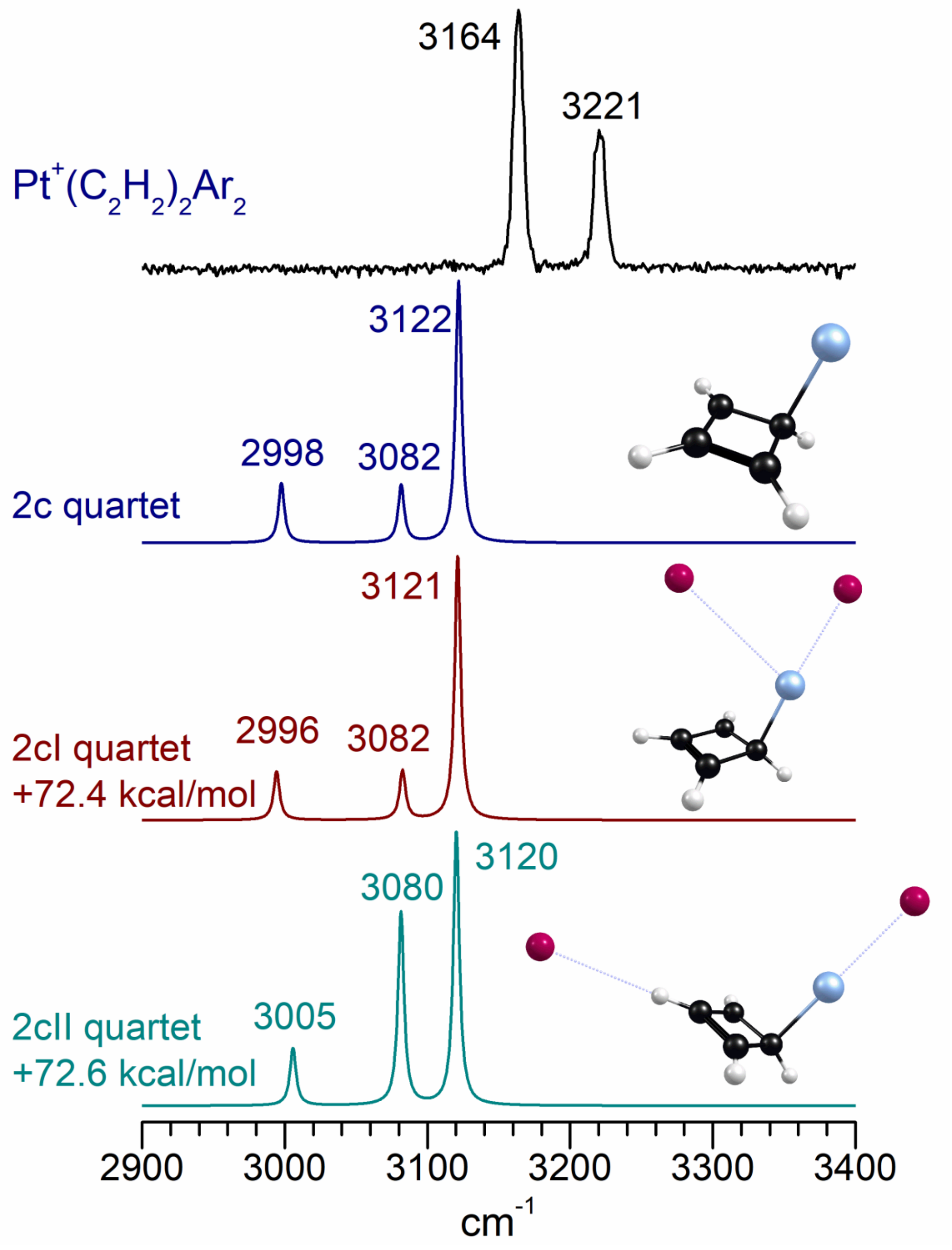

3164
3221
Pt+(C2H2)2Ar2
3122
2998
3082
2c quartet
3121
2996
3082
2cI quartet
+72.4 kcal/mol
3120
3080
3005
2cII quartet
+72.6 kcal/mol
2900
3000
3100
3200
3300
3400
cm-1

S46.

Table S7.

Isomer	2s + 1	E (hartree)	Relative E (kcal/mol)
3a	2	-351.433277	0
3a	4	-351.384757	30.44676618
3b	2	-351.406341	16.9025988
3b	4	-351.322507	69.50923928
3c	2	-351.36955	39.98930479
3c	4	-351.295487	86.46454889
3d	2	-351.360337	45.77055081
3d	4	-351.28296	94.32536175
3e	2	-351.350917	51.68169132
3e	4	-351.283406	94.04549247
3f	2	-351.314674	74.42452204
3f	4	-351.234949	124.4527255
3g	2	-351.312461	75.8132008
3g	4	-351.100356	208.9111262
TS1	2	-351.284526	93.3426817
TS2	2	-351.312329	75.89603207

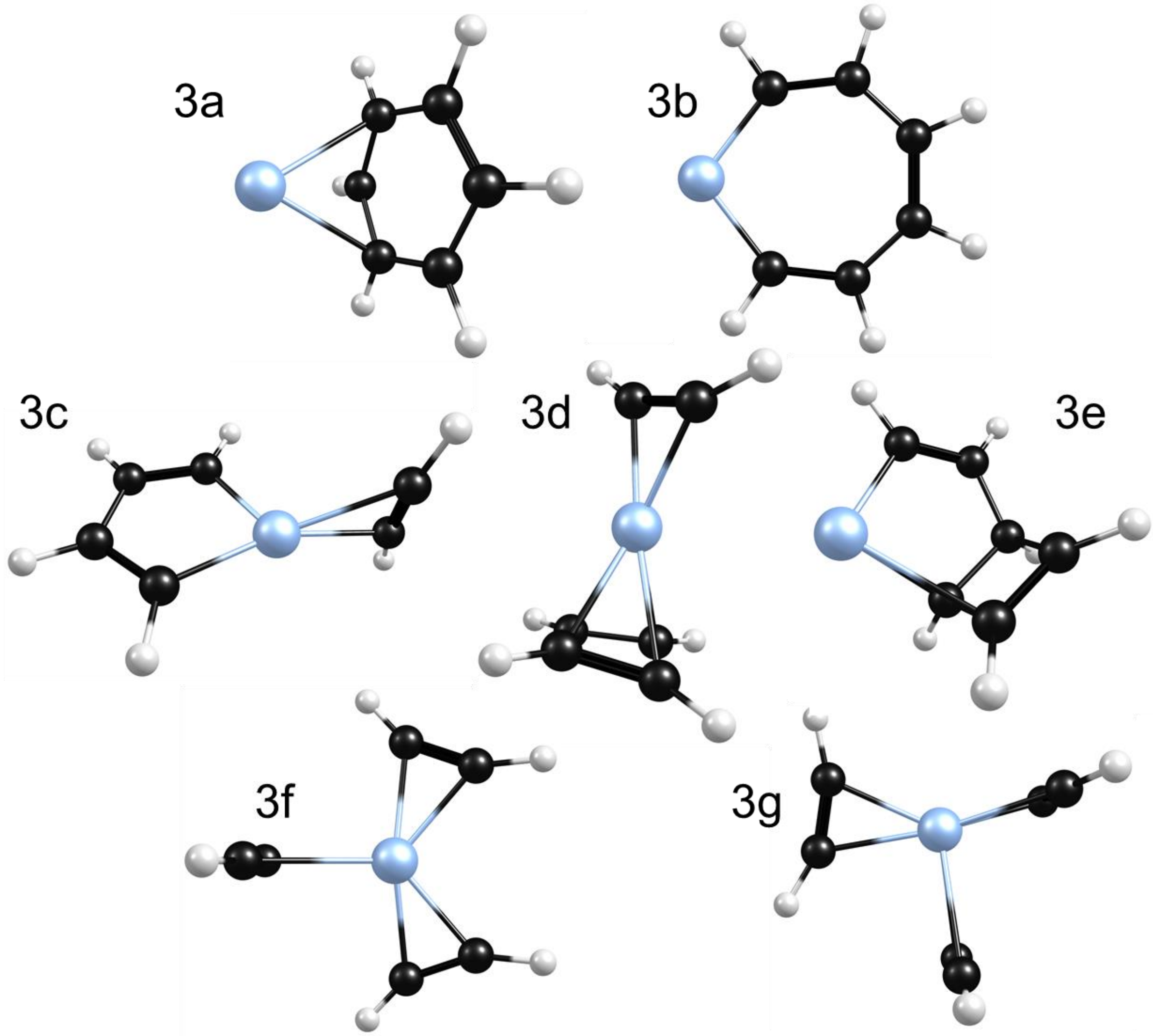

Doublets S47.

Table S8. Cartesian coordinates for the optimized geometry of isomer 3a-doublet of $Pt^{+}(C_2H_2)_3$ followed by its predicted frequencies (cm^{-1}) and IR intensities (km/mol).

Z	x	y	z
78	0.798623000	0.000001000	-0.152802000
6	-2.471308000	0.000073000	-0.725295000
6	-1.946835000	1.200290000	-0.261293000
6	-1.946923000	-1.200216000	-0.261385000
1	-2.292595000	2.141443000	-0.666232000
1	-2.292765000	-2.141308000	-0.666392000
6	-0.956482000	1.217557000	0.754806000
6	-0.956556000	-1.217635000	0.754696000
1	-0.641416000	2.162812000	1.173454000
1	-0.641533000	-2.162948000	1.173245000
6	-0.567837000	-0.000081000	1.404655000
1	-0.094872000	-0.000140000	2.377265000
1	-3.253797000	0.000129000	-1.469914000

Frequency (cm^{-1})	Intensity (km/mol)	Frequency (cm^{-1})	Intensity (km/mol)
108.9735	1.3393	1052.0428	0.5615
124.4919	1.9121	1159.3954	1.8551
272.8528	0.6402	1187.9326	0.2933
330.5121	1.9822	1189.7866	1.1303
482.2185	0.6236	1317.4871	2.5734
581.06	0.0141	1379.1069	0.3987
593.4877	0.3989	1466.0345	24.3536
649.6367	11.3746	1484.2494	18.0843
763.0124	59.8979	1537.7241	14.0051
905.3273	1.9871	1556.1526	3.4239
918.7886	2.7501	3186.4345	2.2498
957.5624	9.8756	3193.6912	2.8945
989.2987	1.7103	3195.7857	0.6283
994.928	4.4759	3205.5549	9.2138
1013.8466	3.4361	3205.8436	6.0607
1024.2493	0.6878	3215.1288	1.0823
1029.6437	0.4449		

Table S9. Cartesian coordinates for the optimized geometry of isomer 3b-doublet of $Pt^{+}(C_2H_2)_3$ followed by its predicted frequencies (cm^{-1}) and IR intensities (km/mol).

Z	x	y	z
6	-2.683602000	-0.700943000	-0.000018000
6	-1.660496000	-1.652542000	0.000017000
6	-0.296454000	-1.500100000	0.000040000
6	-2.683599000	0.700944000	-0.000023000
6	-1.660492000	1.652543000	0.000015000
6	-0.296450000	1.500106000	0.000044000
1	-1.995446000	2.685699000	0.000024000
1	-3.677776000	-1.130800000	-0.000041000
1	-1.995447000	-2.685699000	0.000029000
1	0.323107000	-2.403840000	0.000085000
1	-3.677772000	1.130804000	-0.000053000
1	0.323108000	2.403847000	0.000099000
78	0.851113000	-0.000001000	-0.000008000

Frequency (cm^{-1})	Intensity (km/mol)	Frequency (cm^{-1})	Intensity (km/mol)
61.2939	0.0742	1068.0681	3.7216
202.9585	0	1199.5978	8.5661
215.3172	0.1858	1249.5791	22.3854
339.4483	0.4271	1304.9811	1.427
365.0077	1.4807	1332.5572	0.5051
550.4129	0.3719	1398.1194	2.7774
552.9496	0	1483.0925	39.4469
573.0799	3.9096	1510.1356	17.5048
651.7674	107.2168	1562.6947	13.2331
698.7211	0	1572.1521	35.5475
734.5131	13.5793	3037.3116	12.4382
823.8309	0.4019	3042.7764	0.122
854.2945	0.1628	3135.2006	8.0297
895.0641	1.0011	3135.9598	1.9916
939.1341	0	3166.3921	1.3844
1001.4289	0.8739	3180.6574	0.9046
1046.0134	0		

Table S10. Cartesian coordinates for the optimized geometry of isomer 3c-doublet of $Pt^{+}(C_2H_2)_3$ followed by its predicted frequencies (cm^{-1}) and IR intensities (km/mol).

Z	x	y	z
78	-0.258873000	0.154959000	-0.000001000
6	0.984454000	-1.332946000	0.000000000
6	2.340374000	-1.061377000	0.000001000
1	3.088009000	-1.845271000	0.000002000
1	0.575175000	-2.338092000	-0.000003000
6	2.637183000	0.317786000	0.000003000
6	1.499590000	1.106728000	0.000002000
1	1.566588000	2.191012000	0.000003000
1	3.646193000	0.710358000	0.000006000
6	-2.356188000	-0.346620000	-0.612111000
6	-2.356185000	-0.346617000	0.612121000
1	-2.589617000	-0.413249000	1.654149000
1	-2.589633000	-0.413255000	-1.654136000

Frequency (cm^{-1})	Intensity (km/mol)	Frequency (cm^{-1})	Intensity (km/mol)
43.8795	3.1278	963.6688	0.9293
52.9223	3.1967	998.3008	0.0902
126.5279	0.7747	1065.063	1.2303
203.0435	1.0381	1112.0248	6.5894
251.763	15.854	1128.427	0.2643
354.5122	3.9527	1277.7275	19.1931
376.8999	2.672	1383.8013	6.4033
385.2836	8.3822	1473.4718	50.1579
527.6578	5.2069	1511.2733	14.2501
674.7919	59.5094	1919.9593	2.9469
683.4526	8.7891	3140.7878	1.1231
688.0398	0.0017	3154.5697	2.1415
749.3408	34.7543	3181.873	2.4757
773.2161	67.3115	3194.3207	2.8967
798.6996	4.4601	3324.0947	180.6738
805.4119	0.0504	3410.1787	73.4398
818.9538	3.5184		

Table S11. Cartesian coordinates for the optimized geometry of isomer 3d-doublet of $Pt^+(C_2H_2)_3$ followed by its predicted frequencies (cm^{-1}) and IR intensities (km/mol).

Z	x	y	z
6	1.669643000	0.000065000	-1.019002000
6	1.798194000	1.031591000	0.000005000
6	1.669711000	0.000007000	1.018957000
6	1.798285000	-1.031505000	-0.000051000
1	1.715296000	0.000098000	-2.096395000
1	1.940667000	2.098877000	0.000032000
1	1.715401000	-0.000022000	2.096349000
1	1.940835000	-2.098781000	-0.000083000
78	-0.228091000	-0.000026000	0.000012000
6	-2.157080000	0.633496000	-0.000014000
6	-2.157140000	-0.633376000	-0.000029000
1	-2.625464000	-1.600697000	-0.000015000
1	-2.625305000	1.600864000	-0.000039000

Frequency (cm^{-1})	Intensity (km/mol)	Frequency (cm^{-1})	Intensity (km/mol)
93.1875	7.2348	927.7771	7.4057
105.3893	8.7747	934.4372	5.3332
171.4483	0	952.973	4.0762
327.325	6.4504	977.5909	1.1037
356.0093	10.6842	1148.0493	0
357.4007	15.5849	1206.1251	0
444.8189	4.0819	1249.3973	0.0063
542.1688	0.2598	1332.6658	13.4393
549.4952	0.9455	1341.5551	25.6056
741.5603	0.5264	1727.3092	6.3205
744.8195	70.4816	3234.3231	0.9179
758.8306	0	3242.9031	21.8833
774.1268	47.0001	3258.4324	52.0894
826.3846	1.361	3263.4739	79.1577
854.6961	55.1051	3267.7374	8.8583
900.1373	0	3322.514	67.4929
916.2646	3.8012		

Table S12. Cartesian coordinates for the optimized geometry of isomer 3e-doublet of $Pt^+(C_2H_2)_3$ followed by its predicted frequencies (cm^{-1}) and IR intensities (km/mol).

Z	x	y	z
6	-0.967623000	-0.673535000	0.907786000
6	-2.071392000	0.333805000	0.421414000
6	-2.261642000	-0.674467000	-0.701800000
6	-1.282028000	-1.531187000	-0.236041000
1	-0.788083000	-0.966298000	1.937274000
1	-2.933749000	0.430116000	1.080912000
1	-2.946259000	-0.714687000	-1.538462000
1	-0.964130000	-2.519401000	-0.537082000
78	0.751089000	-0.074799000	-0.019830000
6	-1.473417000	1.647277000	0.016950000
6	-0.167043000	1.658423000	-0.218790000
1	0.467239000	2.501513000	-0.471183000
1	-2.081080000	2.541198000	-0.061820000

Frequency (cm^{-1})	Intensity (km/mol)	Frequency (cm^{-1})	Intensity (km/mol)
88.9793	0.5115	1021.5871	4.8106
201.9542	1.0342	1093.1893	14.5529
276.6158	2.619	1140.4332	4.5747
314.049	0.6925	1173.1652	8.3652
437.0573	5.7614	1208.1712	1.987
492.4876	4.8586	1268.2666	53.0011
602.6212	20.8243	1300.2265	3.8594
697.1891	62.245	1328.4612	24.319
707.9821	9.1883	1434.3953	42.05
770.8444	38.3865	1600.6948	2.945
870.5857	4.5359	3085.7822	0.5887
886.0846	0.8872	3146.7306	4.9847
890.9746	17.141	3153.2864	11.8783
908.3006	15.2648	3174.6145	11.8508
963.0512	3.8662	3197.0872	5.3295
985.0371	15.7135	3218.3845	2.7746
1006.9305	11.3663		

Table S13. Cartesian coordinates for the optimized geometry of isomer 3f-doublet of $Pt^{+}(C_2H_2)_3$ followed by its predicted frequencies (cm^{-1}) and IR intensities (km/mol).

Z	x	y	z
78	-0.013231000	-0.092991000	0.000000000
1	1.017677000	2.748813000	0.000000000
6	-1.136288000	2.021708000	0.000000000
6	0.042966000	2.313372000	0.000000000
1	-2.201958000	1.952568000	0.000000000
1	0.385550000	0.494898000	2.827935000
6	0.301247000	-1.401870000	1.576356000
6	0.301247000	-0.224128000	2.035579000
1	0.387614000	-2.468185000	1.653982000
1	0.385550000	0.494898000	-2.827935000
6	0.301247000	-0.224128000	-2.035579000
6	0.301247000	-1.401870000	-1.576356000
1	0.387614000	-2.468185000	-1.653982000

Frequency (cm^{-1})	Intensity (km/mol)	Frequency (cm^{-1})	Intensity (km/mol)
27.5714	0.0425	767.4385	44.686
47.7261	1.518	774.5844	14.4631
89.5349	0.0197	802.9727	1.5129
120.411	6.6043	809.2253	47.366
138.8205	10.2158	834.1314	7.6526
184.6386	6.4871	859.6353	8.2811
218.8859	0.4586	901.0734	48.4969
256.7229	6.5766	1708.3967	5.5432
372.7187	0.0069	1738.508	0.2262
451.7972	2.0299	1969.9307	7.842
514.4816	0.1145	3285.418	31.5149
581.2928	11.2712	3285.9319	120.9554
677.8529	0.771	3344.0657	63.7022
730.2043	2.0608	3344.7324	20.8432
735.5258	0.8045	3350.6788	178.3139
753.8453	18.4684	3442.4551	27.2874
756.6221	96.3805		

Table S14. Cartesian coordinates for the optimized geometry of isomer 3g-doublet of $Pt^+(C_2H_2)_3$ followed by its predicted frequencies (cm^{-1}) and IR intensities (km/mol).

Z	x	y	z
78	0.026105000	-0.176346000	0.000005000
6	-1.921875000	-0.726372000	-0.622138000
6	-1.922021000	-0.725824000	0.622225000
1	-2.282219000	-0.826442000	1.626504000
1	-2.281817000	-0.827889000	-1.626418000
6	2.127488000	0.151239000	-0.000229000
6	1.933361000	-1.077332000	0.000173000
1	2.129865000	-2.131231000	0.000508000
1	2.695649000	1.058852000	-0.000531000
6	-0.255060000	2.171873000	-0.607552000
6	-0.254634000	2.171916000	0.607465000
1	-0.270018000	2.344435000	1.660837000
1	-0.271190000	2.344263000	-1.660933000

Frequency (cm^{-1})	Intensity (km/mol)	Frequency (cm^{-1})	Intensity (km/mol)
27.6451	0.1357	753.6998	48.1979
67.2701	0.11	767.227	70.6496
98.6919	5.2428	796.3172	40.4699
130.3692	4.7108	814.338	9.0014
132.9023	3.8383	819.8545	31.3327
151.5454	8.2542	821.6039	5.2218
181.7046	1.0836	835.5376	23.2579
189.4564	0.1316	1812.6774	1.1958
366.5104	3.5654	1835.0534	9.9573
390.6761	11.0379	1967.8043	14.9799
465.5353	6.3242	3300.1313	112.668
482.0081	4.4651	3301.1825	99.5083
672.7121	0.1101	3353.6176	182.1304
713.4473	0.4284	3370.9787	68.2521
722.0802	7.7542	3372.5276	47.4349
736.2815	2.7888	3445.933	19.0016
747.8723	49.8987		

Table S15. Cartesian coordinates for the optimized geometry of isomer 3a-quartet of $Pt^{+}(C_2H_2)_3$ followed by its predicted frequencies (cm^{-1}) and IR intensities (km/mol).

Z	x	y	z
78	0.727727000	0.000007000	0.000013000
6	-1.355214000	-0.034849000	1.407199000
6	-1.355177000	-1.236130000	0.673420000
6	-1.355234000	1.201269000	0.733757000
1	-1.329431000	-2.184797000	1.190269000
1	-1.329533000	2.123204000	1.296908000
6	-1.355128000	-1.201296000	-0.733808000
6	-1.355176000	1.236105000	-0.673471000
1	-1.329353000	-2.123230000	-1.296956000
1	-1.329440000	2.184774000	-1.190317000
6	-1.355102000	0.034823000	-1.407249000
1	-1.329268000	0.061556000	-2.487241000
1	-1.329490000	-0.061581000	2.487193000

Frequency (cm^{-1})	Intensity (km/mol)	Frequency (cm^{-1})	Intensity (km/mol)
155.3846	0.2023	1041.2281	0.1164
155.4608	0.2022	1193.9182	0
163.9713	0.4212	1193.9255	0
313.5823	0	1194.3552	0
313.6708	0	1340.7214	0
599.9363	0	1388.7589	0
599.9455	0	1498.5938	38.9471
602.3994	0	1498.601	38.9476
782.3936	83.3917	1570.8634	0
918.7515	0.3515	1570.8755	0
918.7622	0.3517	3199.2287	0
990.1253	3.0972	3205.1696	0
1013.5971	0	3205.1746	0
1013.6045	0	3213.8499	22.5891
1017.4368	0	3213.8548	22.5895
1030.5334	0	3219.846	0.0094
1041.2182	0.1163		

Table S16. Cartesian coordinates for the optimized geometry of isomer 3b-quartet of $Pt^{+}(C_2H_2)_3$ followed by its predicted frequencies (cm^{-1}) and IR intensities (km/mol).

Z	x	y	z
6	2.493704000	-0.708012000	-0.000011000
6	1.547750000	-1.747780000	0.000000000
6	0.166944000	-1.740202000	-0.000014000
6	2.493704000	0.708012000	-0.000035000
6	1.547750000	1.747780000	-0.000024000
6	0.166943000	1.740202000	0.000010000
1	1.999994000	2.736769000	-0.000047000
1	3.507246000	-1.092309000	-0.000005000
1	1.999995000	-2.736769000	0.000021000
1	-0.357277000	-2.693482000	-0.000012000
1	3.507246000	1.092310000	-0.000061000
1	-0.357277000	2.693482000	0.000028000
78	-0.779496000	0.000000000	0.000007000

Frequency (cm^{-1})	Intensity (km/mol)	Frequency (cm^{-1})	Intensity (km/mol)
71.2339	2.4074	1044.5826	0
114.1087	0	1152.7796	16.0572
179.4846	1.1436	1212.7553	7.7993
297.3482	1.4372	1289.2159	15.6615
354.2968	0.6314	1323.775	5.7049
500.5107	0.4526	1383.4206	0.0088
515.1609	0	1470.5954	95.5933
538.6034	0.0095	1483.2038	17.8746
599.2025	88.8334	1541.9285	18.4934
712.3342	8.7328	1543.8914	11.3844
717.0615	0	3107.349	0.1176
826.0665	1.0261	3108.6239	3.3062
837.7178	1.6937	3123.0332	13.7449
846.974	1.5236	3125.0244	0.1315
975.1176	0	3152.3446	1.0446
1014.0393	2.3109	3169.5065	0.3882
1039.8974	5.6246		

Table S17. Cartesian coordinates for the optimized geometry of isomer 3c-quartet of $Pt^{+}(C_2H_2)_3$ followed by its predicted frequencies (cm^{-1}) and IR intensities (km/mol).

Z	x	y	z
78	0.301205000	-0.125816000	-0.188001000
6	-1.121269000	1.367112000	-0.134139000
6	-2.381704000	0.917548000	0.223361000
1	-3.226942000	1.590078000	0.320458000
1	-0.911620000	2.412462000	-0.327017000
6	-2.503863000	-0.470806000	0.452888000
6	-1.353474000	-1.214855000	0.239238000
1	-1.356441000	-2.298583000	0.310627000
1	-3.442503000	-0.920602000	0.754316000
6	2.200175000	-0.242829000	0.660542000
6	1.910438000	0.987213000	0.547014000
1	2.102696000	2.029669000	0.721879000
1	2.839010000	-1.059636000	0.950358000

Frequency (cm^{-1})	Intensity (km/mol)	Frequency (cm^{-1})	Intensity (km/mol)
64.4959	4.4807	975.1154	0.9659
114.8902	4.6364	1000.7837	2.1138
139.6333	1.6263	1032.3473	3.5579
250.3871	1.9701	1099.9295	12.3328
317.135	5.2571	1122.958	0.9287
367.2982	0.6782	1238.4801	7.1182
404.6523	1.8945	1339.9762	18.092
434.8834	6.809	1464.0017	70.1101
492.7998	1.4617	1487.9403	15.9667
583.3636	0.6006	1692.3635	0.7617
681.134	62.6218	3143.2329	2.7806
720.2682	60.6331	3159.9709	1.1871
742.6833	10.1681	3177.2672	4.5582
768.0456	10.8727	3182.835	3.6474
784.138	7.6025	3249.5657	85.2601
795.1143	4.6298	3313.1376	48.4879
832.0699	35.8803		

Table S18. Cartesian coordinates for the optimized geometry of isomer 3d-quartet of $Pt^{+}(C_2H_2)_3$ followed by its predicted frequencies (cm^{-1}) and IR intensities (km/mol).

Z	x	y	z
6	-1.725110000	-0.653121000	-0.501784000
6	-2.150502000	-0.523158000	0.892753000
6	-2.290132000	0.890791000	0.728202000
6	-1.878815000	0.807361000	-0.650068000
1	-1.808006000	-1.471114000	-1.206461000
1	-2.336228000	-1.220826000	1.692638000
1	-2.607706000	1.696955000	1.371183000
1	-1.906926000	1.486321000	-1.486118000
78	0.348902000	-0.131222000	-0.127263000
6	2.131811000	-0.099909000	0.774639000
6	1.987145000	1.003602000	0.147176000
1	2.312582000	1.972409000	-0.185992000
1	2.685510000	-0.781853000	1.395744000

Frequency (cm^{-1})	Intensity (km/mol)	Frequency (cm^{-1})	Intensity (km/mol)
53.8981	0.8862	895.2122	8.8611
77.6964	3.041	930.6028	0.5621
97.764	4.22	949.2328	2.0794
145.1984	9.9782	1006.6085	3.6922
187.2905	5.6846	1203.7488	0.7173
310.4796	0.3422	1233.6065	2.4061
476.5799	13.0923	1255.1585	16.6688
504.6286	1.8376	1330.8994	21.7034
561.3635	1.912	1344.0224	12.212
668.9295	74.5602	1664.1731	0.8179
715.445	4.2671	3179.7419	14.2487
746.3048	35.761	3229.6301	7.0487
766.3693	0.6775	3247.9009	21.8813
801.1591	22.0978	3257.5531	4.6025
811.6414	5.4573	3260.2227	95.3247
830.0309	3.942	3315.8379	57.4153
877.0396	52.6504		

Table S19. Cartesian coordinates for the optimized geometry of isomer 3e-quartet of $Pt^+(C_2H_2)_3$ followed by its predicted frequencies (cm^{-1}) and IR intensities (km/mol).

Z	x	y	z
6	-1.681549000	-0.686299000	1.029719000
6	-2.208880000	0.342786000	0.000167000
6	-1.681740000	-0.686271000	-1.029516000
6	-1.217308000	-1.532632000	0.000048000
1	-1.755309000	-0.742956000	2.103575000
1	-3.289681000	0.472237000	0.000267000
1	-1.755682000	-0.742888000	-2.103361000
1	-0.826603000	-2.542771000	-0.000002000
78	0.759308000	-0.074704000	-0.000049000
6	-1.468423000	1.638168000	0.000108000
6	-0.088847000	1.633700000	0.000012000
1	0.495084000	2.548675000	-0.000031000
1	-2.013342000	2.577909000	0.000130000

Frequency (cm^{-1})	Intensity (km/mol)	Frequency (cm^{-1})	Intensity (km/mol)
58.7761	0.8307	986.5637	10.0742
136.1298	0.0592	1122.1517	4.3048
203.1403	1.788	1129.319	19.4364
225.5636	1.5323	1150.5035	11.9575
294.4469	4.2851	1206.309	1.3812
394.809	0.1363	1246.3422	22.1556
574.3134	1.6993	1299.8232	0.7158
584.2157	32.4267	1345.8147	1.6346
639.4579	30.3104	1381.9653	23.7965
701.2989	19.1774	1454.9106	13.9884
728.1198	16.9954	3103.3212	0.4997
838.5685	18.2218	3142.1932	6.5437
861.8627	3.1589	3159.7819	8.8346
898.463	1.86	3181.2134	1.2215
936.7448	1.4131	3239.54	22.4889
966.0176	10.5332	3246.1092	0.6073
974.2346	11.3363		

Table S20. Cartesian coordinates for the optimized geometry of isomer 3f-quartet of $Pt^+(C_2H_2)_3$ followed by its predicted frequencies (cm^{-1}) and IR intensities (km/mol).

Z	x	y	z
78	-0.000003000	-0.000028000	0.000000000
1	-2.511553000	1.602199000	0.000000000
6	-2.054883000	-0.629553000	0.000000000
6	-2.054885000	0.629641000	0.000000000
1	-2.511600000	-1.602091000	0.000000000
1	1.255770000	1.602210000	2.175062000
6	1.027451000	-0.629543000	1.779579000
6	1.027451000	0.629650000	1.779572000
1	1.255810000	-1.602078000	2.175114000
1	1.255770000	1.602210000	-2.175062000
6	1.027451000	0.629650000	-1.779572000
6	1.027451000	-0.629543000	-1.779579000
1	1.255810000	-1.602078000	-2.175114000

Frequency (cm^{-1})	Intensity (km/mol)	Frequency (cm^{-1})	Intensity (km/mol)
63.9457	14.5396	723.7929	0
85.2678	0	750.038	0
92.8105	7.6373	750.0421	0
92.8178	7.6373	793.791	0
172.5322	0	793.8144	0.0025
172.5439	0	796.6876	133.4264
246.6776	3.0639	849.3391	0
298.517	2.4914	1695.9486	8.8795
298.5241	2.4906	1695.9486	8.8832
386.4481	0	1788.4058	0
386.4483	0	3261.8913	283.0109
442.1908	0	3263.0514	0
666.1411	3.5466	3263.0574	0.003
666.1644	3.5599	3323.7263	84.1594
705.8998	0	3323.7319	84.1611
721.503	97.3028	3327.6194	0.0001
721.5067	97.2914		

Table S21. Cartesian coordinates for the optimized geometry of isomer 3g-quartet of $Pt^{+}(C_2H_2)_3$ followed by its predicted frequencies (cm^{-1}) and IR intensities (km/mol).

Z	x	y	z
78	-0.008860000	-0.103091000	-0.041890000
6	-1.308113000	-1.604717000	0.715769000
6	-1.649128000	-1.397266000	-0.460015000
1	-2.165933000	-1.463695000	-1.396551000
1	-1.276206000	-2.000196000	1.711413000
6	-0.390457000	1.882458000	0.575052000
6	-1.309998000	2.394818000	-0.100407000
1	-2.075181000	2.683844000	-0.787065000
1	0.209679000	2.150886000	1.431649000
6	2.908046000	-0.237767000	0.250696000
6	1.887407000	0.100896000	-0.545266000
1	2.091242000	0.366963000	-1.583794000
1	3.080974000	-0.527192000	1.276763000

Frequency (cm^{-1})	Intensity (km/mol)	Frequency (cm^{-1})	Intensity (km/mol)
55.4377	4.9235	770.036	53.5173
65.9027	6.7213	791.6639	16.7218
87.7499	3.7804	811.0601	6.3114
109.0765	3.4095	824.0562	48.5504
127.2559	0.5415	870.8411	29.9944
154.405	0.9661	906.5674	17.613
187.6327	1.6032	1144.8014	5.4024
238.4033	2.3785	1393.2817	171.0668
313.8854	21.767	1720.6258	16.7313
336.4504	0.6256	1834.1873	3.0748
428.5285	7.9625	3069.9121	5.1147
505.9655	113.6666	3213.1257	51.8292
557.1342	15.1318	3220.9342	9.3468
626.1787	0.5736	3302.451	143.7383
668.4148	2.3899	3377.2893	59.6877
721.2086	1.0674	3392.0918	156.8504
750.776	61.1381		

Table S22. Cartesian coordinates for the optimized geometry of isomer TS1-doublet $Pt^{+}(C_2H_2)_3$ followed by its predicted frequencies (cm^{-1}) and IR intensities (km/mol).

78	0.051749000	0.112099000	0.420223000
1	0.719832000	2.879803000	-0.071500000
6	-0.848966000	1.781198000	1.169488000
6	0.093400000	2.150900000	0.407977000
1	-1.682458000	1.945916000	1.826581000
1	0.318798000	-1.262757000	2.973620000
6	0.244687000	-2.117294000	0.905864000
6	0.355030000	-1.326341000	1.901503000
1	-0.106291000	-3.113592000	0.694986000
1	0.178350000	-0.212961000	-2.471052000
6	0.262973000	-0.670962000	-1.502617000
6	0.781561000	-1.642441000	-0.857343000
1	1.538045000	-2.396880000	-0.994525000

Frequency (cm^{-1})	Intensity (km/mol)	Frequency (cm^{-1})	Intensity (km/mol)
-384.3952	0.0508	777.3434	0.0937
68.2374	7.4589	830.3362	0.0417
120.7794	3.3827	853.2452	18.8868
123.8538	12.5942	857.6543	4.3343
204.3707	1.167	889.0529	40.5058
219.2995	5.8793	985.6435	3.7339
336.6045	0.8683	1070.8357	21.6535
338.5814	7.0327	1607.5806	2.5181
467.1794	4.3138	1716.3335	0.004
468.4097	3.9751	1739.2826	2.3331
539.2956	8.1545	3233.0799	27.4831
546.9151	0.2391	3240.1143	94.627
719.1134	76.6057	3272.5964	87.8284
724.4621	16.4327	3298.9527	142.5077
742.5494	38.8884	3300.766	15.4924
762.6455	64.1275	3331.23	51.2456
764.4839	2.1847		

Table S23. Cartesian coordinates for the optimized geometry of isomer TS2-doublet $Pt^+(C_2H_2)_3$ followed by its predicted frequencies (cm^{-1}) and IR intensities (km/mol).

Z	x	y	z
78	0.409568000	-1.299549000	0.784220000
6	2.275866000	-1.809285000	0.274668000
6	2.956604000	-0.805778000	-0.307659000
1	4.003574000	-0.903156000	-0.567715000
1	2.621205000	-2.804844000	0.526471000
6	2.194515000	0.373643000	-0.605159000
6	0.900865000	0.373697000	-0.191801000
1	0.103090000	1.058022000	-0.444724000
1	2.599404000	1.206858000	-1.168053000
6	0.579843000	-0.119639000	2.515010000
6	0.770389000	1.068579000	2.183933000
1	0.907329000	2.122398000	2.076547000
1	0.472264000	-0.714613000	3.411123000

Frequency (cm^{-1})	Intensity (km/mol)	Frequency (cm^{-1})	Intensity (km/mol)
-239.4126	15.3047	876.6563	18.24
95.6783	0.4423	943.1607	1.4546
128.1527	0.5608	1006.3173	2.2557
230.2892	1.2056	1094.4047	10.8176
273.2907	0.9589	1096.8941	9.3397
357.2789	0.718	1227.477	75.4739
370.2362	0.5657	1284.78	17.0185
451.8089	10.5415	1415.9222	40.8645
497.7938	2.0402	1559.5167	3.8191
589.2173	13.1453	1774.3479	1.8115
651.7406	4.5619	3162.614	1.2166
666.0655	91.7811	3170.7199	11.5654
686.4273	3.1842	3184.702	17.9484
720.2688	89.3392	3200.4396	15.3741
757.8661	9.8463	3208.6655	73.8368
799.2715	2.3677	3391.5708	137.7322
869.5847	8.883		

Table S24. $Pt^+(C_2H_2)_4$ calculated at the

Isomer	2s + 1	E (hartree)	Relative E (kcal/mol)
4a	2	-428.859319	0
4a	4	-428.755964	64.85625554
4b	2	-428.824796	21.6635142
4b	4	-428.760246	62.1692594
4c	2	-428.785234	46.48904931
4c	4	-428.710161	93.59807811
4d	2	-428.779066	50.35952857
4d	4	-428.730217	81.01274542
4e	2	-428.754274	65.91674678
4e	4	-428.71543	90.29172999
4f	2	-428.75206	67.30605305
4f	4	-428.677436	114.13333
4g	2	-428.743445	72.71204832
4g	4	-428.671989	117.5513749
4h	2	-428.733381	79.02730502
4h	4	-428.650154	131.2530472
4i	2	-428.73336	79.04048272
4i	4	-428.714972	90.57912939
4j	2	-428.720552	87.07762578
4j	4	-428.668615	119.6685923
4k	2	-428.708887	94.39752536
4k	4	-428.646945	133.2667255
4l	2	-428.656995	126.9602539
4l	4	-428.575379	178.1750781
4m	2	-428.656262	127.4202185
4n	2	-428.647886	132.676239
4o	5	-428.643971	135.1329391

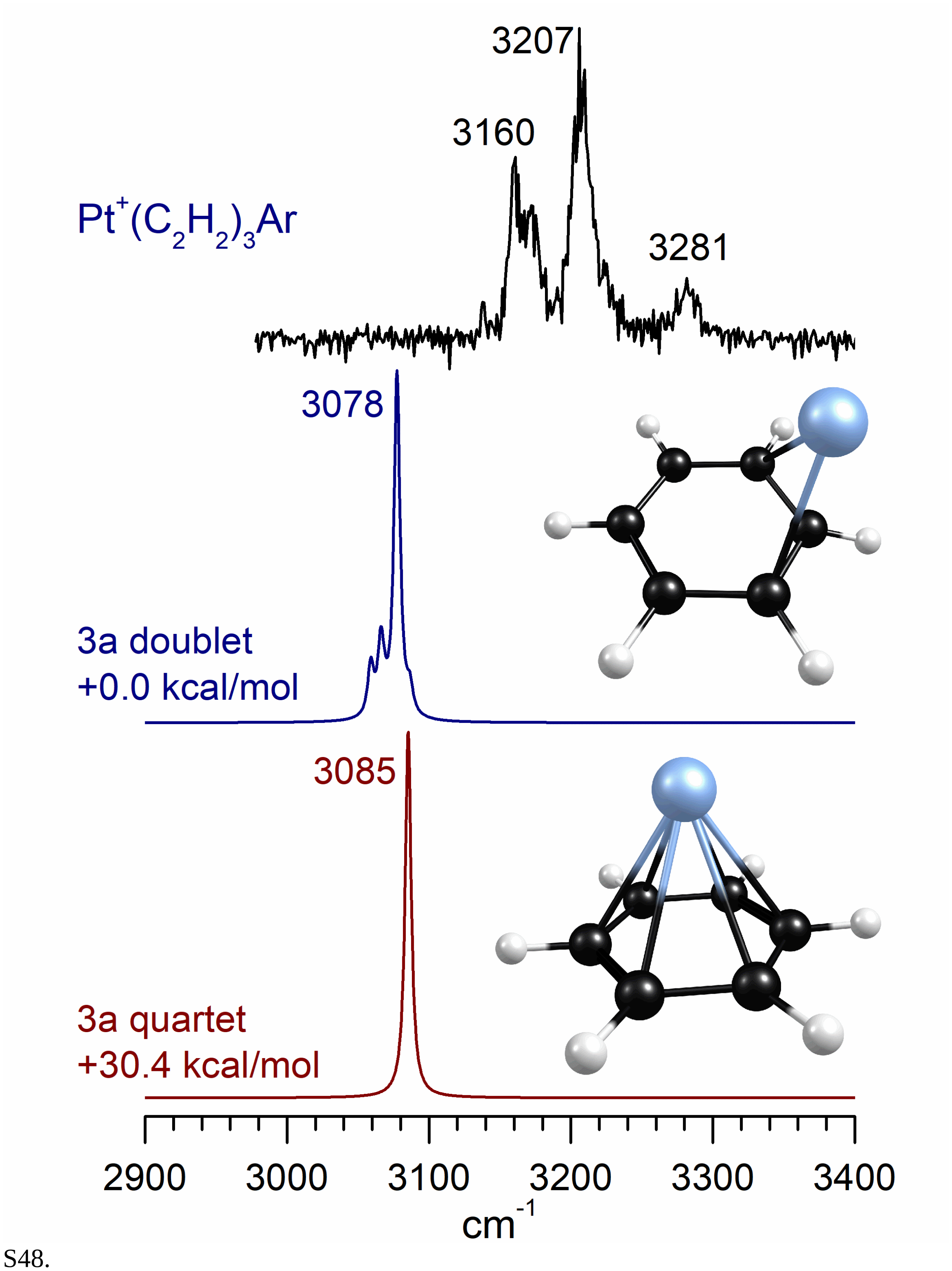
3207
3160
$Pt^+(C_2H_2)_3Ar$
3281
3078
3a doublet
+0.0 kcal/mol
3085
3a quartet
+30.4 kcal/mol
2900
3000
3100
3200
3300
3400
cm^{-1}

S48.

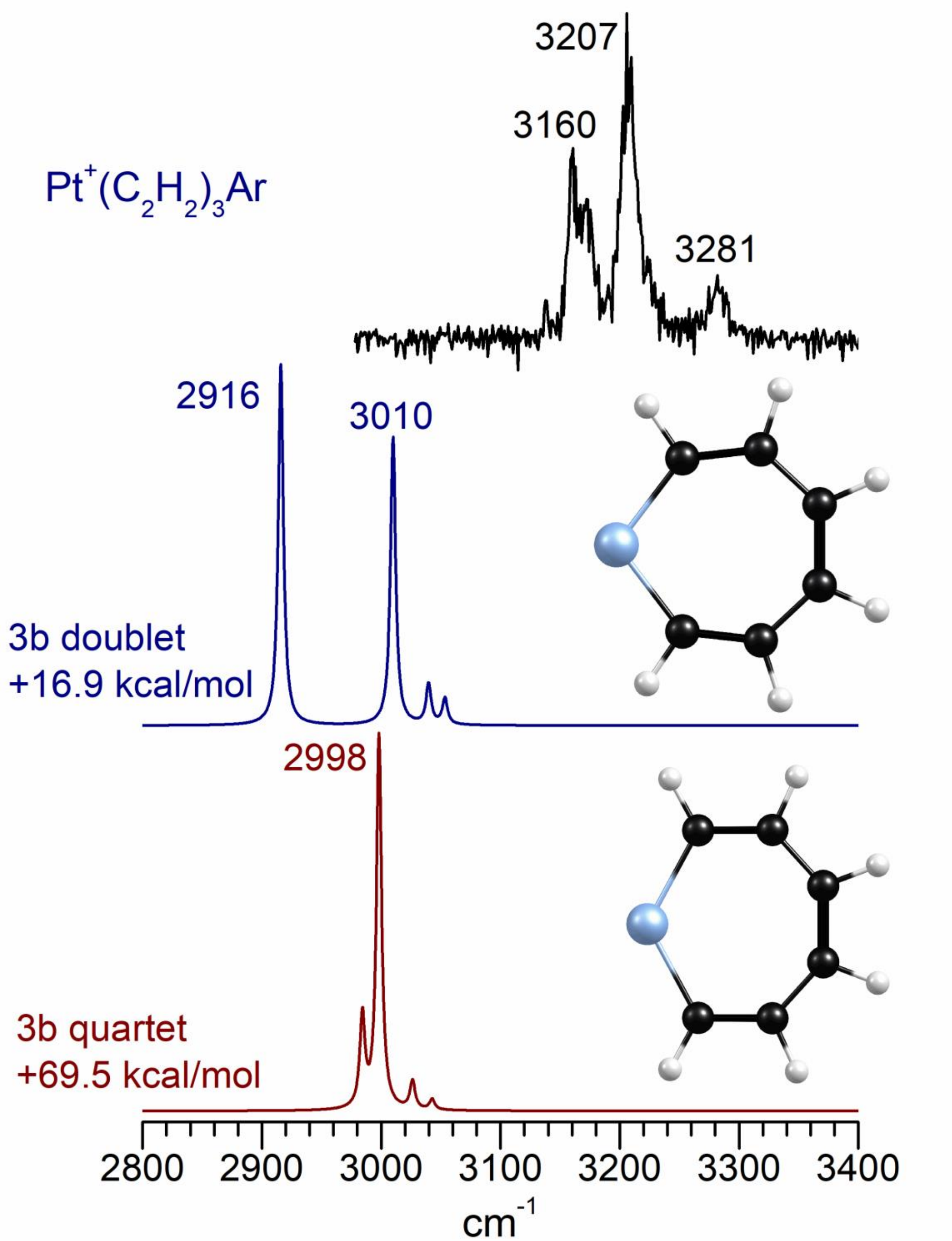
3207
3160
Pt+(C2H2)3Ar
3281
2916
3010
3b doublet
+16.9 kcal/mol
2998
3b quartet
+69.5 kcal/mol
2800
2900
3000
3100
3200
3300
3400
cm-1

S49.

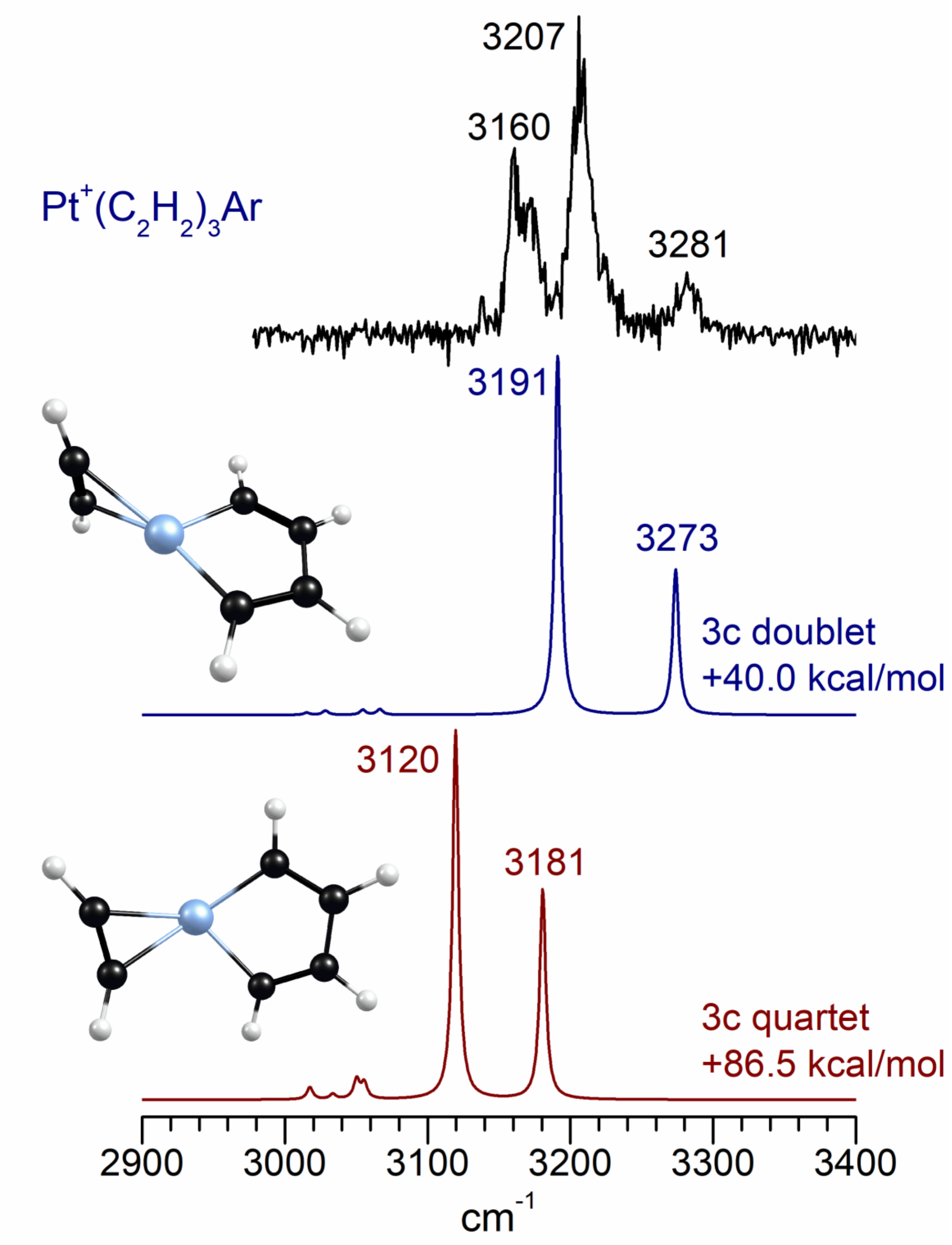
3207
3160
Pt$^+$(C$_2$H$_2$)$_3$Ar
3281
3191
3273
3c doublet
+40.0 kcal/mol
3120
3181
3c quartet
+86.5 kcal/mol
2900
3000
3100
3200
3300
3400
cm^{-1}

S50.

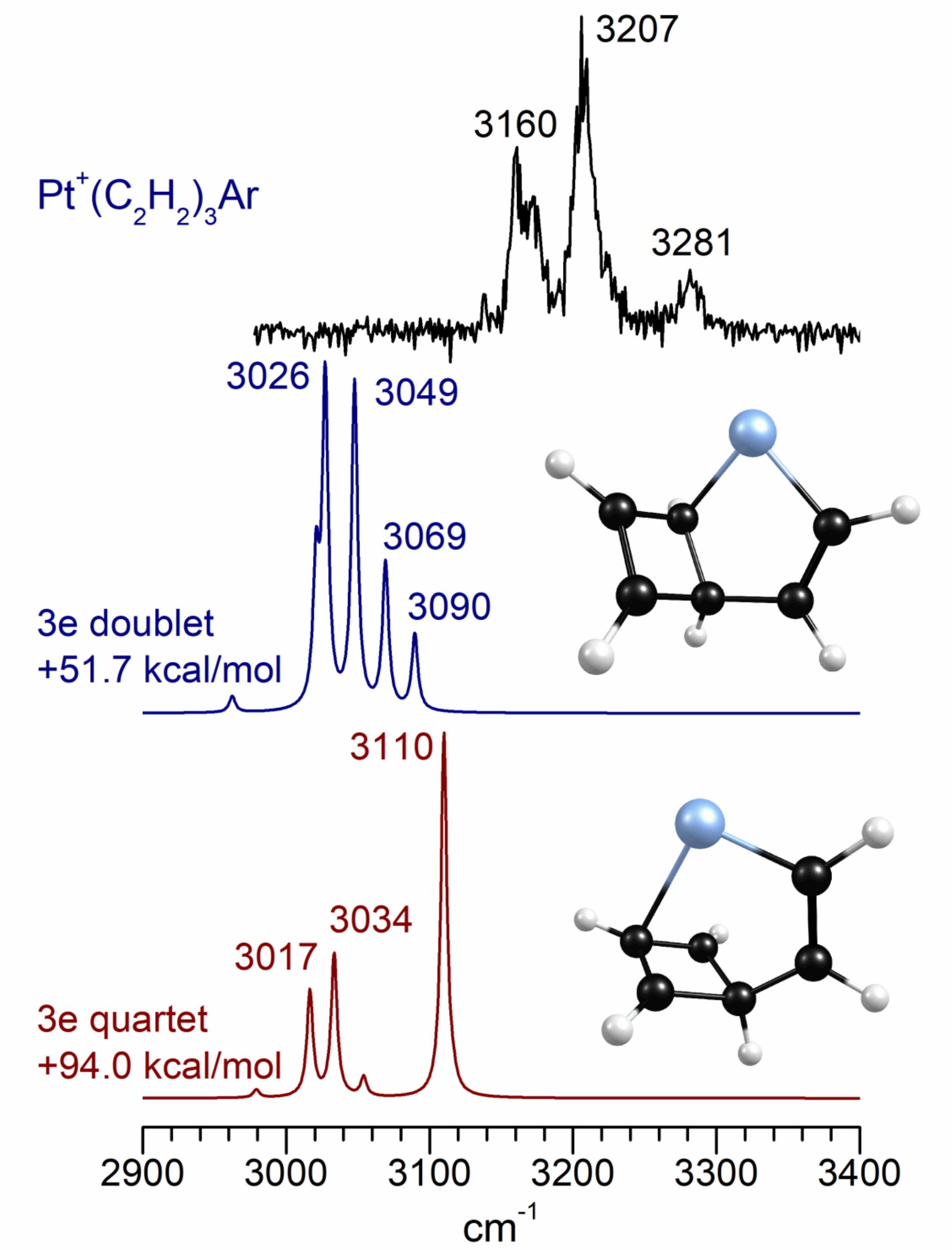
3207
3160
Pt$^+$(C$_2$H$_2$)$_3$Ar
3281
3026
3049
3069
3090
3e doublet
+51.7 kcal/mol
3110
3034
3017
3e quartet
+94.0 kcal/mol
2900
3000
3100
3200
3300
3400
cm^{-1}

S51.

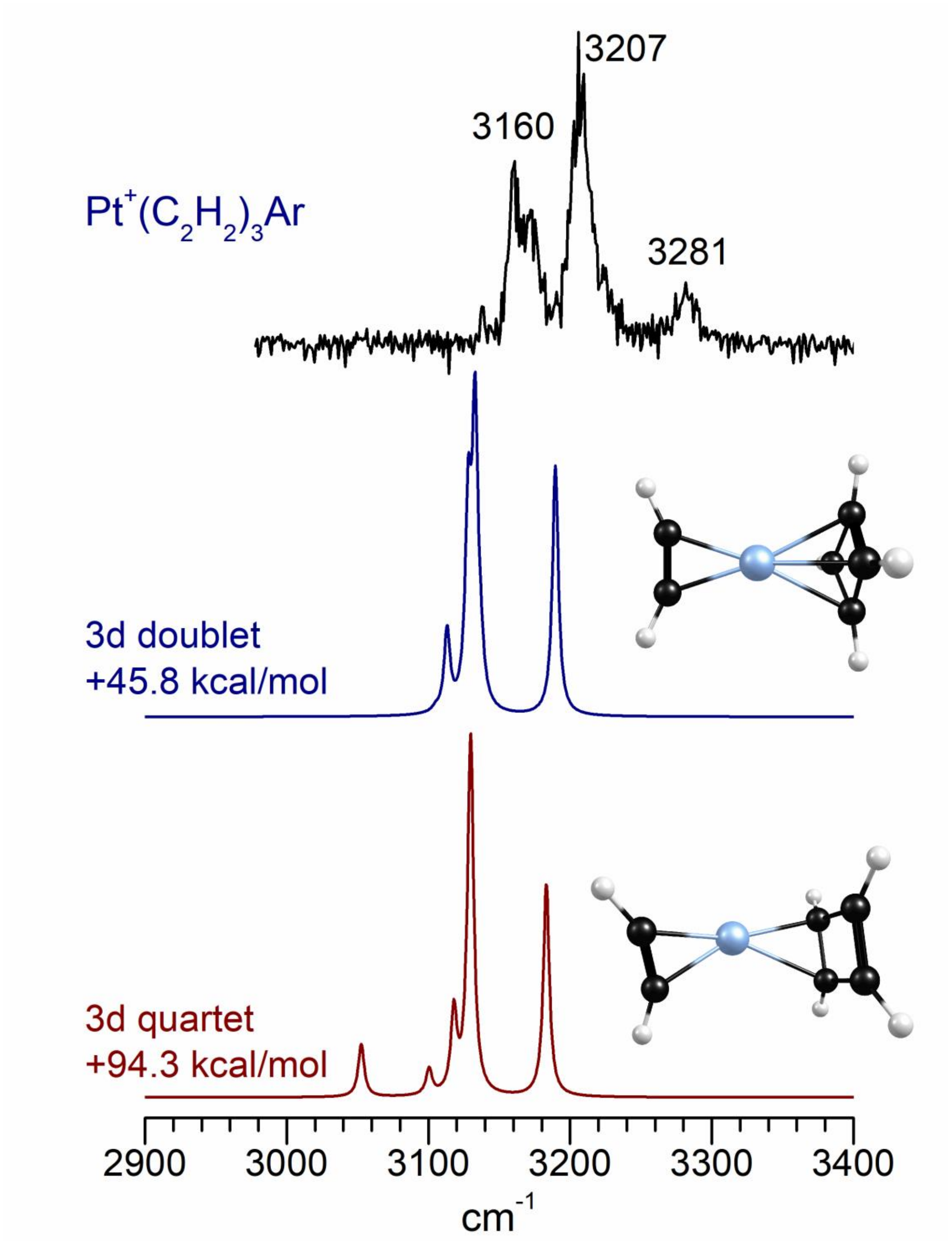

3207
3160
Pt+(C2H2)3Ar
3281
3d doublet
+45.8 kcal/mol
3d quartet
+94.3 kcal/mol
2900
3000
3100
3200
3300
3400
cm-1

S52.

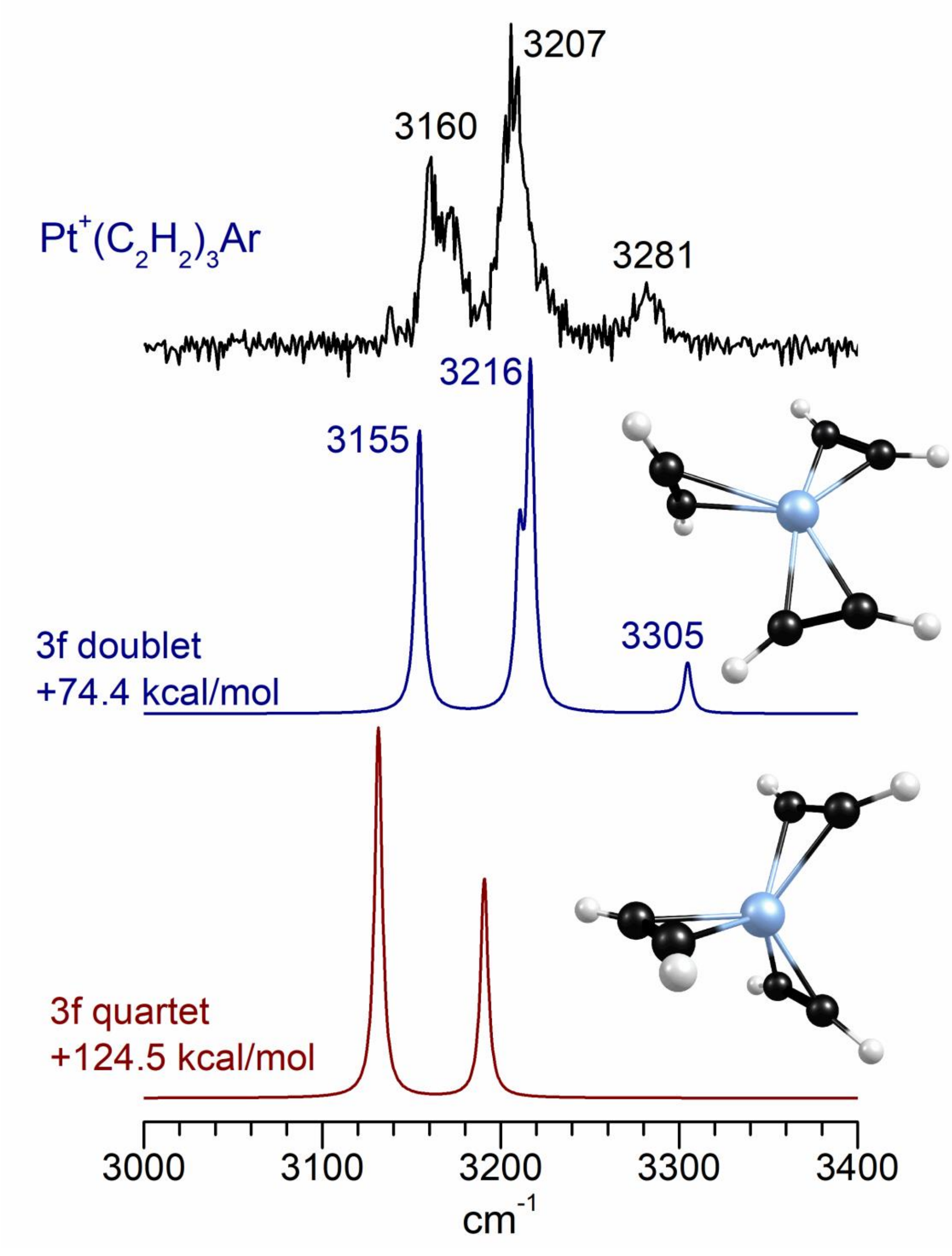
3207
3160
$Pt^+(C_2H_2)_3Ar$
3281
3216
3155
3f doublet
+74.4 kcal/mol
3305
3f quartet
+124.5 kcal/mol
3000
3100
3200
3300
3400
cm^{-1}

S53.

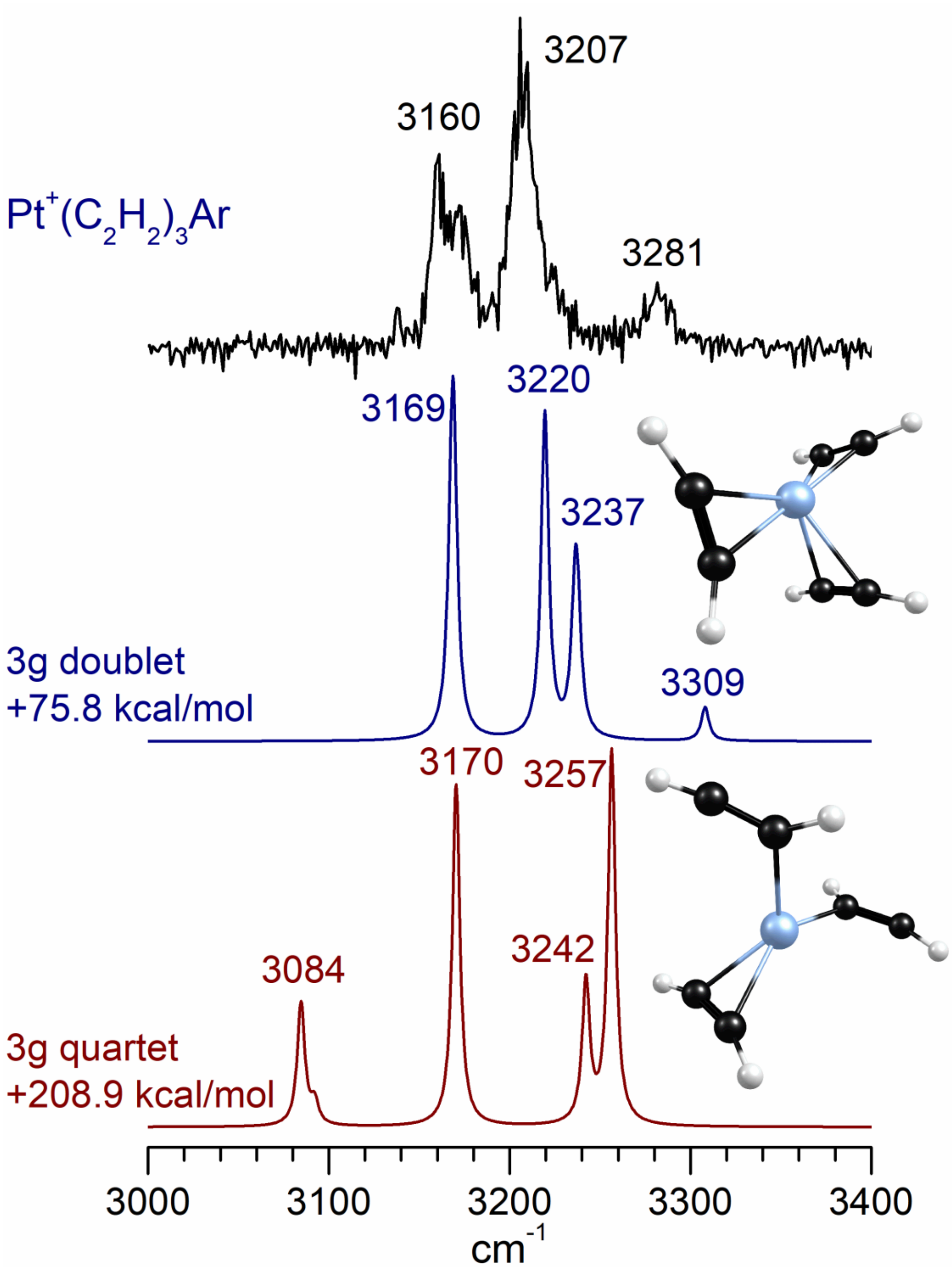
3207
3160
Pt+(C2H2)3Ar
3281
3169
3220
3237
3g doublet
+75.8 kcal/mol
3309
3170
3257
3242
3084
3g quartet
+208.9 kcal/mol
3000
3100
3200
3300
3400
cm-1

S54.

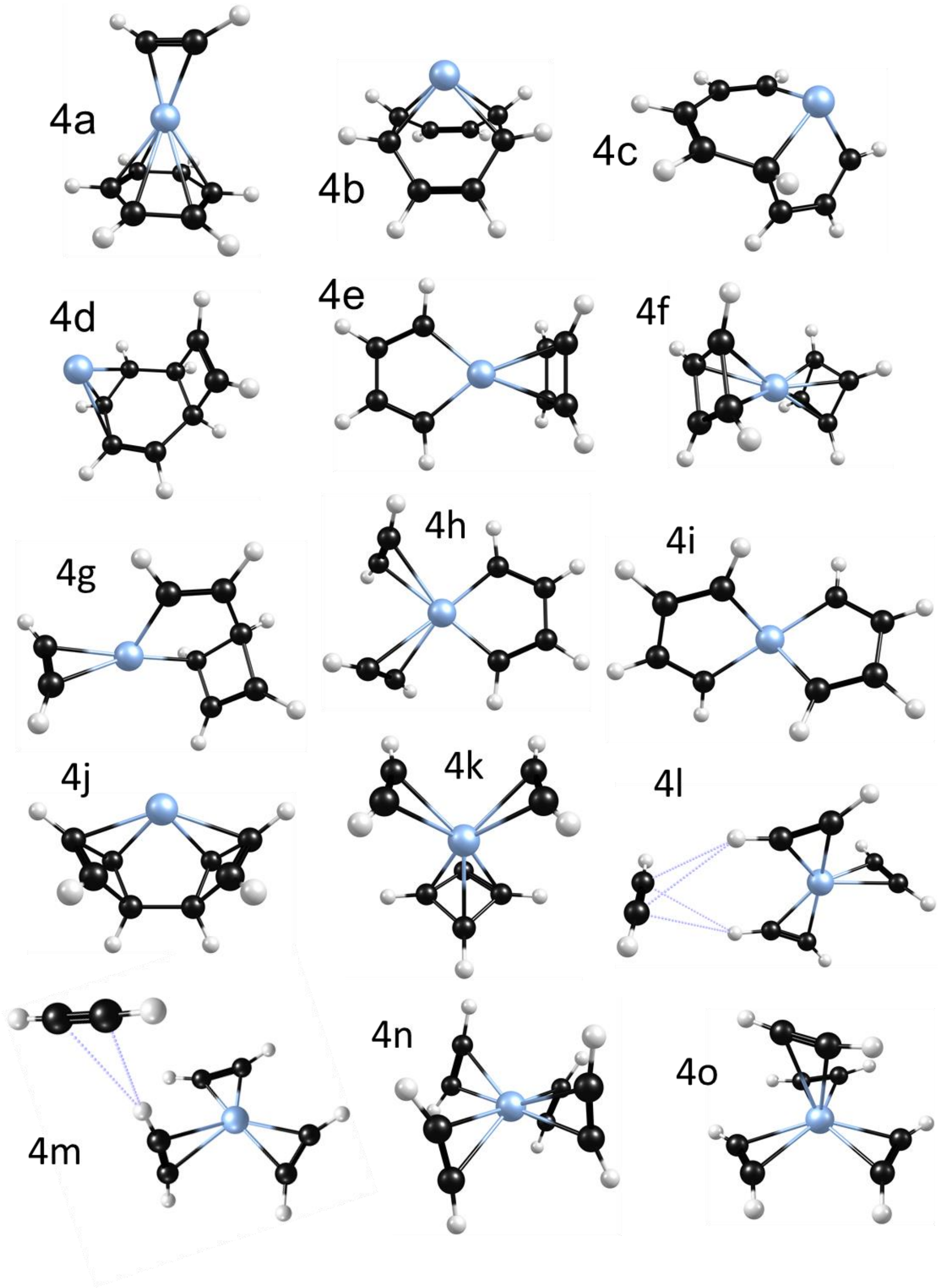

S55.

Table S25. Cartesian coordinates for the optimized geometry of isomer 4a-doublet of $Pt^{+}(C_2H_2)_4$ followed by its predicted frequencies (cm^{-1}) and IR intensities (km/mol).

Z	x	y	z
6	1.621985000	-1.221020000	0.701172000
6	1.609058000	0.000573000	1.401592000
6	1.622008000	-1.221589000	-0.700186000
1	1.581650000	0.001006000	2.481417000
1	1.611984000	-2.155067000	-1.243269000
6	1.621975000	1.221597000	0.700191000
6	1.609096000	-0.000566000	-1.401587000
1	1.611927000	2.155075000	1.243274000
1	1.581714000	-0.000998000	-2.481413000
6	1.621988000	1.221027000	-0.701168000
1	1.611942000	2.154064000	-1.245009000
1	1.611937000	-2.154057000	1.245012000
78	-0.438530000	-0.000002000	0.000002000
1	-2.852660000	1.578161000	-0.000019000
6	-2.328097000	0.640329000	-0.000015000
1	-2.852667000	-1.578158000	-0.000028000
6	-2.328099000	-0.640329000	-0.000019000

Frequency	Intensity	Frequency	Intensity	Frequency	Intensity
19.3357	0	781.5926	68.5881	1336.0278	0.0243
65.3216	4.9174	857.4178	3.7059	1386.5723	0
115.3887	5.4899	913.0023	26.752	1495.9421	27.693
120.3391	1.1936	926.9954	0.096	1503.3617	24.9221
132.6008	8.1342	931.4381	20.3636	1575.8238	0
180.1191	0.3341	994.8026	2.3298	1584.7838	1.5803
383.0389	0.3755	1014.3591	0.2216	1671.6188	0.5882
400.2352	0	1017.1593	0	3200.7721	0.0841
524.8049	1.8218	1024.6416	0.0204	3206.5017	0.0029
577.8423	0.2696	1037.8616	0.7077	3207.1862	0
609.0201	0.1373	1041.4565	0.1687	3215.5831	9.003
609.1083	0.0003	1050.1309	1.3134	3216.6602	8.5983
662.4034	0.1173	1190.6924	0.0019	3222.2613	0.0158
758.9857	42.246	1194.8938	0	3265.6247	59.1911
779.5228	0	1195.3187	0.5595	3317.636	37.6715

Table S26. Cartesian coordinates for the optimized geometry of isomer 4b-doublet of $Pt^+(C_2H_2)_4$ followed by its predicted frequencies (cm^{-1}) and IR intensities (km/mol).

Z	x	y	z
6	-1.603614000	1.536137000	-0.667852000
6	-1.603620000	1.536186000	0.667737000
6	-0.677581000	0.706637000	1.461702000
6	-1.603616000	-1.536186000	-0.667744000
6	-1.603622000	-1.536135000	0.667844000
6	-0.677576000	-0.706531000	1.461746000
1	-2.238243000	-2.220878000	1.217548000
1	-2.238224000	2.220890000	-1.217558000
1	-2.238234000	2.220979000	1.217386000
1	-0.193219000	1.192240000	2.303928000
1	-2.238235000	-2.220970000	-1.217401000
1	-0.193212000	-1.192076000	2.304004000
78	0.826594000	0.000000000	0.000002000
1	-0.193200000	1.192074000	-2.304008000
6	-0.677567000	0.706531000	-1.461749000
6	-0.677568000	-0.706639000	-1.461701000
1	-0.193202000	-1.192241000	-2.303926000

Frequency	Intensity	Frequency	Intensity	Frequency	Intensity
202.7647	1.0118	843.2634	1.055	1402.1826	25.4407
205.3198	2.632	894.5223	0	1415.9515	1.2002
224.5519	11.9815	933.7872	5.6374	1446.395	0
242.9352	0	944.8223	0.3	1463.1817	0.2971
274.5444	1.2686	973.1292	0.0114	1470.6046	10.4271
317.9542	0	983.4147	0	1664.2315	2.4375
325.5172	38.6846	1001.0477	3.1259	1673.212	14.0497
440.3334	3.6645	1019.6732	26.7772	3126.0336	0.0038
446.3258	0.009	1022.8775	0	3126.1278	5.0144
612.1298	12.709	1039.2908	0	3139.4026	0.5305
649.7628	0	1183.3478	1.4448	3141.0659	0.3707
725.1172	2.3118	1204.8945	0.0525	3163.7597	0
737.9419	14.1606	1206.2768	0.8435	3164.3936	3.0573
804.0814	60.222	1212.3685	5.6945	3179.7906	4.0155
822.4117	27.2089	1366.6699	0	3180.24	2.6521

Table S27. Cartesian coordinates for the optimized geometry of isomer 4c-doublet of $Pt^+(C_2H_2)_4$ followed by its predicted frequencies (cm^{-1}) and IR intensities (km/mol).

Z	x	y	z
6	0.787621000	-1.077132000	-1.158218000
6	2.078588000	-0.754029000	-1.035022000
6	2.687296000	0.121395000	-0.036798000
6	0.070767000	2.256955000	-0.263229000
6	0.576803000	1.254046000	0.710112000
6	2.028324000	0.976630000	0.760896000
1	0.161702000	1.462418000	1.704697000
1	0.368978000	-1.631222000	-1.989878000
1	2.762231000	-1.215198000	-1.744333000
1	3.761030000	0.043122000	0.071868000
1	0.661916000	3.140815000	-0.463460000
1	2.580936000	1.491918000	1.539880000
78	-0.494897000	-0.505846000	0.241272000
1	-2.561412000	0.322628000	-1.127166000
6	-1.655304000	0.729194000	-0.691341000
6	-1.149036000	2.008380000	-0.849456000
1	-1.683797000	2.748861000	-1.432451000

Frequency	Intensity	Frequency	Intensity	Frequency	Intensity
57.1632	0.8962	783.018	7.1822	1417.5229	16.5067
125.3663	0.1685	853.1573	13.4308	1425.0205	5.7036
176.2892	2.3056	914.2271	4.9821	1463.3765	9.2102
273.8589	2.4784	936.3501	11.2758	1580.9249	6.9356
299.2153	1.457	943.535	13.2161	1649.9436	6.1499
336.8089	2.489	988.8803	11.3638	2993.4066	2.8302
373.0003	0.9304	1016.1887	7.4562	3120.2435	8.1046
437.0726	6.6558	1022.694	18.3252	3145.4936	5.1577
498.1398	14.9509	1059.0343	1.0583	3159.3913	7.5477
525.2437	12.9687	1080.6434	4.5289	3170.1798	14.7487
590.1745	27.9122	1139.5015	5.056	3174.435	9.7507
614.2743	16.1999	1152.5054	16.965	3187.3313	0.1495
637.3132	15.7219	1231.7287	5.9478	3192.897	7.9676
689.2213	71.0931	1244.3014	23.7886		
719.7207	5.2995	1298.5561	51.2909		
771.3893	18.0406	1313.0703	14.5764		

Table S28. Cartesian coordinates for the optimized geometry of isomer 4d-doublet of $Pt^+(C_2H_2)_4$ followed by its predicted frequencies (cm^{-1}) and IR intensities (km/mol).

Z	x	y	z
78	-0.953360000	-0.232914000	-0.154709000
6	2.469635000	-0.861519000	-0.879815000
1	2.845370000	-1.141283000	-1.853176000
6	2.007448000	-1.538245000	0.172853000
1	1.869621000	-2.588731000	0.385722000
6	1.448541000	1.507756000	-0.782011000
6	0.273719000	1.893103000	-0.184647000
6	2.361348000	0.502741000	-0.191342000
1	-0.314678000	2.691828000	-0.615799000
1	3.339641000	0.964196000	-0.018627000
6	-0.212063000	1.273124000	1.037234000
6	1.806498000	-0.297497000	1.037429000
1	-0.958335000	1.771199000	1.641754000
1	2.453327000	-0.257388000	1.918756000
6	0.404405000	0.049095000	1.451693000
1	0.034942000	-0.415216000	2.359185000
1	1.735005000	1.971361000	-1.718856000

Frequency	Intensity	Frequency	Intensity	Frequency	Intensity
77.8716	0.9846	928.7387	5.5376	1309.6881	6.9872
84.2702	0.2979	946.4158	10.6641	1319.6889	6.3224
183.3363	0.5016	960.3056	1.9453	1385.9592	7.8305
283.6402	1.5395	978.3665	2.0875	1430.9229	19.2897
352.9304	2.8224	983.8205	4.6019	1449.8287	3.8581
417.0296	1.4942	1000.1337	1.698	1545.1152	46.4478
464.7013	8.0011	1013.9798	0.095	1615.5161	1.2811
524.4432	5.0573	1032.8986	17.2288	3015.1607	2.5068
563.9191	0.9963	1066.9844	2.8073	3032.325	2.2365
578.1118	4.6414	1094.5208	17.9051	3150.9093	1.5163
753.8462	55.4404	1144.2577	0.4166	3168.0799	1.2484
766.5744	21.5924	1166.0916	4.2314	3182.2624	2.5295
822.1494	25.2645	1197.4609	6.1012	3194.452	2.9633
833.8385	6.8234	1213.1352	2.7606	3194.698	0.2596
874.1896	9.277	1282.1929	19.2128	3223.1454	1.1037

Table S29. Cartesian coordinates for the optimized geometry of isomer 4e-doublet of $Pt^{+}(C_2H_2)_4$ followed by its predicted frequencies (cm^{-1}) and IR intensities (km/mol).

Z	x	y	z
6	-2.759390000	0.710085000	-0.000290000
6	-2.759350000	-0.710174000	-0.000298000
6	-1.504965000	1.297198000	0.000219000
1	-3.680686000	-1.281196000	-0.000592000
1	-1.419964000	2.378398000	0.000581000
6	-1.504890000	-1.297209000	0.000217000
1	-1.419814000	-2.378403000	0.000576000
1	-3.680760000	1.281053000	-0.000566000
78	0.050053000	0.000024000	0.000081000
6	2.039599000	-0.760453000	0.694202000
6	2.039694000	0.760431000	0.694137000
1	2.130016000	1.518268000	1.454637000
1	2.129850000	-1.518231000	1.454770000
1	2.129486000	-1.518352000	-1.455048000
6	2.039443000	-0.760507000	-0.694522000
1	2.129647000	1.518139000	-1.455172000
6	2.039542000	0.760365000	-0.694582000

Frequency	Intensity	Frequency	Intensity	Frequency	Intensity
56.8654	5.4835	829.093	0.5011	1378.3674	5.5158
77.063	0.8697	831.7541	9.8871	1408.4926	1.3368
89.0415	0	899.2594	0	1478.2082	14.2745
113.4167	6.6822	922.9801	2.3917	1481.6359	58.3502
298.1394	5.6073	944.0031	0	1511.6642	3.7434
310.4417	10.2471	961.1842	2.2874	3151.6158	0.3362
345.3665	0	973.745	1.2595	3157.5994	0.2998
358.4563	9.3702	1011.5008	0	3171.8472	0.6036
360.1408	5.4141	1031.181	3.5762	3181.9686	0.1658
502.8085	0.2288	1040.1867	5.1788	3232.9436	0
556.7068	0	1123.3326	7.2023	3246.25	18.0354
632.3437	4.3564	1136.1138	0.0852	3256.4	12.3931
676.3759	51.1718	1149.8778	3.1361	3264.9316	8.1976
790.2519	0.8513	1202.0779	0		
790.734	0.0019	1293.1077	32.7433		
801.1077	66.3225	1301.1712	4.9562		

Table S30. Cartesian coordinates for the optimized geometry of isomer 4f-doublet of $Pt^{+}(C_2H_2)_4$ followed by its predicted frequencies (cm^{-1}) and IR intensities (km/mol).

Z	x	y	z
6	-1.935933000	-0.750518000	0.708007000
6	-1.935961000	0.750312000	0.708213000
6	-1.935939000	0.750509000	-0.708014000
6	-1.935953000	-0.750315000	-0.708219000
1	-2.048282000	-1.507670000	1.465200000
1	-2.048312000	1.507262000	1.465610000
1	-2.048287000	1.507663000	-1.465207000
1	-2.048311000	-1.507260000	-1.465620000
78	-0.002601000	0.000002000	0.000003000
1	2.026987000	-2.105843000	-0.000343000
6	1.894739000	-1.038527000	-0.000166000
6	1.984329000	-0.000179000	1.019569000
1	2.127991000	-0.000360000	2.088103000
6	1.894751000	1.038518000	0.000155000
1	2.027007000	2.105834000	0.000339000
6	1.984322000	0.000174000	-1.019578000
1	2.127969000	0.000352000	-2.088115000

Frequency	Intensity	Frequency	Intensity	Frequency	Intensity
72.3065	0	818.3353	1.1017	1200.1636	0
101.1983	6.0516	838.8049	0.1218	1241.7593	1.1294
138.753	11.1243	854.5698	7.3069	1299.6014	24.5059
246.5716	0	887.0771	0.7271	1317.9307	18.0316
296.4729	17.4495	891.663	0.9943	1325.9425	1.6494
346.877	11.4245	894.7748	0	1358.818	11.3645
355.1777	3.0885	929.7915	5.2091	1414.9019	2.9261
366.4068	8.0553	935.0691	4.8627	3236.5595	0.6992
367.9656	3.9911	939.1784	0	3240.0988	0
390.4719	0.0409	952.0982	10.7432	3243.1854	14.3335
547.4434	22.8763	978.3163	2.3706	3254.4319	15.2173
757.2484	0	999.5173	9.7105	3260.3817	13.4377
789.3702	0.3138	1104.2036	0	3267.9357	17.9332
807.0157	27.2311	1152.1677	0.0182	3270.5847	15.5748
815.8501	63.2635	1199.118	0	3273.4992	6.8875

Table S31. Cartesian coordinates for the optimized geometry of isomer 4g-doublet of $Pt^{+}(C_2H_2)_4$ followed by its predicted frequencies (cm^{-1}) and IR intensities (km/mol).

Z	x	y	z
6	1.471430000	-0.594626000	-0.857640000
6	2.478429000	0.409299000	-0.223129000
6	2.605254000	-0.664835000	0.854437000
6	1.700811000	-1.510947000	0.221681000
1	1.327225000	-0.801117000	-1.913955000
1	3.396312000	0.592467000	-0.780543000
1	3.203432000	-0.742840000	1.751290000
1	1.365677000	-2.517325000	0.428554000
78	-0.504722000	-0.087776000	-0.124325000
6	1.766324000	1.652295000	0.193552000
1	2.307788000	2.567655000	0.407770000
6	0.437342000	1.607026000	0.268629000
1	-0.212589000	2.430198000	0.536026000
6	-2.573763000	0.236843000	-0.104967000
6	-2.291132000	-0.256140000	1.004542000
1	-2.405858000	-0.609373000	2.009314000
1	-3.181882000	0.653373000	-0.883727000

Frequency	Intensity	Frequency	Intensity	Frequency	Intensity
67.1861	2.2581	763.3978	18.4805	1314.2051	1.3414
67.9633	1.6502	812.1395	14.8137	1348.583	15.063
114.4356	4.9592	814.199	10.5466	1406.8706	27.537
147.2585	1.9414	849.5143	15.3768	1596.0014	1.8012
216.5386	1.088	879.8263	4.9843	1811.1446	2.9573
278.3769	2.4991	889.0868	13.8801	3090.1561	0.4341
291.7959	2.6838	918.3994	8.5224	3138.5075	10.383
356.4323	9.2177	932.4715	9.2865	3152.5046	2.298
436.6294	3.1238	948.6842	1.8456	3185.9801	3.0035
441.1884	6.9554	1019.9601	2.1122	3201.2761	3.273
476.5843	6.1007	1020.7541	7.0858	3222.2139	1.0334
573.5521	5.8313	1116.7093	7.5737	3299.486	116.4288
684.0499	33.7506	1150.3681	11.1638	3370.2081	68.9181
719.3421	0.3982	1170.3451	7.3139		
726.7559	34.003	1209.7264	0.1894		
752.3363	72.9083	1282.5519	23.9781		

Table S32. Cartesian coordinates for the optimized geometry of isomer 4h-doublet of $Pt^+(C_2H_2)_4$ followed by its predicted frequencies (cm^{-1}) and IR intensities (km/mol).

Z	x	y	z
78	-0.124552000	0.000006000	-0.000006000
6	1.395139000	1.265218000	-0.116777000
6	2.661965000	0.699767000	-0.080586000
1	3.570682000	1.283509000	-0.160897000
1	1.282877000	2.341777000	-0.201162000
6	2.661942000	-0.699833000	0.080606000
6	1.395095000	-1.265244000	0.116754000
1	1.282791000	-2.341800000	0.201120000
1	3.570638000	-1.283603000	0.160942000
6	-1.472100000	-1.904354000	-0.439235000
6	-1.971470000	-1.420579000	0.554164000
1	-2.532645000	-1.107620000	1.405593000
1	-1.144436000	-2.481470000	-1.274795000
1	-1.144206000	2.481646000	1.274636000
6	-1.471996000	1.904427000	0.439196000
6	-1.971538000	1.420532000	-0.554058000
1	-2.532861000	1.107481000	-1.405355000

Frequency	Intensity	Frequency	Intensity	Frequency	Intensity
39.5173	1.5584	676.5598	1.0184	1384.7016	7.114
74.5953	0.0657	681.7316	30.2274	1472.8109	50.3014
96.9159	6.4408	723.4323	3.0787	1506.6257	8.7329
103.7853	0.2042	736.2753	53.7738	1980.9405	0.5973
158.3732	2.98	769.8819	75.436	1982.0639	1.4197
161.4989	2.2665	776.2737	34.7277	3140.3371	4.2455
195.6728	0.0916	808.2529	30.5651	3144.6692	0.0962
227.8119	1.8899	815.8154	22.222	3177.7475	0.2006
246.2583	0.4528	823.5905	2.7588	3190.4666	0.0593
275.0253	0.5811	835.3879	3.3284	3357.8647	49.2389
286.6833	10.8878	974.4981	1.8462	3358.3309	253.1882
386.2285	1.6004	999.7451	0.0695	3449.9737	28.3766
392.9789	7.9791	1064.1727	0.3218	3450.8787	44.6194
516.1335	0.211	1120.9993	2.4047		
671.2617	3.6171	1139.7987	0.0456		
672.9138	2.357	1301.3602	8.9252		

Table S33. Cartesian coordinates for the optimized geometry of isomer 4i-doublet of $Pt^+(C_2H_2)_4$ followed by its predicted frequencies (cm^{-1}) and IR intensities (km/mol).

Z	x	y	z
78	0.000001000	-0.000003000	0.302936000
6	1.777677000	-1.120765000	0.288816000
6	2.807428000	-0.423455000	-0.271889000
1	3.832079000	-0.768472000	-0.327557000
1	1.945143000	-2.102128000	0.721142000
6	2.425296000	0.841577000	-0.826233000
6	1.104033000	1.186240000	-0.789333000
1	0.618937000	1.977988000	-1.343730000
1	3.141810000	1.525387000	-1.272541000
1	-0.618949000	-1.977980000	-1.343741000
6	-1.104041000	-1.186234000	-0.789337000
6	-2.425301000	-0.841562000	-0.826239000
1	-3.141818000	-1.525364000	-1.272556000
6	-2.807425000	0.423467000	-0.271885000
1	-3.832075000	0.768492000	-0.327555000
6	-1.777674000	1.120761000	0.288830000
1	-1.945135000	2.102123000	0.721162000

Frequency	Intensity	Frequency	Intensity	Frequency	Intensity
52.7864	0.9743	744.5415	4.7633	1256.7584	12.4658
103.151	4.5302	754.633	206.3545	1276.6852	214.6012
106.1024	0.9782	794.7814	262.3736	1363.1178	1.2142
125.0737	40.1072	806.8169	0.4223	1415.2456	341.3371
274.0122	1.2862	909.6017	396.4687	1484.7443	7.687
330.4738	300.3543	925.8308	485.624	1521.5547	54.8212
348.8022	2.9809	958.4125	0.16	1523.314	7.8322
377.5319	83.8736	998.8058	3.6712	3144.0718	1.6772
458.462	2.999	999.1826	202.4637	3144.6833	0.5872
459.6799	45.1099	1019.5057	0.8854	3149.1416	4.7923
500.1562	6.1185	1103.285	425.3778	3149.3719	0.0717
547.3319	738.1522	1111.1725	0.0076	3183.5958	0.2798
631.195	36.5169	1113.8319	582.5609	3183.6231	0.0017
641.8755	90.6155	1124.2676	4.2318	3197.5079	6.8477
677.0846	75.0452	1129.0783	746.8741	3197.584	11.7475

Table S34. Cartesian coordinates for the optimized geometry of isomer 4j-doublet of $Pt^{+}(C_2H_2)_4$ followed by its predicted frequencies (cm^{-1}) and IR intensities (km/mol).

Z	x	y	z
6	-1.404437000	0.783433000	1.016294000
6	-0.778465000	1.783734000	0.000124000
6	-1.404462000	0.783692000	-1.016269000
6	-2.133224000	0.103145000	-0.000081000
1	-1.506253000	0.811052000	2.090939000
1	-1.241068000	2.771244000	0.000248000
1	-1.506189000	0.811504000	-2.090919000
1	-2.966620000	-0.581890000	-0.000169000
78	-0.000002000	-0.629125000	-0.000013000
6	0.778470000	1.783732000	0.000125000
1	1.241080000	2.771238000	0.000250000
6	1.404438000	0.783425000	1.016295000
6	1.404472000	0.783688000	-1.016268000
1	1.506195000	0.811492000	-2.090918000
1	1.506252000	0.811042000	2.090941000
6	2.133226000	0.103138000	-0.000079000
1	2.966629000	-0.581888000	-0.000167000

Frequency	Intensity	Frequency	Intensity	Frequency	Intensity
124.1599	14.269	861.298	2.0653	1214.5294	0.9901
214.2967	2.8612	880.201	6.0025	1251.1129	0.2408
234.6989	3.9915	897.5388	8.5685	1322.8022	1.5218
237.8856	0	911.4665	0	1341.4676	27.5063
253.4171	1.1134	929.4744	8.3885	1348.3704	0
383.0887	0.2911	951.5147	1.6012	1386.6478	2.3717
410.4789	4.2423	953.9714	7.4338	1395.5669	3.4807
453.371	0	984.7248	0	3072.6357	1.5098
500.3901	4.1431	992.5477	6.9724	3082.4433	3.522
737.6694	0.6925	1007.2553	6.6921	3209.5923	1.0932
757.0018	20.1541	1123.675	25.9723	3213.1096	0.7208
797.4034	0.495	1138.1759	17.4433	3217.7299	0
853.046	0	1150.6152	0	3219.4447	10.6393
855.1667	4.5626	1167.2947	4.5475	3239.5045	21.2861
855.991	9.6672	1212.5606	0	3240.4214	4.2603

Table S35. Cartesian coordinates for the optimized geometry of isomer 4k-doublet of $Pt^+(C_2H_2)_4$ followed by its predicted frequencies (cm^{-1}) and IR intensities (km/mol).

Z	x	y	z
6	-1.921865000	-0.000299000	1.029870000
6	-1.809390000	1.025109000	0.000296000
6	-1.921868000	0.000291000	-1.029858000
6	-1.809385000	-1.025117000	-0.000285000
1	-2.059090000	-0.000597000	2.097783000
1	-1.908024000	2.098031000	0.000615000
1	-2.059098000	0.000589000	-2.097771000
1	-1.908029000	-2.098039000	-0.000604000
78	0.091121000	-0.000001000	0.000001000
6	1.600648000	1.587741000	-0.611868000
6	1.601678000	1.586587000	0.612678000
1	1.794803000	1.800789000	1.641288000
1	1.792036000	1.804165000	-1.640341000
1	1.792141000	-1.804090000	1.640327000
6	1.600715000	-1.587712000	0.611852000
6	1.601656000	-1.586587000	-0.612695000
1	1.794719000	-1.800860000	-1.641303000

Frequency	Intensity	Frequency	Intensity	Frequency	Intensity
15.1035	0	730.6115	63.8976	1242.6048	0.1156
43.8501	0.1488	735.9202	0.6359	1330.5194	9.5195
90.6971	0	736.472	0.0044	1349.0841	21.9997
115.1283	5.4436	740.5073	87.2198	1910.6815	8.7407
138.4968	7.3984	757.2042	24.8595	1919.6755	15.5063
165.9147	1.6097	758.3879	53.2045	3236.6484	0.1551
229.1594	9.7037	768.4677	0.3349	3247.8205	8.6541
304.943	0.2378	797.7439	1.0364	3256.1152	19.4041
308.7274	0.0003	887.139	0.6994	3267.2796	6.3779
310.77	9.2733	903.5486	0.0502	3336.4405	0.0001
320.689	13.8307	919.5793	0	3337.3045	249.9651
347.4279	3.0829	925.8207	10.2871	3417.7314	54.3058
361.5265	0.0164	951.27	6.3912	3419.6603	66.9873
551.7248	2.5221	964.6447	2.6871		
668.8874	0	1162.2363	0		
677.068	0.0354	1202.7569	0		

Table S36. Cartesian coordinates for the optimized geometry of isomer 4l-doublet of $Pt^+(C_2H_2)_4$ followed by its predicted frequencies (cm^{-1}) and IR intensities (km/mol).

Z	x	y	z
78	0.099096000	-0.420682000	0.000000000
1	-0.389118000	1.979556000	1.561341000
6	-0.283973000	-0.257033000	2.029726000
6	-0.283973000	0.910155000	1.548446000
1	-0.387424000	-0.967370000	2.827168000
1	-0.387424000	-0.967370000	-2.827168000
6	-0.283973000	0.910155000	-1.548446000
6	-0.283973000	-0.257033000	-2.029726000
1	-0.389118000	1.979556000	-1.561341000
1	2.368714000	-2.359350000	0.000000000
6	1.306340000	-2.467192000	0.000000000
6	0.140588000	-2.811936000	0.000000000
1	-0.810248000	-3.297017000	0.000000000
1	0.958595000	4.547394000	0.000000000
6	-0.090565000	4.373028000	0.000000000
6	-1.279457000	4.226161000	0.000000000
1	-2.339535000	4.139988000	0.000000000

Frequency	Intensity	Frequency	Intensity	Frequency	Intensity
23.9894	0.0901	569.0524	12.1239	898.4818	77.1668
28.1838	0.032	671.2495	0.007	1717.171	1.3198
32.8673	0.0197	678.8319	1.0508	1740.7153	2.9287
54.3499	0.9777	679.3685	0.2416	1964.1208	6.8392
59.3778	0.0304	729.449	0.7828	2060.977	8.5367
70.0171	1.4226	745.5627	4.9917	3266.1207	23.8052
93.3373	3.4057	755.4348	64.9106	3269.5743	277.9063
102.4565	2.0051	765.119	53.1846	3333.3727	63.5454
135.7267	5.1551	776.8058	4.9025	3335.6719	22.9295
148.6086	5.1743	781.046	82.6118	3351.1927	169.5457
194.6094	10.9594	788.9665	146.9661	3394.761	117.7078
219.5499	1.2546	804.2592	47.3636	3441.9263	30.3913
266.9358	6.0408	804.7373	1.7657	3499.2639	1.1949
374.0289	0.242	807.4296	24.6451		
439.5566	0.7844	830.003	8.0769		
509.6616	0.0101	853.0598	11.3276		

Table S37. Cartesian coordinates for the optimized geometry of isomer 4m-doublet of $Pt^{+}(C_2H_2)_4$ followed by its predicted frequencies (cm^{-1}) and IR intensities (km/mol).

Z	x	y	z
78	-0.552516000	0.027006000	0.003960000
1	0.011247000	-0.875852000	2.819810000
6	1.475242000	-0.498061000	1.121752000
6	0.609828000	-0.670723000	1.960197000
1	2.361126000	-0.393921000	0.519553000
1	0.053267000	2.642318000	1.131007000
6	-1.178259000	1.896350000	-0.630136000
6	-0.436694000	2.048906000	0.383001000
1	-1.822871000	2.247724000	-1.412089000
1	-1.375262000	-2.757926000	0.332267000
6	-1.422832000	-1.844554000	-0.228265000
6	-1.820531000	-1.120156000	-1.180954000
1	-2.377971000	-0.920806000	-2.074994000
6	4.305577000	-0.442247000	-1.153713000
6	4.666654000	0.311038000	-0.295532000
1	5.025628000	0.978971000	0.449928000
1	4.027211000	-1.110307000	-1.932472000

Frequency	Intensity	Frequency	Intensity	Frequency	Intensity
6.013	0.007	573.2616	10.4078	899.2949	40.5635
17.2453	0.0101	660.8251	0.021	1714.3291	2.5798
26.4223	0.0295	679.4446	0.9996	1743.8708	0.1854
54.9217	0.8682	699.7886	5.6752	1949.5344	34.2211
66.6233	0.1577	735.3358	1.5508	2063.0034	7.3426
86.0539	1.5357	741.2211	30.5935	3249.1446	556.9113
92.2506	0.1738	750.1973	7.9652	3285.7609	66.2025
108.4385	5.5907	759.8451	92.6149	3290.0313	79.3752
139.6904	3.3172	771.6974	67.9492	3344.0182	40.6642
147.125	10.7668	772.9572	9.8125	3349.7142	39.633
187.5771	6.215	792.732	122.8378	3398.4456	108.6038
229.1015	0.5311	797.8844	11.7436	3413.6865	43.0466
274.0696	9.9218	822.4662	34.1995	3502.1902	0.7783
376.0826	0.3717	831.1311	12.2188		
444.8339	1.397	856.723	12.9138		
510.5124	0.184	860.9201	26.4982		

Table S38. Cartesian coordinates for the optimized geometry of isomer 4n-doublet of $Pt^{+}(C_2H_2)_4$ followed by its predicted frequencies (cm^{-1}) and IR intensities (km/mol).

Z	x	y	z
78	0.000000000	0.000000000	-0.000004000
1	-1.803155000	-1.197693000	2.174996000
6	-1.502174000	-1.635401000	-0.048423000
6	-1.594560000	-1.342938000	1.138878000
1	-1.678150000	-2.183776000	-0.949683000
1	1.677656000	-2.184184000	0.949569000
6	1.594226000	-1.343222000	-1.138955000
6	1.501808000	-1.635732000	0.048331000
1	1.802865000	-1.197978000	-2.175065000
1	-1.677562000	2.184126000	0.949867000
6	-1.501803000	1.635729000	0.048579000
6	-1.594336000	1.343294000	-1.138718000
1	-1.803096000	1.198096000	-2.174809000
1	1.803365000	1.197554000	2.174898000
6	1.594669000	1.342865000	1.138810000
6	1.502169000	1.635405000	-0.048463000
1	1.678059000	2.183836000	-0.949705000

Frequency	Intensity	Frequency	Intensity	Frequency	Intensity
53.2584	0	339.2793	0	807.3071	0
63.6135	1.0887	669.2135	0	1888.6455	7.7056
87.8802	0.5292	670.5363	5.6543	1890.9653	7.9896
89.5634	0.3996	674.842	4.553	1899.3794	2.5368
92.618	0	675.2812	0.4412	1900.1983	0
130.2402	1.4448	698.9887	13.8581	3332.1818	0.4163
169.7213	9.2338	701.4843	9.4101	3332.4369	55.8765
179.9771	2.6393	711.2629	6.7024	3332.7308	0.0002
184.3911	0	720.2404	0	3333.8252	372.2774
189.6052	11.9596	735.2184	32.9175	3421.0076	50.894
207.5729	0.4221	737.8072	92.1538	3421.0199	97.7839
212.6613	5.2612	741.9438	131.709	3421.8936	104.2293
243.2908	1.8281	746.4245	0	3424.9816	0
244.4516	2.9159	781.4322	0.5118		
249.9494	0	786.3074	2.8537		
324.2856	4.6041	798.7396	15.7853		

Table S39. Cartesian coordinates for the optimized geometry of isomer 4o-doublet of $Pt^+(C_2H_2)_4$ followed by its predicted frequencies (cm^{-1}) and IR intensities (km/mol).

Z	x	y	z
78	-0.159697000	0.052818000	0.000000000
1	-0.414559000	2.557944000	1.607564000
6	-0.361638000	2.153718000	-0.619368000
6	-0.361638000	2.153718000	0.619368000
1	-0.414559000	2.557944000	-1.607564000
1	-0.844888000	-2.568146000	-1.364717000
6	-0.361638000	-0.521507000	-2.201193000
6	-0.593306000	-1.554430000	-1.581987000
1	-0.221053000	0.172135000	-2.999512000
1	-0.844888000	-2.568146000	1.364717000
6	-0.593306000	-1.554430000	1.581987000
6	-0.361638000	-0.521507000	2.201193000
1	-0.221053000	0.172135000	2.999512000
1	2.228305000	-2.051474000	0.000000000
6	2.180496000	-0.987775000	0.000000000
6	2.227937000	0.225766000	0.000000000
1	2.537472000	1.246479000	0.000000000

Frequency	Intensity	Frequency	Intensity	Frequency	Intensity
61.7209	2.8923	412.6523	3.3606	839.844	7.8077
65.3111	4.0741	663.3093	1.7619	1845.9212	5.6654
77.241	3.1718	682.7306	0.2433	1904.7784	5.8861
90.392	5.9262	687.4908	0.6817	1910.6442	1.7499
114.5137	3.5664	710.7745	0.2085	1967.0905	9.4798
121.507	0.0157	712.6943	0.2501	3325.3829	72.4178
124.8483	5.3669	725.2886	0.0058	3351.0917	47.3343
127.8194	2.9212	726.4466	6.4422	3351.9188	125.9527
139.5946	0.9647	739.0442	206.7917	3368.9669	127.2393
156.9936	3.2726	743.1518	16.1895	3395.5946	44.838
189.5906	0.4332	752.8058	16.8139	3429.1814	64.3377
225.0702	0.8765	758.1836	59.9192	3430.2658	19.58
242.1699	1.6662	773.7488	3.3583	3463.1218	20.4327
317.0751	1.9547	798.8536	23.7382		
344.0027	2.7348	813.4044	1.7054		
346.7774	2.5094	836.8543	3.1211		

Table S40. Cartesian coordinates for the optimized geometry of isomer 4a-quartet of $Pt^{+}(C_2H_2)_4$ followed by its predicted frequencies (cm^{-1}) and IR intensities (km/mol).

Z	x	y	z
6	2.069808000	-0.834207000	-0.010600000
6	1.804767000	-0.194944000	1.212220000
6	1.797665000	-0.178095000	-1.222886000
1	1.991383000	-0.710088000	2.143055000
1	1.978838000	-0.680282000	-2.161842000
6	1.274690000	1.109580000	1.224802000
6	1.267713000	1.126598000	-1.214295000
1	1.054291000	1.594063000	2.164808000
1	1.041988000	1.624105000	-2.146200000
6	1.008609000	1.773349000	0.010550000
1	0.585638000	2.767192000	0.018686000
1	2.457858000	-1.842293000	-0.018715000
78	-0.342177000	-0.331443000	0.000018000
1	-3.859351000	1.524396000	-0.000009000
6	-2.873788000	1.080163000	0.000011000
1	-2.891903000	-1.032658000	-0.000013000
6	-2.294289000	-0.114424000	0.000007000

Frequency	Intensity	Frequency	Intensity	Frequency	Intensity
7.323	1.2536	811.7756	38.0935	1339.0401	0.0088
86.1982	3.7861	861.5727	0.3233	1386.6111	0.0038
125.2207	1.0978	920.0664	0.311	1428.2473	258.8369
141.4545	0.9713	921.0052	2.0889	1496.8776	29.0181
161.6743	3.7847	991.0761	3.2463	1497.8162	28.6674
178.2629	1.9705	1012.2983	0.0036	1572.5493	0.0071
257.1051	0.9049	1012.604	0.1225	1573.8882	0.2462
367.0061	0.0238	1021.2402	0.166	3003.3716	0.4858
375.9733	1.0061	1037.884	0.0211	3201.3442	0.0131
541.2118	67.7855	1040.6282	0.515	3207.1381	0.0335
547.2223	25.8548	1042.3863	0.4773	3207.4208	0.0006
604.4619	0.0014	1150.52	20.2601	3209.5788	2.1854
604.9383	1.4717	1190.3059	0.0246	3216.2707	13.8434
639.8373	0.4147	1192.861	1.629	3216.4101	13.4802
780.9125	58.3122	1194.1049	0.0102	3222.4442	0.1081

Table S41. Cartesian coordinates for the optimized geometry of isomer 4b-quartet of $Pt^+(C_2H_2)_4$ followed by its predicted frequencies (cm^{-1}) and IR intensities (km/mol).

Z	x	y	z
6	-2.623478000	0.000001000	-0.466465000
6	-2.027535000	1.238823000	-0.319299000
6	-0.701107000	1.571206000	0.178537000
6	-0.105042000	-1.256523000	1.458678000
6	0.028046000	-0.000005000	2.083684000
6	-0.105043000	1.256516000	1.458683000
1	0.532891000	-0.000006000	3.041476000
1	-3.636751000	0.000000000	-0.846732000
1	-2.584999000	2.095903000	-0.677749000
1	-0.383770000	2.552055000	-0.163434000
1	0.435159000	-2.068069000	1.931135000
1	0.435158000	2.068060000	1.931144000
78	0.740357000	0.000001000	-0.383242000
1	-2.584999000	-2.095901000	-0.677754000
6	-2.027535000	-1.238822000	-0.319303000
6	-0.701105000	-1.571207000	0.178531000
1	-0.383770000	-2.552054000	-0.163445000

Frequency	Intensity	Frequency	Intensity	Frequency	Intensity
132.2569	0.0438	839.8632	0.0015	1437.3507	11.5122
166.1495	0.776	860.7441	2.3616	1470.8307	22.3402
177.8932	1.4746	927.7154	1.5484	1481.2843	14.0941
225.7783	4.6154	956.8547	0.2225	1514.0273	0.5691
269.0891	2.0441	986.1973	2.1488	1525.5036	1.9342
287.6343	1.4153	1002.3762	2.5816	3120.7406	0.0626
348.2302	5.61	1033.0075	16.3895	3121.297	0.7425
364.2572	0.0628	1061.7741	6.4449	3159.8469	2.2297
403.2418	7.0769	1119.1064	2.1777	3163.3329	0.0266
505.9593	10.5124	1162.3545	0.2227	3163.4013	0.4563
675.447	1.9198	1251.8366	1.7924	3166.3928	4.8534
684.5391	42.4608	1274.7771	9.1476	3184.2223	3.6104
763.7648	30.8404	1282.9589	0.0031	3185.9822	0.2284
795.9068	24.4237	1373.0866	0.0799		
800.9886	0.0077	1413.5329	0.005		
801.3368	3.7368	1430.608	9.3069		

Table S42. Cartesian coordinates for the optimized geometry of isomer 4c-quartet of $Pt^+(C_2H_2)_4$ followed by its predicted frequencies (cm^{-1}) and IR intensities (km/mol).

Z	x	y	z
6	0.081877000	-1.772166000	-0.086824000
6	1.403906000	-2.007652000	0.219557000
6	2.529014000	-1.178802000	0.282869000
6	1.034323000	1.980467000	-0.323991000
6	2.253136000	1.317904000	-0.514997000
6	2.866741000	0.138354000	-0.102830000
1	2.935699000	1.933171000	-1.094158000
1	-0.587200000	-2.634775000	-0.090885000
1	1.632016000	-3.048755000	0.434321000
1	3.413805000	-1.728804000	0.583111000
1	0.912908000	2.852771000	-0.956452000
1	3.944901000	0.220640000	-0.207004000
78	-0.906982000	-0.095580000	-0.149380000
1	-0.658121000	0.739967000	2.501386000
6	-0.173400000	0.773704000	1.535374000
6	-0.013577000	1.828242000	0.629543000
1	-0.741563000	2.640766000	0.649090000

Frequency	Intensity	Frequency	Intensity	Frequency	Intensity
76.1263	2.2806	782.3949	3.4589	1395.3161	1.408
85.8778	1.2594	786.5622	19.4734	1431.1224	49.0114
117.0551	1.4985	839.5086	17.8317	1468.8478	32.9762
174.4103	0.2563	888.0188	2.4101	1490.5258	7.1748
205.1177	2.1935	907.7522	7.6374	1514.4313	15.5691
303.9885	0.3468	932.1506	1.6791	1542.8127	21.9648
325.558	3.027	967.0029	10.8496	1563.9553	10.4348
386.2573	13.0423	993.697	12.3387	3066.8808	3.9651
485.4057	8.0115	1013.1718	2.406	3071.6388	4.7083
495.2392	0.4014	1031.2518	0.5867	3115.5005	1.0661
510.9036	1.9855	1105.7973	3.0088	3118.2447	0.4608
567.0668	7.934	1193.2973	9.1341	3133.483	1.0709
655.9329	79.127	1240.5699	11.1669	3157.7332	0.1515
693.7448	8.3049	1312.427	14.0282	3162.2296	0.5012
745.9332	11.5561	1316.7943	5.4941	3200.0987	14.4333

Table S43. Cartesian coordinates for the optimized geometry of isomer 4d-quartet of $Pt^{+}(C_2H_2)_4$ followed by its predicted frequencies (cm^{-1}) and IR intensities (km/mol).

Z	x	y	z
78	-0.873500000	-0.118524000	0.000000000
6	1.042927000	-1.471539000	-0.684370000
1	0.658939000	-2.157830000	-1.426817000
6	1.042929000	-1.471539000	0.684373000
1	0.658945000	-2.157829000	1.426822000
6	1.380182000	0.863649000	-1.441651000
6	0.395946000	1.575543000	-0.724538000
6	2.034649000	-0.299781000	-0.793534000
1	-0.190414000	2.329473000	-1.231909000
1	2.991753000	-0.568716000	-1.235461000
6	0.395953000	1.575549000	0.724536000
6	2.034651000	-0.299779000	0.793532000
1	-0.190406000	2.329481000	1.231907000
1	2.991756000	-0.568712000	1.235458000
6	1.380183000	0.863651000	1.441648000
1	1.483948000	1.012253000	2.508187000
1	1.483949000	1.012253000	-2.508189000

Frequency	Intensity	Frequency	Intensity	Frequency	Intensity
67.7587	7.2427	874.2018	1.1239	1264.6999	59.7944
113.1939	13.3633	929.338	1.5654	1325.412	3.6698
185.5547	0.1899	937.9165	0.3074	1386.3506	102.8039
210.0765	0.6981	939.8926	2.487	1396.2476	15.2881
294.2428	27.5193	950.8001	5.7606	1435.2095	6.0649
352.0642	11.073	951.7689	3.4205	1480.4712	0.461
391.9717	1.7104	968.9378	9.5609	1501.1856	5.7624
416.6402	4.4707	1008.3672	2.0099	3100.2925	0.0548
546.195	0.0003	1058.4214	0.1865	3104.3933	0.0207
567.8861	0.0398	1091.5791	2.5902	3182.0676	0.2162
718.0223	143.6393	1134.4258	0.0144	3186.2775	9.8875
728.9854	6.9556	1153.3691	15.0458	3187.0183	3.0396
772.3328	40.8341	1188.4256	7.8962	3193.1439	9.6608
800.4034	0.0011	1195.599	8.7626	3199.2194	5.262
835.1461	29.7453	1262.5194	9.572	3209.1831	3.8396

Table S44. Cartesian coordinates for the optimized geometry of isomer 4e-quartet of $Pt^{+}(C_2H_2)_4$ followed by its predicted frequencies (cm^{-1}) and IR intensities (km/mol).

Z	x	y	z
6	-2.672530000	0.936898000	-0.000194000
6	-2.859211000	-0.447641000	-0.000325000
6	-1.329521000	1.321864000	0.000147000
1	-3.830054000	-0.925241000	-0.000548000
1	-1.026098000	2.362692000	0.000331000
6	-1.656551000	-1.157586000	-0.000093000
1	-1.637295000	-2.244432000	-0.000072000
1	-3.481452000	1.656877000	-0.000313000
78	0.024118000	-0.121457000	0.000171000
6	2.177125000	-0.549175000	0.737085000
6	2.021963000	0.879460000	0.728098000
1	2.029473000	1.644143000	1.486687000
1	2.382500000	-1.283523000	1.498285000
1	2.380945000	-1.281234000	-1.501509000
6	2.176272000	-0.548079000	-0.738977000
1	2.028131000	1.646442000	-1.485015000
6	2.021231000	0.880583000	-0.727607000

Frequency	Intensity	Frequency	Intensity	Frequency	Intensity
19.7684	0.057	795.9474	6.8798	1315.0824	20.8278
52.4274	6.0535	809.7633	1.0198	1357.3494	65.1229
73.2173	4.0836	842.77	0.0151	1357.8182	12.503
222.9279	3.5949	898.9571	7.7727	1435.4914	52.016
230.2836	4.7942	938.1678	0.5997	1488.1307	82.8256
241.7441	1.0786	947.0223	0.0054	3129.3059	3.2204
255.1394	0.1864	958.9661	9.3691	3159.9229	0.1544
367.2766	5.1784	973.1233	0.0089	3182.7386	0.8233
377.1146	0.2206	984.5361	10.2632	3198.1976	2.6949
450.7322	0.0855	1064.5421	0.3835	3238.187	0.3588
523.828	0.0562	1089.1888	6.106	3250.5331	22.5572
649.5683	7.1052	1118.1979	4.535	3253.3386	22.7576
671.1485	69.7425	1207.8768	0.0001	3267.4813	11.1623
732.2587	79.2586	1212.4771	12.4376		
760.6417	4.2119	1239.374	6.9635		
772.8199	0.0025	1275.1574	18.7985		

Table S45. Cartesian coordinates for the optimized geometry of isomer 4f-quartet of $Pt^{+}(C_2H_2)_4$ followed by its predicted frequencies (cm^{-1}) and IR intensities (km/mol).

Z	x	y	z
6	-2.032465000	0.851865000	0.918329000
6	-1.879131000	1.427087000	-0.357008000
6	-1.926647000	0.088873000	-0.981650000
6	-2.120253000	-0.500832000	0.334795000
1	-2.088081000	1.240953000	1.922837000
1	-1.740991000	2.430550000	-0.725642000
1	-2.109990000	-0.250406000	-1.987911000
1	-2.545933000	-1.429460000	0.678624000
78	-0.004447000	-0.332759000	0.017124000
1	2.518633000	-1.446469000	0.696547000
6	2.106421000	-0.514553000	0.344701000
6	2.063426000	0.847779000	0.909548000
1	2.135084000	1.248473000	1.908541000
6	1.914425000	1.409324000	-0.371125000
1	1.801372000	2.411693000	-0.751585000
6	1.920594000	0.063815000	-0.980063000
1	2.098586000	-0.290272000	-1.982252000

Frequency	Intensity	Frequency	Intensity	Frequency	Intensity
4.0575	8.4832	811.0198	5.1913	1206.0509	0.4009
47.408	1.4694	813.7338	5.6348	1247.7824	6.8964
70.3334	2.3137	843.072	0.675	1270.3689	4.0479
89.3687	2.1798	855.7192	2.1137	1316.0298	0.4115
152.2436	12.9102	910.4263	7.5048	1318.9461	35.3603
235.4515	0.9537	914.4729	0.8451	1383.313	10.5346
253.2597	0.4447	933.2964	24.8982	1390.7667	28.9567
320.9774	2.3787	939.5445	2.2254	3223.097	0.4856
389.2725	2.599	945.3702	8.5066	3224.6303	0.4265
474.3043	9.1857	956.7088	10.7384	3235.6441	15.4957
514.5061	1.2678	979.6021	8.2434	3237.4378	19.1682
718.0098	109.1015	995.1456	3.7423	3240.9811	10.3833
726.9472	14.2211	1181.1941	43.0377	3242.3603	13.8905
792.0557	1.1649	1196.2829	1.471	3254.2773	10.8606
798.9586	38.5031	1205.2537	0.0993	3255.977	3.3927

Table S46. Cartesian coordinates for the optimized geometry of isomer 4g-quartet of $Pt^{+}(C_2H_2)_4$ followed by its predicted frequencies (cm^{-1}) and IR intensities (km/mol).

Z	x	y	z
6	1.497208000	-0.783034000	-0.857278000
6	2.480257000	0.270918000	-0.282986000
6	2.456587000	-0.645208000	0.960925000
6	1.582223000	-1.546226000	0.335003000
1	1.356509000	-1.071845000	-1.890042000
1	3.453505000	0.326413000	-0.768197000
1	2.940969000	-0.587063000	1.923140000
1	1.203540000	-2.517443000	0.619974000
78	-0.490071000	-0.057907000	-0.060819000
6	1.843833000	1.597846000	-0.075894000
1	2.446394000	2.502706000	-0.107820000
6	0.492818000	1.673224000	0.130917000
1	0.004770000	2.614576000	0.348327000
6	-2.522715000	-0.617406000	0.052482000
6	-2.380772000	0.590639000	0.410480000
1	-2.803862000	1.517006000	0.754093000
1	-3.072894000	-1.512147000	-0.177488000

Frequency	Intensity	Frequency	Intensity	Frequency	Intensity
63.0282	3.5712	762.0234	9.1359	1311.9819	2.796
99.2867	14.3109	765.6312	7.144	1348.7905	8.1525
112.7733	0.9824	778.7999	56.1606	1384.4821	28.7297
131.8492	6.1438	854.7502	40.7254	1465.9399	113.8468
192.9263	2.9637	859.9599	14.9646	1691.8773	7.1163
246.9908	1.8444	878.5366	4.0128	3095.5555	0.0292
257.9272	14.3341	896.6699	7.662	3125.5456	0.5859
341.9396	5.3391	924.1619	10.7624	3182.8374	0.7596
362.8326	4.2239	949.5128	3.6683	3186.2724	9.27
397.6474	5.7269	1008.2981	6.8454	3209.7404	4.8155
490.0512	10.735	1025.2976	0.9602	3234.5676	3.4907
555.8862	8.9221	1119.2852	5.6742	3256.8902	69.7154
618.1619	70.2325	1123.9885	64.1995	3314.091	45.2565
691.5318	43.1861	1149.2532	6.7307		
718.7844	0.3276	1210.3353	2.3116		
728.1873	5.1491	1234.2405	86.3974		

Table S47. Cartesian coordinates for the optimized geometry of isomer 4h-quartet of $Pt^+(C_2H_2)_4$ followed by its predicted frequencies (cm^{-1}) and IR intensities (km/mol).

Z	x	y	z
78	-0.259768000	-0.010229000	-0.077321000
6	1.452507000	0.325864000	-1.150796000
6	2.625843000	-0.146259000	-0.561968000
1	3.586965000	-0.084147000	-1.058552000
1	1.483150000	0.829844000	-2.112229000
6	2.485318000	-0.705546000	0.713344000
6	1.203310000	-0.669935000	1.253722000
1	0.993506000	-1.046882000	2.248737000
1	3.323452000	-1.142085000	1.244712000
6	-1.696686000	-1.562874000	0.404805000
6	-1.911866000	-1.255778000	-0.784625000
1	-2.360685000	-1.268007000	-1.758083000
1	-1.827924000	-2.142735000	1.296577000
1	0.644425000	2.857908000	-0.102775000
6	-0.160195000	2.233359000	0.220416000
6	-1.231671000	1.931526000	0.744835000
1	-2.180372000	1.891814000	1.234282000

Frequency	Intensity	Frequency	Intensity	Frequency	Intensity
28.8868	3.4975	689.6051	0.9734	1325.6613	35.1734
61.6296	3.6771	706.9128	23.645	1447.4336	66.9659
73.3781	2.5493	718.9951	13.7264	1489.4663	17.8469
110.6498	2.6352	732.5566	55.0835	1791.3397	14.2528
114.2715	5.2036	737.0063	29.9578	1874.6865	0.1791
134.7988	0.2911	744.5836	49.9771	3140.0513	0.1205
171.0643	4.8237	770.4461	13.7664	3153.6653	0.1867
235.985	2.6777	781.8037	1.3914	3168.3082	0.1191
265.6069	0.6971	790.7321	9.2592	3179.3454	0.6805
306.232	7.902	811.2207	2.3834	3292.018	89.5951
327.6671	1.4552	966.131	1.4548	3336.3887	113.0596
367.4296	4.9363	1000.8143	0.3634	3359.1078	60.689
408.0579	0.1651	1039.066	0.5146	3415.1552	51.3572
425.382	1.9536	1093.0036	10.7609		
573.4584	3.778	1126.391	0.2821		
673.5898	45.5324	1225.9505	3.6389		

Table S48. Cartesian coordinates for the optimized geometry of isomer 4i-quartet of $Pt^+(C_2H_2)_4$ followed by its predicted frequencies (cm^{-1}) and IR intensities (km/mol).

Z	x	y	z
78	0.000051000	0.000875000	0.298451000
6	1.723481000	1.113363000	0.295326000
6	2.793290000	0.525322000	-0.382909000
1	3.759769000	0.999913000	-0.505864000
1	1.881577000	2.080012000	0.766700000
6	2.530739000	-0.759716000	-0.901258000
6	1.289114000	-1.288348000	-0.601226000
1	0.988818000	-2.291196000	-0.880301000
1	3.259342000	-1.314653000	-1.482034000
1	-1.878109000	-2.079850000	0.770871000
6	-1.721856000	-1.113670000	0.297931000
6	-2.792604000	-0.528585000	-0.381367000
1	-3.758442000	-1.004764000	-0.503203000
6	-2.531825000	0.755685000	-0.902490000
1	-3.261143000	1.308323000	-1.484561000
6	-1.290952000	1.286689000	-0.603454000
1	-0.992122000	2.289542000	-0.884129000

Frequency	Intensity	Frequency	Intensity	Frequency	Intensity
62.2845	3.9578	760.4205	5.0799	1265.4668	3.5614
93.282	2.2371	782.0172	4.4508	1349.1037	29.7677
128.345	0.3733	815.2543	2.1668	1360.6076	0.6812
182.7476	5.9384	820.4298	0.0138	1460.8119	127.6556
230.8439	2.415	957.8476	5.4258	1462.7183	11.6012
317.7153	1.3336	964.3609	0.6062	1486.0475	24.3491
343.7698	8.7101	993.7353	1.7673	1494.6854	7.9197
346.1445	11.3015	994.0251	0.0799	3131.1693	0.9694
372.1491	0.1523	1036.785	7.5226	3131.6641	0.294
445.2007	6.7458	1044.9787	0.6043	3158.1984	4.5908
492.255	0.5082	1102.1019	1.5204	3158.2608	0.29
530.6803	2.4207	1106.0046	12.953	3173.6786	0.9363
635.5225	2.9448	1128.2619	1.2167	3174.5202	0.1716
665.7486	50.387	1132.6814	1.2753	3180.7993	2.3468
693.2709	85.3535	1258.7482	24.9151	3180.8626	0.9946

Table S49. Cartesian coordinates for the optimized geometry of isomer 4j-quartet of $Pt^{+}(C_2H_2)_4$ followed by its predicted frequencies (cm^{-1}) and IR intensities (km/mol).

Z	x	y	z
6	1.486790000	1.757130000	0.782015000
6	1.963711000	0.726238000	-0.237659000
6	0.708928000	1.169280000	-1.050055000
6	0.383244000	2.109446000	-0.022218000
1	1.860320000	2.095096000	1.736311000
1	2.902767000	0.996767000	-0.724797000
1	0.557598000	1.221706000	-2.118668000
1	-0.347349000	2.905480000	0.038452000
78	-0.826432000	-0.000030000	-0.000002000
6	1.963741000	-0.726081000	0.237760000
1	2.902764000	-0.996535000	0.725002000
6	0.708889000	-1.169224000	1.050014000
6	1.487022000	-1.757014000	-0.781965000
1	1.860775000	-2.095051000	-1.736150000
1	0.557515000	-1.221749000	2.118617000
6	0.383409000	-2.109416000	0.022111000
1	-0.347119000	-2.905503000	-0.038656000

Frequency	Intensity	Frequency	Intensity	Frequency	Intensity
46.4837	5.1368	857.1104	22.6855	1217.5986	0.0095
144.2356	2.5458	894.5087	0.0982	1268.0354	0.384
167.7045	5.6372	894.5772	15.7596	1318.8324	3.7355
211.5029	0.002	896.0374	0.017	1339.322	9.5192
243.0485	1.3967	913.9268	2.8413	1345.7507	0.0005
273.2554	0.5655	924.6497	4.9358	1371.6447	26.9091
303.5034	1.9194	939.8745	1.5916	1375.7855	6.8516
413.4679	0.0004	1014.9208	0.0947	3058.4008	0.1828
495.9113	3.0616	1017.1749	0.9256	3066.3581	0.1025
602.8254	50.0657	1044.4937	4.098	3186.0846	2.2421
638.6468	32.8957	1127.1907	12.353	3186.7378	1.0829
741.7313	8.8566	1155.1442	9.4393	3204.1661	9.9156
770.0356	0.3559	1155.8013	3.3772	3204.368	2.5897
813.3835	11.1284	1167.2778	2.899	3229.6485	7.8111
816.6687	5.6426	1212.666	0.8008	3229.6881	2.1134

Table S50. Cartesian coordinates for the optimized geometry of isomer 4k-quartet of $Pt^+(C_2H_2)_4$ followed by its predicted frequencies (cm^{-1}) and IR intensities (km/mol).

Z	x	y	z
6	-3.281810000	0.119185000	0.258340000
6	-2.595270000	-0.048861000	-0.975885000
6	-1.665947000	-0.969244000	-0.313377000
6	-2.381274000	-0.729187000	0.949778000
1	-4.197541000	0.618932000	0.540353000
1	-2.752563000	0.270509000	-1.991962000
1	-1.289829000	-1.904479000	-0.705201000
1	-2.307446000	-1.113908000	1.952471000
78	0.495375000	0.040808000	-0.021418000
6	-0.251054000	1.956540000	0.132010000
6	1.006805000	2.057751000	0.117019000
1	1.952000000	2.563712000	0.142422000
1	-1.234011000	2.382638000	0.184999000
1	3.322931000	-0.667428000	0.044014000
6	2.297458000	-0.982151000	0.032673000
6	1.348199000	-1.819999000	0.039257000
1	1.004580000	-2.837232000	0.064592000

Frequency	Intensity	Frequency	Intensity	Frequency	Intensity
13.1229	0.3729	629.2123	6.6664	1207.7711	1.0017
63.4193	0.4883	729.3019	4.9296	1243.5945	2.4238
96.2767	1.8308	732.9489	63.2109	1254.9244	9.6083
97.58	8.754	758.741	26.1766	1340.789	10.5957
106.3916	1.2995	764.4398	2.4511	1352.3395	51.1762
138.8843	0.1818	773.4812	6.326	1695.8034	2.5073
158.6061	0.1203	814.5912	31.2667	1741.5798	26.7653
166.5091	3.8375	835.2646	4.0982	3183.2336	4.0903
203.9102	1.0425	844.8177	15.139	3210.9699	2.2686
325.0638	0.2002	881.2286	92.3465	3252.448	10.3946
452.4419	25.338	893.4729	1.3631	3261.1628	0.105
488.6707	1.1626	898.9166	14.9749	3276.9209	64.0531
496.6615	1.032	935.2414	0.0922	3286.6196	71.6978
576.4464	4.6963	973.0956	0.8269	3336.7784	67.4762
618.1054	88.9451	1007.3319	5.2909	3346.0036	49.081

Table S51. Cartesian coordinates for the optimized geometry of isomer 4l-quartet of $Pt^{+}(C_2H_2)_4$ followed by its predicted frequencies (cm^{-1}) and IR intensities (km/mol).

Z	x	y	z
78	0.488428000	0.000632000	0.070368000
1	0.765400000	2.225253000	2.026129000
6	-0.728020000	1.755387000	0.374772000
6	0.209359000	1.796357000	1.213044000
1	-1.626870000	2.072620000	-0.120405000
1	0.745837000	-2.178600000	2.079000000
6	-0.745201000	-1.734430000	0.418399000
6	0.192728000	-1.764465000	1.256346000
1	-1.648229000	-2.052793000	-0.068412000
1	3.344449000	-0.022801000	-0.742400000
6	2.310849000	-0.022020000	-1.038061000
6	1.378136000	-0.028784000	-1.887687000
1	0.978305000	-0.039799000	-2.884824000
6	-3.917385000	-0.012617000	-1.145892000
6	-4.122240000	0.003253000	0.033892000
1	-4.345466000	0.017380000	1.073251000
1	-3.780132000	-0.026609000	-2.199898000

Frequency	Intensity	Frequency	Intensity	Frequency	Intensity
		392.9495	0.0902	800.8085	36.0097
2.3531	0.1356	442.8252	0.0115	848.7369	2.24
26.2434	0.1122	656.2368	13.0676	1688.9653	10.4072
45.2466	0.3191	668.1393	0.2364	1700.5337	8.8637
56.0553	0.0775	669.6135	0.1098	1787.3536	0.1266
66.6041	12.3969	673.3561	0.0022	2062.7036	6.9962
77.082	5.4976	701.0388	6.8643	3257.3371	103.7405
84.703	4.0083	716.9082	114.2547	3264.1251	183.6383
94.8278	3.5871	717.5812	32.4931	3264.5348	1.4296
97.8702	6.6189	731.4886	46.981	3318.5179	63.1908
173.2284	0.338	745.3194	8.4725	3326.421	66.3139
174.8592	0.0058	755.2615	0.8506	3329.594	24.2213
250.568	3.1913	778.1833	78.2637	3399.6447	103.5788
287.8355	1.4062	781.8892	172.2491	3504.0479	0.9566
308.7551	2.7179	786.8303	1.503		
384.6226	0.0431	793.6792	41.9001		

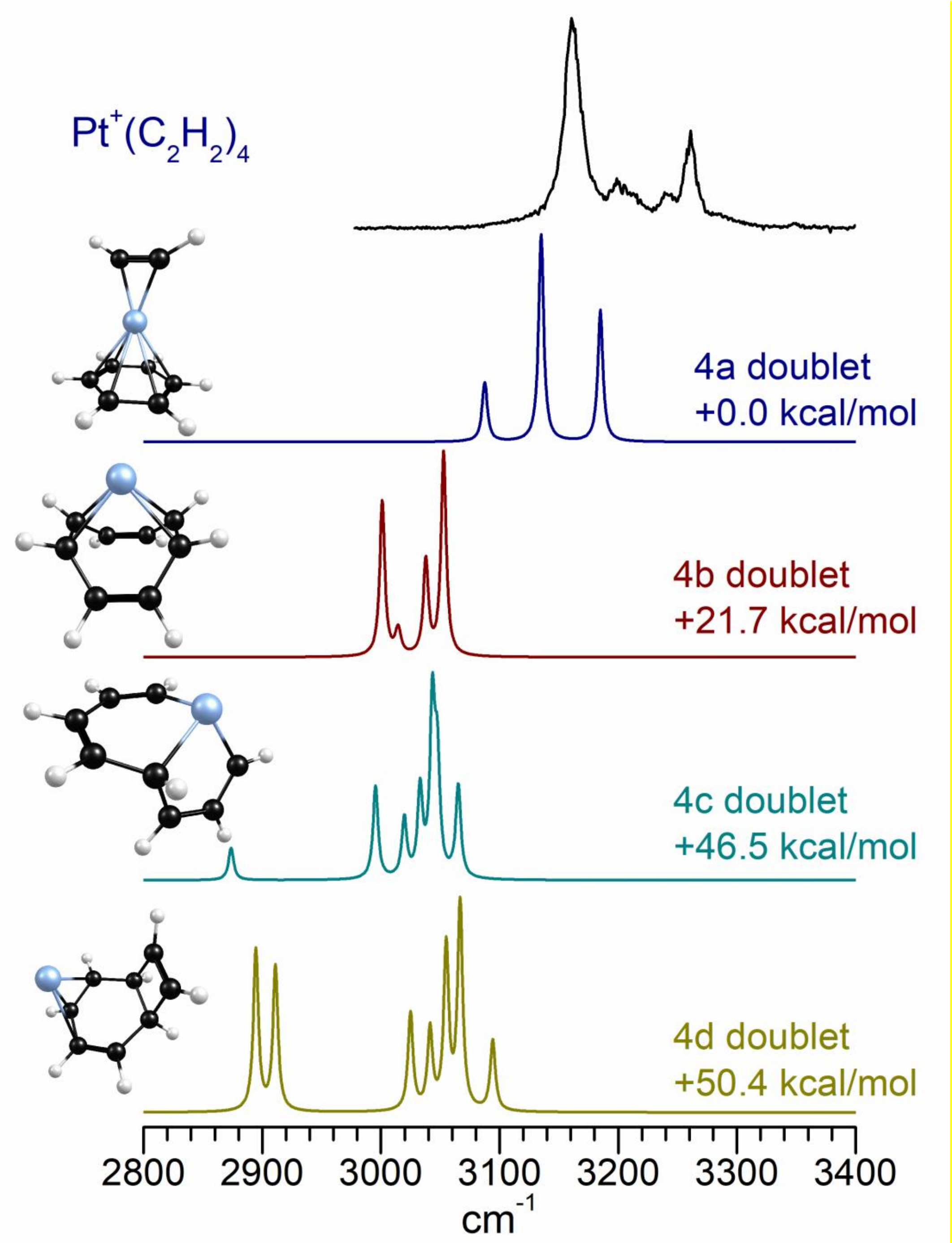

Pt+(C2H2)4
4a doublet
+0.0 kcal/mol
4b doublet
+21.7 kcal/mol
4c doublet
+46.5 kcal/mol
4d doublet
+50.4 kcal/mol
2800
2900
3000
3100
3200
3300
3400
cm-1

S56.

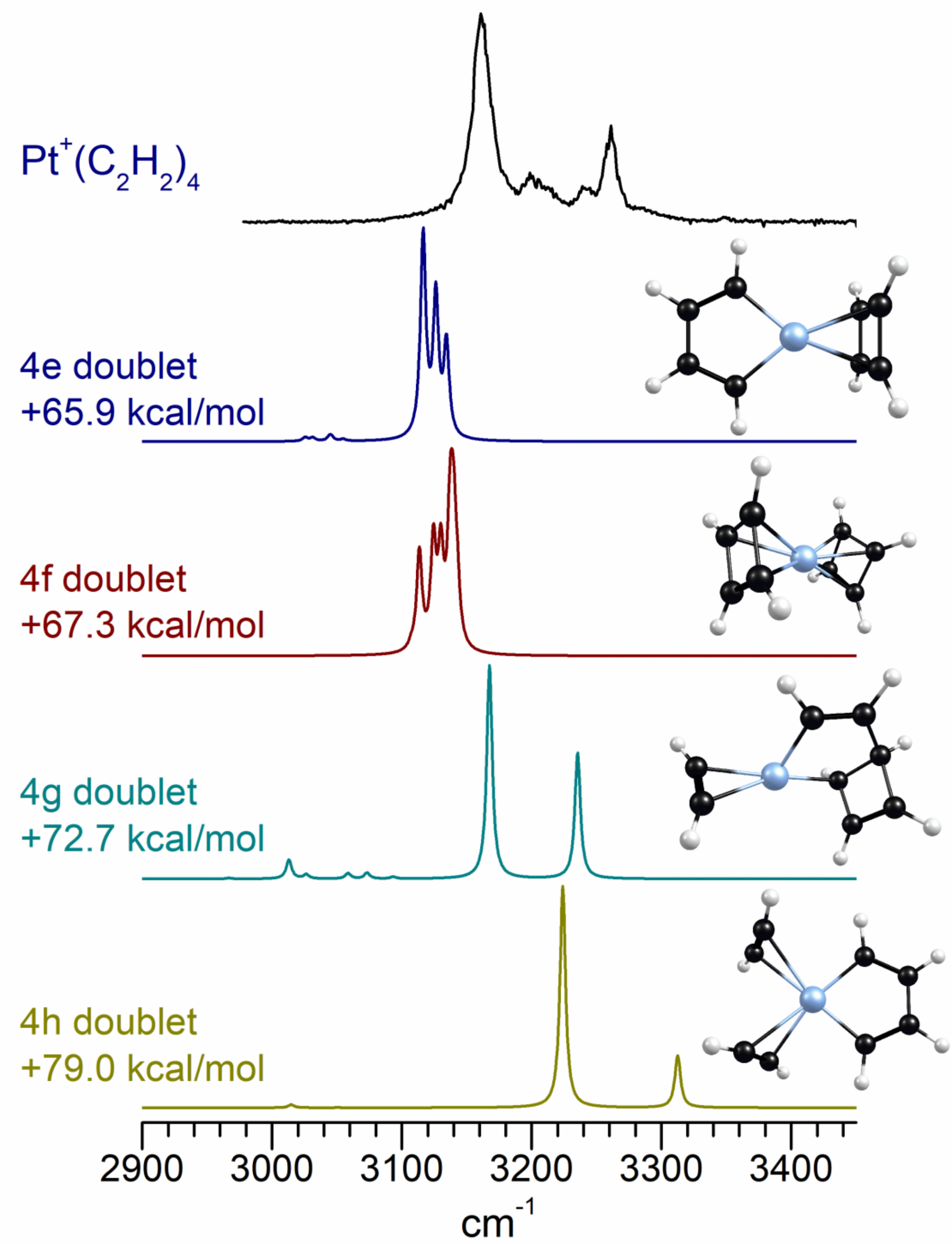

Pt+(C2H2)4
4e doublet
+65.9 kcal/mol
4f doublet
+67.3 kcal/mol
4g doublet
+72.7 kcal/mol
4h doublet
+79.0 kcal/mol
2900
3000
3100
3200
3300
3400
cm-1

S57.

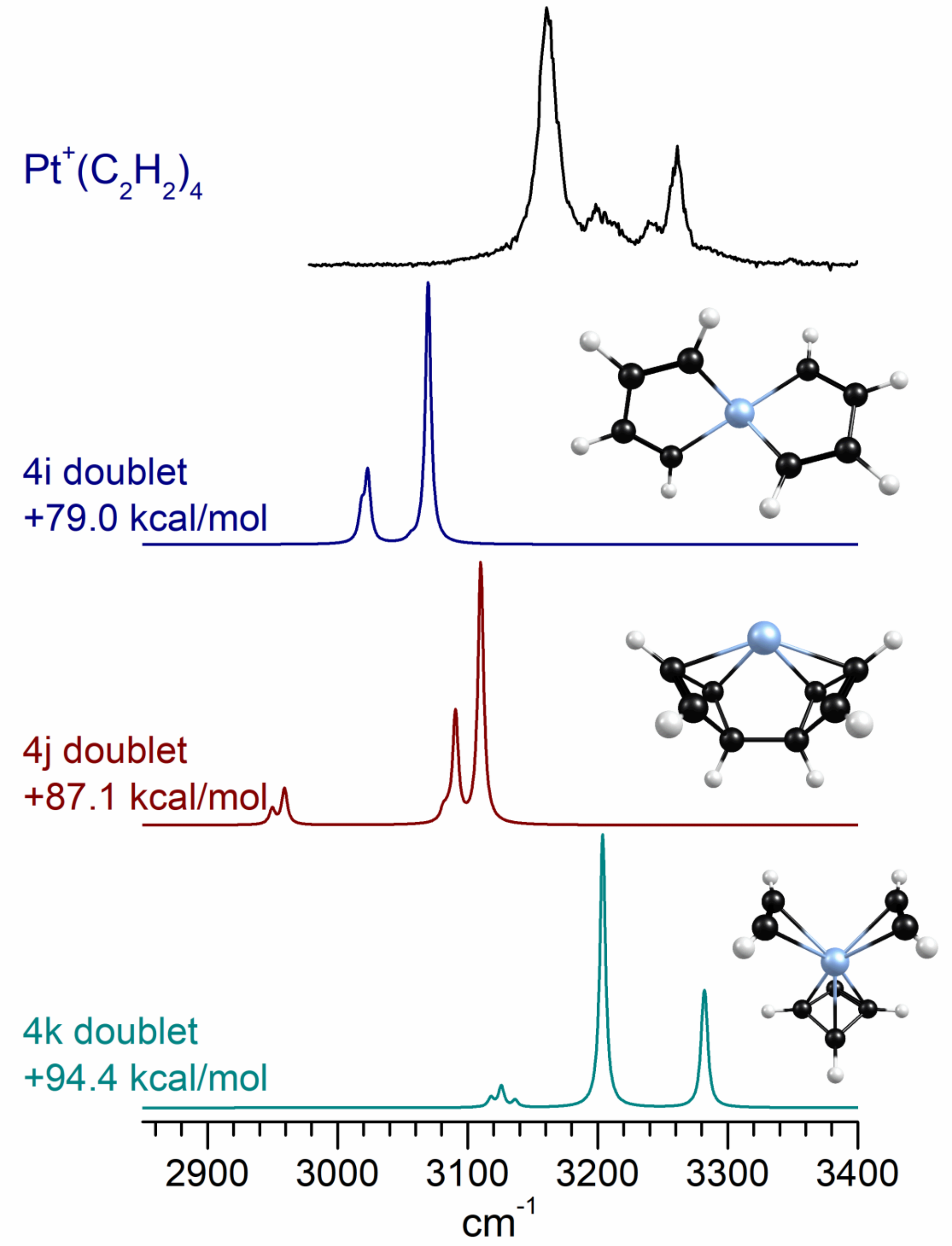
Pt+(C2H2)4
4i doublet
+79.0 kcal/mol
4j doublet
+87.1 kcal/mol
4k doublet
+94.4 kcal/mol
2900
3000
3100
3200
3300
3400
cm-1

S58.

Table S52. $Pt^{+}(C_2H_2)_5$ calculated at the

Isomer	2s + 1	E (hartree)	Relative E (kcal/mol)
5a	2	-506.272954	0
5a	4	-506.182179	56.96218467
5b	2	-506.245885	16.98605758
5b	4	-506.175634	61.06923505
5c	2	-506.23728	22.38577776
5c	4	-506.164952	67.77229269
5d	2	-506.208567	40.40346113
5d	4	-506.164658	67.95678051
5e	2	-506.207266	41.21985113
5e	4	-506.173078	62.67314961
5f	2	-506.205454	42.35689854
5f	4	-506.126465	91.92325497
5g	2	-506.202217	44.38814714
5g	4	-506.121077	95.30427674
5h	2	-506.184795	55.32061953
5h	4	-506.138675	84.26136266
5i	2	-506.170524	64.27580915
5i	4	-506.116801	97.98750782
5j	2	-506.147322	78.83528708
5j	4	-506.123312	93.90179276
5k	2	-506.140206	83.30064545
5k	4	-506.05907	134.214265
5l	2	-506.134735	86.73375051
5l	4	-506.106915	104.1910678
5m	2	-506.133421	87.55829814
5m	4	-506.07357	125.1153757
5n	2	-506.127616	91.20099141
5n	4	-506.102067	107.2332344
5o	2	-506.123327	93.89238012
5o	4	-506.064922	130.5420788
5p	2	-506.105191	105.2728944
5p	4	-506.055232	136.6226469
5q	2	-506.085879	117.3913599
5q	4	-506.043239	144.1483696
5r	2	-506.080111	121.0108353
5r	4	-506.03995	146.2122487
5s	2	-506.073807	124.9666559
5s	4	-506.055269	136.599429
5t	2	-506.073567	125.1172582
5t	4	-506.05478	136.9062812

Isomer	2s + 1	E (hartree)	Relative E (kcal/mol)
5u	2	-506.072878	125.5496123
5u	4	-505.989756	177.709466
5v	2	-506.070794	126.8573424
5v	4	-506.028972	153.1010492
5w	2	-505.999435	171.6358005
5x	2	-505.998392	172.290293
5x	4	-505.917284	223.1863423
5y	2	-505.997313	172.9673759
5y	4	-505.897904	235.3474785
5z	2	-505.986324	179.8630789
5z	4	-505.917298	223.1775572
5aa	2	-505.982387	182.3335843

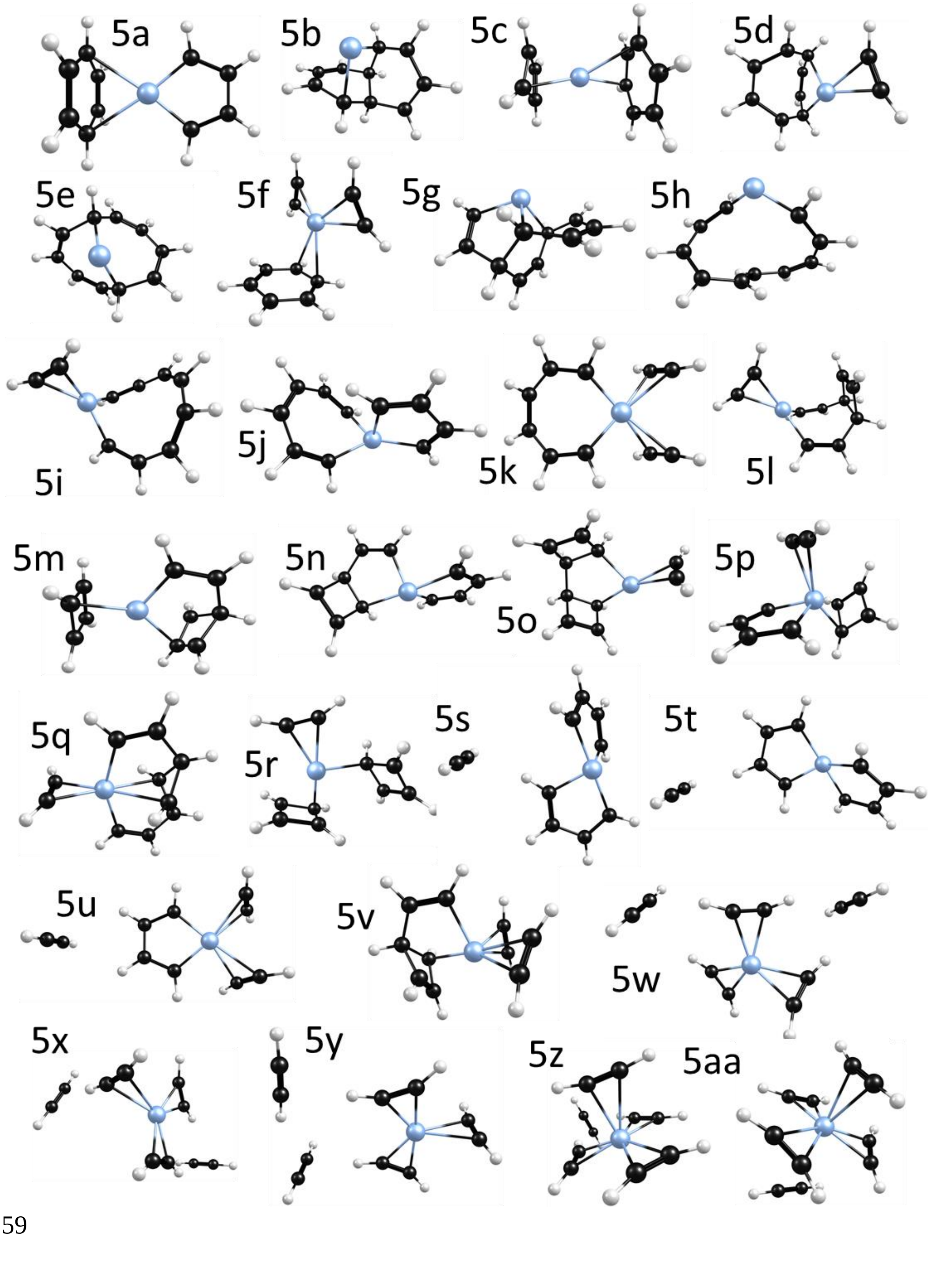
5a
5b
5c
5d
5e
5f
5g
5h
5i
5j
5k
5l
5m
5n
5o
5p
5q
5r
5s
5t
5u
5v
5w
5x
5y
5z
5aa

S59

Table S53. Cartesian coordinates for the optimized geometry of isomer 5a-doublet of $Pt^+(C_2H_2)_5$ followed by its predicted frequencies (cm^{-1}) and IR intensities (km/mol).

Z	x	y	z
78	-0.192403000	0.000006000	-0.000015000
6	-1.693260000	1.242487000	0.000007000
6	-2.977912000	0.697434000	0.000025000
1	-3.873634000	1.304355000	0.000037000
1	-1.539689000	2.317218000	0.000007000
6	-2.977908000	-0.697442000	0.000025000
6	-1.693252000	-1.242486000	0.000006000
1	-1.539671000	-2.317216000	0.000006000
1	-3.873626000	-1.304369000	0.000039000
6	1.925639000	1.396123000	0.000066000
6	1.961600000	0.699811000	1.221217000
6	1.961607000	0.699898000	-1.221134000
1	1.970598000	1.244260000	2.153972000
1	1.970611000	1.244412000	-2.153851000
6	1.961577000	-0.699916000	1.221167000
6	1.961589000	-0.699831000	-1.221184000
1	1.970556000	-1.244431000	2.153884000
1	1.970579000	-1.244279000	-2.153940000
6	1.925599000	-1.396141000	-0.000034000
1	1.909976000	-2.476600000	-0.000072000
1	1.910048000	2.476582000	0.000104000

Table S54. Predicted frequencies (cm^{-1}) and IR intensities (km/mol) of isomer 5a-doublet of $Pt^{+}(C_2H_2)_5$

Frequency	Intensity	Frequency	Intensity	Frequency	Intensity
17.5239	0	824.6722	0.1309	1333.7789	1.8343
52.8584	3.5317	907.6668	0.0029	1377.7133	60.2684
85.1274	2.5451	929.7792	1.4987	1387.6189	0
86.944	1.8063	940.5529	2.3732	1447.2076	60.8969
141.7398	2.5591	981.7363	0	1498.9392	23.7875
152.695	1.9879	995.041	2.7714	1504.6	23.7037
274.5639	2.4617	1012.8228	0	1505.6201	24.6424
378.2382	0	1023.2398	1.0181	1581.3205	0
398.8176	5.4225	1028.0155	0.0672	1596.9917	0.7706
409.4981	2.4507	1041.2682	0.7685	3141.2021	0.5644
412.057	0	1045.3287	0.0001	3146.2582	0.1918
513.3704	1.9947	1052.5297	1.1192	3186.9831	0.0006
610.5402	0	1080.043	0.0011	3195.9777	0.0076
613.4375	0.0067	1099.2779	5.9212	3200.172	0.0001
681.5687	0.1509	1128.1859	0.0935	3200.7864	0.6689
692.847	63.2054	1188.751	0.1883	3205.4797	0
701.4075	7.2078	1196.3828	0	3211.3328	3.4519
767.7518	83.989	1196.7594	0.0496	3215.6114	4.9733
807.6038	0	1276.1722	2.1043	3219.9852	0.0005

Table S55. Cartesian coordinates for the optimized geometry of isomer 5b-doublet of $Pt^{+}(C_2H_2)_5$ followed by its predicted frequencies (cm^{-1}) and IR intensities (km/mol).

Z	x	y	z
1	2.387507000	2.134330000	1.722827000
6	1.804585000	1.679583000	0.933925000
6	0.982182000	2.407940000	0.163798000
6	0.224997000	1.751734000	-0.886918000
6	0.608076000	0.475260000	-1.355317000
6	1.851112000	-0.182137000	-0.750560000
6	1.851031000	0.182850000	0.750572000
6	0.608252000	-0.475052000	1.355313000
6	0.225687000	-1.751673000	0.886899000
6	0.983160000	-2.407577000	-0.163796000
6	1.805288000	-1.678889000	-0.933905000
1	0.858533000	3.471846000	0.308082000
1	-0.471177000	2.334681000	-1.478380000
1	0.312923000	0.172891000	-2.353403000
1	2.388446000	-2.133407000	-1.722765000
1	0.859987000	-3.471542000	-0.308052000
1	-0.470284000	-2.334892000	1.478333000
1	0.312966000	-0.172809000	2.353398000
1	2.729234000	0.240999000	-1.245473000
1	2.729320000	-0.239927000	1.245494000
78	-0.991073000	-0.000185000	-0.000002000

Table S56. Predicted frequencies (cm^{-1}) and IR intensities (km/mol) of isomer 5b-doublet of $Pt^+(C_2H_2)_5$

Frequency	Intensity	Frequency	Intensity	Frequency	Intensity
116.4554	9.1846	914.7561	15.5126	1316.8876	0.5393
163.7093	6.2391	925.2706	3.3209	1377.8758	5.6866
169.2207	1.279	939.7793	15.6763	1382.17	0.3999
243.7768	0.0058	956.4889	40.3155	1415.9218	12.1185
302.2401	8.3967	976.1297	0.9917	1438.3873	12.2815
307.2703	2.0899	999.668	0.6446	1450.7943	11.2223
378.0802	17.8736	1006.2759	2.9158	1466.0748	20.8883
415.9655	0.1278	1008.4838	0.125	1627.0735	30.3322
466.4646	7.0303	1016.0487	0.0101	1641.8468	13.7677
507.1614	5.2795	1024.3026	0.0868	3039.8671	1.343
529.9891	1.0371	1043.249	10.0867	3044.3537	0.6885
599.0757	2.6252	1065.5474	1.8652	3150.1813	4.2613
605.4636	0.8863	1161.9315	1.1835	3150.7434	0.2704
664.9408	4.529	1167.1768	3.7167	3166.2219	5.1662
745.4509	27.4713	1185.9402	0.0622	3167.7448	0.3618
747.4391	36.5636	1193.4645	0.034	3188.6863	0.4026
770.6596	4.5792	1254.6907	0.4868	3188.711	0.4709
848.1452	0.7839	1284.6022	2.4015	3205.3026	0.0503
886.0238	0.405	1310.6354	9.8023	3205.3127	0.0723

Table S57. Cartesian coordinates for the optimized geometry of isomer 5c-doublet of $Pt^{+}(C_2H_2)_5$ followed by its predicted frequencies (cm^{-1}) and IR intensities (km/mol).

Z	x	y	z
78	0.264222000	-0.000003000	0.033735000
6	2.158272000	-1.014972000	-0.247550000
6	2.532944000	-0.000019000	0.725174000
1	2.819156000	-0.000045000	1.762179000
1	2.218984000	-2.091481000	-0.262981000
1	1.893657000	0.000057000	-2.350711000
6	1.967112000	0.000029000	-1.278093000
1	2.218989000	2.091489000	-0.262873000
6	2.158279000	1.014979000	-0.247497000
6	-1.508691000	0.709240000	1.255823000
6	-1.508689000	-0.709210000	1.255838000
6	-1.885607000	1.383842000	0.076021000
1	-1.253883000	-1.262103000	2.147510000
1	-1.833409000	2.462933000	0.052205000
6	-1.885596000	-1.383838000	0.076048000
6	-2.493688000	0.688182000	-0.997253000
1	-1.833394000	-2.462930000	0.052253000
1	-2.914745000	1.244666000	-1.822016000
6	-2.493681000	-0.688202000	-0.997240000
1	-2.914733000	-1.244705000	-1.821993000
1	-1.253886000	1.262152000	2.147482000

Table S58. Predicted frequencies (cm^{-1}) and IR intensities (km/mol) of isomer 5c-doublet of $Pt^{+}(C_2H_2)_5$

Frequency	Intensity	Frequency	Intensity	Frequency	Intensity
52.2079	0.0594	905.2976	1.1721	1343.1837	3.3203
75.1374	2.1192	912.4212	0.9484	1350.7812	3.0812
76.6041	0.0174	931.2917	0.3006	1381.5057	0.0428
107.827	5.0882	933.7911	11.8357	1481.9117	22.9566
114.2889	3.2723	947.0599	3.1388	1497.3662	15.7199
201.2279	2.3458	970.2358	1.3488	1558.4921	0.5861
271.4976	1.6388	979.8496	2.7917	1592.8968	10.7116
306.8907	7.6146	997.6474	0.7328	3193.4324	0.069
395.4678	11.4209	1010.9972	3.0709	3198.9249	0.2559
412.2652	3.9622	1019.2883	0.0025	3202.1385	0.0002
437.6757	0.2309	1022.4138	0.9645	3209.9693	1.6078
553.9587	3.3821	1038.4888	1.0119	3213.0621	2.576
601.1004	2.6269	1049.8049	1.41	3217.9819	0.4927
610.7641	0.2064	1126.1067	0.2601	3230.8014	0.8386
642.4211	54.4039	1178.6925	0.2076	3238.4645	4.5832
667.8508	0.3995	1189.9205	0.0668	3265.3181	20.8664
755.7001	66.4708	1191.8718	0.4157	3273.9841	6.2934
763.7843	32.2473	1201.7414	0.0286		
810.4886	0.0084	1243.9722	0.404		
891.9989	8.9778	1333.523	20.8773		

Table S59. Cartesian coordinates for the optimized geometry of isomer 5d-doublet of $Pt^+(C_2H_2)_5$ followed by its predicted frequencies (cm^{-1}) and IR intensities (km/mol).

Z	x	y	z
6	-2.013110000	-0.436027000	-1.529132000
6	-1.833347000	0.882959000	-1.535533000
6	-0.823537000	1.552689000	-0.697226000
6	-2.013112000	-0.436014000	1.529133000
6	-1.833349000	0.882973000	1.535524000
6	-0.823539000	1.552696000	0.697214000
1	-2.397052000	1.517868000	2.209152000
1	-2.741326000	-0.897835000	-2.184954000
1	-2.397049000	1.517850000	-2.209166000
1	-0.211511000	2.298215000	-1.192554000
1	-2.741330000	-0.897817000	2.184958000
1	-0.211513000	2.298226000	1.192536000
78	0.592215000	-0.202861000	0.000001000
1	-0.801620000	-2.227618000	-1.194645000
6	-1.188291000	-1.342389000	-0.698942000
6	-1.188292000	-1.342383000	0.698952000
1	-0.801621000	-2.227607000	1.194664000
6	2.750040000	-0.131154000	-0.000001000
6	2.354470000	1.039568000	-0.000004000
1	2.411114000	2.107693000	-0.000003000
1	3.371564000	-1.003339000	0.000007000

Table S60. Predicted frequencies (cm^{-1}) and IR intensities (km/mol) of isomer 5d-doublet of $Pt^+(C_2H_2)_5$

Frequency	Intensity	Frequency	Intensity	Frequency	Intensity
72.2165	0.2258	739.2155	10.9493	1368.065	0.0005
93.654	2.8113	785.3019	30.617	1402.2504	22.8641
122.8006	3.8791	808.0304	61.4375	1420.9984	0.5377
169.5613	1.1465	811.2815	17.3639	1449.5165	0.0005
193.2084	9.4584	821.2296	2.1252	1495.2045	1.5548
213.6806	1.5357	837.8389	4.2885	1508.6442	16.168
234.457	0.0875	904.756	1.0119	1684.5801	1.2034
270.5508	4.3238	918.3826	1.6289	1688.845	7.0872
290.9852	6.9824	961.2991	2.3713	1864.5822	15.7707
335.5613	24.3262	975.1193	0.1635	3123.6111	2.1484
395.4621	15.4889	980.3746	0.2838	3139.2921	0.5476
402.5778	1.4911	998.6424	3.3065	3144.0179	0.7624
418.1794	2.0726	1018.5937	3.0083	3155.5979	1.7717
437.1011	0.6205	1025.582	8.9961	3159.8803	0.4159
613.1613	10.8619	1043.2051	3.6374	3162.2788	0.3353
656.8821	2.6811	1191.2763	1.4756	3177.3952	0.8567
689.9089	3.0899	1205.3874	0.1054	3177.9318	0.9109
718.8204	11.2922	1210.5347	4.0185	3313.3793	112.0724
732.4076	52.3822	1221.5006	5.9671	3388.5312	79.0878

Table S61. Cartesian coordinates for the optimized geometry of isomer 5e-doublet of $Pt^+(C_2H_2)_5$ followed by its predicted frequencies (cm^{-1}) and IR intensities (km/mol).

Z	x	y	z
6	0.541040000	1.693078000	-0.808672000
6	-0.031597000	2.522537000	0.172915000
6	-1.045283000	2.173517000	1.099398000
6	-1.315787000	0.935624000	1.581008000
6	-0.592411000	-0.355562000	1.384993000
6	-1.338356000	-1.530612000	0.904466000
6	-2.184662000	-1.541172000	-0.156443000
6	-2.423300000	-0.488270000	-1.120439000
6	-1.556451000	0.384210000	-1.659957000
6	-0.092323000	0.561120000	-1.489900000
1	0.417506000	0.327629000	-2.427248000
1	-1.938107000	1.008963000	-2.464181000
1	-3.418645000	-0.486475000	-1.550073000
1	-2.752650000	-2.449459000	-0.323655000
1	-1.218551000	-2.444949000	1.471267000
1	-0.066638000	-0.590122000	2.320616000
1	-2.102739000	0.855340000	2.325782000
1	-1.593958000	2.996467000	1.541474000
1	0.358952000	3.531352000	0.237339000
1	1.426709000	2.087108000	-1.293829000
78	0.911832000	-0.396957000	0.009209000

Table S62. Predicted frequencies (cm^{-1}) and IR intensities (km/mol) of isomer 5e-doublet of $Pt^{+}(C_2H_2)_5$

Frequency	Intensity	Frequency	Intensity	Frequency	Intensity
96.5217	0.6499	830.2797	18.168	1379.5438	6.3683
136.8195	0.3655	859.466	2.0335	1414.0658	7.4811
149.1606	2.5414	879.1688	8.1843	1424.2396	9.8359
167.6119	1.1348	920.8559	12.4427	1448.8378	9.7288
210.0735	1.7719	930.07	6.0414	1467.268	1.1681
252.4178	8.6112	969.4489	2.7075	1488.9253	5.2529
280.3547	2.2419	981.6594	2.3246	1562.8097	24.2291
332.1807	1.0473	1000.7916	0.5945	1579.5511	36.019
360.6503	3.5766	1009.1459	1.7501	1660.1394	11.5696
422.4634	3.9185	1027.5859	1.7213	2974.1629	0.4912
481.9686	2.3667	1049.1796	17.0104	3046.3689	0.1357
507.0529	1.3405	1065.7946	18.0341	3115.9992	1.6453
517.4155	13.8093	1091.4338	13.8897	3130.8018	2.3377
630.0365	13.6177	1132.1978	3.0164	3149.4036	0.9546
648.5871	21.5395	1239.63	3.0644	3151.3374	0.2559
721.0285	33.316	1254.3204	5.1949	3160.8028	0.3077
727.2383	14.6076	1262.4502	8.9118	3162.8534	0.7249
775.514	20.5318	1281.0548	0.963	3172.9664	1.106
818.9687	1.5057	1339.2409	5.5965	3177.5348	0.5924

Table S63. Cartesian coordinates for the optimized geometry of isomer 5f-doublet of $Pt^+(C_2H_2)_5$ followed by its predicted frequencies (cm^{-1}) and IR intensities (km/mol).

Z	x	y	z
6	-1.553636000	-0.703563000	1.103010000
6	-2.225706000	-1.397382000	0.078119000
6	-1.553720000	0.703539000	1.103043000
1	-2.246801000	-2.478254000	0.088373000
1	-1.175185000	1.238403000	1.962479000
6	-2.889229000	-0.698749000	-0.916883000
6	-2.225868000	1.397327000	0.078189000
1	-3.420601000	-1.236975000	-1.689163000
1	-2.247090000	2.478197000	0.088494000
6	-2.889311000	0.698662000	-0.916847000
1	-3.420747000	1.236865000	-1.689099000
1	-1.175069000	-1.238421000	1.962435000
78	0.632602000	0.000009000	0.018727000
6	1.909720000	-1.528552000	-0.638624000
6	1.196718000	-2.017027000	0.266387000
1	0.854845000	-2.786719000	0.927687000
1	2.690604000	-1.521436000	-1.371781000
1	2.690417000	1.521528000	-1.371986000
6	1.909621000	1.528599000	-0.638733000
1	0.854891000	2.786706000	0.927729000
6	1.196704000	2.017043000	0.266364000

Table S64. Predicted frequencies (cm^{-1}) and IR intensities (km/mol) of isomer 5f-doublet of $Pt^{+}(C_2H_2)_5$

Frequency	Intensity	Frequency	Intensity	Frequency	Intensity
26.2065	0.1712	730.1471	2.2523	1392.1306	0.0693
41.4592	0.9073	737.9972	104.2331	1503.6287	16.5641
65.9694	1.4918	750.4896	67.6498	1509.4959	14.73
66.4683	1.2829	803.7344	0.5175	1601.5449	9.0907
101.7027	0.599	822.2083	11.2632	1612.7927	2.7132
115.5981	2.0107	833.4853	7.2923	1783.0662	2.5787
134.4156	4.0233	875.1034	47.8458	1791.6179	0.4687
170.6526	5.094	889.2681	0.4595	3181.112	0.1418
198.1502	9.3511	912.8336	3.5415	3188.2564	0.1205
361.0943	8.2973	990.8842	14.3964	3189.8074	0.2612
400.2718	0.2818	997.3637	0.0001	3200.5959	0.2455
412.4335	0.7075	1015.4526	0.2598	3200.9275	0.0174
414.1849	0.0005	1034.2427	0.0938	3208.4548	0.8581
463.2309	0.145	1043.9498	0.183	3303.8141	21.6777
501.1261	8.0789	1053.6325	1.7533	3304.2315	122.1531
613.4174	0.3012	1054.3075	0.5122	3368.2373	58.2259
614.2545	1.143	1185.5109	0.3904	3368.7547	30.3341
698.2135	0.1099	1202.2203	3.9616		
720.7917	0	1204.4397	0.8971		
727.9164	55.0615	1353.5202	10.6983		

Table S65. Cartesian coordinates for the optimized geometry of isomer 5g-doublet of $Pt^{+}(C_2H_2)_5$ followed by its predicted frequencies (cm^{-1}) and IR intensities (km/mol).

Z	x	y	z
6	-1.006024000	1.460544000	-1.497715000
6	-0.431183000	2.343582000	-0.685962000
6	0.377807000	1.996469000	0.546836000
6	-2.430640000	-0.164246000	0.741932000
6	-1.608798000	0.567784000	1.542792000
6	-0.159464000	0.746827000	1.279068000
1	-1.975952000	0.909709000	2.504697000
1	-1.475560000	1.828245000	-2.405392000
1	-0.475767000	3.392006000	-0.949584000
1	0.355172000	2.850421000	1.230167000
1	-3.409661000	-0.445112000	1.110640000
1	0.418847000	0.594733000	2.191117000
78	0.403300000	-0.755775000	-0.005164000
1	-0.916393000	-0.524937000	-2.324164000
6	-1.076868000	-0.017799000	-1.374622000
6	-2.018589000	-0.685216000	-0.522318000
1	-2.488316000	-1.590072000	-0.888271000
6	1.805259000	1.643184000	0.170129000
6	2.041155000	0.372427000	-0.155501000
1	2.577317000	2.405019000	0.167286000
1	2.976982000	-0.050911000	-0.501538000

Table S66. Predicted frequencies (cm^{-1}) and IR intensities (km/mol) of isomer 5g-doublet of $Pt^{+}(C_2H_2)_5$

Frequency	Intensity	Frequency	Intensity	Frequency	Intensity
130.1327	0.7098	828.847	13.8707	1308.1359	3.43
156.6769	0.7176	848.6756	14.747	1336.8155	1.8707
185.7258	2.9326	862.3437	9.795	1387.7515	0.7499
232.8431	1.3536	910.6885	13.7332	1400.0037	2.8589
248.0127	1.1676	914.9915	2.9158	1407.6686	18.6678
282.8172	1.9525	952.4806	10.3912	1455.802	2.8979
300.8796	8.1448	980.3415	1.6289	1543.8408	24.154
375.2356	0.263	987.0047	3.2772	1560.6952	6.6017
423.667	9.8045	1006.544	3.4636	1692.7259	0.779
441.2115	3.008	1029.9678	10.2552	3031.6283	0.5183
503.3332	1.0574	1046.9913	2.2909	3064.7438	0.9349
543.0758	0.6098	1061.5587	5.085	3099.3473	0.3439
548.5406	4.8263	1086.6241	18.3249	3133.7753	2.4339
638.108	7.9985	1107.2811	13.5173	3149.1835	2.9791
684.9649	8.0878	1181.5062	0.9975	3151.9635	1.3394
714.0448	12.6743	1210.5736	2.8894	3168.1202	1.0065
718.2005	51.5773	1239.6624	10.0638	3170.8568	7.3074
760.4371	43.7535	1256.1807	22.7075	3178.9889	2.8606
810.6178	18.9385	1265.8284	30.426	3184.3346	0.2081

Table S67. Cartesian coordinates for the optimized geometry of isomer 5h-doublet of $Pt^{+}(C_2H_2)_5$ followed by its predicted frequencies (cm^{-1}) and IR intensities (km/mol).

Z	x	y	z
6	-2.270567000	2.073518000	0.396947000
6	-1.126960000	2.334360000	-0.409828000
6	0.027876000	1.643173000	-0.609996000
6	-0.710683000	-2.774396000	-0.410692000
6	-0.094330000	-2.342434000	0.822163000
6	0.459416000	-1.153972000	1.162426000
1	0.016140000	-3.125293000	1.573152000
1	-2.790019000	2.969092000	0.721021000
1	-1.124245000	3.323495000	-0.863289000
1	0.749085000	2.092994000	-1.321237000
1	-0.483365000	-3.789505000	-0.717500000
1	0.787486000	-0.994093000	2.188263000
78	1.130321000	0.224092000	-0.018751000
1	-3.496702000	0.952190000	1.673491000
6	-2.784758000	0.884105000	0.858453000
6	-1.526246000	-2.032704000	-1.209433000
1	-1.756601000	-2.412763000	-2.198254000
1	-2.639891000	-1.219203000	1.116155000
6	-2.463674000	-0.412673000	0.413267000
6	-2.048792000	-0.758105000	-0.855442000
1	-2.194604000	-0.041317000	-1.656432000

Table S68. Predicted frequencies (cm^{-1}) and IR intensities (km/mol) of isomer 5h-doublet of $Pt^{+}(C_2H_2)_5$

Frequency	Intensity	Frequency	Intensity	Frequency	Intensity
72.9596	0.6572	817.0429	6.6621	1361.2339	6.9605
103.3673	0.1024	832.0416	9.3774	1366.0723	2.2667
115.837	0.7292	852.1332	5.707	1454.8906	19.5059
138.9307	0.6015	903.8642	13.7995	1476.2194	75.3674
195.5373	0.1329	912.7281	8.087	1502.7221	37.9032
215.7157	0.4816	925.2773	7.8915	1538.2196	13.4474
243.5987	0.2675	944.5829	8.7012	1557.2301	14.0562
287.5729	0.5415	955.1329	4.3811	1581.5612	82.6803
340.9258	0.6428	970.6353	19.8714	1599.5598	0.217
383.4469	0.1068	1002.1215	12.866	2912.3234	4.9229
397.4801	21.5323	1014.6917	6.6928	3084.8916	2.6425
466.9903	4.3118	1051.766	0.835	3108.2662	5.5052
501.5516	8.8337	1112.3672	1.0625	3109.3373	2.847
544.123	9.1175	1186.157	13.4118	3144.0168	0.895
595.525	23.1105	1202.625	1.6988	3148.3422	0.742
641.5345	21.4537	1227.391	2.3459	3152.8323	0.0678
671.3841	23.7392	1260.3661	6.98	3157.5547	1.163
687.4713	101.6596	1305.2838	6.7341	3165.1028	0.683
784.657	23.1079	1310.5182	24.2963	3167.3299	1.0048

Table S69. Cartesian coordinates for the optimized geometry of isomer 5i-doublet of $Pt^+(C_2H_2)_5$ followed by its predicted frequencies (cm^{-1}) and IR intensities (km/mol).

Z	x	y	z
6	-2.290019000	1.717137000	-0.225219000
6	-1.456700000	1.855995000	0.904322000
6	-0.288614000	1.218964000	1.205485000
6	-2.289890000	-1.717309000	-0.224837000
6	-1.456615000	-1.855786000	0.904786000
6	-0.288592000	-1.218590000	1.205817000
1	-1.707419000	-2.696637000	1.545631000
1	-2.857832000	2.618338000	-0.438516000
1	-1.707536000	2.697012000	1.544936000
1	0.200547000	1.492676000	2.136747000
1	-2.857596000	-2.618622000	-0.437946000
1	0.200549000	-1.491987000	2.137182000
78	0.798268000	0.000045000	0.097754000
1	-3.060223000	1.088971000	-2.024934000
6	-2.553087000	0.702733000	-1.147150000
6	-2.553031000	-0.703142000	-1.147002000
1	-3.060128000	-1.089607000	-2.024707000
6	1.786001000	-0.000326000	-1.757238000
6	2.660668000	-0.000181000	-0.873523000
1	3.624078000	-0.000130000	-0.405408000
1	1.339921000	-0.000467000	-2.730455000

Table S70. Predicted frequencies (cm^{-1}) and IR intensities (km/mol) of isomer 5i-doublet of $Pt^+(C_2H_2)_5$

Frequency	Intensity	Frequency	Intensity	Frequency	Intensity
58.7761	2.2131	736.3049	1.2685	1380.9717	16.8659
67.6534	0.9581	748.4807	1.0416	1392.7879	24.0764
98.8589	2.9286	779.7191	61.5342	1480.3455	28.6816
112.5939	2.2459	795.4161	3.3238	1510.3317	20.0512
122.7122	8.303	811.432	15.2023	1521.7865	85.3654
156.1822	0.2111	814.5899	10.8727	1529.5409	25.4472
216.1037	2.122	840.9334	0.0473	1565.0765	76.4766
223.6255	0.1921	876.4884	0.5251	1575.4418	17.7873
323.7554	0.3014	925.7138	2.074	1833.6314	2.5248
342.423	3.941	943.8159	0.0095	3114.4154	0.2636
375.6657	2.8884	978.3045	1.9757	3115.3142	1.09
468.6095	4.7876	985.9179	9.2776	3124.0595	0.2595
470.6325	0.0448	1005.0194	4.7364	3127.3808	0.3406
497.6521	6.368	1030.6675	10.4264	3133.7848	0.1512
507.4319	13.9836	1112.3934	0.6563	3137.4219	0.2178
584.2527	10.7693	1197.8797	0.2109	3143.0241	0.0605
642.3098	0.2302	1240.3437	6.1875	3154.4655	0.415
670.069	55.9217	1308.8525	0.02	3309.0714	101.8404
723.5567	15.9181	1315.9047	16.5831	3380.9757	62.783

Table S71. Cartesian coordinates for the optimized geometry of isomer 5j-doublet of $Pt^+(C_2H_2)_5$ followed by its predicted frequencies (cm^{-1}) and IR intensities (km/mol).

Z	x	y	z
6	-2.847201000	-0.947232000	-0.690570000
6	-2.161394000	-0.069821000	-1.566889000
6	-1.055291000	0.678990000	-1.309103000
6	-2.847139000	-0.947478000	0.690401000
6	-2.161256000	-0.070380000	1.566973000
6	-1.055180000	0.678529000	1.309364000
1	-2.580048000	0.016157000	2.565979000
1	-3.563311000	-1.610035000	-1.160721000
1	-2.580271000	0.017071000	-2.565828000
1	-0.724102000	1.402456000	-2.056451000
1	-3.563208000	-1.610449000	1.160380000
1	-0.723931000	1.401739000	2.056933000
78	0.414072000	0.480525000	0.000040000
6	2.469658000	0.184442000	0.000046000
6	2.850687000	-1.098991000	-0.000096000
1	3.886654000	-1.420059000	-0.000077000
1	3.151899000	1.028934000	0.000155000
1	1.923658000	-3.148367000	-0.000443000
6	1.758960000	-2.076008000	-0.000287000
6	0.532852000	-1.581505000	-0.000283000
1	-0.433153000	-2.061682000	-0.000418000

Table S72. Predicted frequencies (cm^{-1}) and IR intensities (km/mol) of isomer 5j-doublet of $Pt^{+}(C_2H_2)_5$

Frequency	Intensity	Frequency	Intensity	Frequency	Intensity
30.2693	1.2439	712.4871	0.2055	1359.9207	12.0135
86.2458	0.3558	797.3078	15.1545	1484.0818	17.9242
94.2935	0	851.9622	0.1932	1510.859	20.0563
127.2903	8.3792	871.2125	1.162	1556.269	13.4334
171.7167	0.065	887.989	2.5277	1576.7364	4.5182
176.9843	14.8784	932.2628	0.1851	1589.8651	34.8449
254.6225	0.0201	944.7486	2.1542	1644.2959	35.3652
334.9754	3.7904	960.0071	3.5277	3076.4925	5.4528
340.5395	5.3658	975.157	44.0971	3076.5105	7.6909
370.3988	0.631	984.3536	4.6932	3128.84	2.0223
423.9201	0.0796	1034.2849	0.0044	3129.0352	1.049
447.3293	37.5345	1055.6183	0.2298	3132.121	6.7284
509.6694	0.3684	1084.82	58.4333	3152.6474	2.3813
526.2626	2.5842	1097.5124	16.8198	3164.9742	4.1708
586.6622	8.3783	1184.4072	7.8542	3165.6339	0.5192
586.7582	9.1583	1218.5785	7.0396	3180.2312	0.1003
631.1973	76.8851	1231.7286	68.2464	3220.6774	3.0291
657.8146	13.734	1277.045	17.6478		
678.3625	56.7315	1307.0588	2.0534		
708.0617	1.6051	1311.9324	67.0157		

Table S73. Cartesian coordinates for the optimized geometry of isomer 5k-doublet of $Pt^{+}(C_2H_2)_5$ followed by its predicted frequencies (cm^{-1}) and IR intensities (km/mol).

Z	x	y	z
6	-3.127016000	-0.696981000	0.403447000
6	-2.195918000	-1.609059000	-0.116351000
6	-0.882279000	-1.421768000	-0.478956000
6	-3.126990000	0.697052000	0.403489000
6	-2.195861000	1.609126000	-0.116255000
6	-0.882229000	1.421808000	-0.478881000
1	-2.580860000	2.617316000	-0.242150000
1	-4.046233000	-1.144153000	0.763342000
1	-2.580957000	-2.617224000	-0.242317000
1	-0.429230000	-2.278992000	-0.972903000
1	-4.046190000	1.144236000	0.763414000
1	-0.429156000	2.279051000	-0.972772000
78	0.414952000	-0.000011000	-0.098393000
6	2.248601000	-1.546546000	-0.158995000
6	1.732597000	-1.708321000	0.925826000
1	1.395218000	-1.990582000	1.897389000
1	2.821097000	-1.532934000	-1.058406000
1	1.395676000	1.990203000	1.897565000
6	1.732751000	1.708256000	0.925806000
6	2.248610000	1.546554000	-0.159093000
1	2.820773000	1.533232000	-1.058721000

Table S74. Predicted frequencies (cm^{-1}) and IR intensities (km/mol) of isomer 5k-doublet of $Pt^{+}(C_2H_2)_5$

Frequency	Intensity	Frequency	Intensity	Frequency	Intensity
46.9175	0.001	670.63	0.2502	1306.2328	3.6946
63.5501	2.0955	677.4739	7.9131	1356.0063	0.5654
86.0497	0.3593	705.4609	15.3775	1399.0638	15.5391
115.9068	0.207	715.075	1.7204	1484.0028	38.8712
125.8277	2.4147	733.7085	17.4025	1520.5291	15.974
151.5663	2.8482	748.951	0.0086	1565.8637	20.1982
179.2857	1.5305	765.6708	62.2963	1573.1102	40.9504
180.4537	0.0526	776.6394	72.3048	1980.458	0.8306
222.1727	1.4435	795.0063	31.646	1984.2992	0.0056
228.4492	2.7187	811.7722	3.6624	3102.4292	1.7389
230.5708	1.8899	883.5802	0.6905	3103.1353	0.5795
284.5228	1.1124	887.1524	1.7785	3128.2209	2.4936
291.3495	8.0756	920.2003	4.4592	3128.6729	0.0913
363.3768	0.0787	979.3862	13.2544	3157.2941	0.1172
378.5559	7.7907	1010.011	2.1846	3172.6431	1.3102
516.3817	3.2357	1041.2971	1.3802	3361.2668	4.1848
536.3758	1.1828	1077.8356	0.0363	3363.0914	274.6636
594.5544	0.0609	1222.0991	3.3058	3452.5283	29.309
662.5014	37.6239	1273.006	8.2513	3454.7077	56.2384

Table S75. Cartesian coordinates for the optimized geometry of isomer 5l-doublet of $Pt^+(C_2H_2)_5$ followed by its predicted frequencies (cm^{-1}) and IR intensities (km/mol).

Z	x	y	z
6	2.518227000	-0.150741000	-0.846922000
6	2.518258000	-0.150234000	0.846958000
6	1.971650000	-1.548109000	0.667304000
6	1.971620000	-1.548506000	-0.666411000
1	3.554682000	-0.112903000	-1.179544000
1	3.554724000	-0.112190000	1.179520000
1	1.692351000	-2.273451000	1.418366000
1	1.692287000	-2.274295000	-1.417028000
78	-0.751046000	0.136301000	-0.000024000
6	1.682948000	0.822601000	1.566838000
1	2.178238000	1.452727000	2.301958000
6	0.370544000	1.041807000	1.355602000
1	-0.172688000	1.783185000	1.932533000
6	-2.762276000	-0.475399000	0.000102000
6	-2.069066000	-1.510576000	0.000411000
1	-1.834739000	-2.555570000	0.000674000
1	-3.620503000	0.166566000	-0.000069000
1	-0.172751000	1.782036000	-1.933568000
6	0.370503000	1.041006000	-1.356211000
6	1.682898000	0.821666000	-1.567357000
1	2.178162000	1.451340000	-2.302881000

Table S76. Predicted frequencies (cm^{-1}) and IR intensities (km/mol) of isomer 5l-doublet of $Pt^+(C_2H_2)_5$

Frequency	Intensity	Frequency	Intensity	Frequency	Intensity
60.0759	4.2809	740.3411	4.0502	1276.9232	4.1821
72.6697	1.855	758.733	22.8781	1296.4385	28.4264
86.2704	0.2039	781.687	61.5191	1315.9521	9.7662
118.8702	2.4446	803.2795	16.1043	1348.029	4.0525
195.7155	0.2209	811.8418	38.1282	1370.8764	0.2653
210.6519	6.1894	816.7704	4.1003	1498.2384	90.7109
265.9415	0.5218	856.1238	6.7642	1563.3963	8.6773
268.029	3.584	867.937	10.763	1612.0649	23.8986
351.1888	3.6611	921.164	6.2902	1819.0295	0.1674
390.1176	0.388	939.8135	2.6304	3078.8278	2.2227
410.1504	15.2595	956.3699	16.2992	3086.8412	3.7793
438.4161	4.9022	976.1687	0.246	3116.5736	0.0423
476.3982	2.6668	1042.6368	6.6384	3117.0418	1.9076
503.7933	4.2898	1045.3622	10.0557	3150.2096	2.1651
520.7507	7.5469	1112.2319	49.2991	3150.7941	1.2091
562.1738	2.9801	1124.9608	3.19	3188.3161	0.0684
669.6144	5.3956	1165.3782	18.0339	3216.13	3.1217
680.913	1.9709	1183.287	5.9565	3304.613	116.1205
725.1747	25.4151	1185.3661	6.8546	3375.7084	69.1747

Table S77. Cartesian coordinates for the optimized geometry of isomer 5m-doublet of $Pt^+(C_2H_2)_5$ followed by its predicted frequencies (cm^{-1}) and IR intensities (km/mol).

Z	x	y	z
6	-1.747507000	-0.882983000	1.018103000
6	-2.619548000	-0.105291000	0.000010000
6	-1.747326000	-0.882802000	-1.018081000
6	-1.194315000	-1.704176000	0.000008000
1	-1.769151000	-0.934546000	2.096298000
1	-3.663726000	-0.413612000	-0.000093000
1	-1.768963000	-0.934331000	-2.096278000
1	-0.667849000	-2.646163000	-0.000014000
78	0.195779000	0.044999000	-0.000047000
6	-2.354315000	1.358589000	0.000103000
1	-3.150962000	2.093388000	0.000256000
6	-1.040854000	1.658271000	0.000000000
1	-0.678828000	2.678467000	-0.000016000
1	2.327569000	-1.492961000	1.464374000
6	2.222956000	-0.737351000	0.703656000
6	2.078540000	0.735395000	0.725578000
1	2.189527000	1.494570000	1.480865000
6	2.222996000	-0.736291000	-0.704568000
6	2.078439000	0.736510000	-0.724289000
1	2.189577000	1.496826000	-1.478404000
1	2.327664000	-1.490755000	-1.466418000

Table S78. Predicted frequencies (cm^{-1}) and IR intensities (km/mol) of isomer 5m-doublet of $Pt^{+}(C_2H_2)_5$

Frequency	Intensity	Frequency	Intensity	Frequency	Intensity
35.9673	1.2106	854.8042	3.3632	1303.0009	4.0584
79.6607	0.4622	892.469	1.9492	1324.3817	21.2772
107.3248	10.6172	897.6674	7.7716	1336.8452	6.628
121.9834	4.4781	899.0086	10.918	1342.5757	2.7439
200.839	1.5137	901.3775	17.525	1385.8507	6.2775
232.6468	2.4717	919.9205	3.7074	1411.3033	5.57
294.1361	4.9795	950.2565	0.5786	1538.9488	57.23
341.3048	0.8802	954.7188	11.2475	3100.2026	2.2162
352.7317	5.7418	961.6789	3.2289	3159.4968	0.4574
428.1615	4.5092	965.1616	1.1113	3182.3952	3.1121
434.0679	1.7978	986.7304	1.2054	3210.5603	0.8876
468.6655	4.9887	991.648	7.8056	3222.4752	6.2765
570.3177	24.4133	1023.4653	3.8156	3235.6995	3.4077
583.7136	0.5458	1118.8844	2.6267	3236.9521	0.1682
715.738	22.1773	1141.2994	12.9376	3253.1409	12.474
745.3479	3.4928	1150.9198	6.2946	3256.1449	10.3984
747.3552	7.1146	1170.7453	17.7566	3267.7448	13.9312
795.7264	2.0935	1201.5486	0.0884		
811.5805	42.8704	1207.0958	0.7496		
845.8324	14.2838	1273.8689	16.4803		

Table S79. Cartesian coordinates for the optimized geometry of isomer 5n-doublet of $Pt^{+}(C_2H_2)_5$ followed by its predicted frequencies (cm^{-1}) and IR intensities (km/mol).

Z	x	y	z
6	-2.782503000	-0.479058000	0.965385000
6	-3.076294000	0.303747000	-0.239302000
6	-1.499751000	-0.707919000	1.240859000
1	-4.092618000	0.559055000	-0.515607000
1	-1.053790000	-1.155210000	2.117291000
6	-2.018601000	0.693805000	-0.943587000
1	-1.993499000	1.322150000	-1.824482000
1	-3.564680000	-0.847184000	1.620495000
78	-0.261859000	-0.118845000	-0.222703000
6	3.253545000	-0.095248000	-0.533116000
6	2.510588000	-1.255808000	-0.554783000
1	2.520875000	-2.127744000	-1.192657000
1	4.060927000	0.295438000	-1.135553000
1	3.270021000	0.446490000	1.599716000
6	2.583345000	0.406286000	0.752078000
1	1.429266000	-1.572167000	1.412950000
6	1.783194000	-0.918011000	0.624425000
6	1.756014000	1.649931000	0.671716000
6	0.465113000	1.617427000	0.359294000
1	2.225509000	2.608738000	0.873183000
1	-0.224948000	2.449414000	0.357661000

Table S80. Predicted frequencies (cm^{-1}) and IR intensities (km/mol) of isomer 5n-doublet of $Pt^+(C_2H_2)_5$

Frequency	Intensity	Frequency	Intensity	Frequency	Intensity
42.9918	0.4531	757.1211	22.43	1233.2567	73.6376
51.7727	1.0567	788.5614	2.8411	1284.5551	31.9322
66.8484	0.2944	839.248	26.705	1319.9678	64.1206
89.9237	0.7577	871.0594	3.8412	1326.8167	12.6139
183.1987	0.1063	886.9761	26.9521	1345.3879	33.7394
218.8518	6.7068	897.2831	12.1337	1439.1057	32.0498
246.0994	5.4161	906.8949	5.8171	1588.4256	19.5989
266.0266	0.6935	933.9416	14.2705	1617.0432	1.3157
343.7991	0.953	942.5689	11.2347	1626.4282	5.0212
394.1953	1.341	954.0758	4.8096	3062.7111	0.7519
445.2339	5.3092	974.8875	8.592	3137.1933	5.9456
464.5388	8.4409	1019.2922	4.3937	3154.3013	0.2116
490.3047	0.9384	1025.0442	3.328	3158.3501	3.5701
573.0426	12.6336	1084.8095	40.2806	3165.043	5.2613
614.7005	10.5678	1102.5426	7.116	3185.9102	7.1843
640.3217	93.9851	1118.7979	6.8527	3199.5624	6.2342
680.0533	34.7071	1155.8995	5.2878	3203.7902	1.8489
726.5091	33.0825	1186.5077	7.7575	3206.2863	4.8992
745.034	2.1788	1216.5483	5.1002	3225.6564	0.4382

Table S81. Cartesian coordinates for the optimized geometry of isomer 5o-doublet of $Pt^+(C_2H_2)_5$ followed by its predicted frequencies (cm^{-1}) and IR intensities (km/mol).

Z	x	y	z
6	-1.935042000	-1.195242000	1.117259000
6	-2.239759000	-0.773496000	-0.312955000
6	-0.910391000	-1.528201000	-0.636540000
6	-0.790674000	-1.902947000	0.745940000
1	-2.454409000	-1.065984000	2.055842000
1	-3.136391000	-1.266194000	-0.692213000
1	-0.649369000	-2.094487000	-1.523584000
1	-0.155561000	-2.595006000	1.280558000
78	0.690455000	-0.179680000	-0.135962000
6	-2.194254000	0.706356000	-0.671836000
1	-2.700439000	0.889989000	-1.620413000
6	-2.455899000	1.732850000	0.417306000
6	-0.731001000	1.258669000	-0.600435000
1	-0.287704000	1.696478000	-1.497142000
1	-3.370456000	2.075224000	0.882352000
6	-1.176159000	2.138333000	0.501845000
1	-0.669296000	2.894218000	1.082797000
6	2.242661000	0.669966000	1.098411000
6	2.551870000	0.833864000	-0.092048000
1	3.140881000	1.141790000	-0.932637000
1	2.259162000	0.698058000	2.167784000

Table S82. Predicted frequencies (cm^{-1}) and IR intensities (km/mol) of isomer 5o-doublet of $Pt^{+}(C_2H_2)_5$

Frequency	Intensity	Frequency	Intensity	Frequency	Intensity
63.9767	0.8992	795.2764	12.2633	1217.3638	0.1957
71.053	1.4479	799.8011	0.6119	1223.4494	1.7654
108.7871	3.4469	822.4871	39.7513	1286.2387	0.4405
120.3545	1.9825	855.5926	6.3098	1325.5205	24.1398
141.2744	0.6461	865.1129	2.336	1337.5463	4.3533
197.1681	2.6094	883.2236	7.6497	1366.2288	7.5343
258.353	2.8704	897.4251	3.285	1399.8145	12.8549
267.8236	2.6161	919.796	6.5502	1576.6254	20.4178
308.3243	4.0239	931.5414	13.9688	1835.4083	6.9192
334.5553	5.4241	939.8911	40.69	3055.4591	6.8751
397.5113	8.0441	985.6045	9.4266	3061.5173	1.6606
438.7026	6.3261	1009.766	14.4707	3073.1837	6.9385
443.0591	7.0964	1018.7593	0.6397	3154.3761	3.0044
531.7602	9.195	1057.1692	82.597	3190.1685	0.0138
647.5456	29.8373	1079.3698	32.1844	3199.3408	2.6506
702.4081	1.1808	1138.7706	15.0005	3220.5201	1.7688
734.4108	27.9611	1154.1294	13.1251	3222.6099	1.1157
743.8885	89.186	1165.8909	2.8422	3307.8524	109.5362
790.9438	9.5187	1190.843	2.9919	3383.0728	67.3742

Table S83. Cartesian coordinates for the optimized geometry of isomer 5p-doublet of $Pt^+(C_2H_2)_5$ followed by its predicted frequencies (cm^{-1}) and IR intensities (km/mol).

Z	x	y	z
6	2.607760000	-0.760145000	-0.706121000
6	2.607766000	-0.760179000	0.706062000
6	1.378705000	-0.532237000	-1.284709000
1	3.500890000	-0.959708000	1.286581000
1	1.216220000	-0.705445000	-2.342677000
6	1.378717000	-0.532293000	1.284670000
1	1.216242000	-0.705532000	2.342634000
1	3.500882000	-0.959637000	-1.286656000
78	-0.115116000	0.035418000	-0.000001000
6	-1.989249000	-0.545471000	1.033244000
6	-1.541989000	-1.497525000	0.000029000
1	-1.321669000	-2.551626000	0.000045000
1	-2.104162000	-0.585474000	2.102046000
1	-2.858673000	1.336949000	-0.000017000
6	-2.415042000	0.353442000	-0.000002000
1	-2.104148000	-0.585535000	-2.102020000
6	-1.989258000	-0.545506000	-1.033216000
6	0.541361000	2.241760000	0.609897000
6	0.541122000	2.241806000	-0.609837000
1	0.607301000	2.485657000	1.645869000
1	0.606774000	2.485832000	-1.645798000

Table S84. Predicted frequencies (cm^{-1}) and IR intensities (km/mol) of isomer 5p-doublet of $Pt^+(C_2H_2)_5$

Frequency	Intensity	Frequency	Intensity	Frequency	Intensity
61.9362	0.1897	735.267	4.2915	1201.0812	0.0021
77.3845	0.041	744.5494	62.6896	1245.9995	2.8082
94.1727	2.6878	755.6004	0.0086	1263.7505	6.8741
122.2911	0.6583	776.6686	14.9909	1269.66	88.5345
125.1776	2.343	777.3119	0.2522	1362.1812	17.0911
135.5541	5.7415	812.9491	0.0895	1370.9265	4.0712
211.4381	7.5608	818.4592	31.8803	1483.2769	28.8007
275.9401	7.3462	894.3908	22.3839	1491.2278	44.5873
283.8197	3.3317	910.7609	17.6919	1938.6136	3.1166
313.4614	3.8289	931.2566	6.9425	3150.1033	0.0449
349.8508	11.2726	944.1229	10.23	3156.3557	1.643
351.9114	3.1991	961.7288	14.5944	3170.2374	0.2793
435.7289	10.1445	971.9737	9.0313	3180.4385	2.1326
436.0253	5.7449	977.0981	9.8053	3229.636	0.0608
466.9368	0.0068	992.6303	0.6526	3248.1961	6.7886
605.3575	1.8925	1041.8123	4.2171	3265.2613	10.7451
616.42	2.1202	1113.2966	2.2974	3271.9987	11.2653
678.6209	0.5169	1122.7553	1.8072	3362.0736	126.1658
702.896	59.0015	1189.7544	0.0062	3445.8767	42.3035

Table S85. Cartesian coordinates for the optimized geometry of isomer 5q-doublet of $Pt^{+}(C_2H_2)_5$ followed by its predicted frequencies (cm^{-1}) and IR intensities (km/mol).

Z	x	y	z
6	-1.795955000	-0.000002000	-0.790651000
6	-2.271145000	-1.179797000	0.016137000
6	-1.696730000	-0.000001000	0.770740000
6	-2.271148000	1.179793000	0.016137000
1	-1.191503000	-0.000001000	-1.692721000
1	-3.336676000	-1.367859000	0.084052000
1	-1.072223000	0.000000000	1.667316000
1	-3.336679000	1.367852000	0.084051000
78	0.501907000	0.000001000	0.032342000
6	-1.313702000	-2.315500000	-0.041616000
1	-1.682719000	-3.335458000	-0.051715000
6	-0.010920000	-2.042325000	-0.078912000
1	0.787739000	-2.766447000	-0.184782000
6	2.469731000	-0.000002000	-0.680257000
6	2.518845000	0.000002000	0.567042000
1	2.937225000	0.000005000	1.553871000
1	2.815011000	-0.000003000	-1.694784000
1	0.787733000	2.766449000	-0.184783000
6	-0.010925000	2.042325000	-0.078913000
6	-1.313707000	2.315498000	-0.041617000
1	-1.682726000	3.335455000	-0.051714000

Table S86. Predicted frequencies (cm^{-1}) and IR intensities (km/mol) of isomer 5q-doublet of $Pt^+(C_2H_2)_5$

Frequency	Intensity	Frequency	Intensity	Frequency	Intensity
50.9235	7.5382	746.3188	26.2567	1257.2123	81.3076
97.095	2.2982	747.9847	2.4652	1276.5026	1.7086
112.7518	0.0209	798.9583	30.0386	1346.7177	0.5748
125.7686	4.4017	833.2466	9.6238	1377.7352	8.8607
217.9153	0.2196	834.7239	0.8656	1589.8332	1.3508
269.701	2.1927	846.2462	7.5219	1600.22	2.7652
276.5665	1.2029	862.4907	14.723	1805.1673	0.1649
287.2017	4.0271	869.4276	4.7563	3053.113	20.1224
359.0491	4.6288	914.1183	10.2956	3131.1651	10.0467
371.2835	1.1408	951.9524	1.4963	3143.9609	4.4018
417.4559	8.6496	956.0942	22.7397	3144.5215	2.3391
455.0603	0.7315	978.9808	6.9081	3152.816	0.0691
465.525	4.6413	1009.6853	0.1847	3152.967	0.3353
469.9682	1.8986	1024.3983	9.2188	3172.1811	16.4199
521.4105	3.7049	1071.9549	6.9665	3173.705	0.382
684.4097	63.9894	1085.71	33.0545	3299.4234	113.8247
700.6618	18.9472	1095.9691	1.0119	3368.4177	62.9456
707.4691	3.7819	1130.4389	21.4238		
729.5581	0.8486	1193.8169	13.9219		
737.401	21.0393	1198.412	7.0872		

Table S87. Cartesian coordinates for the optimized geometry of isomer 5r-doublet of $Pt^+(C_2H_2)_5$ followed by its predicted frequencies (cm^{-1}) and IR intensities (km/mol).

Z	x	y	z
6	2.543640000	-0.884384000	0.680502000
6	3.290131000	-0.588418000	-0.459948000
6	2.587807000	0.593694000	-0.716693000
6	1.736231000	0.391128000	0.520038000
1	2.586768000	-1.647423000	1.441498000
1	4.132678000	-1.065040000	-0.938960000
1	2.656212000	1.376952000	-1.454644000
1	1.823231000	1.114387000	1.326878000
78	-0.369958000	0.212727000	0.050671000
1	-0.150309000	-2.170704000	1.626575000
6	-0.605786000	-1.791826000	0.728246000
6	-0.260819000	-1.797233000	-0.655958000
1	0.583517000	-2.149018000	-1.224298000
6	-1.659211000	-1.389732000	-0.994074000
1	-2.185382000	-1.192292000	-1.913767000
6	-1.997881000	-1.372860000	0.358447000
1	-2.896294000	-1.164663000	0.916337000
6	-1.390856000	2.041702000	-0.203775000
6	-0.225043000	2.320444000	0.137301000
1	-2.408551000	2.221259000	-0.483666000
1	0.605613000	2.948774000	0.387182000

Table S88. Predicted frequencies (cm^{-1}) and IR intensities (km/mol) of isomer 5r-doublet of $Pt^{+}(C_2H_2)_5$

Frequency	Intensity	Frequency	Intensity	Frequency	Intensity
40.907	0.1909	805.3938	54.2217	1214.7284	3.2535
74.101	0.2219	819.9312	16.2376	1309.009	23.3946
113.1447	4.37	850.5333	9.0133	1319.5878	14.3052
119.1491	5.8674	860.9785	3.4444	1330.4058	0.957
138.0275	0.5243	877.2403	9.954	1436.5725	3.8194
161.8682	5.0637	882.3715	1.9372	1443.5099	7.7304
176.4633	3.8283	905.9226	5.0457	1819.0376	6.6054
241.5591	2.4096	908.2551	3.0495	3095.1681	7.5005
254.2056	0.0929	917.5908	1.3786	3204.2011	0.422
325.7609	1.5732	927.94	15.5948	3224.574	0.1852
364.3134	10.0392	937.5076	0.6859	3234.7159	1.9124
390.4485	11.2033	949.6798	3.8787	3237.3598	0.2696
431.4012	10.6471	957.4849	3.7303	3251.8044	13.7483
515.3631	19.1826	963.2205	2.6795	3256.7813	14.4385
542.2371	18.623	1011.7714	2.1841	3268.9265	9.1917
593.2836	3.4377	1102.8954	58.7884	3304.8409	62.3628
620.5386	4.2088	1159.4883	3.4522	3372.9549	53.0616
687.8613	95.6554	1166.4322	0.9562		
746.8757	14.6038	1199.8305	2.5122		
751.4913	32.4105	1201.6219	8.6577		

Table S89. Cartesian coordinates for the optimized geometry of isomer 5s-doublet of $Pt^+(C_2H_2)_5$ followed by its predicted frequencies (cm^{-1}) and IR intensities (km/mol).

Z	x	y	z
78	0.613167000	0.051503000	-0.288431000
6	-1.055556000	0.979383000	0.132523000
6	-0.906460000	2.322692000	0.335568000
1	-1.791795000	2.947510000	0.412798000
1	-1.964406000	0.397381000	0.237528000
6	0.421981000	2.847813000	0.442741000
6	1.405833000	1.920247000	0.256892000
1	2.451090000	2.204261000	0.327170000
1	0.590449000	3.893676000	0.666338000
1	1.664728000	-0.479776000	2.083210000
6	1.274493000	-1.001959000	1.220471000
6	1.094594000	-2.347650000	1.080028000
1	1.569049000	-3.024955000	1.784552000
6	0.271956000	-2.808834000	-0.000148000
1	0.049808000	-3.861618000	-0.119799000
6	-0.198464000	-1.815064000	-0.805917000
1	-0.857562000	-2.041969000	-1.637499000
6	-4.564338000	-0.193506000	-0.383799000
6	-4.479685000	-0.465972000	0.779235000
1	-4.444926000	-0.715020000	1.812040000
1	-4.679591000	0.040387000	-1.414318000

Table S90. Predicted frequencies (cm^{-1}) and IR intensities (km/mol) of isomer 5s-doublet of $Pt^{+}(C_2H_2)_5$

Frequency	Intensity	Frequency	Intensity	Frequency	Intensity
4.936	0.0088	661.9167	0.0314	1127.8452	617.1781
21.0824	0.664	674.3302	8.0533	1257.9269	12.2182
35.7866	0.2905	685.9063	80.7039	1277.7412	209.7741
53.1649	0.8657	746.4065	9.846	1363.3595	1.1264
61.5005	2.0973	772.0258	6.6037	1415.3415	328.6488
83.6469	0.5106	778.0415	334.1613	1485.1915	7.5785
105.5252	3.4742	786.418	126.4225	1520.0969	44.5624
112.2593	18.6689	796.0329	170.8795	1525.8635	21.9324
133.7878	24.3505	806.6263	0.4436	2062.7067	11.4716
274.9454	1.1243	909.8052	457.8911	3141.4257	22.1128
330.1592	299.9732	924.7312	434.1039	3144.6294	0.9539
350.4427	2.2014	958.0658	1.4111	3147.0988	18.7489
378.7073	89.7438	997.7203	7.2943	3150.4749	2.982
458.9452	49.7119	999.7364	195.9195	3158.4456	104.8366
459.692	3.064	1020.6978	3.849	3182.5586	1.018
500.479	10.713	1103.8878	516.1901	3183.263	0.652
545.1231	719.3589	1111.6626	442.1114	3198.2525	8.3235
632.0955	28.341	1118.7555	93.818	3400.7949	104.0249
642.9425	75.1446	1124.2495	4.0619	3504.6306	0.6927

Table S91. Cartesian coordinates for the optimized geometry of isomer 5t-doublet of $Pt^+(C_2H_2)_5$ followed by its predicted frequencies (cm^{-1}) and IR intensities (km/mol).

Z	x	y	z
78	-0.674231000	0.226964000	0.287893000
6	-1.804370000	0.237208000	-1.312041000
6	-2.837309000	-0.650147000	-1.263304000
1	-3.614081000	-0.603495000	-2.021610000
1	-1.513511000	0.865226000	-2.142610000
6	-2.850597000	-1.617557000	-0.201174000
6	-1.827931000	-1.483321000	0.685209000
1	-1.720171000	-2.169397000	1.519113000
1	-3.614729000	-2.383092000	-0.156932000
6	1.827728000	1.601754000	-0.203108000
6	0.499141000	1.920144000	-0.127015000
1	0.177469000	2.953811000	-0.208225000
1	2.629029000	2.317907000	-0.335047000
1	3.117447000	-0.198914000	-0.123348000
6	2.104772000	0.201777000	-0.111819000
6	1.039690000	-0.653550000	-0.014811000
1	1.054609000	-1.726066000	-0.155295000
6	5.687272000	-0.970700000	0.343616000
6	5.611682000	-1.040199000	-0.849313000
1	5.582450000	-1.112209000	-1.909350000
1	5.791058000	-0.919426000	1.400230000

Table S92. Predicted frequencies (cm^{-1}) and IR intensities (km/mol) of isomer 5t-doublet of $Pt^{+}(C_2H_2)_5$

Frequency	Intensity	Frequency	Intensity	Frequency	Intensity
9.3846	0.1693	660.0796	0.0206	1131.1265	829.8261
21.6801	0.7907	674.9429	15.1417	1257.1459	16.2647
24.6524	0.1034	680.6773	60.4385	1278.5864	213.9145
54.5251	1.0008	742.9506	54.0914	1362.4167	1.7638
60.5372	2.4568	756.1409	162.0301	1416.2718	342.7778
91.0457	0.5073	772.0219	78.0978	1485.4476	11.4639
104.1716	7.0498	786.5165	225.9428	1512.4275	31.6824
109.8149	1.5539	796.3785	172.1771	1533.8087	25.9386
128.6518	31.386	805.9903	0.6153	2064.0478	12.1263
273.6757	16.9018	912.6138	418.2336	3103.9564	161.2265
329.0208	277.4081	930.9508	373.8898	3142.8618	0.8229
349.7926	10.7936	970.8131	124.2728	3144.6782	4.0852
379.2568	87.386	998.3174	36.186	3148.451	2.8888
456.6291	23.237	1009.7825	32.376	3181.8274	0.4115
465.019	13.0061	1021.5699	7.6319	3183.3789	0.8324
498.8043	37.3108	1104.1984	193.8866	3193.4398	13.0994
548.1241	665.5201	1114.2395	663.2826	3200.1974	8.7912
630.9334	44.6039	1116.2359	3.9711	3401.5939	106.16
645.7346	66.2905	1124.4265	25.3186	3505.3871	1.4775

Table S93. Cartesian coordinates for the optimized geometry of isomer 5u-doublet of $Pt^{+}(C_2H_2)_5$ followed by its predicted frequencies (cm^{-1}) and IR intensities (km/mol).

Z	x	y	z
78	-0.736389000	-0.000002000	0.000003000
6	0.784328000	1.264139000	0.128162000
6	2.051158000	0.699176000	0.086933000
1	2.965028000	1.274082000	0.171176000
1	0.671185000	2.339917000	0.222008000
6	2.051168000	-0.699174000	-0.086734000
6	0.784344000	-1.264142000	-0.128057000
1	0.671214000	-2.339920000	-0.221920000
1	2.965046000	-1.274074000	-0.170913000
6	-2.582773000	1.414659000	0.567701000
6	-2.082313000	1.908422000	-0.420271000
1	-1.753956000	2.494562000	-1.249116000
1	-3.144969000	1.093668000	1.415321000
1	-1.753957000	-2.494633000	1.248983000
6	-2.082311000	-1.908443000	0.420172000
6	-2.582776000	-1.414625000	-0.567771000
1	-3.144980000	-1.093587000	-1.415367000
6	5.846278000	-0.062831000	0.595165000
6	5.846206000	0.062844000	-0.595334000
1	5.881841000	0.174331000	-1.651586000
1	5.882041000	-0.174317000	1.651413000

Table S94. Predicted frequencies (cm^{-1}) and IR intensities (km/mol) of isomer 5u-doublet of $Pt^{+}(C_2H_2)_5$

Frequency	Intensity	Frequency	Intensity	Frequency	Intensity
13.0711	0.021	661.9243	0.0005	1116.9276	1.5321
16.2829	0.0064	670.5906	0.9329	1136.2159	5.598
35.9434	0.0016	671.9248	0.9124	1297.8382	11.8988
40.1898	1.5152	672.9322	2.506	1383.909	3.0543
49.2445	1.0421	676.5642	2.148	1471.9221	48.8936
74.3949	0.0713	687.2798	22.8179	1506.0498	19.3497
76.789	1.4313	722.6901	2.797	1980.51	0.4042
100.8579	4.3159	736.3322	52.8186	1981.6128	1.1184
105.4526	0.1919	769.0801	70.2497	2065.313	5.7177
158.6686	2.9155	772.9695	87.5725	3138.9592	5.2674
161.6619	2.1018	775.459	40.0339	3143.1787	0.3076
194.7422	0.1009	782.2719	133.953	3180.4107	0.165
227.3171	2.0876	806.6869	30.6388	3191.7293	5.287
246.6425	0.4879	814.5934	22.7931	3358.8825	48.8577
275.9859	0.4917	823.74	1.6829	3359.3372	246.0925
287.9784	10.827	834.9933	3.1826	3403.3903	103.8649
385.9475	1.6063	981.8105	0.9972	3450.7806	28.1036
393.2002	10.4488	1011.7312	0.0522	3451.6595	47.4075
515.8128	0.3717	1064.1746	0.0479	3507.4132	1.0354

Table S95. Cartesian coordinates for the optimized geometry of isomer 5v-doublet of $Pt^{+}(C_2H_2)_5$ followed by its predicted frequencies (cm^{-1}) and IR intensities (km/mol).

Z	x	y	z
6	1.658863000	0.686249000	-0.981294000
6	2.395761000	-0.056002000	0.177937000
6	1.529133000	-1.232447000	-0.372094000
6	1.284860000	-0.542101000	-1.595611000
1	1.771492000	1.689802000	-1.366206000
1	3.460719000	-0.181546000	-0.015668000
1	1.505738000	-2.281961000	-0.114539000
1	0.948923000	-0.828601000	-2.579828000
78	-0.257497000	0.006471000	-0.093219000
6	2.040195000	0.395203000	1.558057000
1	2.796377000	0.596832000	2.309409000
6	0.743451000	0.518147000	1.761654000
1	0.195311000	0.830191000	2.639440000
6	-1.887795000	-1.768405000	-0.130444000
6	-1.652392000	-1.478300000	1.031360000
1	-1.660027000	-1.467075000	2.098958000
1	-2.198504000	-2.138193000	-1.081583000
1	-2.511404000	1.480389000	1.166829000
6	-1.882612000	1.520064000	0.304654000
6	-1.405969000	1.861801000	-0.772020000
1	-1.164861000	2.370190000	-1.678950000

Table S96. Predicted frequencies (cm^{-1}) and IR intensities (km/mol) of isomer 5v-doublet of $Pt^+(C_2H_2)_5$

Frequency	Intensity	Frequency	Intensity	Frequency	Intensity
38.0006	0.1124	685.3003	1.1776	1153.6101	12.6395
66.1585	0.276	709.9783	6.5945	1205.8406	0.8723
95.6071	0.114	711.4635	1.8449	1247.0143	49.6382
125.5328	3.8056	734.69	62.5431	1341.2599	2.637
132.1412	3.873	745.2385	63.6755	1346.4808	12.1797
149.8254	2.8728	760.3212	13.7575	1385.1527	0.7431
178.2916	2.1594	769.0445	3.1224	1636.5473	12.8401
215.3951	3.8037	796.6508	0.9692	1905.1957	2.4218
227.9421	1.8621	798.1832	1.1594	1929.7657	2.1112
251.2784	2.5785	854.3005	3.3973	3083.4521	7.6634
291.6077	1.8598	888.1473	5.7158	3145.2685	0.3058
293.7858	3.7883	892.8019	5.6764	3197.6928	1.2156
333.8877	14.191	897.2905	9.0591	3198.4504	0.8077
342.0107	4.2468	919.3731	2.658	3205.1555	0.2296
426.5035	1.7906	975.6673	2.96	3236.0471	6.1192
449.644	1.0302	988.7152	2.5558	3344.7962	89.3436
510.1805	3.2914	999.9636	21.067	3350.2994	131.9447
666.9894	41.7813	1055.1838	3.8816	3424.9042	50.4444
672.4514	0.7189	1138.2484	13.7344	3435.8503	55.4517

Table S97. Cartesian coordinates for the optimized geometry of isomer 5w-doublet of $Pt^{+}(C_2H_2)_5$ followed by its predicted frequencies (cm^{-1}) and IR intensities (km/mol).

Z	x	y	z
78	-0.018610000	-0.481006000	0.068776000
1	-2.658657000	-0.271225000	-1.169292000
6	-1.024617000	-1.856584000	-1.088002000
6	-1.769529000	-0.839345000	-0.967312000
1	-0.781211000	-2.822553000	-1.487360000
1	1.470837000	2.067539000	0.098183000
6	-0.707978000	1.543346000	0.533547000
6	0.493118000	1.658913000	0.262935000
1	-1.722911000	1.791710000	0.776704000
1	1.255065000	-2.788116000	1.339659000
6	1.509213000	-1.885541000	0.825714000
6	2.154233000	-0.968527000	0.315797000
1	2.941559000	-0.322348000	-0.019033000
1	-4.714478000	0.748333000	1.363873000
6	-4.461049000	1.407488000	0.568767000
6	-4.221543000	2.169100000	-0.324278000
1	-4.047978000	2.861881000	-1.112306000
6	4.018520000	2.021042000	-1.009067000
6	4.238959000	2.097898000	0.166089000
1	4.471904000	2.188829000	1.200120000
1	3.861518000	1.977672000	-2.060223000

Table S98. Predicted frequencies (cm^{-1}) and IR intensities (km/mol) of isomer 5w-doublet of $Pt^{+}(C_2H_2)_5$

Frequency	Intensity	Frequency	Intensity	Frequency	Intensity
11.15	0.2511	436.0573	0.9829	850.7193	7.0623
23.5994	0.0601	538.6176	2.3417	888.9052	47.1691
30.1858	0.2847	669.6918	0.0149	1713.3055	2.535
35.9704	0.6272	671.2799	0.0136	1847.3455	4.1204
52.8985	0.1519	679.5747	0.2527	1875.2227	2.6784
53.2256	0.1108	681.4827	0.5973	2060.1051	11.9088
64.7191	0.0948	712.7548	15.0513	2061.1752	8.2858
69.9478	1.1518	737.8827	19.4722	3269.4568	50.1948
76.1097	2.2278	745.5238	10.7966	3272.3804	371.2938
87.5481	0.253	774.4949	18.9883	3295.9987	304.1703
94.1367	0.8146	779.5272	7.8565	3330.7729	40.7523
96.7605	3.4782	780.0418	41.6248	3352.4429	77.0785
122.3583	3.4973	782.2207	122.4812	3389.6894	49.1087
141.0367	4.1667	786.0386	69.109	3392.7622	117.1148
167.5262	14.5406	789.2412	137.4342	3395.7096	117.5584
187.8129	3.6979	792.4611	103.5537	3497.0637	1.2813
237.3611	2.3369	795.5105	75.3407	3500.1544	1.1259
318.8015	2.3406	799.1997	19.3896		
384.226	6.5421	826.6131	11.6425		
409.1803	12.072	844.2281	9.55		

Table S99. Cartesian coordinates for the optimized geometry of isomer 5x-doublet of $Pt^{+}(C_2H_2)_5$ followed by its predicted frequencies (cm^{-1}) and IR intensities (km/mol).

Z	x	y	z
78	0.035541000	-0.429190000	0.000085000
1	-2.860442000	0.260897000	-0.000144000
6	-1.892022000	-1.808066000	0.000249000
6	-2.313467000	-0.665490000	0.000038000
1	-1.729340000	-2.862928000	0.000436000
1	2.253967000	0.639303000	1.548799000
6	0.434652000	-0.659682000	2.023821000
6	1.345246000	0.066186000	1.538697000
1	-0.053012000	-1.193527000	2.816158000
1	2.254223000	0.638254000	-1.548989000
6	1.345568000	0.065038000	-1.538647000
6	0.435173000	-0.661324000	-2.023389000
1	-0.052268000	-1.195873000	-2.815388000
1	3.451814000	3.286921000	-0.000824000
6	3.964029000	2.354919000	-0.000487000
6	4.582837000	1.329255000	-0.000113000
1	5.168579000	0.441567000	0.000216000
6	-4.182639000	2.376913000	-0.599348000
6	-4.182961000	2.376946000	0.598216000
1	-4.212534000	2.407084000	1.660555000
1	-4.211646000	2.406992000	-1.661705000

Table S100. Predicted frequencies (cm^{-1}) and IR intensities (km/mol) of isomer 5x-doublet of $Pt^{+}(C_2H_2)_5$

Frequency	Intensity	Frequency	Intensity	Frequency	Intensity
6.4244	0.0227	432.5793	0.4058	826.9968	23.68
14.7724	0.0069	505.4714	0.0358	850.59	26.4842
17.1036	0.0075	561.5508	11.238	850.791	15.6195
32.2297	0.0364	660.8641	0	897.8721	69.7781
32.6575	0.0097	671.0443	0.0033	1724.7012	0.1811
52.395	0.0113	678.7512	0.7013	1743.674	3.8315
57.625	0.4932	679.0151	0.2098	1945.9486	30.4591
73.2137	0.8976	703.0624	5.7025	2061.2531	8.4684
73.7579	0.0731	738.6933	13.3965	2063.2914	6.9567
82.9798	0.3638	741.6093	0.6861	3254.2474	536.5293
98.971	6.0316	756.7414	76.113	3268.3262	21.9659
100.5004	2.4253	771.6516	75.4687	3271.7177	269.9224
117.4898	3.0559	776.493	1.3507	3336.1736	59.8309
149.1227	1.1981	780.7884	85.5717	3338.2826	22.7981
151.0569	7.1917	788.324	147.8087	3395.4009	116.1306
197.872	10.5661	792.0132	115.6232	3399.1079	106.584
228.232	1.1936	799.9122	5.9977	3413.8143	44.3301
279.8608	9.229	805.1171	28.836	3499.8627	1.1596
379.1288	0.9099	825.305	23.7073	3502.8195	0.745

Table S101. Cartesian coordinates for the optimized geometry of isomer 5y-doublet of $Pt^{+}(C_2H_2)_5$
followed by its predicted frequencies (cm^{-1}) and IR intensities (km/mol).

Z	x	y	z
78	0.846814000	-0.010235000	-0.006003000
1	1.411528000	-2.831792000	0.457610000
6	-0.472655000	-1.603579000	0.135827000
6	0.704929000	-2.042554000	0.286160000
1	-1.544124000	-1.695159000	0.072638000
1	-4.019144000	-2.227202000	-1.763874000
6	-4.136900000	-2.182039000	0.493588000
6	-4.056660000	-2.195082000	-0.701539000
1	-4.241812000	-2.190046000	1.551899000
1	3.250569000	0.029875000	-1.828664000
6	3.150771000	0.009044000	-0.766308000
6	3.239234000	-0.014286000	0.443352000
1	3.491603000	-0.034071000	1.479903000
1	-1.555120000	1.666897000	0.027239000
6	-0.479228000	1.584970000	0.041943000
6	0.702166000	2.036685000	0.102515000
1	1.411925000	2.838175000	0.176442000
6	-3.874090000	2.844188000	0.051304000
6	-4.140101000	1.679889000	-0.039636000
1	-4.407943000	0.653016000	-0.119347000
1	-3.673766000	3.885237000	0.131161000

Table S102. Predicted frequencies (cm^{-1}) and IR intensities (km/mol) of isomer 5y-doublet of $Pt^+(C_2H_2)_5$

Frequency	Intensity	Frequency	Intensity	Frequency	Intensity
4.4024	0.0141	459.9984	8.3432	828.0161	34.4915
20.9333	0.2554	521.7835	0.5966	843.8001	15.4854
27.2605	0.0078	592.2571	25.8884	890.1106	5.4749
33.2298	0.0259	667.6557	1.0746	939.1516	30.3083
38.5648	0.085	675.9392	0.2622	1693.6567	17.3904
49.9656	2.9483	676.3162	1.6701	1730.6383	6.0833
57.397	0.7621	680.903	0.0949	1979.2782	8.2786
65.7628	1.2353	687.8808	2.4961	2059.9909	10.553
73.3232	2.7613	725.227	0.2203	2061.7849	3.4645
86.144	1.2993	755.0956	52.541	3210.5589	325.1071
93.2771	1.0193	757.7267	17.4927	3234.8145	345.9956
103.9762	1.7687	767.1493	35.1446	3319.7257	90.1798
114.9331	0.4768	780.4676	100.6801	3323.2989	31.155
132.484	3.2294	784.3059	37.8399	3357.3022	169.0917
153.8429	2.9418	789.187	61.7243	3388.4414	158.6175
186.3726	7.1687	793.0416	34.3643	3395.5858	116.815
211.3835	2.759	800.7949	212.165	3449.7732	26.8948
243.1953	5.4542	806.7663	33.6584	3495.4024	0.2644
365.5012	0.1196	812.1345	1.0265	3499.3585	0.9647

Table S103. Cartesian coordinates for the optimized geometry of isomer 5z-doublet of $Pt^{+}(C_2H_2)_5$
followed by its predicted frequencies (cm^{-1}) and IR intensities (km/mol).

Z	x	y	z
78	-0.460239000	0.066238000	-0.152086000
1	-2.928580000	1.754170000	-0.281938000
6	-2.562611000	-0.416335000	-0.810484000
6	-2.569036000	0.766819000	-0.470278000
1	-2.916221000	-1.353475000	-1.179790000
1	2.263165000	1.304495000	0.015678000
6	0.171354000	2.193086000	-0.016826000
6	1.217408000	1.537039000	-0.005825000
1	-0.467849000	3.048961000	-0.012480000
1	2.233134000	-1.290488000	-0.217692000
6	1.208490000	-1.507036000	-0.437332000
6	0.172311000	-2.052794000	-0.797367000
1	-0.513148000	-2.777007000	-1.175604000
1	4.670639000	0.135640000	-1.656154000
6	4.614985000	0.021230000	-0.600106000
6	4.598975000	-0.109820000	0.590545000
1	4.631600000	-0.227367000	1.646985000
6	-1.163247000	-1.109464000	2.008707000
6	-0.570084000	-0.072691000	2.228768000
1	-0.095127000	0.766504000	2.684228000
1	-1.690242000	-2.028185000	1.900653000

Table S104. Predicted frequencies (cm^{-1}) and IR intensities (km/mol) of isomer 5z-doublet of $Pt^{+}(C_2H_2)_5$

Frequency	Intensity	Frequency	Intensity	Frequency	Intensity
26.6144	0.2707	335.5274	3.3183	797.8609	9.9789
44.1383	0.221	373.3313	2.9168	818.5652	1.8934
48.2637	0.1712	390.356	4.4419	835.6725	5.628
63.0573	0.0502	662.9529	1.7835	860.7893	7.7286
64.5541	0.401	668.5947	0.0349	1861.7889	4.5844
84.7269	8.1242	680.2801	0.4163	1884.4428	8.5813
90.0198	4.0122	690.8044	2.3565	1911.4358	4.4497
93.145	3.0459	695.5593	7.4598	1966.338	7.5385
98.7215	9.167	708.4289	2.0429	2061.1059	10.3177
116.6558	4.5951	716.9801	6.1151	3298.7982	148.9858
124.1136	0.7769	726.1719	72.7017	3319.0787	296.7702
134.6285	3.2008	743.1276	16.2436	3341.8999	76.9076
144.5601	1.9699	756.5795	69.5296	3370.3404	119.1076
153.5827	1.6119	764.879	5.9156	3390.3525	54.8895
159.1435	0.9954	768.5784	6.0999	3394.8738	114.9741
192.3328	0.3519	778.356	58.8848	3416.0304	70.5288
227.2896	0.2358	780.0322	107.2175	3417.2442	19.5107
244.9151	1.1203	788.1926	27.1802	3464.1395	20.697
313.0941	1.3535	791.9274	141.8159	3498.7918	1.1842

Table S105. Cartesian coordinates for the optimized geometry of isomer 5aa-doublet of $Pt^{+}(C_2H_2)_5$
followed by its predicted frequencies (cm^{-1}) and IR intensities (km/mol).

Z	x	y	z
78	0.164384000	-0.000005000	-0.050797000
1	-0.132165000	1.610730000	-2.557129000
6	-0.074396000	-0.618006000	-2.168342000
6	-0.074443000	0.617356000	-2.168516000
1	-0.132010000	-1.611495000	-2.556675000
1	-0.311298000	1.378022000	2.593103000
6	0.062506000	2.196390000	0.516238000
6	-0.116461000	1.582630000	1.564318000
1	0.183585000	2.998423000	-0.176812000
1	-0.311236000	-1.377334000	2.593478000
6	-0.116371000	-1.582231000	1.564757000
6	0.062655000	-2.196275000	0.516855000
1	0.183817000	-2.998487000	-0.175972000
1	2.775295000	-0.000100000	-1.425436000
6	2.575729000	0.000029000	-0.378248000
6	2.660419000	0.000189000	0.831137000
1	2.773507000	0.000329000	1.889567000
6	-3.403221000	-0.598970000	0.150271000
6	-3.403295000	0.598941000	0.150121000
1	-3.445205000	1.660593000	0.153044000
1	-3.444978000	-1.660626000	0.153468000

Table S106. Predicted frequencies (cm^{-1}) and IR intensities (km/mol) of isomer 5aa-doublet of $Pt^{+}(C_2H_2)_5$

Frequency	Intensity	Frequency	Intensity	Frequency	Intensity
23.75	1.7491	358.0502	2.8682	801.2989	41.2394
26.4058	0.1271	359.1105	1.7976	816.036	3.3279
43.5271	0.9677	398.8219	2.9282	833.0639	6.1397
45.9923	0.927	662.4511	0.8958	834.705	3.0136
64.821	3.8817	663.4662	0.6137	1862.9801	9.1324
75.7174	3.5251	674.925	0.0057	1898.2582	9.4151
79.1132	0.6394	687.1632	0.0846	1904.9547	3.3324
87.6832	9.3682	691.6971	0.2954	1978.2313	20.1739
101.9967	1.2038	704.3534	0.0776	2060.039	26.653
105.7222	3.4037	708.601	0.5934	3333.1117	68.3795
124.6196	1.0494	723.254	0.0793	3349.5527	41.9426
133.0642	1.5567	723.2723	10.3769	3350.1761	117.9888
135.2311	1.1089	736.912	237.3466	3374.2645	121.7174
141.5926	1.0083	746.0752	15.7448	3404.9698	42.6176
143.7752	0.0908	751.6713	14.9045	3406.4251	91.3948
182.8547	1.7308	762.7477	58.209	3426.3631	62.0356
233.3691	2.2429	769.4683	0.0007	3427.2336	17.6519
246.6956	3.5508	772.8269	65.289	3469.024	17.5151
314.6527	1.9927	783.2231	118.8935	3508.9028	0.1236

Table S107. Cartesian coordinates for the optimized geometry of isomer 5a-quartet of $Pt^+(C_2H_2)_5$ followed by its predicted frequencies (cm^{-1}) and IR intensities (km/mol).

Z	x	y	z
78	-0.277823000	-0.007461000	0.111198000
6	-1.875359000	1.210476000	-0.387420000
6	-3.062615000	0.576132000	0.004342000
1	-3.980773000	1.123592000	0.187102000
1	-1.837542000	2.284482000	-0.541060000
6	-2.999770000	-0.823005000	0.149482000
6	-1.720901000	-1.378292000	0.150654000
1	-1.564420000	-2.450626000	0.116309000
1	-3.891441000	-1.424277000	0.282933000
6	1.832708000	1.423722000	0.093061000
6	2.595540000	0.671202000	0.987460000
6	1.390328000	0.842827000	-1.119308000
1	2.935270000	1.128865000	1.906432000
1	0.970237000	1.452674000	-1.904821000
6	2.979771000	-0.648980000	0.683333000
6	1.654583000	-0.540400000	-1.341309000
1	3.626917000	-1.189647000	1.358227000
1	1.322909000	-1.000981000	-2.260444000
6	2.490214000	-1.255149000	-0.457716000
1	2.741686000	-2.281568000	-0.685145000
1	1.640371000	2.468267000	0.291552000

Table S108. Predicted frequencies (cm^{-1}) and IR intensities (km/mol) of isomer 5a-quartet of $Pt^+(C_2H_2)_5$

Frequency	Intensity	Frequency	Intensity	Frequency	Intensity
33.9162	1.4726	793.1052	11.3997	1307.395	61.7009
49.3743	3.2608	888.2142	2.7444	1343.7764	31.0764
67.355	2.5549	912.2285	0.3939	1386.0877	0.911
88.7189	1.7847	923.4182	0.7826	1407.0275	68.8754
107.2211	1.9613	966.3555	0.462	1435.1929	53.5368
171.2826	21.7475	972.5826	2.2648	1487.3362	24.8083
230.9432	0.2968	990.7038	1.478	1495.4156	28.5074
303.5798	0.3398	1008.9185	15.7791	1557.4558	5.4042
349.5156	43.8812	1018.8927	0.1256	1577.7304	7.6338
380.1624	2.7526	1024.3231	0.589	3139.3692	1.3308
427.5016	27.3142	1026.6805	0.7473	3155.8258	0.0954
432.3424	0.5348	1032.156	1.5066	3162.8531	0.1835
530.9756	58.2835	1049.0754	1.2004	3178.466	0.4731
595.7135	2.0315	1084.5676	7.5009	3190.2182	0.0857
606.119	0.8451	1100.5864	16.3792	3195.7456	0.2434
642.9235	48.8351	1177.4544	0.7412	3200.6667	0.3325
660.3511	7.6761	1191.9354	0.2118	3207.4658	1.9668
738.4126	20.8021	1194.2425	6.3951	3211.9988	2.4452
745.9684	83.8284	1217.1097	15.9616	3216.0687	0.2806

Table S109. Cartesian coordinates for the optimized geometry of isomer 5b-quartet of $Pt^+(C_2H_2)_5$ followed by its predicted frequencies (cm^{-1}) and IR intensities (km/mol).

Z	x	y	z
1	-1.168261000	-1.165723000	2.304915000
6	-0.896091000	-1.278876000	1.264432000
6	0.413625000	-1.586842000	0.932318000
6	0.795641000	-1.821701000	-0.457118000
6	-0.128322000	-1.599130000	-1.466737000
6	-1.305901000	-0.731254000	-1.153484000
6	-1.957438000	-1.102833000	0.208161000
6	-2.978643000	-0.072846000	0.605514000
6	-2.767180000	1.244423000	0.312418000
6	-1.646542000	1.654517000	-0.433168000
6	-0.777060000	0.711540000	-1.061366000
1	1.111265000	-1.868789000	1.710332000
1	1.722160000	-2.339684000	-0.661086000
1	0.083214000	-1.892108000	-2.485913000
1	-0.310244000	1.070656000	-1.977096000
1	-1.468926000	2.709968000	-0.593546000
1	-3.472982000	1.991860000	0.648676000
1	-3.848083000	-0.387749000	1.165460000
1	-2.058841000	-0.781973000	-1.942158000
1	-2.461588000	-2.072570000	0.106584000
78	1.017433000	0.413258000	0.118180000

Table S110. Predicted frequencies (cm^{-1}) and IR intensities (km/mol) of isomer 5b-quartet of $Pt^{+}(C_2H_2)_5$

Frequency	Intensity	Frequency	Intensity	Frequency	Intensity
90.6579	1.1942	903.7935	37.1973	1320.4785	8.1443
120.7589	8.9052	910.5652	4.8391	1355.9483	17.3294
170.0275	2.8283	929.0633	18.2857	1387.5121	14.0829
180.2146	0.7303	956.0616	10.9588	1391.2141	12.2313
205.8582	17.7811	965.8063	0.1091	1427.7991	16.8739
287.0982	8.1928	975.0403	5.2752	1443.0335	8.5567
341.6018	2.7313	987.4026	8.139	1456.6068	21.583
402.0414	5.3654	1005.431	6.895	1528.2246	7.9325
423.0499	0.0294	1008.2725	19.8651	1540.2748	16.8892
453.2767	20.3622	1019.9764	4.4776	2989.1332	0.8009
494.2454	9.6267	1037.9694	1.9554	3057.8328	0.9736
576.5538	1.275	1077.5154	0.5062	3088.7987	3.2643
589.0138	1.0065	1158.9683	4.9019	3173.4921	3.3676
642.3635	0.9237	1166.1985	4.6384	3178.4357	3.2719
713.8674	27.7795	1179.1857	3.7278	3188.4292	0.0871
732.0472	36.7674	1186.3993	4.8255	3190.8737	3.9697
761.0593	31.9539	1234.1424	5.4048	3193.8884	4.7536
775.2918	29.4129	1258.8405	29.995	3201.0668	1.7965
855.1242	0.389	1299.8882	7.1049	3203.9718	6.6873

Table S111. Cartesian coordinates for the optimized geometry of isomer 5c-quartet of $Pt^+(C_2H_2)_5$ followed by its predicted frequencies (cm^{-1}) and IR intensities (km/mol).

Z	x	y	z
78	0.187490000	-0.310423000	-0.050009000
6	2.049409000	0.002682000	-1.003888000
6	2.783344000	-0.531859000	0.177189000
1	3.119609000	-1.506756000	0.486913000
1	2.051250000	-0.339358000	-2.032103000
1	2.143626000	2.353581000	-0.855548000
6	2.315878000	1.356893000	-0.486523000
1	3.505588000	1.213249000	1.495966000
6	2.992534000	0.793350000	0.643737000
6	-1.474076000	-0.157047000	1.432082000
6	-1.964910000	-1.075212000	0.476500000
6	-1.446919000	1.212138000	1.075014000
1	-1.991457000	-2.130589000	0.705679000
1	-1.014360000	1.927610000	1.759750000
6	-2.582733000	-0.599211000	-0.706236000
6	-2.117779000	1.671019000	-0.080673000
1	-2.981747000	-1.315862000	-1.410672000
1	-2.158128000	2.732743000	-0.279026000
6	-2.706759000	0.768348000	-0.952216000
1	-3.229444000	1.115068000	-1.831596000
1	-1.157122000	-0.483284000	2.411435000

Table S112. Predicted frequencies (cm^{-1}) and IR intensities (km/mol) of isomer 5c-quartet of $Pt^+(C_2H_2)_5$

Frequency	Intensity	Frequency	Intensity	Frequency	Intensity
17.5647	0.4195	861.211	36.2234	1238.0591	5.9121
38.0461	1.8226	884.3644	16.6546	1332.4301	6.1003
63.8345	1.8451	902.3605	2.1453	1337.0822	11.3112
73.326	1.6701	909.6159	0.1823	1353.5503	21.1554
96.7602	0.4043	928.3862	0.4777	1385.5077	0.0813
102.8308	2.05	955.2766	2.0001	1483.9472	27.8252
124.6875	1.2538	977.0485	7.7683	1502.5345	20.5356
223.4896	2.2794	995.467	2.9567	1563.6011	6.3232
336.7634	0.4352	999.515	2.4582	1580.2312	9.9619
364.9823	1.0717	1008.194	1.6524	3160.5161	4.6551
439.521	1.2998	1022.5164	0.0288	3190.9106	0.0125
529.8077	10.659	1024.7995	0.4378	3196.1109	0.1648
596.9561	0.5513	1034.185	0.549	3199.8157	0.2476
603.477	0.1913	1056.2025	1.1561	3206.6765	2.0892
619.8348	27.8506	1177.9802	0.6235	3211.2305	3.3259
656.4965	23.5331	1189.3099	0.8009	3215.6099	0.2182
658.0612	45.6019	1193.9032	3.2435	3218.5421	1.3153
753.0167	60.0418	1196.069	1.7787	3250.2916	9.8772
804.9117	16.8274	1216.9198	1.4831	3258.9055	0.1449

Table S113. Cartesian coordinates for the optimized geometry of isomer 5d-quartet of $Pt^+(C_2H_2)_5$ followed by its predicted frequencies (cm^{-1}) and IR intensities (km/mol).

Z	x	y	z
6	-1.906636000	-1.846013000	-0.171464000
6	-1.027089000	-1.316191000	-1.130897000
6	-0.679389000	0.000258000	-1.553364000
6	-2.803519000	1.297179000	0.748563000
6	-1.906907000	1.846004000	-0.171020000
6	-1.027281000	1.316549000	-1.130573000
1	-1.902676000	2.929052000	-0.163414000
1	-1.902243000	-2.929063000	-0.164126000
1	-0.554843000	-2.077467000	-1.739238000
1	-0.107939000	0.000413000	-2.480686000
1	-3.340953000	2.057414000	1.302453000
1	-0.555110000	2.078042000	-1.738702000
78	0.962523000	0.000032000	-0.093295000
1	-3.340656000	-2.057997000	1.301951000
6	-2.803330000	-1.297547000	0.748250000
6	-3.178643000	-0.000257000	1.123185000
1	-3.940259000	-0.000405000	1.893471000
6	2.275337000	-0.640816000	1.256101000
6	2.275390000	0.640488000	1.256235000
1	2.630217000	1.592625000	1.607757000
1	2.630055000	-1.593061000	1.607439000

Table S114. Predicted frequencies (cm^{-1}) and IR intensities (km/mol) of isomer 5d-quartet of $Pt^{+}(C_2H_2)_5$

Frequency	Intensity	Frequency	Intensity	Frequency	Intensity
46.4785	0.0067	757.07	0.0088	1451.3721	1.3976
48.7895	0.3259	770.4545	0.1942	1473.0212	0.0031
76.9253	1.6808	774.0918	0.0264	1498.9955	16.5395
89.2683	5.1569	807.8603	15.9555	1499.0951	2.9779
103.3882	2.9569	825.0212	14.4115	1503.14	11.9938
111.8824	0.1321	891.8451	41.155	1558.8382	2.4107
173.8418	0.067	894.0021	0.3736	1562.5418	3.7715
291.3634	1.8183	908.227	0.0059	1627.7186	0.2429
330.2016	0.1916	932.9225	2.4849	1653.2848	8.2062
330.2321	0.008	940.1972	2.1012	3088.3133	6.0551
442.4685	0.2934	975.6758	0.6728	3146.4562	0.0737
461.4998	0.2644	987.0738	0.4323	3151.0353	0.0978
510.9615	11.6485	999.1432	0.359	3157.6456	0.6327
575.5818	0.8558	1010.8401	0.0337	3162.5449	1.4818
590.9775	0.3892	1197.0998	0.4381	3170.0523	0.0753
669.4464	85.7056	1201.4645	1.0193	3174.7257	0.3018
734.5445	39.0196	1316.3251	0.4136	3178.6228	1.7501
749.6372	8.7991	1334.3711	2.1589	3260.6476	73.6159
753.2959	4.2465	1370.8983	0.0115	3313.2683	55.7253

Table S115. Cartesian coordinates for the optimized geometry of isomer 5e-quartet of $Pt^+(C_2H_2)_5$ followed by its predicted frequencies (cm^{-1}) and IR intensities (km/mol).

Z	x	y	z
6	-0.223674000	1.683948000	-0.886283000
6	-1.108301000	2.260557000	0.089297000
6	-1.879966000	1.581751000	1.041838000
6	-1.662997000	0.336476000	1.576525000
6	-0.411015000	-0.445314000	1.578288000
6	-0.224015000	-1.683985000	0.886183000
6	-1.108892000	-2.260312000	-0.089309000
6	-1.880626000	-1.581269000	-1.041636000
6	-1.663421000	-0.336022000	-1.576259000
6	-0.411161000	0.445307000	-1.578273000
1	0.178605000	0.329702000	-2.486758000
1	-2.456086000	0.101150000	-2.177063000
1	-2.747106000	-2.117423000	-1.413885000
1	-1.232043000	-3.335223000	-0.024477000
1	0.499438000	-2.363038000	1.329579000
1	0.178836000	-0.330069000	2.486765000
1	-2.455619000	-0.100315000	2.177662000
1	-2.746203000	2.118125000	1.414333000
1	-1.231270000	3.335482000	0.024376000
1	0.499918000	2.362775000	-1.329800000
78	0.960973000	-0.000102000	-0.000038000

Table S116. Predicted frequencies (cm^{-1}) and IR intensities (km/mol) of isomer 5e-quartet of $Pt^{+}(C_2H_2)_5$

Frequency	Intensity	Frequency	Intensity	Frequency	Intensity
107.4076	6.3575	801.8925	21.0258	1360.3235	6.2811
122.3661	1.4096	813.9531	2.4521	1413.3125	15.9956
147.6288	0.4767	859.2235	1.0327	1421.5687	12.4005
181.685	1.6276	867.4408	5.5031	1424.7856	9.3768
210.6817	0.0551	894.5013	11.4086	1435.9847	6.7808
280.6815	0.0728	916.4657	1.2413	1473.7836	0.8271
288.8588	8.0024	926.5685	5.8615	1482.4138	10.9705
341.3739	1.6904	945.4604	0.3111	1509.2053	13.9843
372.1855	4.7014	963.5542	5.4791	1513.71	9.06
411.3158	21.8574	990.5792	0.0019	3086.1002	7.8591
443.2588	0.673	1002.0637	28.0618	3086.4376	3.2202
452.6816	0.2208	1019.195	2.9343	3122.7566	1.8399
483.7062	2.9146	1094.4997	0.986	3124.0322	0.7227
609.2227	21.5286	1152.1499	5.1176	3128.1224	1.0585
610.2704	40.1958	1197.5624	7.8817	3128.3533	2.045
706.3721	11.4159	1225.6079	3.4371	3148.459	1.0112
706.9285	23.8327	1240.5356	0.0385	3148.6888	0.0011
774.8139	12.4496	1244.2291	1.6072	3163.8451	2.4211
793.8917	18.1201	1359.6575	0.7114	3164.1285	0.7099

Table S117. Cartesian coordinates for the optimized geometry of isomer 5f-quartet of $Pt^{+}(C_2H_2)_5$ followed by its predicted frequencies (cm^{-1}) and IR intensities (km/mol).

Z	x	y	z
6	-2.916066000	-0.095469000	1.216865000
6	-1.809366000	-0.921974000	1.226377000
6	-3.468630000	0.312784000	-0.000319000
1	-1.399330000	-1.283305000	2.158729000
1	-4.346223000	0.945126000	-0.000722000
6	-1.228126000	-1.336908000	0.000726000
6	-2.916007000	-0.096941000	-1.216986000
1	-0.553170000	-2.183425000	0.001255000
1	-3.369868000	0.213290000	-2.147428000
6	-1.809308000	-0.923454000	-1.225451000
1	-1.399240000	-1.285913000	-2.157351000
1	-3.369951000	0.215899000	2.146914000
78	0.812322000	0.127037000	-0.000011000
6	2.265181000	-1.197594000	-0.642750000
6	2.265440000	-1.197128000	0.643035000
1	2.716198000	-1.541494000	1.560163000
1	2.715561000	-1.542620000	-1.559816000
1	0.131800000	2.560552000	-1.563603000
6	0.256711000	2.014025000	-0.642916000
1	0.132370000	2.561777000	1.561865000
6	0.256955000	2.014524000	0.641564000

Table S118. Predicted frequencies (cm^{-1}) and IR intensities (km/mol) of isomer 5f-quartet of $Pt^{+}(C_2H_2)_5$

Frequency	Intensity	Frequency	Intensity	Frequency	Intensity
16.4321	0.0161	676.1249	2.4323	1205.9729	13.1347
65.3402	7.9176	734.795	22.0674	1359.5627	9.3867
68.3064	1.2524	744.6748	73.7475	1389.3559	0.3105
76.7325	3.5967	793.9267	0.2093	1497.5035	14.0149
77.6836	0.1816	801.0013	1.3158	1499.9001	22.2139
101.3251	6.7646	854.7186	1.0815	1575.2973	1.2422
132.6412	2.2146	861.489	4.6768	1582.6511	5.0719
161.6451	0.8352	870.4877	2.9557	1613.8105	102.057
250.0196	0.0075	875.4632	94.6566	1675.7874	5.9305
365.9656	0.4823	908.8925	20.1283	3168.7259	1.5983
400.963	0.008	973.01	26.52	3185.1065	0.1776
402.8737	0.451	1002.0355	0.9383	3193.0955	0.2875
424.2182	0.8122	1016.365	0.0031	3198.1409	0.0307
493.9275	0.0909	1030.4673	0.5038	3205.434	0.0453
506.0845	0.0007	1039.3373	0.0957	3208.5972	46.9616
604.0095	0.3946	1045.34	2.3456	3209.3048	0.9752
612.6765	4.485	1057.4243	1.731	3213.4665	22.7272
645.1053	7.3895	1180.9732	0.6092	3257.8842	27.3816
671.3981	69.1594	1194.914	2.4638	3263.0578	17.6905

Table S119. Cartesian coordinates for the optimized geometry of isomer 5g-quartet of $Pt^+(C_2H_2)_5$ followed by its predicted frequencies (cm^{-1}) and IR intensities (km/mol).

Z	x	y	z
6	0.048668000	-1.044536000	1.748136000
6	1.356562000	-1.284103000	1.314809000
6	2.029607000	-1.111431000	0.000135000
6	-1.298098000	-1.192955000	-1.269626000
6	0.048768000	-1.044896000	-1.747992000
6	1.356632000	-1.284371000	-1.314540000
1	0.027903000	-0.821938000	-2.810232000
1	0.027746000	-0.821354000	2.810328000
1	2.073023000	-1.342286000	2.126067000
1	2.949778000	-1.694802000	0.000220000
1	-2.023220000	-1.132258000	-2.071153000
1	2.073138000	-1.342748000	-2.125745000
78	-0.397405000	0.678946000	-0.000051000
1	-2.023337000	-1.131975000	2.071233000
6	-1.298178000	-1.192763000	1.269746000
6	-1.873809000	-1.329156000	0.000056000
1	-2.951563000	-1.427072000	0.000029000
6	2.421651000	0.402573000	-0.000016000
6	1.476883000	1.366402000	-0.000121000
1	3.481361000	0.633428000	-0.000031000
1	1.750681000	2.414676000	-0.000240000

Table S120. Predicted frequencies (cm^{-1}) and IR intensities (km/mol) of isomer 5g-quartet of $Pt^{+}(C_2H_2)_5$

Frequency	Intensity	Frequency	Intensity	Frequency	Intensity
73.774	2.5305	801.4786	34.6435	1376.8573	0.1124
119.6728	4.0103	847.547	5.5476	1423.4208	2.275
122.5228	0.1193	854.5379	11.9844	1451.3952	0.0284
137.5621	0.4128	860.7603	0.0304	1467.0365	26.4971
174.5423	1.8384	888.3549	4.0176	1475.7604	14.7341
234.5842	0.3918	900.7349	0.5777	1489.218	1.7139
288.1434	3.8397	940.3164	1.8675	1501.1236	5.4403
289.7273	0.9861	955.1649	2.2889	1543.4751	6.0298
336.345	1.0439	995.1465	1.5518	1552.2156	0.7199
344.2427	0.9403	1003.9937	4.5913	3078.8988	0.6196
412.9838	1.3495	1017.1571	0.8125	3127.7824	0.0311
503.1931	6.2257	1125.3577	0.7875	3127.7965	0.2421
547.0778	9.5133	1126.0871	17.2731	3148.9497	0.9121
659.6123	2.8404	1171.2041	2.8039	3158.4911	0.5301
683.5571	4.8796	1216.3813	3.5072	3159.1098	1.1978
704.606	10.633	1276.8244	29.1548	3166.3086	0.5674
718.4226	31.7444	1297.5244	2.0453	3171.4762	1.3522
759.9475	16.5814	1338.8828	0.3794	3171.7588	1.4474
770.1443	2.6569	1363.3524	1.5834	3185.7618	1.7794

Table S121. Cartesian coordinates for the optimized geometry of isomer 5h-quartet of $Pt^{+}(C_2H_2)_5$ followed by its predicted frequencies (cm^{-1}) and IR intensities (km/mol).

Z	x	y	z
6	2.698208000	1.388935000	0.483579000
6	2.753148000	0.377325000	-0.528290000
6	1.790870000	-0.536136000	-0.830248000
6	-2.698560000	1.388630000	-0.483521000
6	-2.753392000	0.376653000	0.527972000
6	-1.790909000	-0.536600000	0.829901000
1	-3.715221000	0.271329000	1.024907000
1	3.658251000	1.727449000	0.855673000
1	3.714891000	0.272476000	-1.025492000
1	1.979640000	-1.210485000	-1.670551000
1	-3.658645000	1.727077000	-0.855566000
1	-1.979681000	-1.211183000	1.670021000
78	0.000138000	-0.904505000	0.000004000
1	1.753294000	2.519276000	1.968311000
6	1.590331000	1.932912000	1.070418000
6	-1.590762000	1.933060000	-1.070117000
1	-1.753837000	2.519680000	-1.967822000
1	-0.517258000	1.961137000	1.435546000
6	0.223356000	1.838511000	0.654132000
6	-0.223794000	1.838825000	-0.653852000
1	0.516807000	1.961964000	-1.435198000

Table S122. Predicted frequencies (cm^{-1}) and IR intensities (km/mol) of isomer 5h-quartet of $Pt^{+}(C_2H_2)_5$

Frequency	Intensity	Frequency	Intensity	Frequency	Intensity
51.009	0.4399	792.2235	5.9574	1321.4931	11.3417
82.5663	0.042	818.6749	15.5706	1357.3525	3.1242
111.0312	0.1219	855.9838	4.4401	1462.7351	131.9467
118.973	0.8847	919.1776	11.1376	1466.3053	3.9496
171.176	14.6607	926.5438	0.0157	1477.0742	61.9039
186.0178	0.2082	938.76	1.9428	1523.7163	7.946
216.8107	1.7204	938.7646	0.9979	1537.9026	3.6173
305.9248	6.5893	970.2778	2.2301	1580.3652	43.0215
321.8084	9.8482	995.6454	4.5113	1596.2591	1.7314
337.3385	4.4032	1016.9355	3.6258	3045.6833	12.7064
407.5124	2.1403	1017.4101	13.2762	3046.8621	0.9869
440.4926	0.8715	1040.0466	5.8294	3115.0047	0.816
472.3877	20.1325	1084.2896	0.8498	3115.1109	0.0313
524.3576	0.8081	1156.0331	8.0189	3146.6481	1.2451
562.2503	7.9132	1158.0284	10.7127	3148.9561	0.3947
629.5214	124.4082	1241.1211	4.1322	3159.1067	0.1481
643.6389	40.6608	1255.7394	1.3286	3167.0278	0.5657
644.5548	0.5164	1287.1748	57.4405	3169.4084	0.0312
768.8016	9.4137	1294.2201	1.7244	3171.2538	1.9807

Table S123. Cartesian coordinates for the optimized geometry of isomer 5i-quartet of $Pt^+(C_2H_2)_5$ followed by its predicted frequencies (cm^{-1}) and IR intensities (km/mol).

Z	x	y	z
6	2.953102000	-1.031043000	-0.121904000
6	1.992314000	-2.039035000	0.058948000
6	0.641923000	-1.853544000	0.009237000
6	1.281983000	2.382022000	0.105738000
6	-0.020452000	2.817146000	-0.029114000
6	-1.010568000	1.871450000	-0.136145000
1	-0.262201000	3.873534000	-0.088755000
1	3.979140000	-1.333003000	-0.287000000
1	2.353058000	-3.056541000	0.178851000
1	-0.013424000	-2.720531000	0.045886000
1	2.131308000	3.048722000	0.042956000
1	-2.072965000	2.067413000	-0.219862000
78	-0.476801000	-0.136488000	0.001416000
1	3.428540000	0.979827000	-0.574832000
6	2.662903000	0.328655000	-0.164681000
6	1.480217000	0.946278000	0.277723000
1	1.095005000	0.541595000	1.248606000
6	-2.304922000	-0.910916000	-0.653973000
6	-2.358722000	-0.945558000	0.591485000
1	-2.719889000	-1.110404000	1.586340000
1	-2.634783000	-1.037260000	-1.666490000

Table S124. Predicted frequencies (cm^{-1}) and IR intensities (km/mol) of isomer 5i-quartet of $Pt^+(C_2H_2)_5$

Frequency	Intensity	Frequency	Intensity	Frequency	Intensity
65.7207	2.9298	681.2532	32.3	1243.4447	4.9027
94.6311	0.6905	716.9868	6.3608	1287.6791	8.224
112.6888	3.6475	727.9143	7.1582	1306.4025	16.6926
137.1464	1.4789	749.547	41.6233	1426.6323	22.9475
153.0503	0.0791	768.6758	32.9388	1457.3192	7.4411
198.8441	0.3974	796.285	11.3609	1487.1519	32.2691
267.5729	3.0006	818.9077	1.2446	1512.5428	2.363
279.1377	3.7911	832.4909	27.2571	1558.0956	24.6008
296.4577	13.7991	855.3748	3.5506	1801.339	0.5746
341.2382	3.1517	925.8308	2.8608	2753.4429	18.1022
368.5901	3.7445	962.1503	1.6383	3112.606	3.3082
407.3911	1.7219	998.9167	1.3598	3136.2415	6.6495
427.2068	6.1229	1007.2655	1.0554	3143.1301	0.8305
443.7198	1.3787	1054.8303	24.2069	3145.8542	0.0898
469.9672	0.3025	1069.1534	2.1152	3165.367	8.5453
487.4504	0.465	1124.159	0.1333	3186.768	0.5053
556.5199	1.1342	1150.4513	6.8073	3191.475	0.4326
648.8128	70.0841	1171.1684	3.5506	3298.3978	116.5516
669.7243	41.0399	1203.5497	39.5523	3369.4641	71.888

Table S125. Cartesian coordinates for the optimized geometry of isomer 5j-quartet of $Pt^{+}(C_2H_2)_5$ followed by its predicted frequencies (cm^{-1}) and IR intensities (km/mol).

Z	x	y	z
6	-3.289106000	0.448697000	-0.352166000
6	-2.436809000	1.539776000	-0.108520000
6	-1.081570000	1.537711000	0.162072000
6	-3.161280000	-0.914457000	-0.076536000
6	-2.138633000	-1.660288000	0.545771000
6	-0.824126000	-1.362339000	0.781325000
1	-2.456307000	-2.641778000	0.893921000
1	-4.271662000	0.724540000	-0.717853000
1	-2.933457000	2.505276000	-0.149704000
1	-0.688457000	2.528591000	0.386871000
1	-4.063800000	-1.483784000	-0.265233000
1	-0.258209000	-2.028567000	1.423524000
78	0.273362000	0.063520000	-0.094257000
6	1.874084000	1.123175000	0.536072000
6	3.097684000	0.478704000	0.439041000
1	3.996516000	0.894476000	0.880504000
1	1.755892000	2.104821000	0.982650000
1	4.015189000	-1.254116000	-0.512407000
6	3.095696000	-0.725354000	-0.290019000
6	1.833195000	-1.168860000	-0.687428000
1	1.767270000	-2.084620000	-1.267885000

Table S126. Predicted frequencies (cm^{-1}) and IR intensities (km/mol) of isomer 5j-quartet of $Pt^{+}(C_2H_2)_5$

Frequency	Intensity	Frequency	Intensity	Frequency	Intensity
45.3219	3.9968	792.5567	5.8384	1384.0427	9.6477
81.678	0.2668	798.8864	3.5419	1456.6261	69.0243
86.5721	2.6021	860.2319	0.5399	1478.6589	37.0507
125.712	4.1252	873.2989	2.6509	1482.9407	36.7939
178.8331	3.501	900.0616	3.9345	1502.5188	27.1401
198.9797	0.9672	938.2122	11.9209	1550.2301	26.1204
208.6813	0.947	976.5429	4.4189	1560.4573	30.128
322.3398	1.2615	988.043	3.6461	3087.485	3.6866
337.6501	2.9755	1001.482	0.2727	3098.0229	1.4866
339.5663	10.9705	1038.4349	0.68	3126.7331	3.0729
375.3772	1.3116	1043.3937	3.2182	3137.9902	1.9298
461.9253	11.1522	1064.7709	0.0975	3150.3267	1.6859
472.4269	3.4848	1101.6583	4.5243	3151.6055	0.695
505.3389	0.8501	1128.5925	1.2711	3155.0719	0.0674
569.9728	7.7656	1189.6928	1.5532	3166.6953	2.3348
592.0935	2.5097	1249.8118	3.1954	3171.2068	0.6375
657.3847	13.825	1263.1252	15.4839	3178.3256	0.5806
666.6381	8.4623	1304.1371	23.9028		
675.1476	87.0846	1313.24	9.806		
732.6953	11.682	1349.5501	35.3305		

Table S127. Cartesian coordinates for the optimized geometry of isomer 5k-quartet of $Pt^{+}(C_2H_2)_5$ followed by its predicted frequencies (cm^{-1}) and IR intensities (km/mol).

Z	x	y	z
6	-3.048420000	-0.123151000	-0.693563000
6	-2.073748000	0.215928000	-1.661510000
6	-0.721429000	0.374045000	-1.551153000
6	-3.048426000	-0.123236000	0.693528000
6	-2.073761000	0.215724000	1.661525000
6	-0.721440000	0.373858000	1.551197000
1	-2.483212000	0.355717000	2.659786000
1	-4.019175000	-0.343633000	-1.122470000
1	-2.483192000	0.356045000	-2.659757000
1	-0.194856000	0.686734000	-2.451092000
1	-4.019184000	-0.343771000	1.122401000
1	-0.194876000	0.686438000	2.451180000
78	0.511947000	-0.065661000	0.000002000
6	1.220056000	2.167462000	0.000122000
6	2.324600000	1.645820000	0.000063000
1	3.323282000	1.271151000	0.000011000
1	0.462176000	2.919193000	0.000187000
1	1.425224000	-2.419009000	-1.607299000
6	1.306835000	-2.008872000	-0.623986000
6	1.306852000	-2.008936000	0.623760000
1	1.425269000	-2.419187000	1.607023000

Table S128. Predicted frequencies (cm^{-1}) and IR intensities (km/mol) of isomer 5k-quartet of $Pt^{+}(C_2H_2)_5$

Frequency	Intensity	Frequency	Intensity	Frequency	Intensity
42.0085	0.9715	666.0238	6.8124	1315.8554	0.9726
46.7842	2.0546	676.2841	0.877	1321.1285	8.5365
70.6673	2.4399	683.3744	16.0563	1371.6271	9.973
75.2119	1.4451	707.2022	10.9655	1492.581	37.0717
96.0409	0.6901	714.6258	72.6037	1531.2032	3.5714
105.5594	3.6975	732.5217	2.0782	1551.7785	30.0039
131.076	5.9711	757.6488	16.3906	1579.9632	43.3794
140.7453	1.0499	758.5673	37.0826	1787.0272	17.8882
158.7239	0.5296	771.2253	38.8054	1918.4219	1.727
178.6556	5.7836	780.4645	10.3528	3092.5905	0.1082
233.783	0	856.1061	1.1147	3093.8876	0.2986
311.0755	3.8434	859.2319	0.0207	3112.8994	0.4271
324.7808	1.1959	887.6055	1.1401	3114.5917	0.4407
353.5981	1.1099	985.5193	0.9507	3151.1232	0.0183
360.1939	1.1902	998.4779	1.11	3168.199	1.3634
470.7416	1.9858	1041.4177	0.0182	3288.766	76.561
479.5698	1.3452	1056.1968	0.6256	3354.4114	119.8929
551.1482	0.1552	1190.1752	1.4161	3355.5979	58.2081
612.2709	40.5135	1225.9968	7.9837	3439.8564	56.8251

Table S129. Cartesian coordinates for the optimized geometry of isomer 5l-quartet of $Pt^{+}(C_2H_2)_5$ followed by its predicted frequencies (cm^{-1}) and IR intensities (km/mol).

Z	x	y	z
6	2.613209000	-1.369510000	0.069509000
6	1.758791000	1.756355000	0.205138000
6	1.346191000	0.573751000	0.988579000
6	2.229014000	-0.564979000	1.123963000
1	3.471677000	-2.018659000	0.194111000
1	2.805802000	2.021407000	0.142692000
1	0.898949000	0.879032000	1.938081000
1	2.611413000	-0.799255000	2.112646000
78	-0.518671000	-0.090785000	0.036093000
6	0.756899000	2.500644000	-0.398257000
1	0.950132000	3.494677000	-0.787695000
6	-0.475241000	1.906185000	-0.540247000
1	-1.331195000	2.373872000	-1.008010000
6	-2.703524000	-0.202821000	0.033060000
6	-2.310007000	-1.210889000	0.639451000
1	-2.331674000	-2.143836000	1.165471000
1	-3.381345000	0.510319000	-0.388987000
1	0.127688000	-1.071567000	-2.273905000
6	0.599635000	-0.992228000	-1.299114000
6	1.896396000	-1.436765000	-1.141913000
1	2.366737000	-1.923240000	-1.990652000

Table S130. Predicted frequencies (cm^{-1}) and IR intensities (km/mol) of isomer 5l-quartet of $Pt^+(C_2H_2)_5$

Frequency	Intensity	Frequency	Intensity	Frequency	Intensity
44.7285	3.8451	731.5274	11.1839	1335.0092	1.1374
82.2966	1.1653	737.7049	53.3925	1424.9386	8.7607
103.2387	0.6872	748.6544	32.8351	1430.5512	33.7959
112.5902	1.1309	770.0588	13.0245	1436.6502	10.3406
138.4259	1.7089	777.2185	4.816	1480.9588	11.3022
173.6899	2.4136	805.1136	10.7615	1515.5082	31.2647
235.1748	5.9437	857.5165	0.3005	1828.0364	7.9346
267.6057	2.3075	931.1411	1.9132	3037.1422	1.125
293.0002	2.4914	938.2829	8.5177	3135.125	0.3945
324.9073	6.5241	955.869	5.793	3143.9762	0.4899
337.6376	1.0901	982.8091	2.3378	3148.3094	0.1712
374.2796	11.4761	990.6295	8.3428	3151.6724	2.8015
402.5245	1.9717	1021.5807	0.1622	3175.2478	1.171
425.716	0.1425	1070.074	8.1962	3184.387	0.068
486.1997	2.0074	1094.4919	3.1814	3189.2881	1.4542
513.0697	15.8287	1142.8778	1.9195	3305.6594	102.3252
625.665	8.5019	1177.1789	4.3382	3377.9345	79.1068
669.4763	22.3092	1205.4055	0.079		
686.4922	29.9563	1228.6803	22.7387		
707.4864	1.4906	1300.0675	8.5772		

Table S131. Cartesian coordinates for the optimized geometry of isomer 5m-quartet of $Pt^{+}(C_2H_2)_5$
followed by its predicted frequencies (cm^{-1}) and IR intensities (km/mol).

Z	x	y	z
6	-1.605533000	-0.726402000	0.916024000
6	-2.683717000	0.309829000	0.458874000
6	-2.895498000	-0.675104000	-0.691943000
6	-1.909249000	-1.541468000	-0.221457000
1	-1.372770000	-1.025973000	1.930817000
1	-3.545630000	0.415531000	1.117586000
1	-3.566865000	-0.687982000	-1.538026000
1	-1.574492000	-2.517517000	-0.541956000
78	0.238051000	-0.054970000	-0.024404000
6	-2.079500000	1.617293000	0.059409000
1	-2.703295000	2.501863000	-0.031524000
6	-0.773265000	1.676226000	-0.198974000
1	-0.231049000	2.573630000	-0.462534000
1	2.520154000	-1.691214000	1.257585000
6	2.385028000	-0.835585000	0.615466000
6	2.547325000	0.578006000	0.785180000
1	2.732214000	1.225248000	1.625631000
6	2.199100000	-0.656343000	-0.839315000
6	2.243091000	0.780910000	-0.627047000
1	2.308354000	1.626055000	-1.292211000
1	2.298693000	-1.296127000	-1.699149000

Table S132. Predicted frequencies (cm^{-1}) and IR intensities (km/mol) of isomer 5m-quartet of $Pt^+(C_2H_2)_5$

Frequency	Intensity	Frequency	Intensity	Frequency	Intensity
23.7593	3.0287	802.5901	7.9264	1213.7172	0.6788
59.3092	0.6636	840.5845	5.3785	1248.756	1.01
87.944	1.5075	846.7368	5.8078	1274.7081	32.4501
91.2876	7.821	866.6326	12.1576	1313.5733	2.2959
178.9883	7.608	880.6355	4.875	1318.7415	17.5845
188.0742	2.5478	904.1797	14.3333	1345.9723	8.4913
235.9332	8.6564	912.1948	4.0831	1351.7626	10.3027
258.7951	0.4979	915.7592	6.3224	1390.6903	12.7182
289.9989	0.626	930.4282	0.46	1588.825	1.0469
309.5884	3.4881	943.8961	4.5826	3081.1665	2.0095
409.2262	5.4909	947.433	11.3093	3134.187	1.6482
437.9434	1.528	972.5536	0.6605	3162.1073	3.0689
478.6107	4.8748	1012.0427	7.5988	3191.3543	0.8869
544.2758	1.6617	1025.7785	0.7856	3204.0867	1.4831
669.4063	30.2884	1104.0804	9.5814	3224.4653	2.0484
684.3565	74.8456	1151.7273	7.6096	3233.0375	2.6981
707.596	26.368	1165.1945	5.7213	3243.6036	8.4873
727.164	28.9486	1192.1991	1.0602	3254.386	21.7267
748.3157	17.6889	1204.5216	0.1997	3265.9539	5.3614

Table S133. Cartesian coordinates for the optimized geometry of isomer 5n-quartet of $Pt^+(C_2H_2)_5$ followed by its predicted frequencies (cm^{-1}) and IR intensities (km/mol).

Z	x	y	z
6	-3.081062000	0.416284000	0.102093000
6	-2.907667000	-0.829331000	-0.533252000
6	-1.906200000	1.041178000	0.507892000
1	-3.735492000	-1.382670000	-0.960574000
1	-1.929397000	2.019837000	0.975778000
6	-1.602003000	-1.293642000	-0.560080000
1	-1.353832000	-2.260081000	-0.987284000
1	-4.058522000	0.857632000	0.255676000
78	-0.235904000	0.036690000	0.134416000
6	1.630740000	-1.012909000	-0.725135000
6	1.944549000	-1.511513000	0.565032000
1	1.609835000	-2.370108000	1.129425000
1	1.288509000	-1.519113000	-1.617594000
1	3.567741000	-0.006145000	-1.244477000
6	2.705993000	0.094401000	-0.583477000
1	3.612011000	-0.347422000	1.603245000
6	2.922750000	-0.549190000	0.796932000
6	2.150704000	1.482418000	-0.580833000
6	0.841222000	1.690453000	-0.399141000
1	2.838303000	2.320157000	-0.673092000
1	0.367208000	2.657230000	-0.505747000

Table S134. Predicted frequencies (cm^{-1}) and IR intensities (km/mol) of isomer 5n-quartet of $Pt^+(C_2H_2)_5$

Frequency	Intensity	Frequency	Intensity	Frequency	Intensity
50.069	1.7614	793.1501	9.7562	1252.9638	1.0651
68.06	2.9043	802.2031	0.7756	1270.0557	19.4798
75.3524	3.0013	818.824	6.8872	1319.5994	5.3838
109.3794	2.6217	873.461	11.0413	1346.2921	32.6613
189.7977	0.3748	885.5155	11.3245	1351.9902	28.5401
235.6371	6.8125	905.6963	21.6358	1411.7988	8.7164
251.8277	0.5988	932.7861	11.6494	1460.456	53.9886
281.9626	0.8622	947.4601	30.1514	1479.9563	13.977
352.4396	11.4999	954.6346	8.8025	1557.8381	20.7872
385.6904	1.614	992.1752	0.3524	3074.438	0.9317
401.3479	1.013	1011.8777	3.3575	3118.4977	0.9287
421.3005	10.9553	1020.1339	0.4901	3144.658	0.2573
485.196	1.3212	1046.9974	4.3438	3152.5272	0.1114
521.1797	1.0583	1103.5149	4.2409	3172.0134	0.2548
628.182	10.2526	1108.791	5.6593	3181.4764	0.8395
657.7997	35.7927	1127.4623	0.686	3184.0874	2.1122
684.047	61.6525	1149.6101	18.6718	3184.6328	0.9491
703.5982	47.1533	1170.7764	5.6678	3205.9722	1.6468
755.9823	33.6541	1216.4214	1.3353	3227.7482	1.9694

Table S135. Cartesian coordinates for the optimized geometry of isomer 5o-quartet of $Pt^{+}(C_2H_2)_5$ followed by its predicted frequencies (cm^{-1}) and IR intensities (km/mol).

Z	x	y	z
6	2.227439000	1.722196000	0.680856000
6	2.389189000	0.629201000	-0.370699000
6	1.045616000	1.197128000	-0.916427000
6	1.037248000	2.169453000	0.129762000
1	2.846644000	2.060293000	1.498907000
1	3.240315000	0.787557000	-1.036448000
1	0.721320000	1.297633000	-1.943823000
1	0.390913000	3.005518000	0.353623000
78	-0.675306000	0.003308000	0.056972000
6	2.335346000	-0.799376000	0.134657000
1	3.263508000	-1.078014000	0.639286000
6	1.061617000	-1.178525000	0.939026000
6	1.846668000	-1.877100000	-0.828190000
1	2.239494000	-2.292054000	-1.744531000
1	0.874088000	-1.151194000	2.005425000
6	0.746757000	-2.180361000	-0.021158000
1	0.004183000	-2.963768000	-0.056121000
6	-2.657968000	-0.258013000	0.319993000
6	-2.479457000	0.528076000	-0.680967000
1	-2.893427000	1.110430000	-1.484956000
1	-3.327923000	-0.750508000	1.003719000

Table S136. Predicted frequencies (cm^{-1}) and IR intensities (km/mol) of isomer 5o-quartet of $Pt^{+}(C_2H_2)_5$

Frequency	Intensity	Frequency	Intensity	Frequency	Intensity
33.0085	1.7457	792.6164	8.8665	1217.7573	0.519
77.0519	2.9044	799.2193	11.7362	1223.8268	0.1229
99.7667	2.5881	808.5416	6.9593	1295.334	0.4108
106.6374	11.1904	814.8528	5.9599	1332.7026	2.437
150.2096	1.0442	863.949	3.1006	1348.1445	19.5071
160.8065	2.9394	881.2312	65.1675	1353.1739	4.5829
197.432	2.4995	893.3086	1.3464	1402.5923	14.4183
236.9341	0.8677	902.4418	8.3327	1420.8999	20.7432
261.758	3.2221	922.5948	1.6693	1630.9288	5.4098
335.9683	4.241	927.2248	4.5892	3050.4858	1.0652
413.6078	0.7038	942.6054	11.0938	3058.9945	3.8495
445.5757	0.1187	953.8372	4.0665	3169.0275	4.5055
482.8644	4.9143	1018.7425	0.0555	3176.8996	2.4821
519.4502	0.8541	1022.1172	0.1948	3204.1476	1.0677
608.0766	30.0644	1061.3353	3.7494	3208.2748	1.6692
661.5671	25.2018	1148.492	11.0663	3224.9711	0.1439
728.8679	39.6918	1162.5601	8.7131	3227.8751	0.5722
762.5409	31.9308	1165.9183	3.9397	3244.0676	54.4977
787.2306	3.7536	1194.0166	0.2377	3295.5902	39.8284

Table S137. Cartesian coordinates for the optimized geometry of isomer 5p-quartet of $Pt^+(C_2H_2)_5$ followed by its predicted frequencies (cm^{-1}) and IR intensities (km/mol).

Z	x	y	z
6	2.717030000	1.226554000	-0.960572000
6	2.855581000	1.381639000	0.420456000
6	1.589572000	0.488717000	-1.331763000
1	3.666954000	1.923610000	0.887962000
1	1.346850000	0.286252000	-2.368819000
6	1.832248000	0.761595000	1.141955000
1	1.801252000	0.796094000	2.227966000
1	3.408609000	1.633280000	-1.687873000
78	0.435696000	-0.192118000	0.123777000
6	-0.927531000	-1.995486000	0.561697000
6	-0.843581000	-1.881534000	-0.869872000
1	-0.447807000	-2.505456000	-1.653757000
1	-0.652893000	-2.752067000	1.277722000
1	-2.348776000	-0.267579000	1.341489000
6	-1.764497000	-0.779779000	0.595169000
1	-2.112637000	-0.035635000	-1.570575000
6	-1.661003000	-0.677267000	-0.833053000
6	-4.769361000	1.312363000	-0.101766000
6	-3.997878000	2.217323000	0.036913000
1	-5.483972000	0.535045000	-0.224221000
1	-3.345339000	3.046877000	0.160490000

Table S138. Predicted frequencies (cm^{-1}) and IR intensities (km/mol) of isomer 5p-quartet of $Pt^+(C_2H_2)_5$

Frequency	Intensity	Frequency	Intensity	Frequency	Intensity
10.7433	0.1136	672.6295	4.6057	1209.4239	14.3133
15.4253	0.0927	737.6509	68.3178	1238.9647	6.6214
27.8404	0.1262	767.9224	3.5006	1274.6033	20.8259
38.1419	0.053	773.402	61.5612	1312.1696	22.9938
47.7114	1.4793	773.8368	22.939	1354.537	25.4145
62.7702	5.8208	782.6672	131.1801	1356.2754	61.0981
74.0069	4.1141	803.2841	5.458	1434.2657	50.986
81.0058	0.9182	810.0628	1.2482	1487.4341	83.4358
224.983	3.2185	850.4661	0.1448	2064.5388	5.5408
233.9981	4.8956	896.4777	8.3192	3129.0418	2.9204
244.9016	1.0273	936.5359	0.5134	3159.5856	0.1631
258.3027	0.6503	947.0845	0.1966	3182.4687	0.6287
367.6823	5.0558	956.972	15.3655	3197.9557	2.1785
378.7891	0.2056	971.8533	0.0356	3239.655	0.8539
452.0152	0.3522	981.108	7.6155	3252.0571	21.1745
524.0422	0.0476	1064.2927	0.3277	3255.1735	24.0212
650.3445	7.0882	1087.9867	5.8929	3269.3355	14.728
662.365	0.0128	1117.6789	5.4626	3402.4289	96.6462
671.1505	65.5878	1202.6119	0.3579	3506.5817	1.2042

Table S139. Cartesian coordinates for the optimized geometry of isomer 5q-quartet of $Pt^{+}(C_2H_2)_5$ followed by its predicted frequencies (cm^{-1}) and IR intensities (km/mol).

Z	x	y	z
6	1.412128000	-0.000001000	-0.881947000
6	2.230203000	1.097131000	-0.164334000
6	2.783990000	-0.000003000	0.755736000
6	2.230201000	-1.097136000	-0.164334000
1	1.390778000	-0.000002000	-1.969457000
1	3.042605000	1.540985000	-0.757107000
1	3.552378000	-0.000004000	1.514554000
1	3.042601000	-1.540991000	-0.757108000
78	-0.550405000	0.000001000	-0.152371000
6	1.341488000	2.131286000	0.394901000
1	1.732176000	3.031363000	0.861209000
6	0.009984000	1.895102000	0.269077000
1	-0.758244000	2.632735000	0.489863000
6	-2.834004000	-0.000010000	-0.179631000
6	-2.560751000	0.000007000	1.009599000
1	-2.537108000	0.000020000	2.076922000
1	-3.295769000	-0.000021000	-1.143346000
1	-0.758250000	-2.632727000	0.489879000
6	0.009980000	-1.895100000	0.269083000
6	1.341483000	-2.131290000	0.394901000
1	1.732169000	-3.031369000	0.861204000

Table S140. Predicted frequencies (cm^{-1}) and IR intensities (km/mol) of isomer 5q-quartet of $Pt^+(C_2H_2)_5$

Frequency	Intensity	Frequency	Intensity	Frequency	Intensity
18.682	42.9517	725.08	226.1605	1185.1625	33.3085
88.3621	2.5871	725.7565	44.0757	1204.195	155.7215
99.7175	2.7077	739.3717	8.3297	1227.7289	22.5782
120.2656	3.6833	765.7679	58.7915	1273.4201	9.9746
154.2739	108.3473	797.6125	9.738	1282.8322	1.7577
228.9489	0.3257	806.7378	25.2875	1336.1608	11.6596
234.904	5.7826	835.5689	10.1224	1421.6185	396.6464
285.5298	0.3678	869.7353	152.843	1523.4062	2.5951
314.6242	140.0048	899.3448	10.5265	1937.3836	1.4028
321.7572	7.7113	924.2296	0.0774	2965.5852	0.0903
349.002	29.0827	960.9901	0.0809	2965.8144	0.5258
357.1428	20.1835	966.4089	66.8328	3101.3709	1.9886
424.8066	9.9515	970.1472	4.5262	3114.363	0.1852
482.9343	12.7818	1041.0172	22.2682	3115.9253	0.047
483.0954	1.589	1043.3429	15.2209	3144.4276	0.042
592.06	1.6711	1069.7222	4.2549	3145.4021	1.0236
664.8664	97.6239	1074.3937	15.979	3210.1104	2.9744
671.3834	5.5442	1123.8971	2.5466	3343.4831	154.1345
688.8422	4.9084	1169.0758	15.3647	3430.8189	53.7132

Table S141. Cartesian coordinates for the optimized geometry of isomer 5r-quartet of $Pt^{+}(C_2H_2)_5$ followed by its predicted frequencies (cm^{-1}) and IR intensities (km/mol).

Z	x	y	z
6	2.594880000	0.060990000	1.024179000
6	3.391723000	-0.475864000	-0.000233000
6	2.594816000	0.061948000	-1.024089000
6	1.736569000	0.735896000	0.000390000
1	2.644988000	0.089889000	2.099711000
1	4.325914000	-1.019714000	-0.000520000
1	2.644833000	0.091826000	-2.099598000
1	1.625681000	1.816766000	0.000916000
78	-0.403858000	0.200284000	0.000013000
1	-0.961448000	-1.897752000	2.100471000
6	-0.932579000	-1.780785000	1.030981000
6	0.085854000	-1.919777000	0.000004000
1	1.102950000	-2.272138000	0.000130000
6	-0.932306000	-1.780718000	-1.031240000
1	-0.960884000	-1.897602000	-2.100746000
6	-1.920893000	-1.512750000	-0.000255000
1	-2.986491000	-1.349416000	-0.000393000
6	-1.095693000	2.125436000	0.622307000
6	-1.095305000	2.125586000	-0.622224000
1	-1.248991000	2.487906000	1.618786000
1	-1.248029000	2.488306000	-1.618701000

Table S142. Predicted frequencies (cm^{-1}) and IR intensities (km/mol) of isomer 5r-quartet of $Pt^+(C_2H_2)_5$

Frequency	Intensity	Frequency	Intensity	Frequency	Intensity
30.104	0.0099	720.4506	0.0923	1202.6528	0.0156
34.2359	0.8222	723.0174	91.0039	1214.207	7.1832
81.6934	2.5777	738.5604	67.6202	1215.1654	0.6641
100.4535	1.748	793.231	4.7576	1247.9575	0.0898
126.009	0.3852	798.1073	27.0386	1329.8971	10.5561
131.0609	1.2867	826.477	8.9293	1334.6694	3.9821
148.2354	0.4472	888.3859	16.2412	1342.5406	15.5801
206.1204	0.1015	901.6613	8.2118	1401.8428	60.1586
209.2301	9.9947	902.3253	73.6196	1813.4247	21.7202
273.8872	2.8059	914.4865	23.9041	3116.3193	7.5149
282.7683	2.1899	922.567	8.5944	3203.2853	0.7106
321.2628	6.6389	938.3819	10.2707	3237.6696	10.0403
370.301	7.4668	939.9722	5.262	3243.1672	1.9916
419.743	2.9055	958.1242	0.9157	3251.5207	0.044
507.9927	18.2522	958.46	5.5072	3251.9053	7.5367
521.5447	9.1863	981.1437	6.9594	3259.9091	20.6392
615.7464	0.3574	1035.5517	111.5804	3271.7659	4.2696
632.2924	131.9306	1160.0884	5.5792	3298.0423	93.6284
707.7652	0.6818	1194.2936	0.2996	3367.234	63.8056

Table S143. Cartesian coordinates for the optimized geometry of isomer 5s-quartet of $Pt^{+}(C_2H_2)_5$ followed by its predicted frequencies (cm^{-1}) and IR intensities (km/mol).

Z	x	y	z
78	0.497771000	-0.130452000	0.306652000
6	-0.408873000	1.571443000	-0.333225000
6	0.452748000	2.564480000	-0.761356000
1	0.085402000	3.468730000	-1.234340000
1	-1.490391000	1.642115000	-0.384313000
6	1.823199000	2.338868000	-0.517081000
6	2.120917000	1.099122000	0.052397000
1	3.154486000	0.889276000	0.315708000
1	2.571611000	3.086445000	-0.752783000
1	-1.994396000	-1.174885000	1.182245000
6	-1.134707000	-1.347855000	0.541177000
6	-1.100472000	-2.518329000	-0.220502000
1	-1.902379000	-3.247121000	-0.236232000
6	0.075364000	-2.697901000	-0.978363000
1	0.217605000	-3.543921000	-1.641862000
6	1.055579000	-1.738694000	-0.807578000
1	2.028669000	-1.795959000	-1.280377000
6	-4.203564000	0.891131000	-0.689993000
6	-4.185073000	1.281040000	0.442136000
1	-4.206310000	1.635759000	1.444110000
1	-4.261152000	0.554951000	-1.696680000

Table S144. Predicted frequencies (cm^{-1}) and IR intensities (km/mol) of isomer 5s-quartet of $Pt^+(C_2H_2)_5$

Frequency	Intensity	Frequency	Intensity	Frequency	Intensity
16.3406	0.7684	668.3201	37.9035	1258.4682	28.9286
22.7899	0.4714	675.3268	6.946	1266.0581	3.6532
47.8741	0.1909	697.1569	75.1616	1348.9099	29.374
55.0519	0.9803	762.6732	7.1887	1360.7071	0.5009
62.783	3.827	775.8885	77.125	1460.7787	134.1464
79.5598	0.3204	784.5848	130.8059	1462.7434	11.1379
99.9378	3.3177	785.8187	6.0778	1485.4788	24.1072
132.2146	0.8726	815.8172	2.5652	1493.9779	7.193
185.1119	6.6343	827.8019	1.0364	2062.4917	11.5658
234.2515	1.9868	957.1198	4.4521	3130.3418	0.1494
318.1003	1.2335	966.6549	1.3467	3140.1879	0.3295
343.9125	11.2912	992.875	1.6866	3145.8829	44.3541
346.7432	9.0853	993.4566	1.1654	3157.4769	2.0512
374.5372	0.4885	1037.3452	5.9103	3163.4727	9.6483
445.3759	6.4759	1046.0165	0.8122	3173.7616	0.223
492.6308	0.4971	1102.5049	1.434	3177.0093	2.3908
532.1634	1.8796	1106.7424	23.3052	3180.3851	1.4795
636.6153	2.8387	1128.2303	1.3382	3400.3938	104.6058
664.9621	5.0032	1132.8249	1.0012	3504.5136	0.7686

Table S145. Cartesian coordinates for the optimized geometry of isomer 5t-quartet of $Pt^{+}(C_2H_2)_5$ followed by its predicted frequencies (cm^{-1}) and IR intensities (km/mol).

Z	x	y	z
78	0.681380000	0.097912000	0.343047000
6	2.246740000	-1.224239000	0.248181000
6	3.179324000	-0.948359000	-0.754190000
1	4.042307000	-1.570981000	-0.959330000
1	2.420588000	-2.084808000	0.889161000
6	2.923915000	0.225169000	-1.493337000
6	1.840885000	0.976288000	-1.075746000
1	1.579578000	1.932235000	-1.513597000
1	3.545564000	0.537352000	-2.325306000
6	-2.134243000	0.858666000	0.155251000
6	-0.885505000	1.418946000	0.431772000
1	-0.838499000	2.477044000	0.676707000
1	-3.059329000	1.422373000	0.129997000
1	-3.020480000	-1.075850000	-0.352993000
6	-2.120453000	-0.530814000	-0.088576000
6	-0.896435000	-1.155364000	0.058465000
1	-0.761144000	-2.226491000	-0.032231000
6	-5.775778000	-0.234501000	-1.166617000
6	-5.925283000	-0.446174000	0.002229000
1	-6.094548000	-0.636444000	1.034108000
1	-5.680672000	-0.049232000	-2.208782000

Table S146. Predicted frequencies (cm^{-1}) and IR intensities (km/mol) of isomer 5t-quartet of $Pt^{+}(C_2H_2)_5$

Frequency	Intensity	Frequency	Intensity	Frequency	Intensity
8.8141	0.3345	673.6776	10.1628	1348.718	23.7032
16.2573	0.3645	697.5309	76.4356	1359.8404	2.4466
34.9912	0.143	760.2667	5.0021	1460.6671	127.0923
52.0418	1.5375	772.982	83.2118	1462.6603	10.67
62.1512	3.0083	781.6932	11.8209	1485.7452	34.7425
84.5115	0.3519	783.7021	135.5082	1494.1716	11.1872
95.0916	1.8594	814.6978	1.7582	2064.5729	8.3058
128.9739	0.4128	820.5331	0.0354	3129.9145	1.2997
182.6185	6.7284	959.2201	4.3622	3130.791	0.6056
229.715	2.4441	969.29	1.1064	3154.7057	25.2557
318.4123	1.5724	993.1922	0.9458	3157.8439	2.1609
344.1415	10.2443	1007.4491	0.9467	3173.3186	1.0527
346.7823	11.9998	1036.5799	8.0069	3174.9045	1.464
372.4889	0.1071	1044.7476	0.6912	3179.5728	6.1927
444.7987	8.4188	1100.6622	4.0471	3180.4415	1.444
492.0086	0.5983	1105.0226	12.5509	3402.5196	104.3645
531.0447	3.6546	1126.679	3.0569	3506.5759	1.043
635.7018	3.0801	1131.716	1.5604		
661.8101	0.0314	1256.058	19.2739		
667.1076	40.9702	1264.3247	5.732		

Table S147. Cartesian coordinates for the optimized geometry of isomer 5u-quartet of $Pt^+(C_2H_2)_5$ followed by its predicted frequencies (cm^{-1}) and IR intensities (km/mol).

Z	x	y	z
78	-0.862212000	0.038379000	-0.068642000
6	0.747562000	-0.490113000	-1.224592000
6	1.995027000	-0.264928000	-0.643511000
1	2.926348000	-0.440263000	-1.169103000
1	0.670163000	-0.913967000	-2.221405000
6	1.978494000	0.205078000	0.675292000
6	0.721760000	0.336455000	1.257522000
1	0.599094000	0.655137000	2.286966000
1	2.895290000	0.450686000	1.199426000
6	-2.135071000	-1.765793000	0.636815000
6	-1.118879000	-2.197287000	0.091333000
1	-0.424210000	-2.924909000	-0.269430000
1	-3.069623000	-1.613162000	1.131246000
1	-1.996039000	2.330689000	1.465415000
6	-1.984831000	1.792151000	0.538910000
6	-2.268392000	1.601931000	-0.660855000
1	-2.722202000	1.759568000	-1.619143000
6	5.727275000	0.722068000	-0.193252000
6	5.799330000	-0.380251000	0.268166000
1	5.900373000	-1.355700000	0.677634000
1	5.699729000	1.702465000	-0.602527000

Table S148. Predicted frequencies (cm^{-1}) and IR intensities (km/mol) of isomer 5u-quartet of $Pt^+(C_2H_2)_5$

Frequency	Intensity	Frequency	Intensity	Frequency	Intensity
15.3081	0.1333	572.2773	4.1017	1089.9326	9.9244
16.1619	0.0425	661.8799	0.0058	1123.5568	4.1264
27.825	3.3454	670.4089	2.5045	1220.5424	5.4601
36.3073	0.6659	681.3973	31.0985	1324.2793	23.3193
50.1951	1.1961	690.8467	1.542	1446.8086	66.0217
60.5846	3.0877	704.5054	21.4632	1488.8566	32.7185
71.9415	1.6441	719.1174	19.223	1790.4039	16.2699
83.9875	0.6346	731.25	59.4166	1869.6748	0.5537
110.0064	3.1623	736.4536	33.3018	2064.9761	7.0745
115.6454	4.2173	744.878	46.1471	3139.9272	0.3069
135.3804	0.296	769.4929	33.5018	3153.1225	1.9625
172.8801	5.8794	773.1369	61.3193	3168.4225	0.3864
241.4044	2.5561	780.6456	2.1246	3178.5543	11.3122
267.6464	0.8307	782.5437	134.5644	3292.2237	86.724
306.1161	9.5319	789.6407	13.6967	3335.1341	108.9234
329.4387	1.4394	812.9743	1.8802	3359.0749	59.989
367.9951	5.12	973.4771	0.9799	3403.2143	101.8709
406.5682	0.1411	1013.2036	0.351	3413.4844	54.3142
423.5432	2.1135	1038.1675	0.9309	3507.2434	1.0342

Table S149. Cartesian coordinates for the optimized geometry of isomer 5v-quartet of $Pt^{+}(C_2H_2)_5$ followed by its predicted frequencies (cm^{-1}) and IR intensities (km/mol).

Z	x	y	z
6	3.157118000	0.698722000	-0.007943000
6	2.546372000	-0.696866000	-0.207665000
6	1.549751000	0.071377000	-1.116075000
6	2.242510000	1.297142000	-0.826081000
1	4.031075000	1.038212000	0.528285000
1	3.215986000	-1.381605000	-0.734134000
1	1.195741000	-0.251564000	-2.088951000
1	2.126721000	2.309543000	-1.184334000
78	-0.498186000	-0.042109000	0.038373000
6	1.931628000	-1.346208000	0.981272000
1	2.573731000	-1.930325000	1.635743000
6	0.621805000	-1.207370000	1.255058000
1	0.160193000	-1.684378000	2.113137000
6	-2.278746000	-1.125266000	-0.555168000
6	-1.781397000	-0.737603000	-1.627764000
1	-1.651613000	-0.620377000	-2.683939000
1	-2.980577000	-1.608706000	0.094470000
1	-2.057506000	1.239955000	2.177918000
6	-1.501573000	1.453796000	1.287684000
6	-0.997505000	2.126096000	0.375794000
1	-0.694975000	2.970827000	-0.205918000

Table S150. Predicted frequencies (cm^{-1}) and IR intensities (km/mol) of isomer 5v-quartet of $Pt^+(C_2H_2)_5$

Frequency	Intensity	Frequency	Intensity	Frequency	Intensity
45.2059	0.0268	709.428	16.1202	1183.8247	3.9123
59.2369	3.0987	712.1045	16.1528	1209.6553	3.1272
82.7849	2.4917	719.1641	14.7101	1274.5487	10.1453
95.8429	0.7268	723.3322	27.8179	1319.0196	8.5567
109.8405	1.7625	732.1508	38.527	1337.996	32.5284
119.1166	2.3901	740.1448	34.1455	1464.4989	23.9279
142.7568	2.4975	746.6213	6.7323	1532.8122	65.2238
161.7088	0.5879	765.7705	5.1288	1800.0436	11.8977
211.6794	4.621	798.6863	103.5858	1824.7989	12.9255
233.7614	0.7356	838.2412	21.9138	3045.4445	1.34
258.4369	0.8769	865.5479	2.5081	3121.7558	0.5969
282.7026	2.9501	887.4543	1.8192	3145.6697	3.5379
321.9095	1.7327	905.2077	6.0113	3148.7682	0.2601
334.6396	5.352	935.9204	8.6932	3201.3562	0.8752
389.1957	1.6057	955.423	1.0293	3225.3757	0.4747
448.1012	4.6381	1018.3857	5.3866	3301.6833	99.9577
532.9103	11.3665	1029.5669	2.5512	3310.4341	103.2821
677.3313	21.19	1112.5166	8.7559	3372.4471	70.3856
690.8792	16.4649	1137.274	5.6943	3385.6596	55.7085

Table S151. Cartesian coordinates for the optimized geometry of isomer 5x-quartet of $Pt^+(C_2H_2)_5$ followed by its predicted frequencies (cm^{-1}) and IR intensities (km/mol).

Z	x	y	z
78	0.000000000	0.342652000	-0.000001000
1	2.659244000	-0.914412000	0.506774000
6	0.735676000	-0.695451000	1.729307000
6	1.671245000	-0.697355000	0.887371000
1	0.279752000	-0.922385000	2.674875000
1	-2.659248000	-0.914405000	-0.506773000
6	-0.735681000	-0.695448000	-1.729308000
6	-1.671250000	-0.697349000	-0.887372000
1	-0.279757000	-0.922388000	-2.674875000
1	1.177772000	2.864085000	-1.075832000
6	0.465875000	2.389311000	-0.425696000
6	-0.465865000	2.389313000	0.425694000
1	-1.177754000	2.864091000	1.075836000
1	-5.579208000	-0.064701000	-0.450923000
6	-5.191362000	-0.948293000	-0.004762000
6	-4.797608000	-1.958165000	0.504386000
1	-4.487414000	-2.866326000	0.961728000
6	5.191362000	-0.948304000	0.004787000
6	4.797606000	-1.958157000	-0.504398000
1	4.487412000	-2.866300000	-0.961775000
1	5.579209000	-0.064729000	0.450979000

Table S152. Predicted frequencies (cm^{-1}) and IR intensities (km/mol) of isomer 5x-quartet of $Pt^+(C_2H_2)_5$

Frequency	Intensity	Frequency	Intensity	Frequency	Intensity
9.4317	0.0019	384.6809	0.2411	811.1534	4.7041
10.7925	0.0011	393.3404	1.0206	811.6536	64.0144
17.8694	0.0357	442.6985	0.0404	821.4106	23.4175
19.3988	0.1822	660.2619	0.0097	867.618	0.0441
30.9044	0.0342	660.2861	0.0006	1686.2356	8.2013
35.6222	0.1513	674.7483	0.5731	1698.325	10.629
55.5801	3.0919	675.7143	8.3937	1782.8186	1.4374
80.3631	2.5133	679.8243	0.2961	2063.0696	19.8065
83.6259	0.7212	679.843	5.8747	2063.2562	2.1463
86.1402	7.4963	714.6033	11.2558	3182.2277	1003.5902
93.0602	18.4119	721.3939	53.0083	3187.0031	75.2085
95.3961	3.2885	734.8899	81.067	3257.6372	75.5058
101.6513	2.1465	751.3066	15.3457	3305.4841	109.3
128.6132	0.0436	771.3653	106.7102	3308.1431	48.5374
183.0637	0.4427	771.4728	32.1431	3318.4539	31.4802
191.6124	0.33	789.6148	260.4313	3399.8339	61.7624
251.9014	6.6886	790.805	5.781	3399.8951	150.9532
293.757	3.3831	791.4728	46.8125	3503.5734	1.4173
309.1674	3.1902	793.6497	26.9722	3503.5962	0.1731

Table S153. Cartesian coordinates for the optimized geometry of isomer 5y-quartet of $Pt^+(C_2H_2)_5$ followed by its predicted frequencies (cm^{-1}) and IR intensities (km/mol).

Z	x	y	z
78	0.395770000	-0.089300000	0.129040000
1	2.284642000	0.277112000	-2.181762000
6	2.721719000	-0.689134000	-0.175843000
6	2.364608000	-0.151325000	-1.208203000
1	3.203147000	-1.152814000	0.654980000
1	-2.123871000	-0.513177000	-1.330252000
6	-0.278430000	-1.840926000	-1.408024000
6	-1.182345000	-1.026080000	-1.296111000
1	0.424128000	-2.610050000	-1.637432000
1	-0.426170000	2.499876000	0.175394000
6	0.475093000	1.928727000	-0.069546000
6	1.598057000	2.581405000	-0.341757000
1	1.924060000	3.614082000	-0.367135000
1	-2.075121000	0.246368000	1.614287000
6	-1.082191000	-0.162364000	1.638488000
6	-0.144373000	-0.800431000	2.160583000
1	0.427285000	-1.329396000	2.894304000
6	-4.222407000	1.038121000	-0.595119000
6	-4.527934000	-0.056138000	-0.215437000
1	-4.840300000	-1.017418000	0.115567000
1	-3.998638000	2.019697000	-0.937287000

Table S154. Predicted frequencies (cm^{-1}) and IR intensities (km/mol) of isomer 5y-quartet of $Pt^+(C_2H_2)_5$

Frequency	Intensity	Frequency	Intensity	Frequency	Intensity
19.2922	0.0559	288.6369	2.5889	839.2893	30.6485
29.4839	0.0277	405.5109	1.4478	855.9325	11.0824
37.6192	0.2673	492.021	10.3352	865.8258	1.761
50.6236	0.0524	553.1117	48.7049	1157.4962	5.9188
68.7149	0.2618	660.3391	1.7194	1471.2084	126.0209
70.3775	1.3727	668.2542	0.0345	1777.2668	3.7942
73.283	2.6489	678.0502	1.449	1913.7139	8.6683
92.727	0.8094	686.0997	11.0692	1943.9617	1.4539
99.1628	3.5086	697.6596	34.0559	2061.341	9.0683
117.7523	0.3992	705.7226	15.4394	3004.2328	5.5412
122.5886	3.0245	706.8104	5.4828	3178.3614	0.3621
128.3944	2.0967	728.5112	17.389	3278.6741	66.7891
148.2669	3.8147	748.952	40.094	3293.9768	340.4667
152.8248	2.2389	755.7759	42.9832	3361.8341	120.5743
179.8898	3.9266	777.2809	16.5832	3366.1479	75.067
190.6608	3.9678	778.4574	66.9523	3395.9908	106.2299
212.0374	2.9827	786.5611	137.1345	3422.0221	52.7971
249.3267	4.678	791.775	72.9519	3448.0307	38.1107
269.6961	3.5523	812.9825	6.3563	3500.3521	1.23

Table S155. Cartesian coordinates for the optimized geometry of isomer 5z-quartet of $Pt^{+}(C_2H_2)_5$ followed by its predicted frequencies (cm^{-1}) and IR intensities (km/mol).

Z	x	y	z
78	-0.000005000	-0.682996000	-0.000001000
1	-3.925846000	2.911374000	1.661076000
6	-3.892605000	2.886969000	-0.598758000
6	-3.892707000	2.886797000	0.598781000
1	-3.925559000	2.911855000	-1.661052000
1	1.595017000	-1.918121000	2.197531000
6	-0.631078000	-1.679538000	1.786363000
6	0.631220000	-1.679546000	1.786308000
1	-1.594843000	-1.918104000	2.197664000
1	1.624524000	1.807706000	-0.000042000
6	0.627364000	1.392374000	-0.000016000
6	-0.627352000	1.392377000	0.000020000
1	-1.624508000	1.807718000	0.000046000
1	3.925598000	2.911757000	1.661056000
6	3.892629000	2.886924000	0.598761000
6	3.892720000	2.886803000	-0.598778000
1	3.925852000	2.911423000	-1.661072000
6	0.631079000	-1.679546000	-1.786360000
6	-0.631219000	-1.679555000	-1.786306000
1	-1.595016000	-1.918131000	-2.197528000
1	1.594843000	-1.918114000	-2.197661000

Table S156. Predicted frequencies (cm^{-1}) and IR intensities (km/mol) of isomer 5z-quartet of $Pt^{+}(C_2H_2)_5$

Frequency	Intensity	Frequency	Intensity	Frequency	Intensity
6.5496	0	377.7478	2.559	802.4441	74.854
12.6092	0.0145	398.6114	0	812.2292	0
15.4868	0.0522	442.691	0.0756	818.2631	30.6029
24.6353	0	660.2063	0	864.6763	0.0149
30.1164	0.6084	660.2702	0.0769	1679.6978	15.3883
30.5443	0.0011	668.4905	2.6275	1705.3105	0.176
56.6909	4.4049	678.0456	32.2297	1784.0684	7.6763
68.2265	3.2809	678.4376	0.0002	2063.0934	12.3242
85.0332	0	678.7026	0	2063.3514	8.3629
85.5802	4.8147	702.1532	39.1508	3164.4822	704.8069
87.8922	6.1876	738.0116	0	3240.1046	372.071
102.8378	17.3377	743.1536	101.6602	3256.9389	152.0279
118.384	0	758.9326	0.1795	3257.8163	0
118.4836	0.4774	770.7148	24.5584	3316.7422	68.2872
175.6843	0.114	772.4807	101.1264	3318.9778	17.2674
200.8749	0	786.516	0	3399.8278	0.0003
253.4366	6.0691	789.4681	231.4392	3399.9846	210.4633
280.7103	2.8552	791.836	69.275	3503.5573	0.8608
320.2132	2.6147	801.238	32.8162	3503.6141	0.7036

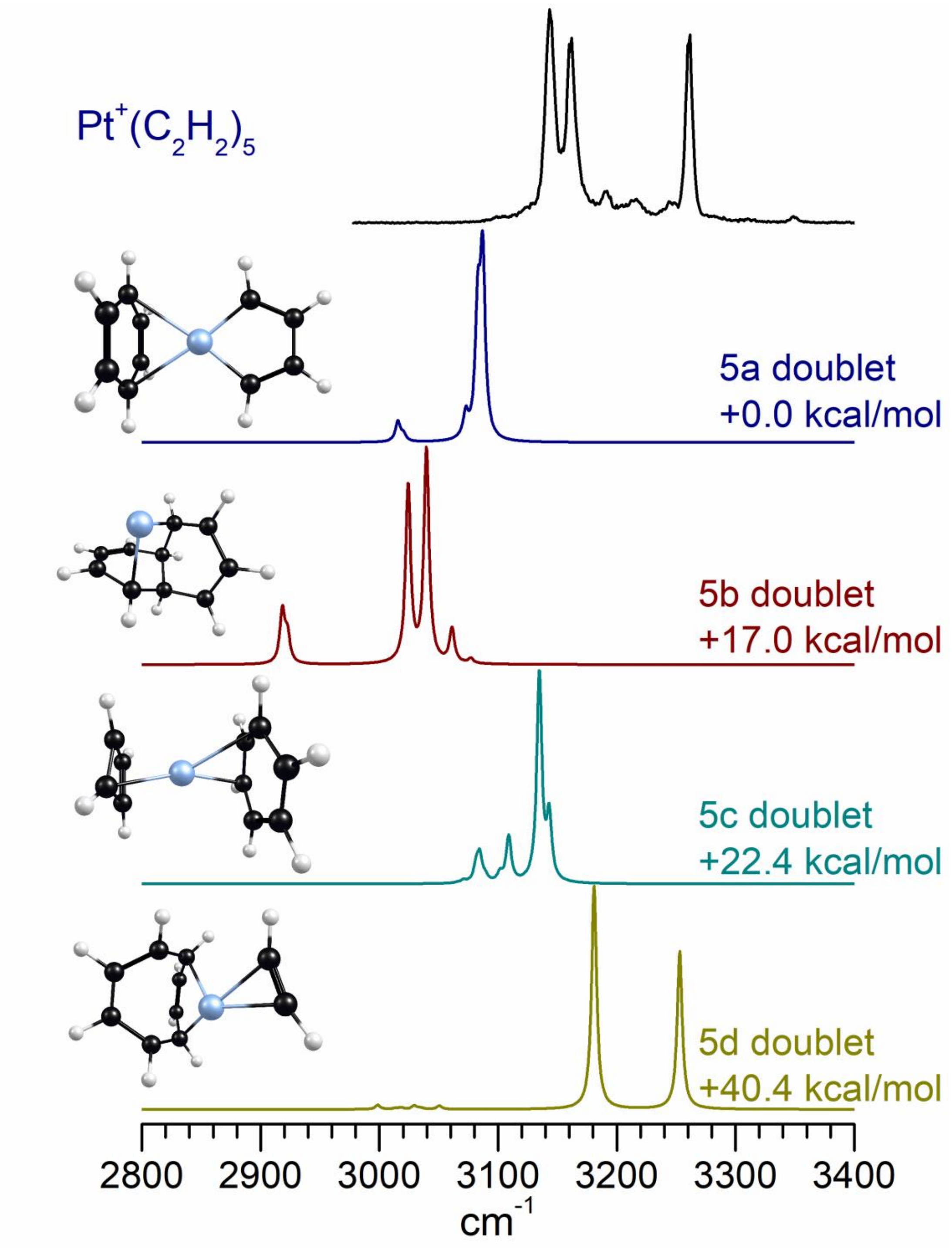

Pt+(C2H2)5
5a doublet
+0.0 kcal/mol
5b doublet
+17.0 kcal/mol
5c doublet
+22.4 kcal/mol
5d doublet
+40.4 kcal/mol
2800
2900
3000
3100
3200
3300
3400
cm-1

S60

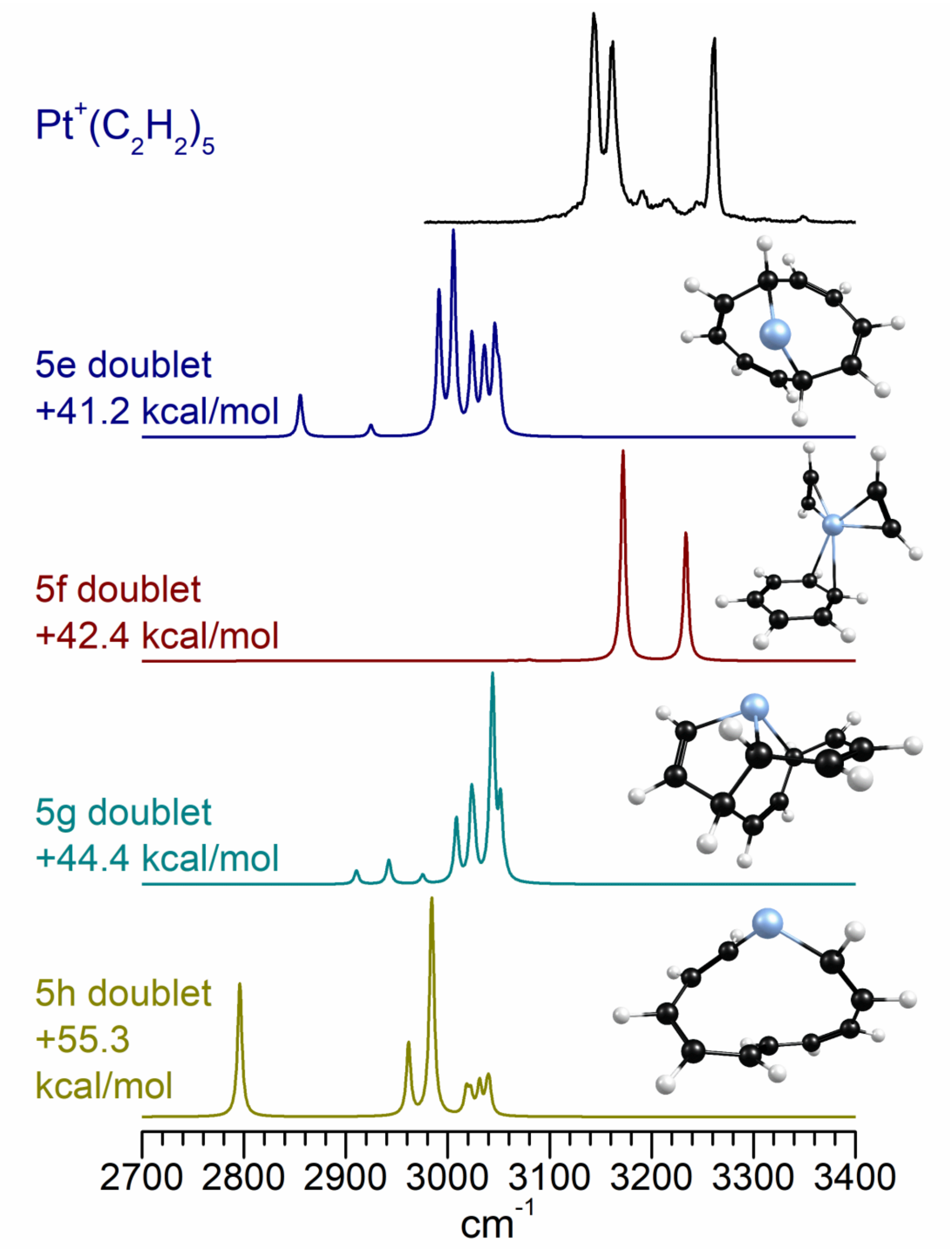

$Pt^+(C_2H_2)_5$
5e doublet
+41.2 kcal/mol
5f doublet
+42.4 kcal/mol
5g doublet
+44.4 kcal/mol
5h doublet
+55.3
kcal/mol
2700 2800 2900 3000 3100 3200 3300 3400
cm^{-1}

S61

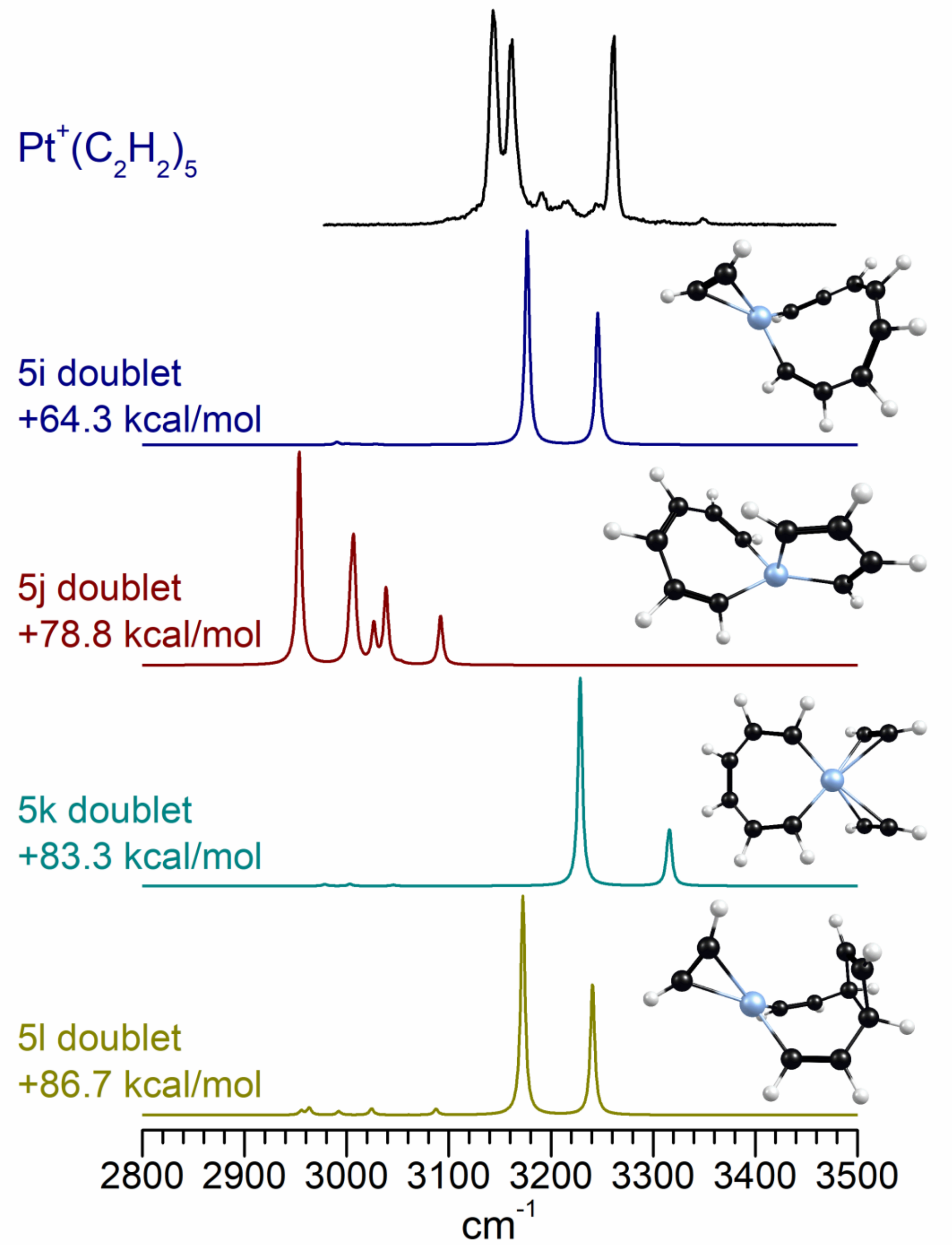
$Pt^+(C_2H_2)_5$
5i doublet
+64.3 kcal/mol
5j doublet
+78.8 kcal/mol
5k doublet
+83.3 kcal/mol
5l doublet
+86.7 kcal/mol
2800 2900 3000 3100 3200 3300 3400 3500
cm^{-1}

S62

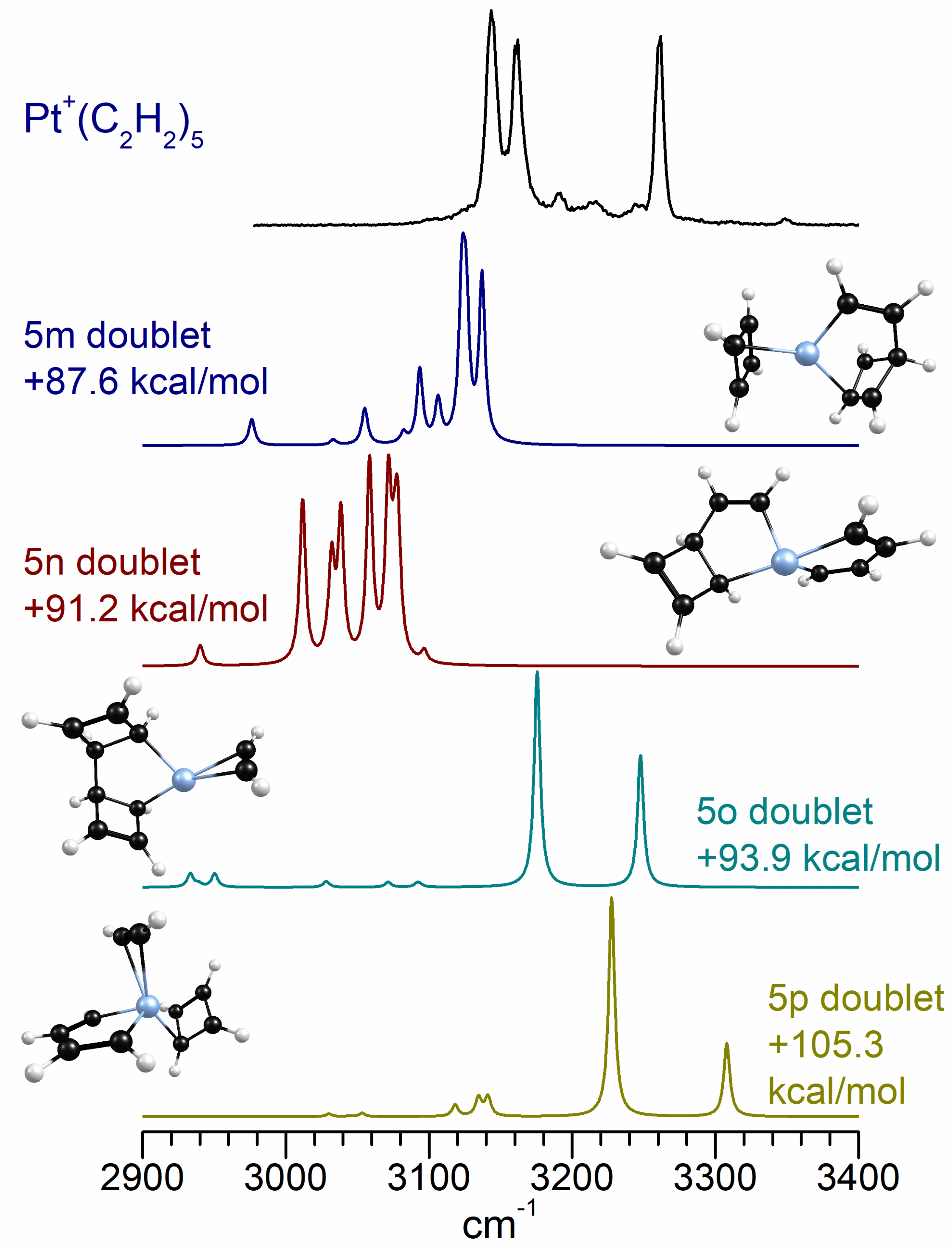
Pt+(C2H2)5
5m doublet
+87.6 kcal/mol
5n doublet
+91.2 kcal/mol
5o doublet
+93.9 kcal/mol
5p doublet
+105.3
kcal/mol
2900
3000
3100
3200
3300
3400
cm-1

S63

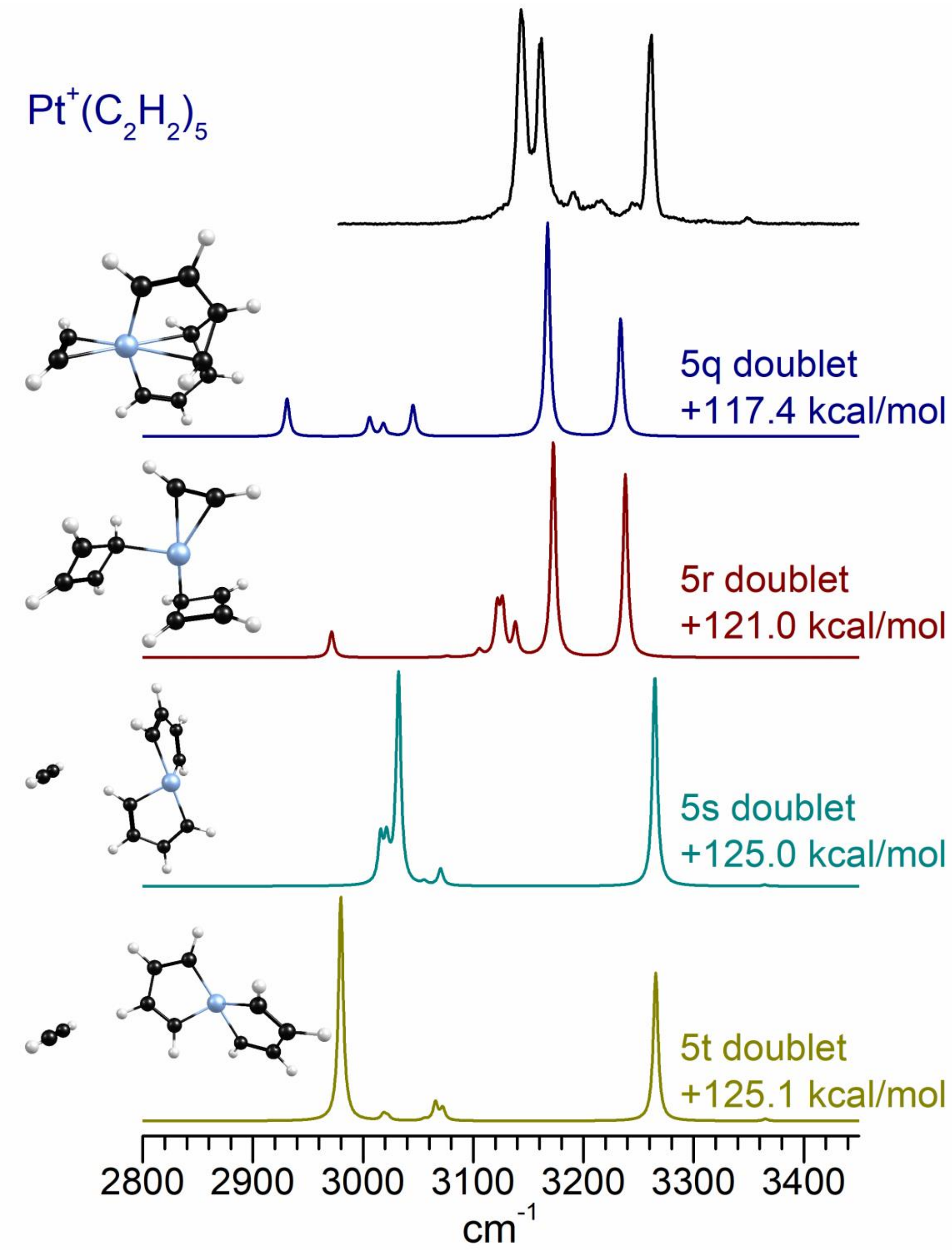

Pt+(C2H2)5
5q doublet
+117.4 kcal/mol
5r doublet
+121.0 kcal/mol
5s doublet
+125.0 kcal/mol
5t doublet
+125.1 kcal/mol
2800
2900
3000
3100
3200
3300
3400
cm-1

S64

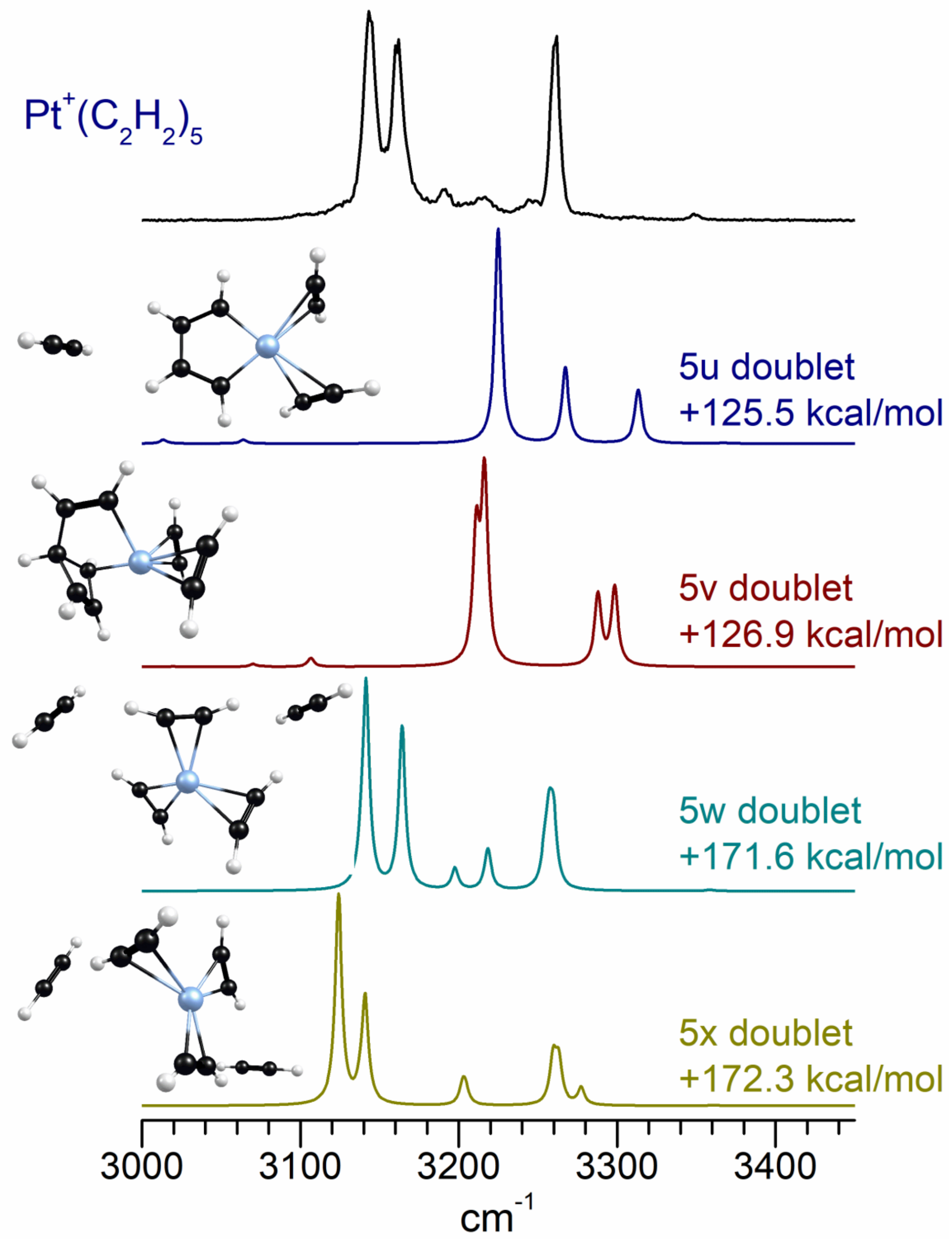

$Pt^+(C_2H_2)_5$
5u doublet
+125.5 kcal/mol
5v doublet
+126.9 kcal/mol
5w doublet
+171.6 kcal/mol
5x doublet
+172.3 kcal/mol
3000
3100
3200
3300
3400
cm^{-1}

S65

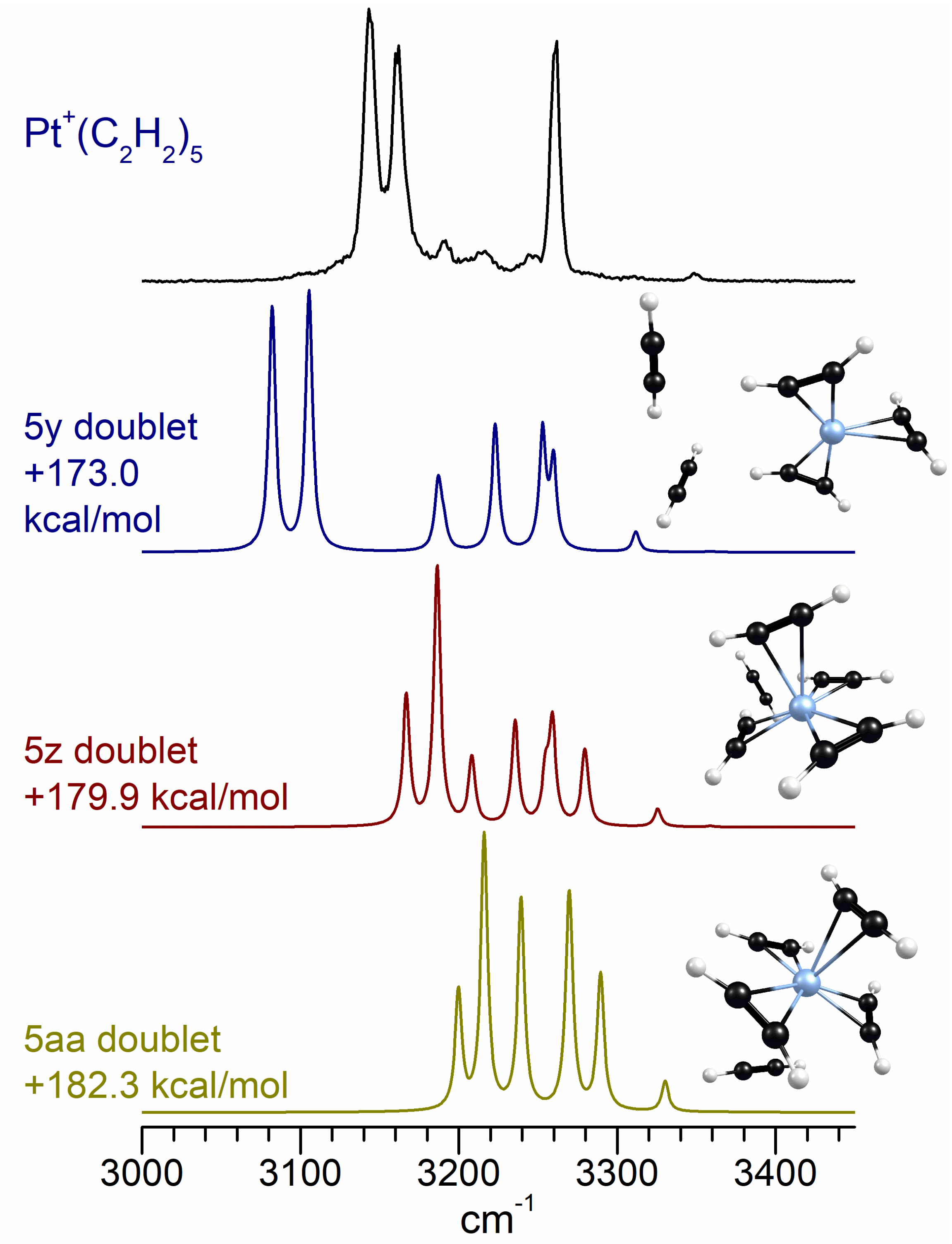

$Pt^+(C_2H_2)_5$
5y doublet
+173.0
kcal/mol
5z doublet
+179.9 kcal/mol
5aa doublet
+182.3 kcal/mol
3000
3100
3200
3300
3400
cm^{-1}

S66

Table S157. $V^+(C_2H_2)_6$ calculated at the B3LYP/Def2TZVP level of theory using Gaussian09.

Isomer	2s + 1	E (hartree)	Relative E (kcal/mol)
6a	2	-583.340709	0
6a	4	-583.258165	51.79715309
6b	2	-583.339991	0.450551898
6b	4	-583.258151	51.80593822
6c	2	-583.328396	7.726525804
6c	4	-583.237823	64.56195353
6d	2	-583.327857	8.064753482
6d	4	-583.237015	65.0689813
6e	2	-583.325977	9.244471545
6f	2	-583.324375	10.24974194

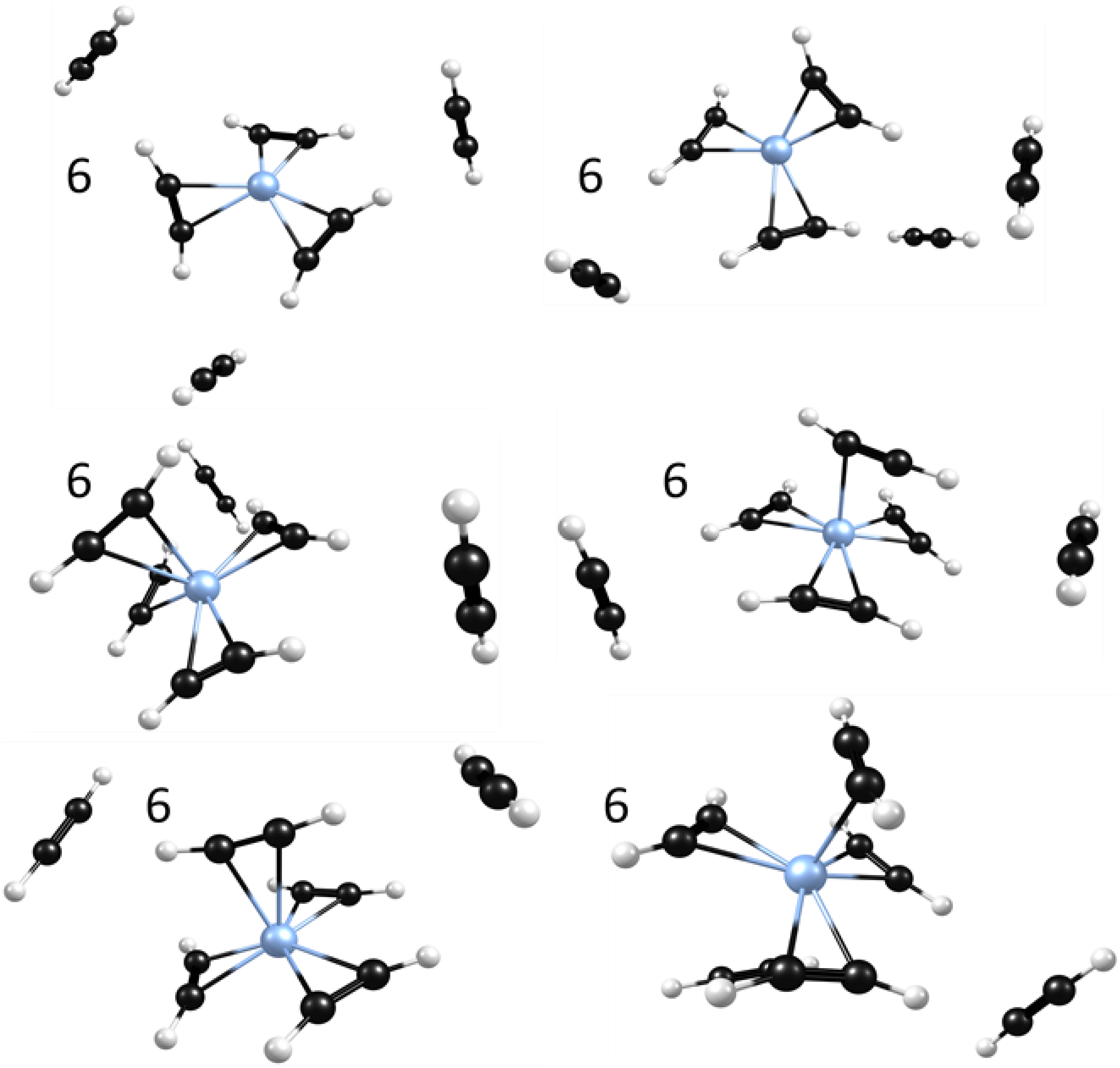

S67

Table S158. Cartesian coordinates for the optimized geometry of isomer 6a-doublet of $Pt^{+}(C_2H_2)_5$
followed by its predicted frequencies (cm^{-1}) and IR intensities (km/mol).

Z	x	y	z
78	0.059315000	-0.054549000	0.078258000
1	-2.678628000	-0.985413000	-0.467121000
6	-1.903300000	1.097750000	0.049725000
6	-2.173659000	-0.067879000	-0.224982000
1	-1.929189000	2.152806000	0.233627000
1	2.677110000	0.251751000	-1.172882000
6	1.050721000	1.752248000	-0.628486000
6	1.806607000	0.795791000	-0.862085000
1	0.669036000	2.753523000	-0.581258000
1	2.227201000	-1.617838000	1.234604000
6	1.175941000	-1.414251000	1.148019000
6	-0.043243000	-1.578589000	1.455429000
1	-0.827102000	-2.041855000	2.023373000
1	4.117741000	-2.679155000	-1.396398000
6	4.464385000	-1.906606000	-0.753245000
6	4.899722000	-1.053536000	-0.033894000
1	5.324163000	-0.310935000	0.597958000
6	-4.239400000	-3.019573000	-0.138457000
6	-3.476080000	-3.414008000	-0.972754000
1	-2.826108000	-3.795707000	-1.722390000
1	-4.941461000	-2.698344000	0.592695000
1	-0.629440000	4.750927000	1.607637000
6	-0.945130000	4.711231000	0.592731000
6	-1.310765000	4.716219000	-0.548144000
1	-1.644683000	4.762275000	-1.557078000

Table S159. Predicted frequencies (cm^{-1}) and IR intensities (km/mol) of isomer 6a-doublet of $Pt^{+}(C_2H_2)_5$

Frequency	Intensity	Frequency	Intensity	Frequency	Intensity
8.0494	0.035	357.444	10.2937	824.1438	28.2364
15.0283	0.0057	434.9673	5.8402	835.8985	8.2297
18.6413	0.0494	446.8577	4.4808	850.2727	15.1339
25.6232	0.0049	547.6536	3.3467	863.5335	4.4972
27.2714	0.3068	663.1513	0.029	890.7744	49.7739
32.331	0.1541	669.5259	0.0136	1706.5026	1.1475
42.6353	0.3585	670.9244	0.0155	1825.0624	4.5571
50.5045	0.052	678.3617	0.2769	1891.7105	11.7568
53.1731	0.0601	679.4418	0.4878	2060.5064	11.7899
62.8646	0.2891	681.0992	0.2731	2061.653	8.9187
69.6418	0.585	736.7258	4.6763	2062.8535	7.11
70.4017	0.4874	741.6393	31.9163	3250.9109	526.2255
82.4635	1.6456	749.7539	14.3254	3270.5411	67.6565
88.5374	0.7745	773.5199	63.8108	3274.5918	362.9441
92.3783	3.0785	776.2491	27.5078	3330.8474	40.6405
99.3539	2.5996	779.8161	50.8334	3348.955	85.1099
109.3521	2.3738	780.8958	79.1609	3357.614	90.0152
121.7918	2.9494	782.358	52.8599	3393.6779	113.9706
155.1847	2.687	787.7429	154.2844	3396.8592	109.6277
172.4226	15.2067	789.4064	252.132	3398.8403	114.7327
189.5437	6.3019	792.1563	13.8886	3497.9224	1.2779
235.4623	2.5573	792.8015	26.6544	3501.2971	1.0918
324.3974	1.9564	810.5269	44.1557	3502.7739	0.8085

Table S160. Cartesian coordinates for the optimized geometry of isomer 6b-doublet of $Pt^{+}(C_2H_2)_5$
followed by its predicted frequencies (cm^{-1}) and IR intensities (km/mol).

Z	x	y	z
78	0.448497000	-0.553554000	-0.227234000
1	-4.343338000	1.866804000	-0.291877000
6	-3.430915000	2.200036000	-2.334242000
6	-3.900998000	2.021595000	-1.247075000
1	-3.046363000	2.372744000	-3.310300000
1	3.313476000	0.103696000	-0.062340000
6	2.137842000	-1.543971000	-1.118856000
6	2.608691000	-0.589080000	-0.480032000
1	2.120352000	-2.406539000	-1.752028000
1	1.444121000	2.054102000	0.777703000
6	0.584243000	1.656288000	0.277641000
6	-0.479828000	1.496814000	-0.314765000
1	-1.434398000	1.646402000	-0.788723000
1	3.952023000	1.532251000	2.754154000
6	4.025042000	2.032669000	1.818453000
6	4.146354000	2.623660000	0.783396000
1	4.286093000	3.171559000	-0.117637000
6	-4.208997000	0.301178000	2.432025000
6	-4.732192000	-0.405872000	1.619062000
1	-5.226307000	-1.031743000	0.915537000
1	-3.778348000	0.927574000	3.175326000
1	-2.132549000	-0.891780000	1.116362000
6	-1.246569000	-1.314498000	0.672443000
6	-0.488385000	-2.285887000	0.378135000
1	-0.223247000	-3.325435000	0.390937000

Table S161. Predicted frequencies (cm^{-1}) and IR intensities (km/mol) of isomer 6b-doublet of $Pt^+(C_2H_2)_5$

Frequency	Intensity	Frequency	Intensity	Frequency	Intensity
13.7146	0.0821	354.6787	11.8076	824.05	44.0235
19.6468	0.0421	432.8341	4.375	832.7584	20.6843
20.8343	0.0253	446.7026	4.2619	857.1981	4.6999
23.9308	0.3002	542.4206	6.3013	862.7189	14.5679
35.1021	0.1876	667.0501	0.7605	904.9832	26.3168
37.5325	0.006	671.0695	0.019	1711.5547	7.2528
46.2057	0.2187	673.4487	0.9371	1824.883	1.8336
52.1196	0.5176	679.1539	0.2098	1888.747	13.9479
62.8093	1.0436	680.9205	0.2029	2060.3963	15.5006
70.3138	0.6805	685.9507	1.719	2060.747	3.9205
74.598	0.9389	729.2961	10.8857	2062.2719	4.7982
79.6419	0.8151	740.9365	8.9004	3232.6727	100.5709
80.5895	0.4499	745.0596	20.3773	3237.4156	734.1012
83.332	1.0491	776.8658	138.7167	3286.22	225.0255
96.4843	2.3326	780.6185	42.7707	3322.32	53.08
98.9885	2.0319	781.6989	20.0338	3352.4348	109.9986
111.938	3.4458	782.0158	65.9645	3372.1535	50.0314
124.0937	2.0429	787.5381	21.9639	3392.1036	124.3172
153.8507	7.1742	787.991	137.2547	3393.6076	128.5727
173.486	11.4502	791.3845	155.1809	3397.8028	111.6893
192.6978	1.2301	799.2836	133.1986	3497.5065	0.3864
237.8733	0.7143	804.2332	8.4241	3497.8971	1.1712
326.8932	1.7099	808.251	12.1714	3501.8481	0.9568

Table S162. Cartesian coordinates for the optimized geometry of isomer 6c-doublet of $Pt^{+}(C_2H_2)_5$
followed by its predicted frequencies (cm^{-1}) and IR intensities (km/mol).

Z	x	y	z
78	0.403065000	-0.152505000	0.000000000
1	-2.095265000	0.096068000	1.630870000
6	-1.754114000	0.071810000	-0.616002000
6	-1.754114000	0.071810000	0.616002000
1	-2.095265000	0.096068000	-1.630870000
1	2.823106000	-1.233391000	1.427118000
6	0.837753000	-0.475843000	2.205300000
6	1.846615000	-0.841794000	1.605626000
1	0.136983000	-0.269512000	2.987399000
1	2.823106000	-1.233391000	-1.427118000
6	1.846615000	-0.841794000	-1.605626000
6	0.837753000	-0.475843000	-2.205300000
1	0.136983000	-0.269512000	-2.987399000
1	-0.297592000	2.703514000	0.000000000
6	0.651084000	2.216765000	0.000000000
6	1.837852000	1.957970000	0.000000000
1	2.892879000	1.816227000	0.000000000
6	-2.123172000	0.594662000	4.383057000
6	-2.289457000	-0.588148000	4.292604000
1	-2.456488000	-1.637542000	4.247311000
1	-1.998055000	1.643882000	4.502353000
1	-2.456488000	-1.637542000	-4.247311000
6	-2.289457000	-0.588148000	-4.292604000
6	-2.123172000	0.594662000	-4.383057000
1	-1.998055000	1.643882000	-4.502353000

Table S163. Predicted frequencies (cm^{-1}) and IR intensities (km/mol) of isomer 6c-doublet of $Pt^{+}(C_2H_2)_5$

Frequency	Intensity	Frequency	Intensity	Frequency	Intensity
23.4845	0.0323	307.9333	0.8434	791.6707	63.0711
26.9793	0.2775	360.9018	2.1039	798.6825	2.8942
29.2423	0.1793	361.0765	6.2299	818.9668	1.1512
48.259	0.6216	372.1882	4.3523	853.774	13.5105
50.3162	0.1434	663.2037	1.658	862.352	3.713
54.432	0.1768	668.2485	0.0219	1873.2143	2.0305
62.8356	0.0453	668.3588	0.0113	1886.1012	1.9857
65.167	0.0869	679.5699	0.0044	1895.2916	9.2367
65.8523	0.1508	679.8917	0.1853	1965.5622	5.7729
88.4004	1.3915	694.7658	1.2089	2061.4509	14.9434
89.5846	5.2856	699.7725	24.6674	2061.675	5.0861
95.3951	5.662	716.3534	5.8009	3286.2866	278.2973
101.39	17.0576	745.4661	1.7246	3315.5219	252.4202
102.3284	3.143	747.0554	22.2593	3315.598	204.5075
118.5685	4.6048	754.7334	44.824	3367.8792	99.5052
125.9795	0.1576	767.7128	50.9572	3371.6207	112.4012
150.9605	0.4062	769.7969	6.786	3395.8801	1.2472
153.1856	2.4527	776.9004	34.682	3396.0076	223.6143
159.2011	1.8888	777.8335	32.081	3405.912	80.7468
169.5324	0.0511	781.6321	132.2126	3407.3379	18.3387
193.7375	0.3456	787.4464	84.9901	3465.0536	21.0567
236.8141	0.2948	789.6802	140.5248	3499.8156	1.7677
237.7854	0.0043	790.0075	86.4682	3499.8688	0.5911

Table S164. Cartesian coordinates for the optimized geometry of isomer 6d-doublet of $Pt^{+}(C_2H_2)_5$
followed by its predicted frequencies (cm^{-1}) and IR intensities (km/mol).

Z	x	y	z
78	0.057854000	-0.516176000	-0.150092000
1	2.996320000	-0.247637000	0.337016000
6	1.570801000	-2.014196000	0.438721000
6	2.181907000	-0.943829000	0.336994000
1	1.419861000	-3.057884000	0.610459000
1	-1.253364000	1.730581000	-1.682083000
6	0.800449000	1.597918000	-0.735489000
6	-0.333017000	1.510574000	-1.190533000
1	1.782652000	1.934098000	-0.477062000
1	-2.642914000	-0.587596000	-1.482828000
6	-1.947162000	-1.193894000	-0.946696000
6	-1.445750000	-2.171473000	-0.392985000
1	-1.320796000	-3.168303000	-0.032739000
1	0.337161000	-0.444997000	2.780447000
6	-0.411281000	-0.033873000	2.141955000
6	-1.387617000	0.532158000	1.690853000
1	-2.262590000	1.044336000	1.349537000
6	4.313400000	2.217539000	0.605712000
6	4.529049000	1.892526000	-0.526900000
1	4.754897000	1.624598000	-1.531138000
1	4.161212000	2.528300000	1.611236000
1	-3.428480000	3.613410000	0.010370000
6	-3.945685000	2.686776000	0.078709000
6	-4.573046000	1.669816000	0.161817000
1	-5.168871000	0.792557000	0.241001000

Table S165. Predicted frequencies (cm^{-1}) and IR intensities (km/mol) of isomer 6d-doublet of $Pt^+(C_2H_2)_5$

Frequency	Intensity	Frequency	Intensity	Frequency	Intensity
12.7362	0.0179	316.1662	1.9088	795.903	23.515
23.2275	0.12	333.6544	3.0204	815.903	10.8616
29.5864	0.0846	370.7757	2.805	826.56	18.0315
44.5718	0.1614	402.5254	4.5369	837.1505	9.001
48.8689	0.073	667.9225	0.0272	860.7553	8.9192
52.4062	0.0749	668.6589	0.0273	1853.0811	5.7099
64.2006	0.2352	675.2903	0.8042	1887.6868	7.9114
64.9157	0.2239	679.9157	0.3189	1914.3436	4.0574
81.0015	0.0355	688.7991	2.8572	1958.4743	19.8312
89.2361	9.427	695.2058	3.4617	2061.3743	12.0058
91.4802	1.4911	696.3684	9.9161	2061.697	5.8815
94.5298	7.7394	711.3828	3.0567	3299.5797	141.3028
98.1828	1.8929	726.736	91.3833	3321.139	235.4203
104.1305	6.2395	740.1154	17.6249	3324.3305	367.8215
127.1985	7.3179	744.8057	12.7429	3344.5075	93.7161
130.1101	2.6069	763.2929	2.9128	3386.9724	55.071
143.4451	0.4007	767.3445	12.4638	3395.5285	112.9264
148.3117	0.0503	778.434	69.4805	3396.3002	106.2274
159.103	1.8075	778.5885	22.753	3418.4941	58.9203
165.3821	1.8624	779.2935	145.5415	3420.4241	21.6078
194.1908	0.2715	788.1137	33.2108	3432.0214	16.5709
225.3238	0.0302	789.493	152.7098	3499.438	1.204
247.5951	0.8419	791.3556	113.5873	3500.5545	1.1814

Table S166. Cartesian coordinates for the optimized geometry of isomer 6e-doublet of $Pt^{+}(C_2H_2)_5$
followed by its predicted frequencies (cm^{-1}) and IR intensities (km/mol).

Z	x	y	z
78	0.020328000	-0.645271000	0.097268000
1	1.998603000	-1.953946000	-1.714685000
6	2.133421000	-1.160925000	0.400472000
6	1.846317000	-1.519652000	-0.750235000
1	2.743793000	-1.009314000	1.264576000
1	-2.916001000	-0.956341000	-0.558859000
6	-1.057023000	-1.321325000	-1.801462000
6	-1.951932000	-1.080438000	-0.998396000
1	-0.543398000	-1.610722000	-2.690265000
1	-2.339050000	-0.176826000	1.929026000
6	-1.272519000	-0.181106000	1.930766000
6	-0.107042000	-0.097915000	2.304264000
1	0.751347000	0.041116000	2.921971000
1	1.720929000	1.693154000	-0.660890000
6	0.657596000	1.595565000	-0.576226000
6	-0.545969000	1.757365000	-0.532424000
1	-1.590924000	1.984484000	-0.520543000
6	4.022533000	2.812001000	0.110048000
6	4.418348000	2.008461000	-0.684560000
1	4.806856000	1.317340000	-1.392310000
1	3.702905000	3.544715000	0.810806000
1	-4.428350000	1.695862000	-1.813635000
6	-4.265141000	1.995977000	-0.806616000
6	-4.121886000	2.365206000	0.323786000
1	-4.032515000	2.722318000	1.321394000

Table S167. Predicted frequencies (cm^{-1}) and IR intensities (km/mol) of isomer 6e-doublet of $Pt^+(C_2H_2)_5$

Frequency	Intensity	Frequency	Intensity	Frequency	Intensity
11.0113	0.0532	320.0763	2.6004	813.8433	31.7174
17.7774	0.1158	346.2621	2.313	818.5237	3.7781
21.8343	0.0555	351.6088	2.5486	833.889	5.8079
31.8384	0.1134	417.4898	3.0375	841.2512	10.665
35.5774	0.0268	662.5384	0.0726	851.4163	30.5056
49.9625	0.0417	667.8519	0.0828	1844.2287	7.3052
58.9565	0.0784	674.7954	0.0803	1903.6557	7.131
61.8432	0.3389	676.1102	0.2635	1909.5818	2.0935
73.1567	3.6818	683.5016	0.3103	1957.8804	25.6762
80.9096	1.6298	687.6224	1.6281	2061.7797	8.4436
84.8222	6.0304	707.0095	0.1702	2064.1154	5.968
91.0456	6.6334	717.8431	7.7508	3292.5664	476.4043
94.6809	1.278	721.5575	2.6641	3327.8508	64.6652
111.6957	2.4467	723.8202	0.632	3352.3457	53.3905
120.8322	1.7865	736.5992	234.2932	3353.6655	129.7019
127.01	4.4227	746.3869	9.0924	3395.9146	69.2749
133.565	4.9711	750.8681	13.3078	3396.6597	104.1413
134.5772	4.1135	763.018	3.4815	3397.7079	23.1895
161.2141	0.8651	772.4921	18.5289	3401.5665	103.1791
168.4469	2.8047	773.985	74.6982	3430.6831	55.3548
193.2291	0.86	779.4722	79.872	3431.9908	19.4604
224.7242	0.7431	786.7371	155.2154	3500.9074	1.248
244.9848	0.9999	789.909	84.3914	3505.4728	0.8708

Table S168. Cartesian coordinates for the optimized geometry of isomer 6f-doublet of $Pt^{+}(C_2H_2)_5$ followed by its predicted frequencies (cm^{-1}) and IR intensities (km/mol).

Z	x	y	z
78	-0.432478000	-0.183852000	0.043583000
1	-0.487338000	0.010115000	3.028306000
6	1.227330000	-0.041514000	1.552175000
6	0.172177000	-0.033215000	2.189740000
1	2.273669000	-0.016845000	1.325399000
1	-2.943835000	0.421691000	-1.497730000
6	-2.572599000	-0.013792000	0.691825000
6	-2.577258000	0.190933000	-0.522184000
1	-2.934270000	-0.133594000	1.688863000
1	-0.519765000	0.357987000	-2.917542000
6	0.175230000	0.159656000	-2.133186000
6	1.228746000	-0.016633000	-1.530229000
1	2.266348000	-0.111622000	-1.287606000
1	0.182668000	-2.846532000	1.133559000
6	-0.330598000	-2.587679000	0.235791000
6	-0.934989000	-2.608323000	-0.815738000
1	-1.465996000	-2.673548000	-1.735888000
6	-0.020240000	3.528984000	-0.535526000
6	-0.857909000	3.549831000	0.319935000
1	-1.602443000	3.608258000	1.075514000
1	0.720898000	3.545733000	-1.296304000
1	4.637552000	1.617590000	0.109933000
6	4.630407000	0.555463000	0.056058000
6	4.669209000	-0.640275000	-0.003634000
1	4.748720000	-1.699402000	-0.056150000

Table S169. Predicted frequencies (cm^{-1}) and IR intensities (km/mol) of isomer 6f-doublet of $Pt^+(C_2H_2)_5$

Frequency	Intensity	Frequency	Intensity	Frequency	Intensity
12.153	0.0819	312.145	1.6264	790.6486	133.5895
19.2662	0.8251	349.64	2.9572	799.2132	23.9462
32.9545	0.3183	378.1509	2.7501	819.565	2.622
39.0523	0.3905	382.568	3.8141	831.8808	4.6624
41.0409	0.5791	662.2523	0.8054	858.5966	7.3616
45.088	0.3261	663.0717	0.761	1871.6171	5.4259
48.9985	0.2491	668.0902	0.0192	1884.7534	10.6567
62.9803	0.5597	671.049	0.0451	1905.0992	5.7713
67.5389	0.9385	679.3744	0.1509	1974.2966	13.6388
71.7913	0.2263	692.7379	0.7253	2061.5711	9.6452
88.9287	7.2893	697.559	9.0566	2062.6545	16.5942
92.0572	5.7278	705.1748	4.1052	3305.4729	131.1369
93.5352	7.6727	712.9704	6.1296	3321.1003	280.9876
105.416	3.7036	724.3803	84.9785	3343.0329	72.0283
109.9272	3.3457	744.542	15.342	3374.0548	116.2717
125.0379	0.4128	759.9324	71.3963	3396.0177	110.5216
139.8602	1.9456	762.4431	7.5661	3396.6247	52.3412
145.504	1.7343	765.3914	11.9589	3408.0143	86.3978
151.9666	0.4079	772.7004	67.6087	3414.3628	54.4662
157.5699	0.673	776.1258	201.6695	3417.5992	31.5306
186.1166	0.4811	777.7266	71.4766	3468.4514	19.2037
232.7809	0.9629	780.2689	21.7389	3499.847	1.063
247.1205	1.7955	785.7496	26.74	3510.7152	0.1775

Table S170. Cartesian coordinates for the optimized geometry of isomer 6a-quartet of $Pt^{+}(C_2H_2)_5$ followed by its predicted frequencies (cm^{-1}) and IR intensities (km/mol).

Z	x	y	z
78	0.313203000	-0.619136000	-0.179703000
1	-2.186688000	-1.190140000	1.338450000
6	0.027849000	-1.677934000	1.653884000
6	-1.116915000	-1.307178000	1.277728000
1	0.708109000	-2.170131000	2.324464000
1	-0.603198000	2.175725000	-0.513052000
6	1.390445000	1.234177000	0.125908000
6	0.234159000	1.549521000	-0.244731000
1	2.409875000	1.395125000	0.443937000
1	-0.821843000	-1.417041000	-2.836372000
6	-0.009225000	-1.441281000	-2.133641000
6	1.145149000	-1.787282000	-1.751645000
1	2.083693000	-2.294665000	-1.885021000
1	-3.626971000	2.263968000	-0.173747000
6	-3.052200000	2.957580000	-0.740450000
6	-2.433717000	3.757840000	-1.382188000
1	-1.916509000	4.488332000	-1.956203000
6	-4.755238000	-0.363784000	0.910869000
6	-4.438109000	0.198617000	1.919736000
1	-4.193696000	0.699053000	2.825594000
1	-5.075933000	-0.860598000	0.027245000
1	5.329955000	1.778891000	-0.375319000
6	4.958503000	1.844212000	0.618568000
6	4.579927000	1.934604000	1.751045000
1	4.279575000	2.029537000	2.766355000

Table S171. Predicted frequencies (cm^{-1}) and IR intensities (km/mol) of isomer 6a-quartet of $Pt^+(C_2H_2)_5$

Frequency	Intensity	Frequency	Intensity	Frequency	Intensity
9.4709	0.0023	321.5657	2.9145	799.9989	195.2819
14.7312	0.0346	379.525	1.8251	808.337	31.7746
15.9876	0.0396	400.9984	0.4914	815.9049	2.095
25.4279	0.1869	442.8776	0.1028	821.1918	36.9523
29.4428	0.029	660.2821	0.0213	870.0564	1.1059
34.0326	0.2158	667.2809	0.3182	1677.7182	12.123
35.5606	0.0132	673.5206	12.8347	1705.0267	0.5111
39.6701	0.1335	675.1505	1.6868	1783.1608	6.2491
53.9737	1.7415	676.7653	5.8168	2059.822	9.2553
67.6078	3.5015	678.2158	0.6015	2062.1259	5.2282
72.5126	3.4103	683.0134	6.3445	2063.4699	9.4354
81.5233	2.8086	687.565	11.9356	3170.3515	689.2938
83.7697	3.4969	712.1843	36.2702	3221.7744	281.5916
87.787	0.8144	741.9545	48.8977	3246.0665	292.7188
93.9501	4.0449	750.5266	35.5563	3253.0434	63.0245
98.439	2.5498	771.3818	50.9024	3303.3841	66.8923
105.7103	13.8777	776.3137	31.9523	3313.1489	29.6701
119.9538	5.1609	778.3419	65.4533	3388.2476	151.4408
127.8244	0.2404	781.0749	49.4729	3397.6574	104.5612
182.6704	0.4129	787.0504	93.8094	3400.4191	108.2086
198.1289	0.5381	789.2246	21.3499	3494.7308	0.1644
258.1014	5.8415	790.0192	163.5011	3501.699	0.8952
277.8167	2.1201	796.2371	62.5104	3504.088	0.7847

Table S172. Cartesian coordinates for the optimized geometry of isomer 6b-quartet of $Pt^{+}(C_2H_2)_5$ followed by its predicted frequencies (cm^{-1}) and IR intensities (km/mol).

Z	x	y	z
78	0.299768000	-0.597479000	-0.144451000
1	-2.411657000	4.157098000	0.792073000
6	-2.824819000	3.356326000	-1.281410000
6	-2.594565000	3.764305000	-0.179036000
1	-3.052741000	3.027263000	-2.266403000
1	0.533423000	-1.962376000	2.485827000
6	-1.214029000	-1.102660000	1.311916000
6	-0.104680000	-1.495057000	1.759080000
1	-2.279675000	-0.927786000	1.292426000
1	2.524093000	1.313835000	0.349665000
6	1.495509000	1.188219000	0.044288000
6	0.360313000	1.554636000	-0.351061000
1	-0.417288000	2.238670000	-0.647824000
1	4.640333000	1.535056000	2.566934000
6	4.821353000	1.644909000	1.525251000
6	5.067649000	1.783614000	0.361593000
1	5.321933000	1.919268000	-0.661695000
6	1.073476000	-1.962830000	-1.582768000
6	-0.047449000	-1.576491000	-2.021049000
1	-0.846081000	-1.565252000	-2.739925000
1	1.977366000	-2.541647000	-1.646063000
6	-4.506270000	0.280371000	0.805183000
6	-4.851453000	-0.786440000	1.226472000
1	-5.193575000	-1.721286000	1.600123000
1	-4.228255000	1.237114000	0.431291000

Table S173. Predicted frequencies (cm^{-1}) and IR intensities (km/mol) of isomer 6b-quartet of $Pt^+(C_2H_2)_5$

Frequency	Intensity	Frequency	Intensity	Frequency	Intensity
10.5362	0.0036	316.2824	2.9423	799.2899	201.1604
14.6372	0.0222	383.133	0.9922	812.115	6.6406
16.1762	0.0595	397.9231	1.0176	814.1521	39.6069
26.192	0.1106	442.6624	0.1071	820.7843	31.9046
30.6174	0.0657	660.2903	0.0144	871.9691	1.2171
32.2069	0.0525	667.2267	0.4879	1680.4331	10.2852
35.9254	0.098	673.8358	9.6762	1702.2784	4.4812
38.2185	0.1004	674.9775	2.6108	1782.1539	3.4512
55.1429	2.1989	677.6027	1.4367	2059.9731	9.2321
61.2338	2.0574	678.4952	1.6062	2062.1019	4.5564
80.2722	3.4099	683.1311	13.5031	2063.4419	10.2284
82.9891	1.6874	686.6854	3.4643	3180.9559	677.8883
86.0084	1.7352	717.2124	20.8536	3191.9792	374.996
86.6692	1.0333	732.8389	73.837	3253.3657	68.7754
90.3312	4.5369	752.5101	19.7725	3264.7481	219.6346
100.6917	17.1272	770.193	25.1235	3304.9853	64.2138
106.9132	7.3351	773.2946	67.2494	3313.5682	29.3624
111.9436	0.7818	779.1728	117.8025	3388.9952	150.7448
130.9944	0.5989	781.24	11.7093	3397.604	103.7716
181.9129	0.2288	786.9936	107.09	3400.4419	108.6276
197.2545	0.568	789.9997	133.4565	3495.2827	0.1256
257.879	5.9358	791.489	68.0822	3501.6607	0.8577
284.3476	2.5121	793.4219	11.8781	3504.1293	0.8085

Table S174. Cartesian coordinates for the optimized geometry of isomer 6c-quartet of $Pt^{+}(C_2H_2)_5$ followed by its predicted frequencies (cm^{-1}) and IR intensities (km/mol).

Z	x	y	z
78	0.784604000	-0.035176000	0.126648000
1	2.046028000	1.545470000	-2.108952000
6	3.008827000	0.895455000	-0.159856000
6	2.387513000	1.185750000	-1.165008000
1	3.682573000	0.750459000	0.653580000
1	-1.028132000	-1.716091000	-1.449237000
6	1.241692000	-1.737039000	-1.573431000
6	0.037217000	-1.596105000	-1.426033000
1	2.258096000	-1.936230000	-1.827335000
1	-1.420643000	1.551189000	0.264083000
6	-0.348572000	1.644357000	0.061909000
6	0.178257000	2.855329000	-0.079419000
1	-0.174828000	3.877491000	-0.013841000
1	-1.333252000	-1.418943000	1.542200000
6	-0.301952000	-1.118965000	1.573879000
6	0.844379000	-1.082699000	2.070483000
1	1.632476000	-1.209239000	2.783263000
6	-3.557214000	-1.745490000	-0.331213000
6	-3.195595000	-2.887199000	-0.286289000
1	-2.914734000	-3.912240000	-0.248228000
1	-3.913849000	-0.741479000	-0.366342000
1	-4.229663000	2.174336000	-1.724327000
6	-4.334606000	2.029537000	-0.676590000
6	-4.485557000	1.889437000	0.502992000
1	-4.649486000	1.784801000	1.548064000

Table S175. Predicted frequencies (cm^{-1}) and IR intensities (km/mol) of isomer 6c-quartet of $Pt^{+}(C_2H_2)_5$

Frequency	Intensity	Frequency	Intensity	Frequency	Intensity
18.1065	0.0469	269.4413	3.1082	812.3527	33.1744
24.9373	0.0605	294.5094	2.9773	842.9895	34.4352
31.4385	0.1562	410.3435	1.2721	859.6538	10.1902
33.4504	0.136	493.6407	8.4762	875.0425	3.2746
44.0953	0.5216	559.8561	44.9046	1164.588	4.7083
47.6519	0.1509	660.3766	1.5221	1465.7259	130.1886
65.3061	0.2213	667.7585	0.0616	1772.2994	4.4058
66.5092	0.1163	673.5089	0.768	1915.8301	6.3895
67.885	0.6814	681.5293	9.624	1948.6564	1.5344
72.2075	2.0264	687.0414	5.6415	2055.3001	9.2559
75.0598	1.7827	691.4954	3.9128	2063.809	5.1241
94.1218	1.8231	697.7131	26.9209	3006.5046	3.4806
104.512	2.4537	700.1078	23.9642	3175.3312	0.4959
108.4232	0.7739	705.0522	3.6752	3271.9159	96.6718
121.5672	0.7654	730.1957	17.5439	3300.2255	291.9563
125.9998	2.9808	749.8411	49.499	3362.7587	30.7412
126.4424	0.8588	763.5438	22.4389	3365.0358	154.3124
148.5791	4.2475	776.2801	60.2003	3370.6517	193.116
155.3466	2.0095	776.8087	3.8226	3401.154	103.0639
177.8142	4.673	783.0761	151.9218	3425.7807	52.472
188.4826	3.3984	784.72	49.9707	3451.4424	37.3917
209.7686	2.3257	803.2385	102.6604	3484.7156	0.6815
247.6137	4.6996	806.9492	94.9718	3504.9147	0.902

Table S176. Cartesian coordinates for the optimized geometry of isomer 6d-quartet of $Pt^{+}(C_2H_2)_5$ followed by its predicted frequencies (cm^{-1}) and IR intensities (km/mol).

Z	x	y	z
78	-0.419073000	-0.514498000	0.127126000
1	1.046141000	-1.721041000	-2.199167000
6	0.448420000	-2.736677000	-0.260619000
6	0.711985000	-2.080522000	-1.252243000
1	0.326089000	-3.440789000	0.530639000
1	-2.123729000	1.348878000	-1.373767000
6	-2.208496000	-0.918365000	-1.500172000
6	-2.028079000	0.280961000	-1.354861000
1	-2.442166000	-1.929011000	-1.747441000
1	1.210264000	1.641726000	0.345912000
6	1.276120000	0.589528000	0.050151000
6	2.469661000	0.061572000	-0.198636000
1	3.505364000	0.385516000	-0.163900000
1	-1.662574000	1.622009000	1.645471000
6	-1.407069000	0.578782000	1.637220000
6	-1.392611000	-0.574829000	2.116937000
1	-1.515790000	-1.371562000	2.820524000
6	-2.093075000	3.937035000	-0.494592000
6	-3.191300000	3.549122000	-0.214948000
1	-4.180430000	3.243444000	0.028471000
1	-1.135924000	4.325364000	-0.746673000
1	5.980158000	1.839115000	-1.623339000
6	5.986252000	1.568204000	-0.595950000
6	6.026375000	1.273051000	0.563398000
1	6.091160000	1.020017000	1.593341000

Table S177. Predicted frequencies (cm^{-1}) and IR intensities (km/mol) of isomer 6d-quartet of $Pt^{+}(C_2H_2)_5$

Frequency	Intensity	Frequency	Intensity	Frequency	Intensity
10.5674	0.004	277.3794	3.0857	809.4151	7.1786
15.7927	0.0111	292.7831	1.6797	845.5363	34.9451
18.6323	0.069	410.8464	1.3044	860.5494	9.5401
20.2217	0.0812	500.2526	10.5828	884.2375	1.3202
31.352	0.0263	582.4559	35.0544	1158.3029	5.5708
39.0989	0.3449	658.4212	0.0148	1459.2646	175.7349
51.4012	1.9809	659.6691	1.9104	1773.2749	4.8031
51.6088	0.3714	668.1224	0.0482	1916.078	7.7197
66.6967	1.094	673.2683	2.123	1941.8422	1.1815
69.0907	0.7198	677.1915	2.058	2061.6218	8.6396
73.7297	2.2601	683.2991	9.6231	2066.1249	6.867
83.4907	0.3239	696.6653	26.8792	3001.1127	9.6445
93.0804	1.3177	699.6705	25.9821	3143.7858	109.3891
100.6582	2.2617	706.5316	3.4835	3277.7208	74.21
118.3165	1.1182	727.8314	17.8495	3298.8926	308.4787
122.5122	2.3541	747.0813	49.1352	3362.6368	101.8767
126.6735	1.2641	756.4509	35.7826	3364.8026	86.6915
149.8176	4.1247	770.3676	75.6486	3396.6812	104.4674
152.1993	2.9161	775.7443	13.4239	3405.4641	100.5086
179.0114	4.1198	778.1016	70.3643	3425.9567	56.0871
187.919	3.0865	782.4205	176.6216	3448.3711	38.4034
210.3084	2.7233	784.1282	88.0378	3501.0366	1.1619
253.1109	4.4516	790.2753	94.0949	3509.1351	0.9313

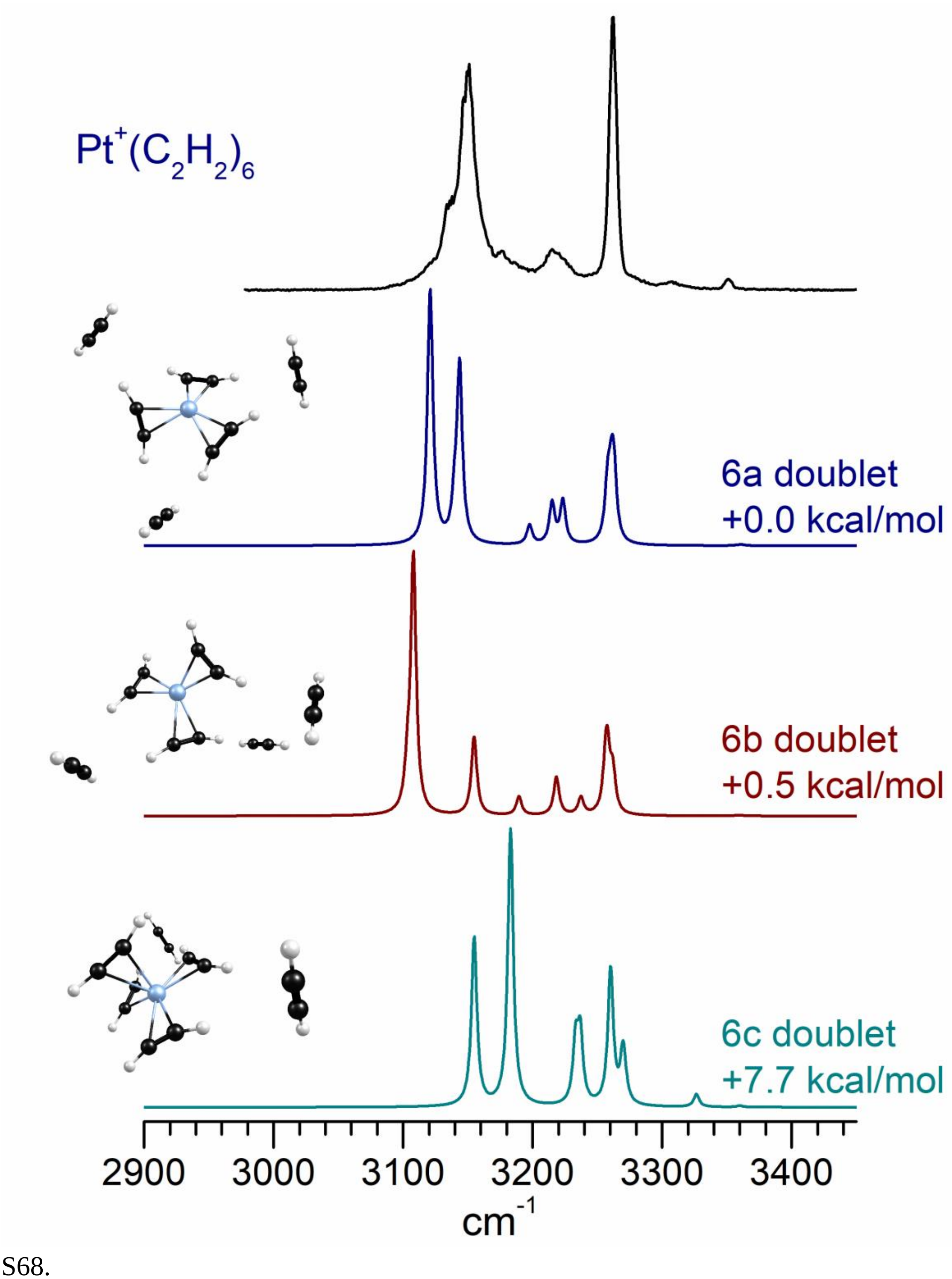

$Pt^+(C_2H_2)_6$
6a doublet
+0.0 kcal/mol
6b doublet
+0.5 kcal/mol
6c doublet
+7.7 kcal/mol
2900
3000
3100
3200
3300
3400
cm^{-1}

S68.

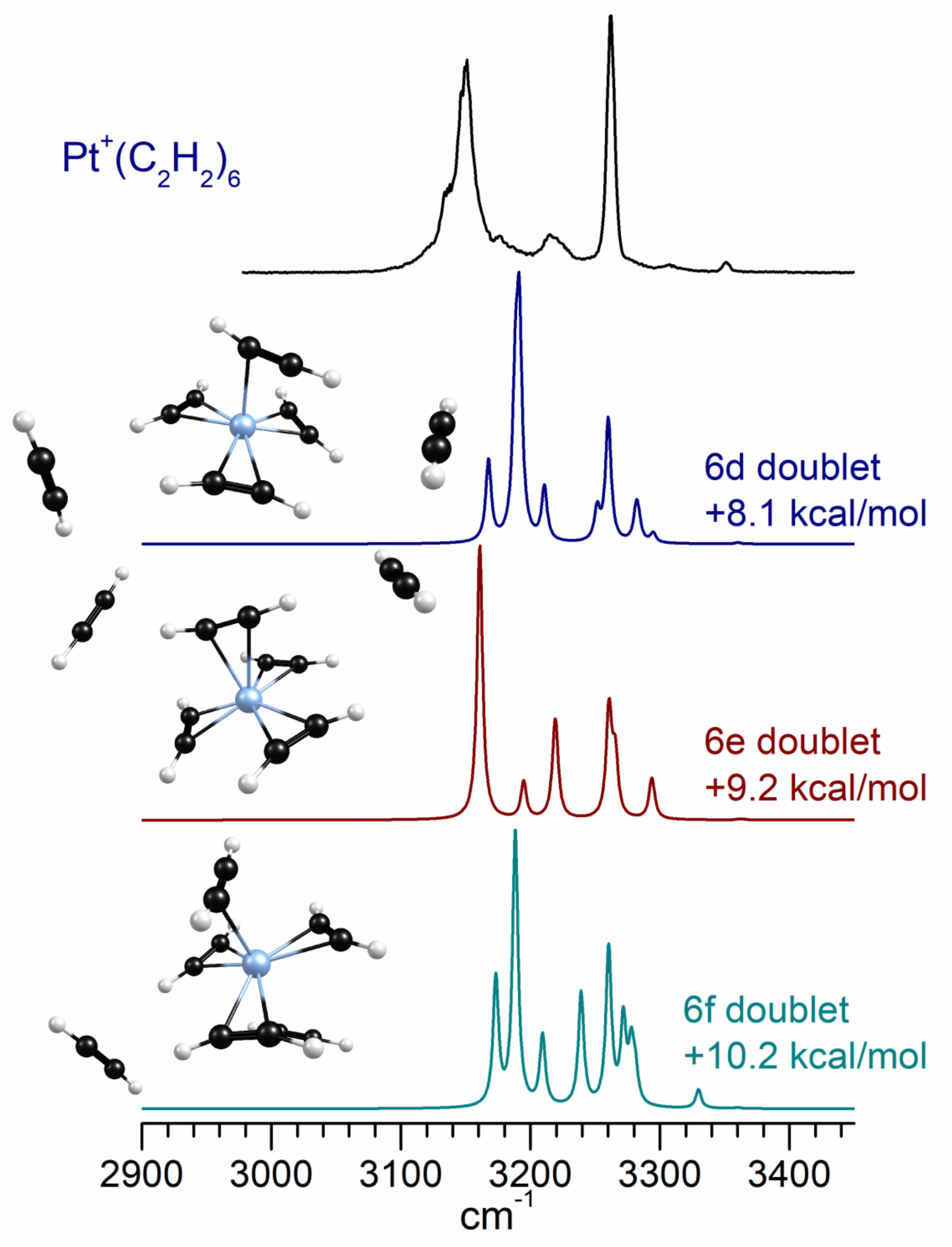
Pt+(C2H2)6
6d doublet
+8.1 kcal/mol
6e doublet
+9.2 kcal/mol
6f doublet
+10.2 kcal/mol
2900
3000
3100
3200
3300
3400
cm-1

S69.
